# Hepatocyte TIA1 constrains metabolic steatohepatitis by translationally suppressing Srebf1 mRNA in stress granules

**DOI:** 10.1038/s41419-026-08682-5

**Published:** 2026-03-24

**Authors:** Rong Liu, Jiaojiao Chen, Jinguang Wang, Ti Zhang, Yujing Xia, Jiao Feng, Chuanyong Guo, Lei Xue, Yingqun Zhou

**Affiliations:** 1 Department of Gastroenterology, Shanghai Tenth People’s Hospital, Tongji University School of Medicine, Shanghai, China; 2 Department of Gastroenterology, Shidong Hospital Affiliated to University of Shanghai for Science and Technology, Shanghai, China; 3 Department of Gastroenterology, The First People’ s Hospital of Bengbu, Bengbu City, Anhui Province China; 4 Department of Hepatobiliary Surgery, Shanghai Tenth People’s Hospital, Tongji University School of Medicine, Shanghai, China; 5 Department of Nuclear Medicine, Shanghai Tenth People’s Hospital, Tongji University School of Medicine, Shanghai, China

**Keywords:** Cell biology, Metabolic disorders

## Abstract

Metabolic dysfunction-associated steatotic liver disease (MASLD) and its inflammatory sequel, metabolic dysfunction-associated steatohepatitis (MASH), pose escalating global health burdens, underscoring the urgent need to elucidate their molecular mechanisms and identify novel therapeutic targets. T-cell intracellular antigen 1 (TIA1), an RNA-binding protein and core organizer of stress granules (SGs), regulates post-transcriptional gene expression during cellular stress. However, its functional role in MASLD pathogenesis remains poorly understood. Hepatocyte-specific TIA1-knockout (TIA1-HKO) and wild-type control mice were subjected to three distinct diet-induced MASLD models. Parallel gain- and loss-of-function studies were conducted in PA-treated AML12 hepatocytes. RNA immunoprecipitation sequencing (RIP-seq), RIP-qPCR, fluorescence in situ hybridization (FISH), dual-luciferase reporter assays, and mRNA stability measurements were employed to map TIA1-sterol regulatory element binding transcription factor 1 (*Srebf1*) mRNA interactions and quantify translational repression. Pharmacological and genetic rescue experiments confirmed mechanistic findings. Integrated transcriptomic analysis of clinical specimens and murine models revealed significant TIA1 upregulation during MASLD progression. Hepatocyte-specific TIA1 deletion exacerbated dietary-induced steatosis, inflammation, and fibrosis. In vitro, TIA1 was essential for SGs assembly and maintenance of lipid homeostasis under lipotoxic stress. Mechanistically, TIA1 directly binds the 3’ UTR of *Srebf1* mRNA, sequestering it within SGs and repressing the translation of sterol regulatory element binding protein 1 (SREBP1)—a master transcriptional regulator of lipogenesis. Inhibition of SREBP1 activity rescued the metabolic perturbations induced by TIA1 ablation. This study identifies TIA1 as a crucial hepatoprotective factor that attenuates MASLD progression by orchestrating SGs-dependent translational control of *Srebf1* mRNA. Impairment of the TIA1-SGs-SREBP1 axis accelerates steatohepatitis, highlighting its potential as a therapeutic target for metabolic liver diseases.

**Figure:**
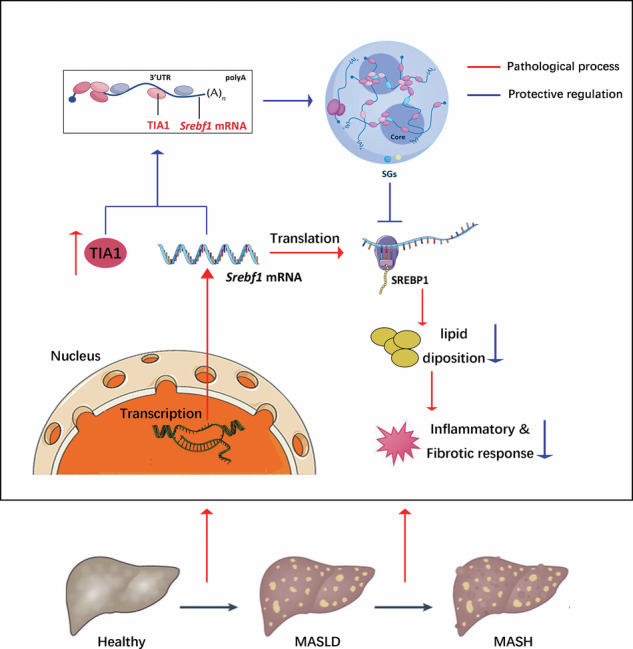
**TIA1 Constrains MASH Progression by Assembling Stress Granules to Suppress SREBP1-Driven Lipogenesis**. This study delineates a hepatoprotective pathway centered on the RNA-binding protein TIA1. In response to metabolic stress. TIA1 nucleates SGs assembly and sequesters *Srebf1* mRNA, leading to translational repression of the master lipogenic transcription factor SREBP1 and its downstream lipogenic program, thereby mitigating steatosis and subsequent inflammatory and fibrotic response.

**TIA1 Constrains MASH Progression by Assembling Stress Granules to Suppress SREBP1-Driven Lipogenesis**. This study delineates a hepatoprotective pathway centered on the RNA-binding protein TIA1. In response to metabolic stress. TIA1 nucleates SGs assembly and sequesters *Srebf1* mRNA, leading to translational repression of the master lipogenic transcription factor SREBP1 and its downstream lipogenic program, thereby mitigating steatosis and subsequent inflammatory and fibrotic response.

## Introduction

Given numerous limitations associated with the terminology nonalcoholic fatty liver disease (NAFLD), an international expert panel recommended standardizing its definition and redefining it as metabolic dysfunction-associated steatotic liver disease (MASLD) in 2020 [1, 2]. This condition is progressively emerging as the predominant chronic liver disorder owing to its substantial global prevalence and propensity to progress toward metabolic dysfunction-associated steatohepatitis (MASH), hepatic fibrosis, and ultimately hepatocellular carcinoma. MASLD fundamentally stems from disrupted energy homeostasis characterized by excessive caloric consumption relative to inadequate expenditure, which manifesting as metabolic dysregulation, pathological lipid deposition, and sustained inflammatory responses [3]. Dysregulated lipid metabolism promotes triglyceride accumulation within hepatocytes, initiating constant lipotoxicity as a pivotal mediator of hepatic injury, which subsequently induces endoplasmic reticulum (ER) perturbation and oxidative burden, aggravating hepatocellular damage and inflammatory cascades, thereby establishing a metabolic environment conducive to carcinogenesis [4]. Additionally, MASLD shares pathophysiological parallels with obesity-related metabolic syndrome, predisposing to multisystem complications via diverse extrahepatic mechanisms, including diabetes mellitus, cardiovascular disorders, and hypertension [5]. The considerable prevalence, elevated mortality risk, and multi-organ complications collectively signify that MASLD-associated hepatic impairment and its sequelae will impose substantial healthcare burdens and socioeconomic strains on affected individuals, families, and communities. While Rezdiffra (Resmetriom) remains the solitary Food and Drug Administration (FDA)-endorsed therapeutic agent, functioning as a selective thyroid hormone receptor-beta agonist that partially ameliorates steatosis [6], critical knowledge gaps persist regarding transcriptional regulation and signal transduction in MASLD pathogenesis, necessitating novel intervention strategies.

Aberrant post-transcriptional gene expression modulation significantly underpins diverse pathological states spanning metabolic dysfunctions, inflammatory conditions, and malignancies [7]. Recently, RNA-binding protein (RBP)-mediated pathogenic mechanisms in MASLD have garnered escalating interest. RBPs, metaphorically designated as molecular custodians of MASLD, critically influence disease progression through multifaceted RNA regulatory processes encompassing transcriptional modulation, alternative splicing patterns, polyadenylation site selection, transcript stability control, and subcellular trafficking [8]. In particular, dysregulation of specific RNA-binding proteins, notably adenylate-uridylate-rich element binding proteins (AUBPs), has been implicated in MASLD, MASH, and hepatocarcinogenesis [9, 10]. Upon interacting with adenylate-uridylate-rich elements (AREs) located within mRNA 3’-untranslated regions (3’-UTRs), these proteins govern messenger RNA degradation dynamics and translational efficiency, potentially resulting in anomalous oncogene overexpression, inflammatory mediator elevation, or conversely, tumor suppressor silencing [11]. T-cell intracellular antigen 1 (TIA1), is a canonical RBP that promotes the assembly of stress granules (SGs), discrete cytoplasmic inclusions into which stalled translation initiation complexes are dynamically recruited in cells subjected to environmental stress [12]. These non-membranous organelles assemble via liquid-liquid phase separation, wherein sequestered mRNAs undergo translational arrest before being sorted for either reinitiation of protein synthesis or targeted decay, contingent upon specific stress contexts [13]. Notably, accumulated evidence indicates the responsiveness of SGs to cellular metabolic status, suggesting their coordination of adaptive stress responses during metabolic perturbations [14, 15]. Pioneering work by Amen and Kaganovich elucidated a regulatory paradigm wherein stress granules govern metabolic stress adaptation; specifically, SGs assembly restricts mitochondrial fatty acid import through modulation of the voltage-dependent anion channel (VDAC) porin [16].

It has been well established that TIA1 plays a central role in promoting SGs assembly both in vivo and in vitro [17–19]. Dysregulation of TIA1 and dysfunction of SGs are well recognized as important pathological factors in neurodegenerative diseases [20]. Recently, TIA1 was also demonstrated to have established connections to inflammatory signaling and multiple disease processes. Prior investigations have proposed TIA1-dependent SGs biogenesis involvement in acute liver injury and hepatocellular carcinoma pathogenesis [21–23]. Nevertheless, the mechanistic contributions, functional significance, and regulatory control of TIA1 and SGs formation during MASLD/MASH progression remain incompletely defined. To address these uncertainties, we systematically examined the integrated in vivo and in vitro functions of TIA1 in hepatic steatosis, inflammatory activation, and fibrogenesis within MASLD/MASH contexts. Furthermore, we delineated potential TIA1’s downstream effector molecules contributing to its pathophysiological impact and evaluated its regulatory influence on SGs dynamics in metabolically stressed hepatocytes.

## Materials And Methods

Full details are available in Supplementary Materials and Methods.

### Human liver samples

A total of 5 patients with biopsy-confirmed MASLD and 5 healthy controls were consecutively recruited at the Shanghai Tenth People’s Hospital, Tongji University School of Medicine. Normal liver tissues were obtained during gallbladder exclusion excision surgery due to gallbladder stones. Patients with MASLD were diagnosed by the presence of ≥ 5% hepatic steatosis. Exclusion criteria included known acute or chronic viral hepatitis, excessive alcohol ingestion, use of pharmacological treatments (hepatic protectants or hepatotoxic agents), chronic kidney disease, serious cardiovascular or cerebrovascular disease, severe gastrointestinal diseases or gastrointestinal surgery, history of malignant tumor. All studies were approved by the Human Subjects Review Committee, Office of Research Services, School of Medicine, Tongji University and conducted in accordance with the 1975 Declaration of Helsinki (SHSY-IEC-KY-4.0/18-/01). Written informed consent was obtained from each participant. The physical and biochemical parameters are summarized in Table S1.

### Animal studies

Male C57BL/6 J wild-type mice (8–10 weeks old) were obtained from Shanghai Jihui Experimental Animal Breeding Co., Ltd. (Shanghai, China). Global TIA1-knockout mice (Strain S-KO-05415) were purchased from Cyagen. Hepatocyte-specific TIA1-knockout (TIA1-HKO) mice were generated by crossing TIA1fl/fl (TIA1-Flox) mice with albumin-Cre transgenic mice. TIA1-Flox littermates were used as controls for TIA1-HKO mice. All animals were housed under specific pathogen-free (SPF) conditions at 22–24 °C with a 12 h light/dark cycle and provided food and water ad libitum. Mice were randomly assigned to experimental groups. After acclimatization, TIA1-Flox and TIA1-HKO mice were fed a high-fat diet (HFD; D12492, Research Diets, 60% kcal from fat) for 12 weeks to induce hepatic steatosis. To establish MASH models, male mice were fed either a high-fat high-cholesterol diet (HFHC; D09100310, Research Diets, 40 kcal% fat, 20 kcal% fructose, 2% cholesterol) for 20 weeks or a methionine-choline-deficient diet (MCD; Research Diets, A02082002B, 16 kcal% protein, 63 kcal% carbohydrate, 21 kcal% fat, 0% L-methionine, 0% choline bitartrate) for 8 weeks. These well documented diets represent well-established models that recapitulate key features of human MASLD/MASH progression [24]. Control mice received control diet (CD; D09100304; Research Diets) (*n* = 10 mice per group).

For AAV8-mediated gene delivery, adult male C57BL/6 J mice (8–10 weeks old) were injected via the tail vein with 100 μL of AAV8-TBG-mTIA1 (1 × 10^11^ vector genomes [vg]/mouse; Vector Biolabs) under isoflurane anesthesia (2–3% in oxygen). Control mice received AAV8-TBG-GFP (Vector Biolabs) at the same titer (*n* = 10 mice per group).

For pharmacological inhibition of SREBP1, TIA1-HKO and TIA1-Flox mice received intraperitoneal injections of either saline or PF-429242 (HY-13447, MedChemExpress; 20 mg/kg), a site-1 protease inhibitor, every other day for the final eight weeks of HFD feeding (*n* = 10 mice per group). PF-429242 was dissolved in saline with sonication to ensure complete dissolution.

At the end of the experiment, mice were anesthetized and euthanized by cervical dislocation. Blood was collected via retro-orbital puncture, and serum was separated by centrifugation at 3000 rpm for 10 min at 4 °C (Centrifuge 5340 R, Eppendorf) and stored at −80 °C. Liver tissues were either fixed in 10% neutral buffered formalin for histology or snap-frozen in liquid nitrogen and stored at −80 °C for further analysis.

All animal procedures were approved by the Animal Care Committee of Shanghai Tenth People’s Hospital, Tongji University School of Medicine (Ethics Approval No. #SHDSYY-2025-1474) and conducted in accordance with the Guide for the Care and Use of Laboratory Animals.

### Cell culture and in vitro experimental design

AML12 murine hepatocytes and HEK293T cells were obtained from the Cell Bank of the Chinese Academy of Sciences (Chinese Academy of Sciences, Shanghai, China). AML12 cells were cultured in Dulbecco’s Modified Eagle’s medium/F12 (DMEM/F12) medium (Gibco) supplemented with 10% fetal bovine serum (FBS), insulin-transferrin-selenium solution (10 μg/mL insulin, 5.5 μg/mL transferrin, 5 ng/mL selenium; Invitrogen), 40 ng/mL dexamethasone (Sigma-Aldrich), and 1% penicillin-streptomycin. HEK293T cells were maintained in high-glucose DMEM (Gibco) containing 10% FBS. All cells were incubated at 37 °C in a humidified atmosphere of 5% CO₂ and passaged every 2–3 days.

Palmitic acid (PA; Sigma-Aldrich, P0500) was conjugated with 0.5% fatty acid-free bovine serum albumin (BSA, Sigma-Aldrich) as previously described [25]. As a preliminary experiment, AML12 cells were treated with BSA (control, BSA), PA (400 μM), a combination of PA and oleic acid (PA + OA, 200 μM each), or OA alone (400 μM). To induce lipotoxic stress, AML12 cells were treated with BSA-conjugated PA at concentrations ranging from 100 to 500 μM for 12–48 h; control cells received an equivalent volume of BSA vehicle. For SREBP1 inhibition, cells were pretreated with 10 μM PF-429242 (HY-13447, MedChemExpress) for 1 h prior to PA exposure. To modulate SGs formation, cells were previously exposed to either 0.5 mM sodium arsenite (Ars; Sigma-Aldrich, S7400) for 1 h to induce SGs assembly, or 25 ng/mL anisomycin (An; Yuanye Biotechnology, S17105) for 30 min to inhibit SG formation, followed by co-incubation with PA for 24 h.

### Immunofluorescence staining and confocal microscopy

The cells were fixed in 4% PFA for 15 min and permeabilized with 0.3% Triton X-100 at RT for 10 min. After blocking with 3% BSA for 1 h, the cells were incubated overnight at 4 °C with 1:200 diluted anti-TIA1 (12133-2-AP, Proteintech, Wuhan, China), anti-G3BP stress granule assembly factor 1 (G3BP1, 13057-2-AP, Proteintech, Wuhan, China) or anti-Albumin antibodies (16475-1-AP, Proteintech, Wuhan, China). The following day, cells were incubated with fluorophore-conjugated secondary antibody against rabbit (Alexa Fluor® 488 goat anti-rabbit IgG (H + L), ab150077, Abcam Inc., Cambridge, MA, USA) or mice (Alexa Fluor® 594 goat anti-mouse IgG (H + L), ab150116, Abcam Inc., Cambridge, MA, USA) at RT for 1 h. To assess fatty acid uptake, AML12 cells were incubated with the non-metabolizable fluorescent fatty acid BODIPY FL C16 (100 nM in PBS, D3821, Thermo Fisher Scientific) for 15 min at room temperature. Nuclei in all images were stained with DAPI (S36939; Invitrogen; Carlsbad, CA, USA). High-resolution z-stack images (with a step size of 0.3–0.5 µm) were acquired using a 63x oil immersion objective on a confocal laser scanning microscope (e.g., Zeiss LSM 880 or equivalent). Identical laser power, gain, offset, and pinhole settings were applied across all experimental groups within the same experiment to enable comparative quantification.

### Quantification of stress granules via manual counting

The manual quantification workflow we employed is referenced and adapted from previous literature [26–30].

### Image pre-processing

All immunofluorescence images were analyzed using ImageJ (Fiji distribution, version 1.54 f). Original confocal microscope images (.czi, .tiff, or .lif formats) were imported into the software. To accurately analyze specific signals, the channels corresponding to the stress granule markers (TIA1 and G3BP1) were retained. Images were converted to 8-bit grayscale using the Image > Type > 8-bit command for subsequent intensity analysis.

### Definition of analysis region

To ensure counting was performed at the single-cell level, the cytoplasmic area of individual cells was first defined. The channel image corresponding to nuclear staining (DAPI) was opened. Using the Polygon selection tool, the cytoplasmic boundary of each cell to be analyzed was manually traced, carefully excluding the nucleus. After delineation, the Analyze > Tools > ROI Manager was used to open the ROI Manager, and the “Add” button was clicked to save the current selection to the list. This step was repeated for each cell to be analyzed.

### Manual identification and counting of stress granules

The view was switched to the 8-bit grayscale channel of the stress granule markers (TIA1 and G3BP1). In the ROI Manager, each saved cytoplasmic ROI was selected sequentially, and “Show All” followed by “Restore” was clicked to precisely locate the cell on the image. Subsequently, the Plugins > Analyze > Cell Counter plugin was activated. The counter type was set to “Point”. Only objects with a cross-sectional area corresponding to a diameter between 0.2 μm and 2.0 μm were counted as bona fide SGs. At least two researchers blinded to the experimental group assignment independently marked all clear, bright punctate fluorescent signals (i.e., stress granules) within the cytoplasmic area of the current cell by manual clicking. The software automatically recorded the coordinates of each marked granule. This process was repeated for all cells analyzed.

### Data collection and normalization

After marking granules in all cells, the counts were exported from the Cell Counter plugin. The primary data collected were the “stressed cells per field of view” and “number of SGs per cell”. For inter-group comparisons, a minimum of 50 field of views or 50 cells per group were analyzed. The final results are presented as the “mean number of stressed cells per field of view ± SEM” and “mean number of SGs per cell ± SEM”.

### RNA immunoprecipitation sequencing (RIP-seq)

RIP assays were conducted with stringent RNase protection using anti-TIA1 antibody (Abcam ab263945) in PA- or BSA-treated AML12 cells. Following lysis in polysome buffer (50 mM Tris-HCl pH 7.5, 150 mM NaCl, 1% NP-40, 0.1% SDS, 0.5% sodium deoxycholate, 1 mM DTT, RNaseOUT) supplemented with protease inhibitors, lysates were divided: 10% preserved as Input control, 80% immunoprecipitated with 5 μg anti-TIA1-protein A/G magnetic beads (1 h 4 °C rotation), and 10% incubated with rabbit IgG antibody (Cell Signaling Technology #2729) as negative control. Ribonucleoprotein complexes were washed with high-stringency buffer (1 M urea, 500 mM NaCl). Co-precipitated RNAs were extracted using TRIzol (Invitrogen 15596026), DNase-treated, and assessed for integrity (RIN ≥ 8.0). Stranded cDNA libraries were prepared with KAPA HyperPrep kit (Roche 07962363001) and sequenced on NovaSeq 6000 (Illumina) for 150 bp paired-end reads (E-GENE Tech). RIP enrichment was quantified using exomePeak v3.8 with stringent thresholds: |log₂FC | ≥1, FDR ≤ 0.05 compared to IgG control. For data analysis, the resulting clean reads were compared with the human reference genome (GRCh38) by STAR v2.7.3a [31]. The exomePeak (Version 3.8) [32] and ChIPseeker package [33] were used for peak calling and annotation. The raw data generated in this study were deposited in NCBI Sequence Read Archive (SRA) under BioProject accession number PRJNA1287280 (http://www.ncbi.nlm.nih.gov/bioproject/1287280/).

### Processing of RIP-seq data

The RIP-seq analysis was conducted by E-GENE Co. Ltd. Raw sequencing fastq files were assessed for quality, adapter content and duplication rates with FastQC v0.12.1, trimmed using trimmomatic v0.38 (parameters: SLIDINGWINDOW:30:15 AVGQUAL:15 LEADING:15 TRAILING:15 MINLEN:30 HEADCROP:5) and aligned with hisat2 v2.2.1 to either the Mus musculus genome using mm10 versions from UCSC database. RNA-binding protein (RBP)-binding region were identified by peak calling for each sample separately using exomepeak v2.16.0 (parameters: WINDOW_WIDTH = 200,SLIDING_STEP = 30,FRAGMENT_LENGTH = 200,FOLD_ENRICHMENT = 1.5). The different peak between groups were determined using exomepeak (parameters:WINDOW_WIDTH = 200,SLIDING_STEP = 30FRAGMENT_LENGTH = 200,FOLD_ENRICHMENT = 1.5,DIFF_PEAK_ABS_FOLD_CHANGE = 1.5,DIFF_PEAK_CUTOFF_PVALUE = 0.05,DIFF_PEAK_CONSISTENT_CUTOFF_PVALUE = 0.05). Peaks and different peaks were annotated to the position of genes using ChIPseeker v1.30.2. The motifs were analyzed using homer. The GO enrichment plots were plotted by R v4.1.1 and the peak visualization on genome was through igvtools v.2.19.6.

### Bioinformatics analysis

#### Analysis of bulk transcriptomic datasets

Normalized gene expression datasets (GSE160016, GSE130970, GSE193080) were downloaded from the Gene Expression Omnibus (GEO). Differential gene expression analysis was performed for each dataset using the limma package in R. Genes with an adjusted *p*-value < 0.05 and an absolute log₂ fold-change ≥ 1 were defined as differentially expressed genes (DEGs). Functional enrichment analysis of Gene Ontology (GO) terms and Kyoto Encyclopedia of Genes and Genomes (KEGG) pathways was conducted using KOBAS 3.0, with a false discovery rate (FDR) < 0.05 considered significant. All statistical analyses and visualization (e.g., volcano plots) were performed using R (version 4.2.1) on the OECloud platform (https://cloud.oebiotech.com).

### Analysis of single-cell RNA sequencing data

A human liver single-cell RNA sequencing atlas was obtained from the publicly accessible GepLiver database (www.gepliver.org). This resource integrates uniformly processed expression profiles from 409,775 single cells derived from 347 human liver samples, spanning multiple phenotypes.

### Correlation with clinical features

Clinical data and RNA-seq expression profiles for HCC patients were retrieved from The Cancer Genome Atlas (TCGA) Liver Hepatocellular Carcinoma (LIHC) project. The correlation between TIA1 expression and various clinical-pathological features was assessed using logistic regression. Results were visualized using the OECloud platform (https://cloud.oebiotech.com) and presented as bar plots and forest plots.

### Survival analysis

The prognostic value of TIA1 expression in HCC was evaluated using the Kaplan-Meier Plotter database (https://kmplot.com/analysis/). Overall survival (OS), recurrence-free survival (RFS), progression-free survival (PFS), and disease-specific survival (DSS) were analyzed. Hazard ratios and log-rank *p*-values were calculated using the Cox proportional hazards model, with a *p*-value < 0.05 considered statistically significant.

### Analysis of TIA1-RNA binding (eCLIP-seq)

To investigate the direct binding of TIA1 to *Srebf1* mRNA, enhanced crosslinking and immunoprecipitation sequencing (eCLIP-seq) data for HepG2 cells were obtained from the ENCODE project (Experiment: ENCSR623VEQ). A total of 1731 high-confidence TIA1 binding peaks were identified genome-wide. The genomic region of *Srebf1* (Chr17:17,810,399-17,836,986, GRCh38/hg38) was extended by 50 kb upstream and downstream of its transcription start and end sites to define a potential regulatory neighborhood. Overlap between TIA1 binding peaks and the extended *Srebf1* locus was assessed using BEDTools (v2.29.2) intersect, with any base-pair overlap (≥1 bp) considered a positive interaction.

### Statistical analysis

Data are presented as the mean ± standard error of the mean (SEM) as indicated. All the data were analysed using unpaired two-tailed Student’s t test, Wilcoxon ranksum test, or one-way analysis of variance (ANOVA) followed by post hoc t tests. Linear regression analysis adjusted for gender, age and BMI was used to compare two groups in human subjects. Pearson correlation analysis was used to determine the correlation between gene expression level and clinical parameters. RIP-seq has two biological replicates. For other experiments, the number of replicates is indicated in the figure legends. All statistical analyses were performed using GraphPad Prism version 10.0 (GraphPad Software, San Diego, California, USA). For all comparisons, *P*-values were considered significant at *p < 0.05, **p < 0.01, ***p < 0.001, ****p < 0.0001, with “ns” indicating not significant.

## Results

### Elevated hepatic TIA1 levels under metabolic stress exhibit negative correlation with MASH progression

**Fig. 1:**
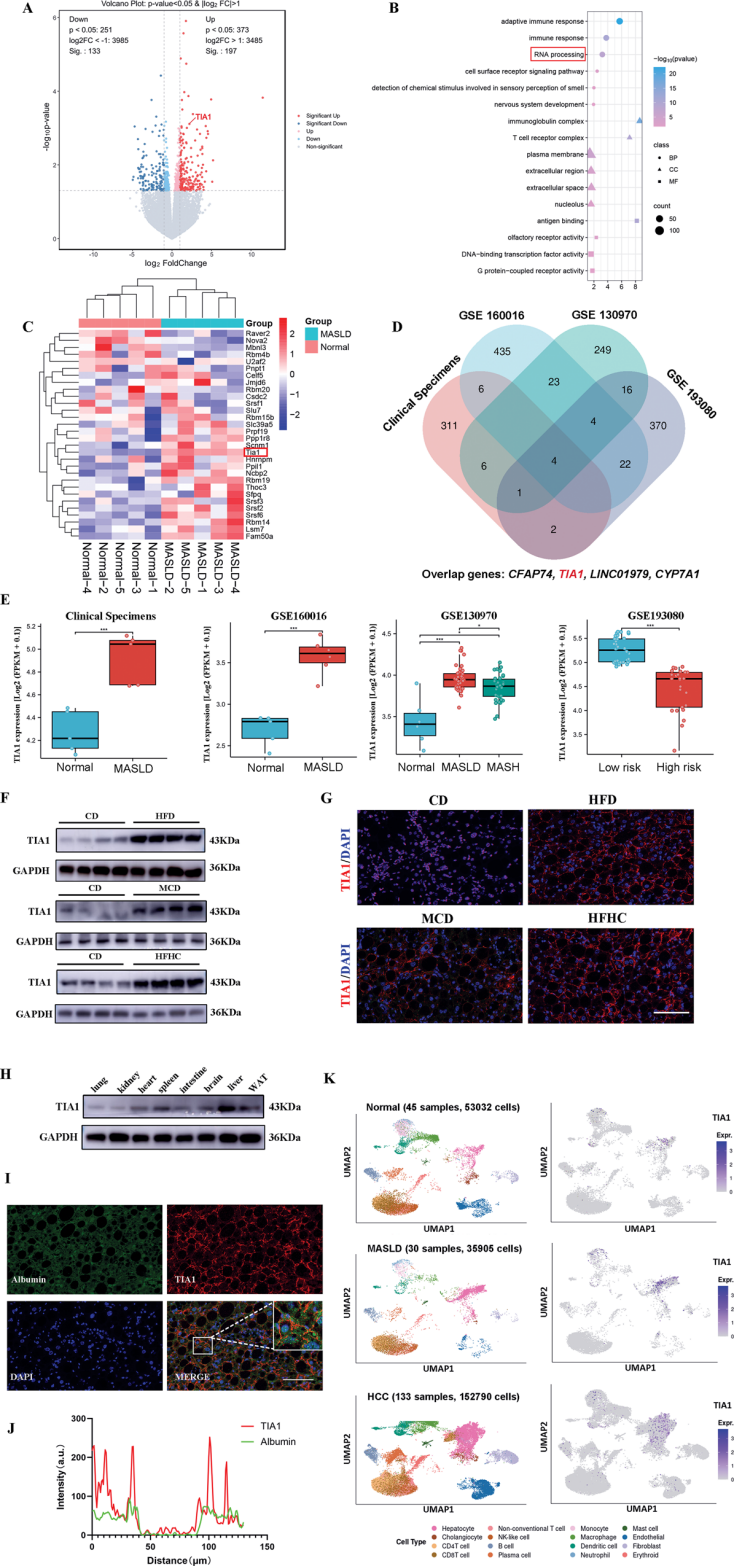
Elevated hepatic TIA1 levels under metabolic stress exhibit negative correlation with MASH progression. **A** Volcano plot depicting differentially expressed genes (DEGs) in liver tissues from MASLD patients (*n* = 5) compared to healthy controls (*n* = 5). Red and blue dots represent significantly up-regulated (*n* = 197) and down-regulated (*n* = 133) genes, respectively ( | log₂(fold change)| > 1 & adjusted *P* < 0.05). The gene TIA1 is highlighted. **B** Gene Ontology (GO) enrichment analysis of upregulated genes in livers of MASLD patients (*n* = 5 per group) when compared to those of healthy controls (*n* = 5 per group) from RNA-seq data. **C** Heat map showing the expression patterns of DEGs significantly enriched in the RNA processing GO biological process. Expression values are row-scaled Z-scores, with red indicating high expression and blue indicating low expression. Each row represents a gene, and each column represents a sample. The clear clustering demonstrates distinct expression profiles between control and MASLD groups. TIA1 exhibits consistently high expression in disease samples. **D** Venn diagram identifies a core set of four genes (*CFAP74, TIA1, LINC01979, CYP7A1*) that are consistently dysregulated across all compared datasets. **E** Integrated TIA1 expression analysis of in-house transcriptomic data with Gene Expression Omnibus (GEO) datasets (GSE160016, GSE130970, GSE193080) confirms consistent upregulation of TIA1 in MASLD and MASH cohorts. **F** Protein levels of TIA1 were determined by western bloting using the livers of mice fed with CD, HFD, MCD or HFHC diet. GAPDH served as a loading control (1 technical replicate of 3 biological replicates for each group). **G** Representative immunofluorescence staining images of TIA1 (red) of the liver sections of the mice fed with CD, HFD, MCD or HFHC diet, in which nuclei were stained with DAPI (blue). Scale bar: 100 μm. **H** TIA1 protein levels in the lung, kidney, heart, spleen, intestine, brain, liver and WAT of MASH mice. GAPDH served as a loading control (1 technical replicate of 3 biological replicates for each group). **I** Representative confocal images of MASLD mice’s liver section stained for albumin protein (green) and TIA1 (red). Nuclei were counterstained with DAPI (blue). Scale bar: 100 μm. **J** Profile intensity showing TIA1/Albumin fluorescence signals of the white box in (**I**). **K** Analysis of a public single-cell RNA-seq dataset from the GepLiver database (www.gepliver.org) confirms that TIA1 mRNA is predominantly expressed in hepatocytes in the context of healthy, MASLD and HCC staus. *P < 0.05, **P < 0.01, ***P < 0.001; ****P < 0.0001; ns indicates not significant.

We conducted RNA-seq analysis of liver samples from 5 MASLD/MASH patients and 5 healthy controls, identifying 197 significantly up-regulated and 133 down-regulated [|log₂(fold change)| > 1 & adjusted *P* < 0.05] differentially expressed genes (DEGs) (Fig. 1A). DAVID-based GO enrichment analysis revealed RNA processing machinery as one of the most significantly altered biological processes (Fig. 1B). The heat map depicted the expression patterns of RNA processing-related differentially expressed genes (Fig. 1C). Next, we integrated transcriptional profiles from clinical specimens with publicly accessible GEO datasets (GSE160016, GSE130970, GSE193080). Taking the intersection of these four lists yielded a core set of four consistently dysregulated genes, including *CFAP74*, *TIA1*, *LINC01979* and *CYP7A* (Fig. 1D). Among which, TIA1, an important RBP exhibited a clear high-expression profile in MASLD and MASH cohorts, particularly among individuals exhibiting lower hepatocellular carcinoma progression risk (Fig. 1A, C, E), suggesting an adverse clinical trajectories of TIA1 expression levels with the progress of MASLD and MASH. Interestingly, we observed a seemingly paradoxical expression pattern that TIA1 was upregulated in early MASLD/MASH but downregulated in advanced HCC, correlating with increased progression risk. This phenomenon suggests that TIA1 may play stage-specific, and potentially opposing, roles during liver disease progression.

Consistently, parallel protein-level investigations via western blotting (Fig. 1F) and immunofluorescence (Fig. 1G) demonstrated that TIA1 expression was upregulated in liver samples derived from three distinct diet-induced MASLD murine models—C57BL/6 mice administered with HFD, MCD, or HFHC diet regimens. Specifically, TIA1 is predominantly localized to the nucleus under CD condition, while primarily exhibits nucleo-cytoplasmic translocation under diet-induced stress according to the co-localization analysis (Figure S1D). These complementary analyses collectively identify TIA1 as a robustly dysregulated gene in the context of MASLD/MASH.

Based on KM-Plotter database (https://kmplot.com/analysis/), survival analysis incorporating OS, RFS, PFS and DSS metrics demonstrated significantly compromised survival outcomes among HCC patients with lower TIA1 expression (Log-rank test, *P* < 0.05; Figure S1A). Bioinformatics analysis of the TCGA database further demonstrated that low TIA1 expression was significantly associated with advanced pathological stage (III/IV), high AFP levels (>400 ng/mL), and severe adjacent hepatic inflammation (*p* < 0.05; Figure S1B, C).

In diet-induced MASLD mice, TIA1 exhibited higher expression levels in liver comparing to extrahepatic tissues including lung, kidney, heart, spleen, intestine, brain, white adipose based on western bolting (Fig. 1H). Dual immunofluorescence staining for TIA1 and albumin (Alb, hepatocyte-specific marker) in MASH mice liver sections confirmed predominant co-localization between TIA1 and Alb-positive hepatocytes, indicating hepatocytes constitute a primary source of TIA1 induction under metabolic perturbation (Fig. 1I, J). Consistently, single-cell RNA-sequencing dataset analysis based on GepLiver database (www.gepliver.org) according to the website’ s guidelines, verified that TIA1 is highly expressed in hepatocytes in the context of healthy, MASLD and HCC status (Fig. 1K).

In summary, these results demonstrate that hepatic TIA1 is significantly elevated in the MASLD/MASH microenvironment. Its expression pattern exhibits a strong negative correlation with disease progression and poor prognosis, suggesting a potential role as a predictive biomarker and a dynamic mediator in the pathogenesis of fatty liver disease and its progression to HCC.

### Hepatocyte-specific TIA1 deletion exacerbates HFD-induced hepatic steatosis and injury

To delineate the role of hepatocyte-expressed TIA1 in MASLD pathogenesis in vivo, *loxP*-flanked TIA1 mice were crossed with Albumin-Cre mice to generate hepatocyte-specific TIA1 knockout mice (TIA1-HKO). The TIA1flox/flox (TIA1-Flox) littermates were used as the control of TIA1-HKO mice. TIA1 expression was largely and specifically ablated in HKO liver tissues, but not in skeletal muscle or adipose tissue (Figure S2A). The hepatic *Tia1* mRNA expression was also validated by qRT-PCR analyses (Figure S2B). Next, TIA1-Flox and TIA1-HKO mice were fed a CD or HFD regimens for 12 weeks. While TIA1-Flox and TIA1-HKO mice on CD exhibited comparable hepatic phenotypes, TIA1 ablation significantly potentiated HFD-induced pathologies.

**Fig. 2:**
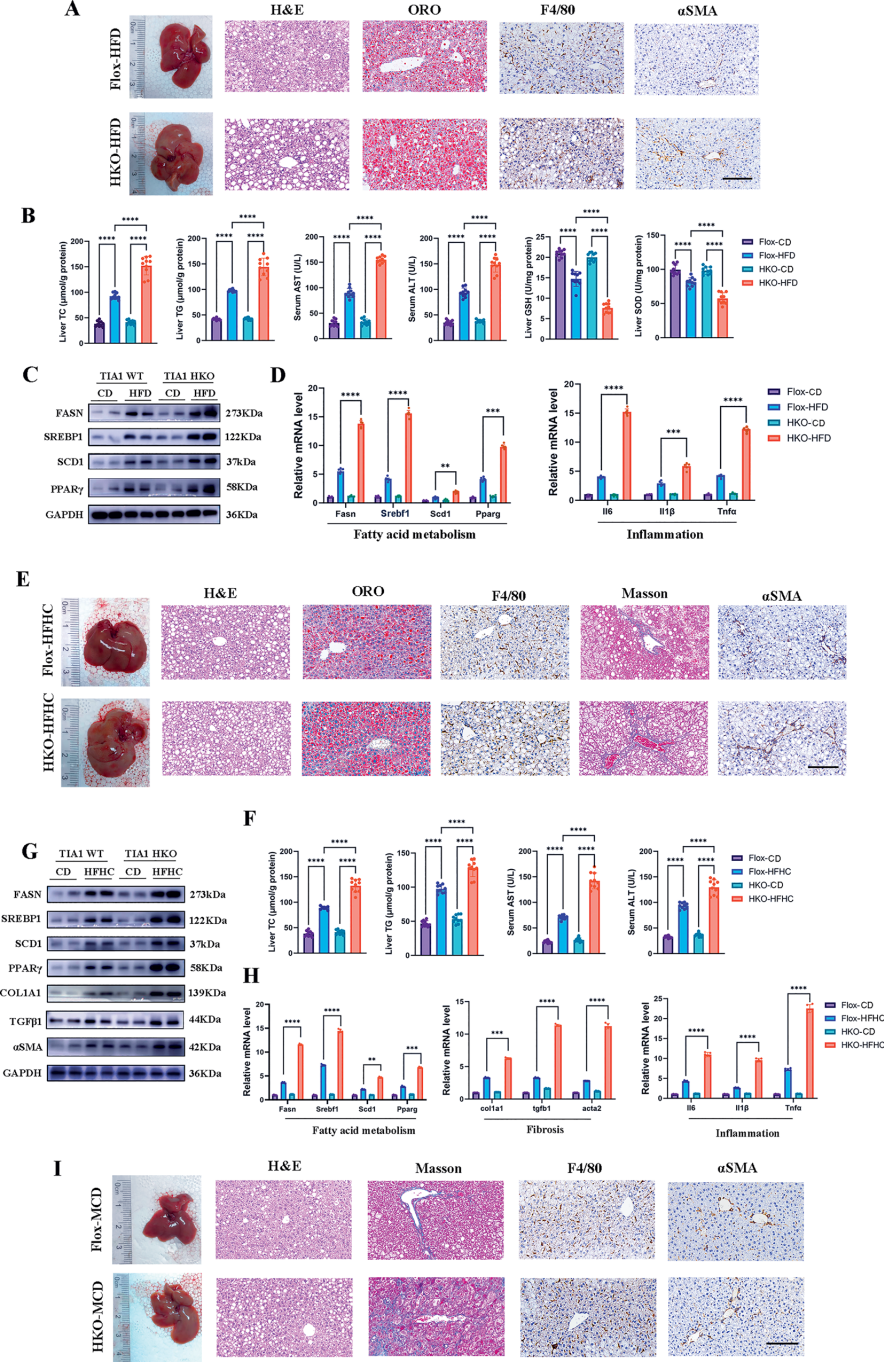
Hepatocyte-specific TIA1 deletion exacerbates die-induced hepatic steatosis, inflammation and fibrosis. **A**, **D** TIA1-Flox and TIA1-HKO mice were fed with CD or HFD for 12 weeks (*n* = 10 mice per group). **A** Representative gross liver morphology and photomicrographs of liver sections stained with H&E, Oil Red O (ORO), F4/80, and αSMA in the indicated groups. Scale bar: 100 μm. **B** Biochemical analyses of hepatic total cholesterol (TC) and triglyceride (TG) levels, serum alanine aminotransferase (ALT) and aspartate aminotransferase (AST) levels (liver injury markers), and hepatic glutathione (GSH) and superoxide dismutase (SOD) levels (oxidative stress markers). (*n* = 10 per group; mean ± SEM). **C** Western blot analysis of proteins involved in fatty acid metabolism (FASN, SREBP1, SCD1 and PPARγ). GAPDH served as a loading control. (*n* = 3 biologically independent mice per condition). **D** qRT-PCR analysis of genes involved in lipid metabolism (*Fasn, Srebf1, Scd1* and *Pparg*) and inflammation (*Il1β, Il6* and *Tnfα*), normalized to *Gapdh*. (*n* = 5 biologically independent mice per condition; mean ± SEM). **E**–**H** TIA1-Flox and TIA1-HKO mice were fed with CD or HFHC diet for 20 weeks (*n* = 10 mice per group). **E** Representative gross liver morphology and photomicrographs of liver sections stained with H&E, ORO, F4/80, Masson and αSMA staining of the liver sections from the indicated groups. Scale bar: 100 μm. **F** Biochemical analyses of hepatic TC and TG levels, serum ALT and AST levels in the indicated groups of mice. (*n* = 10 per group; mean ± SEM) (**G**) Western blot analysis of proteins involved in fatty acid metabolism (FASN, SREBP1, SCD1 and PPARγ) and fibrogenesis (COL1A1, TGF-β1, αSMA). GAPDH served as a loading control. (*n* = 3 biologically independent mice per condition; mean ± SEM). **H** qRT-PCR analysis of genes related to lipid metabolism (*Fasn, Srebf1, Scd1* and *Pparg*), fibrogenesis (*Col1a1, Tgfβ1* and *Acta2*), and inflammation (*Il1β, Il6* and *Tnfα*), normalized to *Gapdh*. (*n* = 5 biologically independent mice per condition; mean ± SEM). **I** TIA1-Flox and TIA1-HKO mice fed CD or MCD for 8 weeks (*n* = 10 mice per group). Representative gross liver morphology and photomicrographs of liver sections stained with H&E, Masson, F4/80, and αSMA staining of the liver sections from the indicated groups. Scale bar: 100 μm. *P < 0.05, **P < 0.01, ***P < 0.001; ****P < 0.0001; ns indicates not significant.

Specifically, while average daily food intake were comparable among groups (Figure S2D), TIA1-HKO mice displayed significant elevation in liver-to-body mass ratios (23.5% increase, *P* < 0.001) post-HFD (Figure S2E). Macroscopically, TIA1-HKO livers exhibited notably pallid parenchyma with increased organ dimensions (Fig. 2A, S2F). Histopathological assessment using H&E staining confirmed that HFD triggered steatosis, ballooning degeneration, necroinflammation, and lobular inflammation in TIA1-Flox controls, which were markedly exacerbated in TIA1-HKO specimens (Fig. 2A; S2F). NAS quantification revealed significantly elevated composite and component scores (steatosis, ballooning, inflammation) in TIA1-HKO cohorts (*P* < 0.05) (Figure S2G). Subsequent Oil Red O staining further supported the amplified lipid deposition (76% increase, P < 0.001) in TIA1-HKO livers (Fig. 2A; S2H). Immunohistochemistry demonstrated expanded F4/80-positive macrophage infiltration (34.6% increase, *P* < 0.001) and αSMA-positive myofibroblast activation domains (24.3% increase, *P* < 0.01) (Fig. 2A; S2H). In the context of our study, which focuses on MASLD progression, an increase in F4/80-positive macrophages and αSMA-positive myofibroblast signifies the recruitment of resident and/or monocyte-derived macrophages and the activation of quiescent hepatic stellate cells (HSCs) into contractile, extracellular matrix (ECM)-producing myofibroblasts, respectively. These observations underlying inflammatory milieu and pro-fibrotic response which are critical steps towards the development of liver fibrosis and the advancement of our disease model.

Biochemical profiling substantiated that TIA1-HKO mice exhibited substantial increment in hepatic TC (64.8% increase, P < 0.001) and TG (47.5% increase, *P* < 0.001) content versus TIA1-Flox controls, paralleled by serum TC (53.8% increase, *P* < 0.001) and TG (48.4% increase, *P* < 0.001) elevations (Fig. 2B; S2I). Furthermore, TIA1-HKO livers manifested heightened hepatocellular injury markers (serum AST/ALT: 73.0%/58.2% increase, *P* < 0.0001) coupled with depleted antioxidant reserves (serum GSH/SOD: 29.3%/84.4% decrease, *P* < 0.001) (Fig. 2B). Western blot (Fig. 2C; S2J) and qRT-PCR analyses (Fig. 2D) revealed TIA1 deletion-driven upregulation of lipogenic regulators including fatty acid synthase (*Fasn*, encodes FASN), sterol regulatory element-binding transcription factor 1 (*Srebf1*, encodes SREBP1), stearoyl-coenzyme a desaturase-1 (*Scd1*, encodes SCD1), peroxisome proliferator–activated receptor gamma (*Pparg*, encodes PPARγ*)* and pro-inflammatory mediators including Interleukin-1β (*Il1β*, encodes IL-1β), Interleukin-6 (*Il6*, encodes IL-6), Tumor necrosis factor-α (*Tnfα*, encodes TNF-α), at both protein and mRNA levels.

To sum up, TIA1-HKO exacerbates HFD-induced hepatic lipid accumulation and inflammation, leading to significant liver damage and metabolic disturbances.

### TIA1 deficiency aggravates HFHC diet- and MCD-induced hepatic steatosis, inflammation and fibrosis

Building upon the influence of TIA1 on HFD-induced hepatic steatosis, we further probed its contribution to MASH pathogenesis using a more aggressive HFHC dietary paradigm, which more faithfully recapitulates human disease histopathology and metabolic perturbations.

Consistent with prior observations, while average daily food intake were comparable among groups (Figure S3B), TIA1-HKO mice subjected to 20-week HFHC feeding manifested substantially elevated liver-to-body weight indices (19.1% increase, *P* < 0.01) relative to TIA1-Flox controls (Figure S3C). TIA1-HKO mice developed conspicuous hepatomegaly with parenchymal discoloration (Fig. 2E; S3D). Histopathology analysis revealed amplified macrovesicular steatosis, enhanced lobular inflammation, and exacerbated bridging fibrosis in TIA1-HKO cohorts via H&E staining and NAS quantification (Fig. 2E; S3D, E). Subsequent Oil Red O and Masson’s trichrome staining corroborated amplified lipid deposition (61.1% increase, *P* < 0.001) and pericellular/perisinusoidal collagen accumulation (62.4% increase, *P* < 0.001) in TIA1-HKO livers (Fig. 2E; S3D, F). Immunohistochemical analyses demonstrated markedly elevated αSMA-positive myofibroblast activation (47.6% increase, *P* < 0.001) and F4/80-positive macrophage infiltration (65.6% increase, *P* < 0.001) in TIA1-HKO mice (Fig. 2E; S3D, F). Biochemical profiling demonstrated augmented lipid accumulation (hepatic TC/TG: 49.0%/30.8%, *P* < 0.0001; serum TC/TG: 37.9%/30.8% increase, *P* < 0.001) alongside elevated hepatocellular injury markers (serum AST/ALT: 82.1%/38.9% increase, *P* < 0.001) and compromised redox homeostasis (serum GSH/SOD: 40.8%/40.2% increase, *P* < 0.001) (Fig. 2F; S3G). Molecular investigation further confirmed coordinated translational (Fig. 2G; S3H) and transcriptional (Fig. 2H) upregulation of lipogenic (FASN, SREBP1, SCD1, and PPARγ), fibrogenic (Collagen Type I Alpha 1 Chain, COL1A1; transforming growth factor-β1, TGF-β1; αSMA) and pro-inflammatory (IL-1β, IL-6, and TNF-α) mediators.

To corroborate these findings, we also confirmed our findings in a 8-week MCD diet model. Histopathology staining, biochemical profiling and molecular investigation together underscored that TIA1-HKO mice similarly exhibited increased liver injury, lipid accumulation, inflammation, and fibrosis (Fig. 2I; S4A–J).

Taken together, these results indicated that absence of hepatic TIA1 exaggerated the HFHC- and MCD-induced hepatic steatosis, inflammation and fibrosis.

### Reconstitution of TIA1 attenuates HFD-induced MASLD and hepatocellular injury

**Fig. 3:**
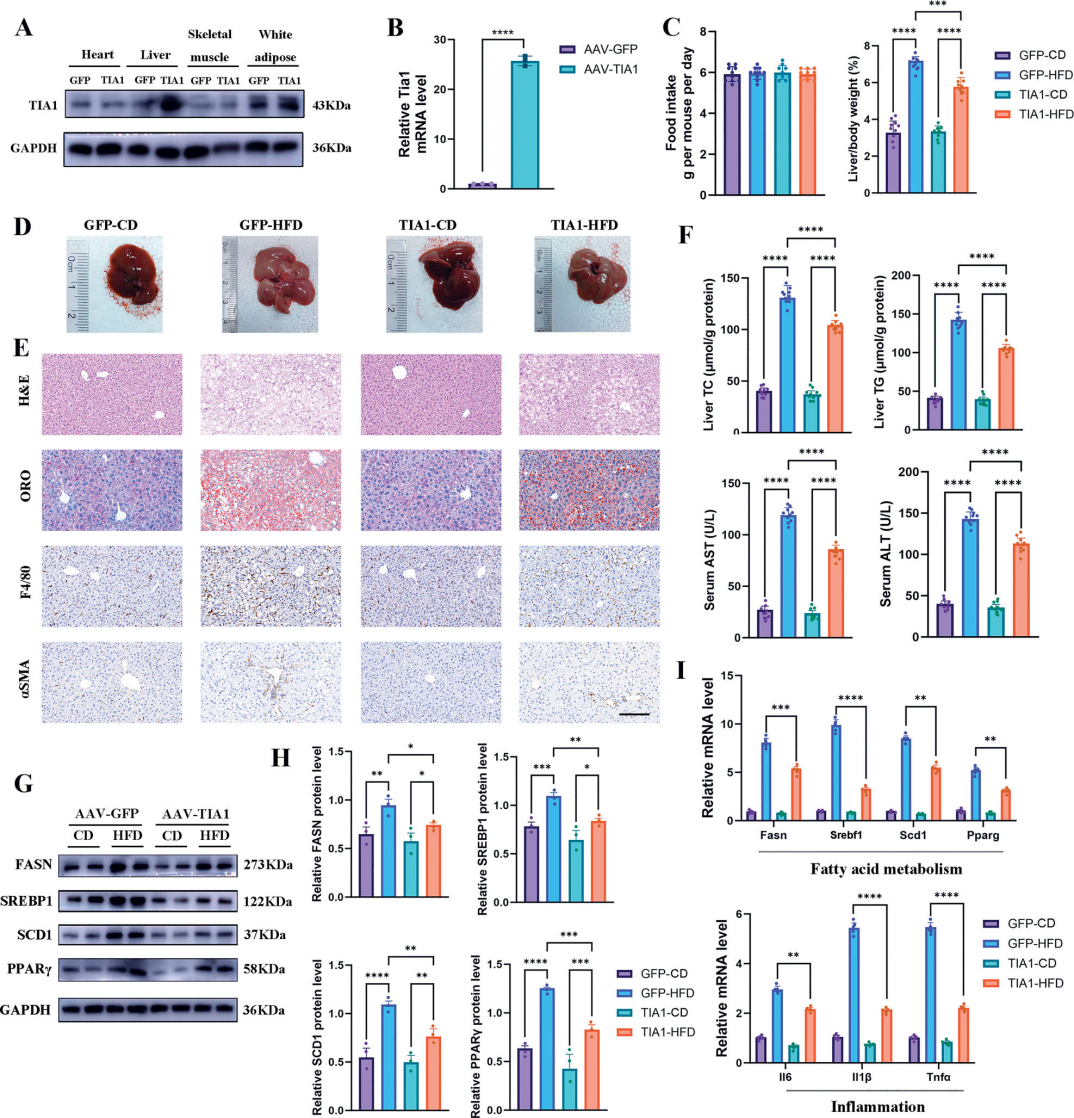
Reconstitution of TIA1 attenuates HFD-induced MASLD and hepatocellular injury. **A** Western blot analysis of TIA1 protein levels in heart, liver, skeletal muscle, and white adipose tissue (WAT) from MASH and control mice treated with AAV-TIA1 (overexpression) or AAV-GFP (control). GAPDH served as a loading control (1 technical replicate of 3 biological replicates for each group). **B** Quantitative RT-PCR analysis of *Tia1* mRNA levels in liver tissues from AAV-TIA1 and AAV-GFP treated mice, normalized to *Gapdh*. (*n* = 3 biologically independent mice per condition). **C** Average daily food intake measured over 3 consecutive days (Left panel) and liver to body weight ratios (%) (Right panel) of TIA1-FLox and TIA1-HKO mice fed CD or HFD diet for 12 weeks (*n* = 10 mice per group; mean ± SEM). **D** Representative gross morphological photographs of livers from the indicated groups. **E** Representative photomicrographs of liver sections stained with H&E, ORO, F4/80 and αSMA staining of the liver sections from indicated groups. Scale bar: 100 μm. **F** Biochemical analyses of hepatic TC and TG levels, and serum AST and ALT activities (*n* = 10 per group; mean ± SEM). **G**, **H** Western blot analysis of key proteins involved in fatty acid metabolism (FASN, SREBP1, SCD1, and PPARγ) in liver lysates from the indicated groups. GAPDH served as a loading control. **H** Densitometric quantification of protein levels from (**G**) (*n* = 3 independent biological replicates; mean ± SEM). **I** qRT-PCR analysis of genes related to lipid metabolism (*Fasn, Srebf1, Scd1* and *Pparg*), fibrogenesis (*Col1a1, Tgfβ1* and *Acta2*), and inflammation (*Il1β, Il6* and *Tnfα*), normalized to *Gapdh*. (*n* = 5 biologically independent mice per condition; mean ± SEM). *P < 0.05, **P < 0.01, ***P < 0.001; ****P < 0.0001; ns indicates not significant.

Given the deteriorated steatohepatitis progression in TIA1-deficient mice, we subsequently investigated whether TIA reconstitution could ameliorate HFD-induced hepatic pathology. Hepatotropic recombinant adeno-associated virus serotype 8 (rAAV8) vectors expressing human TIA1 (rAAV-TIA1) or control GFP (1 × 10⁹ plaque-forming units) were administered via tail vein injection to male C57BL/6 J mice. Following a 7-day convalescence period, cohorts were systematically allocated into four experimental groups: CD with rAAV-GFP (GFP-CD), HFD with rAAV-GFP (GFP-HFD), CD with rAAV-TIA1 (TIA1-CD), and HFD with rAAV-TIA1 (TIA1-HFD) for 12 weeks, with viral transduction in liver but not muscle confirmed through western blot (Fig. 3A) and qRT-PCR (Fig. 3B) analyses.

The TIA1-HFD cohort exhibited significantly attenuated liver-to-body weight ratios (23.7% decrease, P < 0.001) compared to GFP-HFD controls while average daily food intake were comparable among groups (Fig. 3C). Macroscopically, TIA1 replenishment rescued the HFD induced hepatomegaly and pale yellowish discoloration (Fig. 3D). Histopathological examination (Fig. 3E; S5B-C) revealed substantial mitigation of hepatocellular ballooning and lobular inflammation via H&E staining alongside reduced macrovesicular steatosis (Oil Red O positive area: 27.3% decrease, *P* < 0.001), which was biochemically corroborated by decreased hepatic TC/TG (20.3%/25.8% decrease, *P* < 0.001) and circulating TC/TG (21.0%/16.4% decrease, *P* < 0.001) (Fig. 3F; S5D). Immunohistochemical analyses (Fig. 3E; S5C) demonstrated markedly attenuated αSMA-positive myofibroblast activation (17.5% decrease, *P* < 0.05), and suppressed F4/80-positive macrophage infiltration (34.9% decrease, *P* < 0.001). Biochemistry analysis demonstrated the restoration of serum AST (27.7% decrease, *P* < 0.001) and ALT (20.7% decrease, *P* < 0.001) along with GSH (60.9% increase, *P* < 0.001) and SOD (36.4% increase, *P* < 0.001) (Fig. 3F; S5D). Molecular profiling further revealed significant downregulation of lipogenic transcriptional regulators (FASN, SREBP1, SCD1, and PPARγ) and pro-inflammatory mediators (IL-1β, IL-6, TNF-α) at both protein (Fig. 3G, H) and mRNA (Fig. 3I) levels.

The above mentioned data collectively establish that rAAV8-mediated TIA1 reconstitution substantially impedes HFD-driven steatotic, dyslipidemic, and inflammatory hepatic pathogenesis.

### TIA1-positive SGs are activated in PA-treated hepatocytes and participate in the regulation of lipid homeostasis

To elucidate the functional role of TIA1 in AML12 hepatocytes under metabolic stress conditions, an in vitro model of MASLD was established by subjecting AML12 cells to lipotoxic stress.

**Fig. 4:**
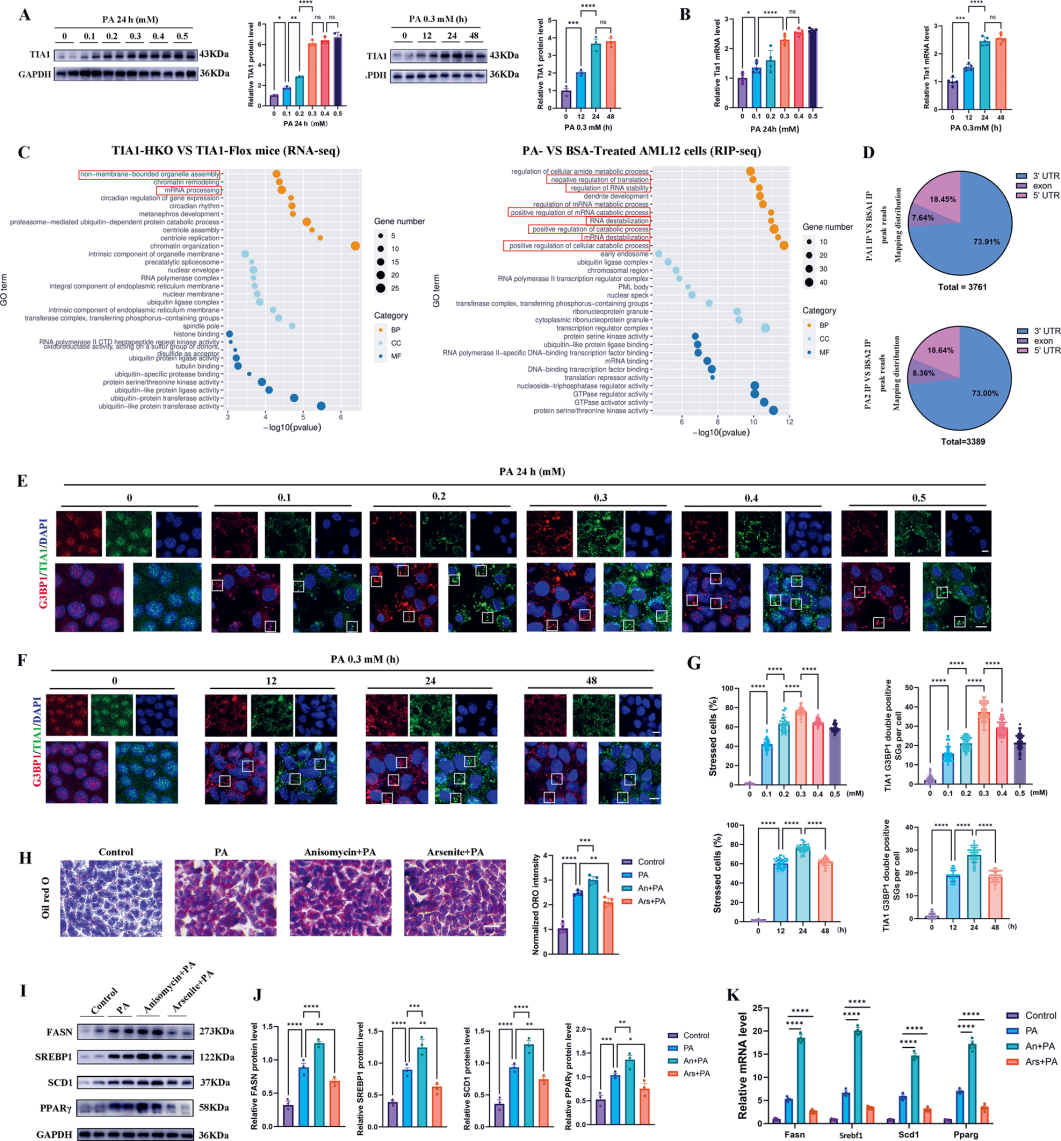
TIA1-positive SGs are activated in PA-treated hepatocytes and participate in the regulation of lipid homeostasis. **A**, **B** AML12 hepatocytes were treated with palmitic acid (PA) at indicated doses and time points. **A** Western blot analysis of TIA1 protein levels. GAPDH served as a loading control. (n = 3 independent biological replicates). **B** qRT-PCR analysis of *Tia1* mRNA expression normalized to *Gapdh*. (*n* = 5 independent biological replicates). **C** Left panel: GO biological process analysis of differential expressed genes in livers of TIA1-HKO mice (*n* = 3 per group) when compared to those of TIA1-Flox controls (*n* = 3 per group) from RNA-seq data. Right panel: GO biological process analysis of genes harboring differential TIA1-binding peaks in PA-treated versus BSA-treated control AML12 cells, identified by RIP-seq. **D** Genomic distribution of TIA1-binding peaks of AML12 cells upon PA treatment from the two biological replicates of RIP-seq. **E**, **F** AML12 hepatocytes were treated with PA at indicated doses and time points. Representative immunofluorescence images of AML12 cells treated with PA, co-stained for TIA1 (green) and the core SGs marker G3BP1 (red). Nuclei were counterstained with DAPI (blue). White boxes in indicate regions shown representative SGs exhibiting co-localization. Scale bar: 10 μm. **G** Quantification of SGs formation from experiments in (**E**, **F**). Left panel: Percentage of cells containing SGs (*n* = 50 cells per group). Right panel: Average number of TIA1 G3BP1 double positive SGs per cell (*n* = 50 views per group). **H**–**K** AML12 cells were pre-treated with the Anisomycin (An, 25 ng/mL for 30 min) or sodium arsenite (Ars, 0.5 mM for 1 h) prior to PA challenge (0.3 mM for 24 h). **H** Representative ORO staining visualizing neutral lipid accumulation and normalized quantification (*n* = 5 independent biological replicates; mean ± SEM). Scale bar: 50 μm. **I** Western blot analysis of lipogenic proteins (FASN, SREBP1, SCD1, PPARγ). GAPDH served as a loading control. **J** Densitometric quantification of protein levels from (**I**) (*n* = 3 independent biological replicates; mean ± SEM). **K** qRT-PCR analysis of lipid metabolism-related gene expression (*Fasn*, *Srebf1*, *Scd1* and *Pparg*), normalized to *Gapdh* (*n* = 5 independent biological replicates; mean ± SEM). *P < 0.05, **P < 0.01, ***P < 0.001; ****P < 0.0001; ns indicates not significant.

Initial screening using various fatty acids revealed that PA (400 μM) significantly upregulated both TIA1 protein and mRNA levels (*p* < 0.001), while a combination of PA and oleic acid (PA + OA, 200 μM each), or OA alone (400 μM) showed less effects (Figure S6A). We next characterized the dose- and time-dependency of PA-induced TIA1 expression. Treatment with increasing concentrations of BSA-conjugated PA (100–500 μM) resulted in a dose-dependent upregulation of TIA1 assessed via western blot and qRT-PCR analyses (Fig. 4A, B). Specifically, our results showed that PA dose-dependently upregulate TIA1 at both protein and mRNA levels from 100 to 300 μM, while no significant change is shown from 300 to 500 μM PA. Therefore, 300 μM PA was determined as the stimulating concentration in the next experiments. Following this determination, AML12 cells were treated with 300 μM PA for 12, 24, and 48-h intervals, revealing time-dependent TIA1 upregulation at both protein and mRNA levels, with peak expression occurring at 24 h of PA exposure according to western blot and qRT-PCR analyses (Fig. 4A, B).

We further investigated downstream targets potentially mediating TIA1’s protective effects against MASH pathogenesis. RNA-seq were conducted to provide an unbiased over-view of the pathways changed by TIA1-HKO, which elicited several significantly disregulated pathways in TIA1-HKO mice, notably “non-membrane-bounded organelle assembly” and “mRNA processing” (Fig. 4C). Given TIA1’s established role as an RBP regulating RNA metabolism, RIP-seq assays were performed on PA- and BSA-treated AML12 cells to identify mRNAs bound by TIA1 under metabolic stress, thereby elucidating molecular mechanisms through which TIA1 post-transcriptionally regulates MASLD/MASH progression. M-versus-A plots (MA plots) and volcano plots confirmed the quality of data and implied significant variance in TIA1-bound transcripts between PA- and BSA-treatments, totally identified 1706 and 1845 distinct binding peaks in the two replicates (Figure S9A, B). The enriched RNA motifs identified from the RIP-seq peaks specifically bound to TIA1 are displayed as sequence logos in Figure S9C. GO enrichment analysis upon distinct TIA1-binding peaks of PA-treated versus BSA-treated AML12 cells highlighting TIA1’s predominant involvement in translational repression, RNA stability modulation, mRNA catabolic process enhancement, and cytoplasmic ribonucleoprotein granule organization (Fig. 4C). Comparative mapping of immunoprecipitated samples demonstrated similar genomic distributions, with predominant enrichment at 3’ UTRs (Fig. 4D).

As a central regulator governing the assembly of pro-survival-SGs in response to homeostatic fluctuations, TIA1’s impact on SGs formation was examined in the PA-induced hepatocyte model to determine whether exacerbated liver injury in TIA1-HKO mice stems from impaired SGs biogenesis. Consistent with its well-documented role in inducing ER stress and oxidative stress, which are necessary for the nucleation of SGs, PA treatment robustly triggered SGs assembly in AML12 cells, whereas the addition of OA (PA + OA) or OA treatment alone failed to elicit a similar response (Figure S6B, C). Confocal microscopy revealed pronounced SGs assembly, demonstrating both dose-dependent and time-dependent induction patterns in AML12 hepatocytes following PA exposure. Specifically, 300 μM PA treating for 24 h induced the most aboundant stressed cells and SGs per cell according to quantitative analysis (Fig. 4E–G). In addition, PA treatment induces a marked redistribution of TIA1 from a predominantly nuclear localization to the cytoplasm, where it coalesces into puncta. This translocation coincided temporally with the formation of G3BP1-positive stress granules, suggesting the relocalized TIA1 is incorporated into these cytoplasmic condensates.

Moreover, pharmacological modulation confirmed that the SGs inhibitor anisomycin suppressed PA-induced SGs formation, whereas the SGs agonist sodium arsenite potentiated it (Figure S6D, E). Consistent with SGs’ protective function, Oil Red O and BODIPY staining demonstrated that SGs counteract PA-induced lipid accumulation in AML12 hepatocytes (Fig. 4H; S6F). Specifically, anisomycin treatment further elevated PA-induced intracellular lipid deposition, while arsenite treatment significantly attenuated it. Corroborating these observations, western blot and qRT-PCR analyses revealed elevated expression of fatty acid uptake and lipogenesis markers at both translational and transtriptional levels in anisomycin-treated cells under PA stress, while Arsenite exhibited the opposite effect (Fig. 4I–K).

Collectively, these findings demonstrate that TIA1-mediated SGs formation represents a protective mechanism against lipotoxic stress in hepatocytes, and that impairment of this pathway exacerbates lipid accumulation and promotes MASH progression.

### Hepatocyte TIA1 is essential for SGs formation during PA-induced hepatic injury

To define the functional requirement of TIA1 for SGs assembly and its metabolic consequences, we established loss-of-function (TIA1 knockdown) and gain-of-function (TIA1 overexpression) models in AML12 hepatocytes. Transfection efficiency was confirmed by western blot and qRT-PCR analyses (Figure S7A, B).

**Fig. 5:**
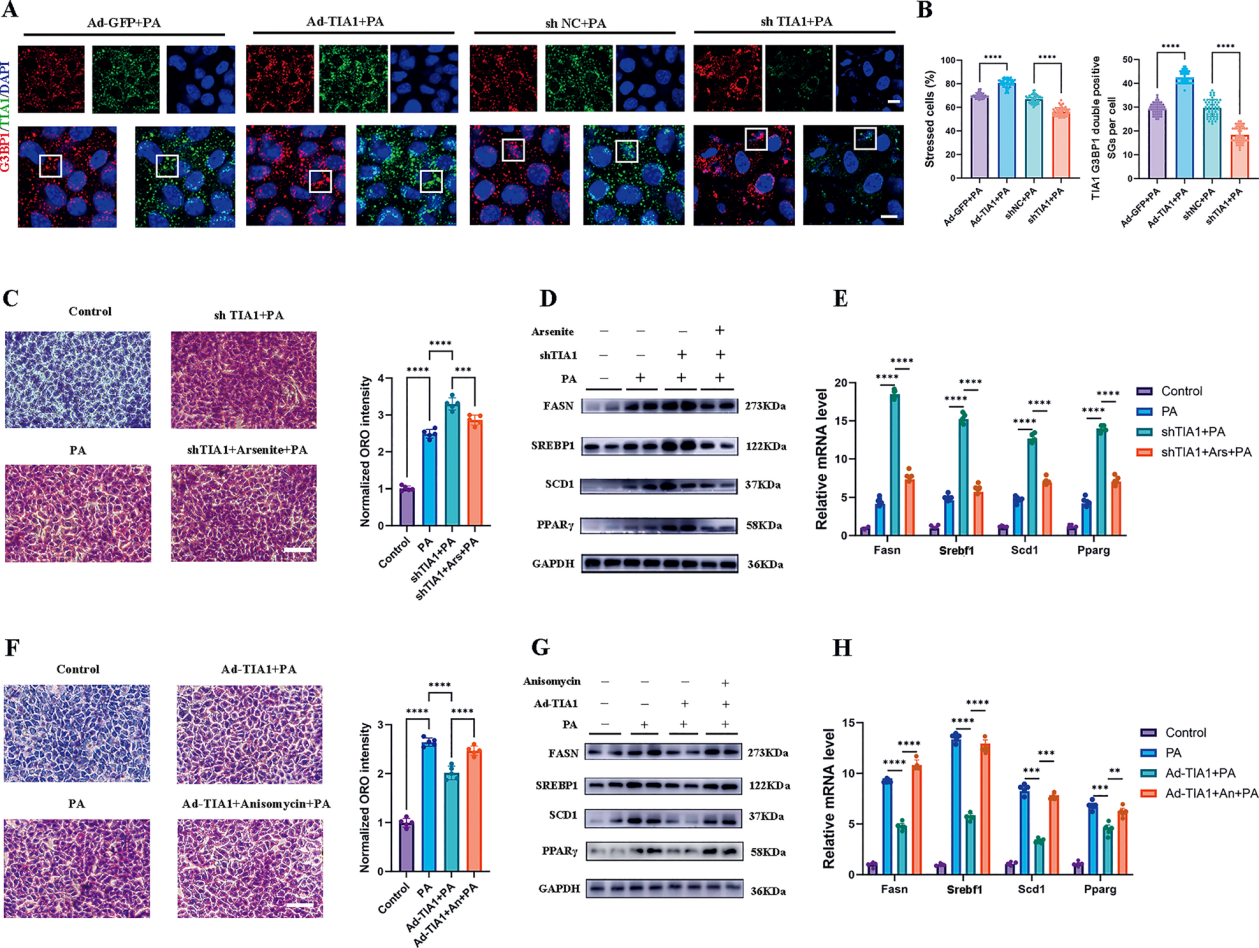
Hepatocyte TIA1 is essential for SGs formation during PA-induced hepatic injury. **A** Representative immunofluorescence images of AML12 cells following genetic manipulation. Cells were infected with adenovirus overexpressing TIA1 (Ad-TIA1) or GFP control (Ad-GFP), or transfected with shRNA targeting TIA1 (shTIA1) or negative control shRNA (shNC), then treated with PA (0.3 mM for 24 hours). Cells were co-stained for TIA1 (green) and the core SGs marker G3BP1 (red). Nuclei were counterstained with DAPI (blue). White boxes in indicate regions shown representative SGs exhibiting co-localization. Scale bar: 10 μm. **B** Quantification of SGs formation from experiments in (**A**). Left panel: Percentage of cells containing SGs (*n* = 50 cells per group). Right panel: Average number of TIA1 G3BP1 double positive SGs per cell (*n* = 50 views per group). **C**–**E** AML12 cells were infected with shTIA1 or shNC, with or without pre-treatment with the SGs inducer sodium arsenite (Ars, 0.5 mM, for 1 hour), followed by PA challenge (0.3 mM for 24 h). **C** Representative ORO staining visualizing intracellular lipid droplets in indicated groups and the normalized quantification (*n* = 5 independent biological replicates; mean ± SEM). Scale bar: 50 μm. **D** Western blot analysis of lipogenic proteins (FASN, SREBP1, SCD1, PPARγ). GAPDH served as a loading control. **E** qRT-PCR analysis of lipid metabolism-related gene expression (*Fasn*, *Srebf1*, *Scd1* and *Pparg*), normalized to *Gapdh* (*n* = 5 independent biological replicates; mean ± SEM). **F**–**H** AML12 cells were infected with Ad-TIA1 or Ad-GFP, with or without pre-treatment with the SGs inducer anisomycin (An, 25 ng/mL, for 30 min), followed by PA challenge (0.3 mM for 24 h). **F** Representative ORO staining and normalized quantification (*n* = 5 independent biological replicates; mean ± SEM). Scale bar: 50 μm. **G** Western blot analysis of lipogenic proteins (FASN, SREBP1, SCD1, PPARγ). GAPDH served as a loading control. **H** qRT-PCR analysis of lipid metabolism-related gene expression (*Fasn*, *Srebf1*, *Scd1* and *Pparg*), normalized to *Gapdh* (*n* = 5 independent biological replicates; mean ± SEM). *P < 0.05, **P < 0.01, ***P < 0.001; ****P < 0.0001; ns indicates not significant.

Immunofluorescence analysis revealed that SGs formation was markedly impaired in TIA1-knockdown cells but enhanced in TIA1-overexpressing cells following PA challenge (Fig. 5A, B). Concordantly, BODIPY staining showed increased lipid accumulation upon TIA1 knockdown and decreased accumulation upon its overexpression (Figure S7C).

Functional analysis confirmed that TIA1 deficiency exacerbated PA-induced lipotoxic stress. TIA1-knockdown cells exhibited enlarged Oil Red O-positive lipid domains and elevated expression of lipogenic genes at both protein and mRNA levels (Fig. 5C–E; S8A). These cells also showed increased oxidative stress, as indicated by elevated DCFH-DA fluorescence, pro-inflammatory cytokines (IL-1β, IL-6, TNF-α), malondialdehyde (MDA), and reduced superoxide dismutase (SOD) activity (Figure S8C, D). Notably, treatment with an SGs agonist rescued these deleterious phenotypes (Fig. 5C–E; S8C, D).

Conversely, TIA1 overexpression attenuated intracellular lipid accumulation and reduced the expression of lipogenic regulators under PA stress (Fig. 5F–H; S8B). It also alleviated oxidative stress markers fluctuation (Figure S8E, F). These protective effects were reversed by pharmacological inhibition of SGs formation (Fig. 5F–H; S8E, F).

Interestingly, we noticed that TIA1 ablation led to a compensatory upregulation of two other core components of SGs, G3BP1 and T-cell inhibitor of apoptosis-related protein (TIAR), at both protein and mRNA levels (Figure S7D). To assess whether these proteins mediate TIA1’s metabolic effects, we manipulated G3BP1 and TIAR individually (Figure. S7E–H). However, neither knockdown nor overexpression of G3BP1 or TIAR alone significantly altered intracellular lipid droplet content or the expression of key lipogenic genes (FASN, SREBP1, SCD1, PPARγ) under PA stress (Figure S7I–L). This indicates that G3BP1 and TIAR, while responsive to TIA1 status, are not sufficient to recapitulate TIA1’s regulatory role in hepatocyte lipid metabolism, suggesting TIA1 acts through a specific complex or distinct downstream targets.

In summary, TIA1 is essential for SGs assembly in hepatocytes under lipotoxic stress. Its downregulation exacerbates liver injury primarily by impairing this pro-survival adaptive mechanism, thereby compromising the cellular adaptive capacity to counteract metabolic and oxidative stress.

### TIA1 impedes MASH progression by binding to the 3’ UTR of *Srebf1* mRNA and suppressing its translational activation

**Fig. 6:**
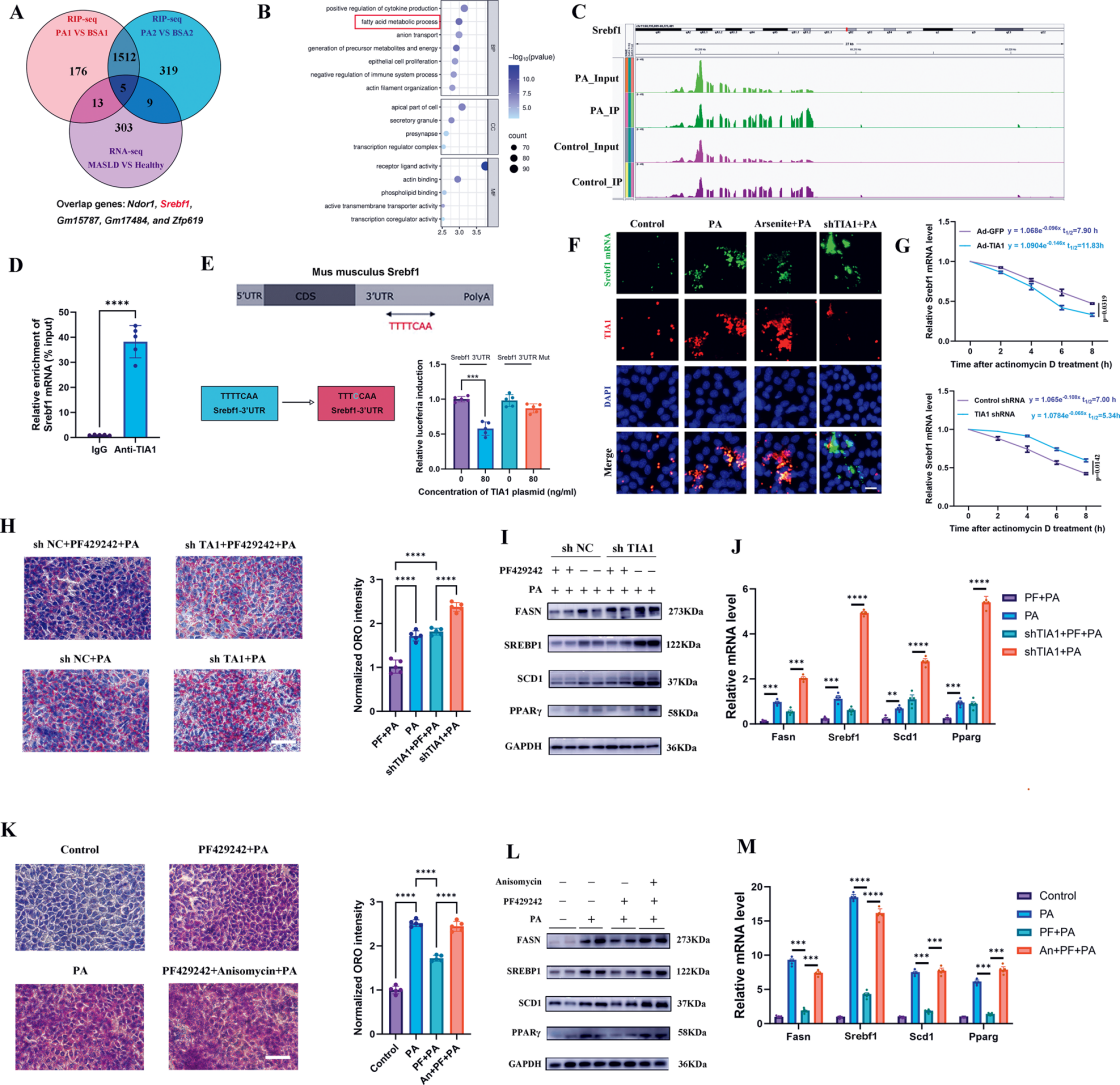
TIA1 impedes MASH progression by binding to the 3’ UTR of *Srebf1* mRNA and suppressing its translational activation. **A** Venn diagram illustrating the overlap between distinct TIA1 binding genes of PA- and BSA-treated AML12 cells (from the two biological replicates of RIP-seq) and DEGs of MASLD and healthy livers (from RNA-seq of clinical specimens). Overlapping genes including *Ndor1*, *Srebf1*, *Gm15787*, *Gm17484*, and *Zfp619*. **B** Gene Ontology (GO) analysis of biological processes enriched among DEGs from RIP-seq of PA- and BSA-treated AML12 cells. **C** Integrative Genomics Viewer (IGV) snapshot showing representative TIA1-binding peaks on *Srebf1* mRNA, as identified by RIP-seq in PA-treated versus BSA-treated AML12 cells (**D**) RIP-qPCR validation of the specific enrichment of *Srebf1* mRNA in TIA1 immunoprecipitates compared to control IgG precipitates from AML12 cell lysates (*n* = 5 independent biological replicates; mean ± SEM). **E** Dual-luciferase reporter assay in AML12 hepatocytes. Luciferase activity of reporters containing the wild-type (WT) or mutant (MUT) *Srebf1* 3’ UTR was measured after co-transfection with a TIA1 overexpression plasmid or control plasmid, followed by PA treatment (0.3 mM for 24 h). (*n* = 5 independent biological replicates; mean ± SEM). The sequences in the left panel showed the predicted TIA1 binding sequence and its mutant. **F** Representative confocal microscopy images of PA-treated AML12 cells subjected to combined immunofluorescence for TIA1 protein (red) and RNA fluorescence in situ hybridization (RNA-FISH) for *Srebf1* mRNA (green). Nuclei are stained with DAPI (blue). Yellow signals in the merged panel indicate co-localization. Scale bar: 50 μm. **G** *Srebf1* mRNA stability assay. AML12 cells transfected with Ad-TIA1, Ad-GFP, shTIA1, or shNC were treated with actinomycin D to halt transcription, and residual *Srebf1* mRNA levels were measured by qRT-PCR over time (*n* = 3 biologically independent replicates, mean ± SEM). **H**–**J** Functional rescue experiment using the SREBP1 inhibitor PF-429242. AML12 cells infected with shTIA1 or shNC were treated with PA (0.3 mM for 24 h) in the presence or absence of PF-429242 (10 μM for 1 h prior to PA exposure). **H** Representative ORO staining and quantification in indicated groups (*n* = 5 independent biological replicates; mean ± SEM). Scale bar: 50 μm. **I** Western blot analysis of lipogenic proteins (FASN, SREBP1, SCD1, PPARγ). GAPDH served as a loading control. **J** qRT-PCR analysis of lipid metabolism-related gene expression (*Fasn*, *Srebf1*, *Scd1* and *Pparg*), normalized to *Gapdh* (*n* = 5 independent biological replicates; mean ± SEM). **K**–**M** Combined modulation of SGs formation and SREBP1 signaling. AML12 cells were pre-treated with the SGs inducer anisomycin (An, 25 ng/mL for 30 min) and/or PF-429242 (10 μM for 1 h) before PA challenge (0.3 mM for 24 h). **K** Representative ORO staining and quantification in indicated groups (*n* = 5 independent biological replicates; mean ± SEM). Scale bar: 50 μm. **L** Western blot analysis of lipogenic proteins (FASN, SREBP1, SCD1, PPARγ). GAPDH served as a loading control. **M** qRT-PCR analysis of lipid metabolism-related gene expression (*Fasn*, *Srebf1*, *Scd1* and *Pparg*), normalized to *Gapdh* (*n* = 5 independent biological replicates; mean ± SEM). *P < 0.05, **P < 0.01, ***P < 0.001; ****P < 0.0001; ns indicates not significant.

To delineate the molecular mechanism by which TIA1 regulates lipid metabolism, we integrated RIP-seq and RNA-seq datasets, identifying five principal TIA1-interacting transcripts, including *Ndor1*, *Srebf1*, *Gm15787*, *Gm17484*, and *Zfp619* (Fig. 6A). Given the functional enrichment of particular differential transcripts between PA- and BSA treated AML12 cells in lipid synthesis pathways (Fig. 6B), we focused subsequent validation on *Srebf1*, which encodes the master lipogenic transcription factor SREBP1.

Analysis of RIP-seq read densities revealed pronounced enrichment of TIA1 binding within the 3’ untranslated region (3’ UTR) of the *Srebf1* transcript (Fig. 6C). This finding was independently corroborated by analysis of publicly available enhanced Crosslinking and Immunoprecipitation (eCLIP) data from the ENCODE Consortium (HepG2 cells), which also showed significant TIA1 binding peaks within the *Srebf1* 3’ UTR (Figure. S9F). Genome-wide analysis indicated a notable clustering of TIA1 peaks around the *Srebf1* locus on chromosome 17 (Figure S9D, E), further supporting a specific interaction. Detailedly, Figure S9D shows TIA1 binding peaks across chromosomes, with chr2 having the highest peak count (203), followed by chr6 (126), chr11 (125), and chr17 (119). Chromosome 17 is highlighted in red to emphasize the *Srebf1* location. Figure S9E shows the distribution of 119 TIA1 peaks along chromosome 17, spanning from ~3-83 Mb. *Srebf1* location at 17.8 Mb (red dashed line) shows obvious clustering of TIA1 peaks, suggesting preferential binding in this region.

The direct binding of TIA1 to *Srebf1* mRNA in PA-stressed AML12 cells was confirmed by RIP-qPCR (Fig. 6D). Functional relevance was assessed using a dual-luciferase reporter assay, which demonstrated that mutation of predicted TIA1-binding motifs within the *Srebf1* 3’ UTR abolished TIA1-mediated translational repression (Fig. 6E).

FISH (Fig. 6F) revealed that *Srebf1* transcripts co-localize with TIA1-positive aggregates upon PA treatment. According to co-localization analyses (Figure S9G), TIA1 knockdown led to diffuse *Srebf1* mRNA distribution and signal accumulation, indicating impaired sequestration and likely increased translational availability. Mechanistically, TIA1 binding accelerated *Srebf1* mRNA decay, as evidenced by mRNA stability assays showing shortened *Srebf1* half-life upon TIA1 overexpression and prolonged half-life upon its knockdown (Fig. 6G).

Finally, we performed pharmacological rescue experiments using PF-429242, a selective SREBP1 inhibitor. PF-429242 treatment normalized the exacerbated lipid accumulation and the elevated expression of lipogenic effectors (FASN, SCD1, PPARγ) induced by either TIA1 knockdown (Fig. 6H–J; S9H) or chemically induced SG formation SGs disruption (Fig. 6K–M; S9I).

These results establish that TIA1 directly binds the 3’ UTR of *Srebf1* mRNA, promotes its decay, and represses its translation, thereby forming a central TIA1-*Srebf1* mRNA axis that critically constrains hepatic lipogenesis and MASH progression.

### Pharmacological inhibition of SREBP1 rescues the exacerbated MASLD phenotype in TIA1-deficient mice

Immunofluorescence co-localization validated a significant reduction in TIA1 positive stress granules under HKO conditions, which is responsible for the aggravated MALSD phenotype and the hyperacticated SREBP1 (Figure S10A). To determine whether SREBP1 hyperactivation is responsible for the exacerbated MASLD phenotype in vivo, we treated both TIA1-Flox and TIA1-HKO mice with the SREBP1 inhibitor PF-429242 (20 mg/kg/d) or vehicle control for the final 8 weeks of a 12-week HFD regimen. In general, PF-429242 treatment significantly attenuated MASLD progression in both genotypes. Notably, the therapeutic efficacy of PF-429242 was more pronounced in TIA1-HKO mice than in wild-type controls, underscoring the central role of SREBP1 hyperactivation in driving disease severity upon TIA1 loss.

**Fig. 7:**
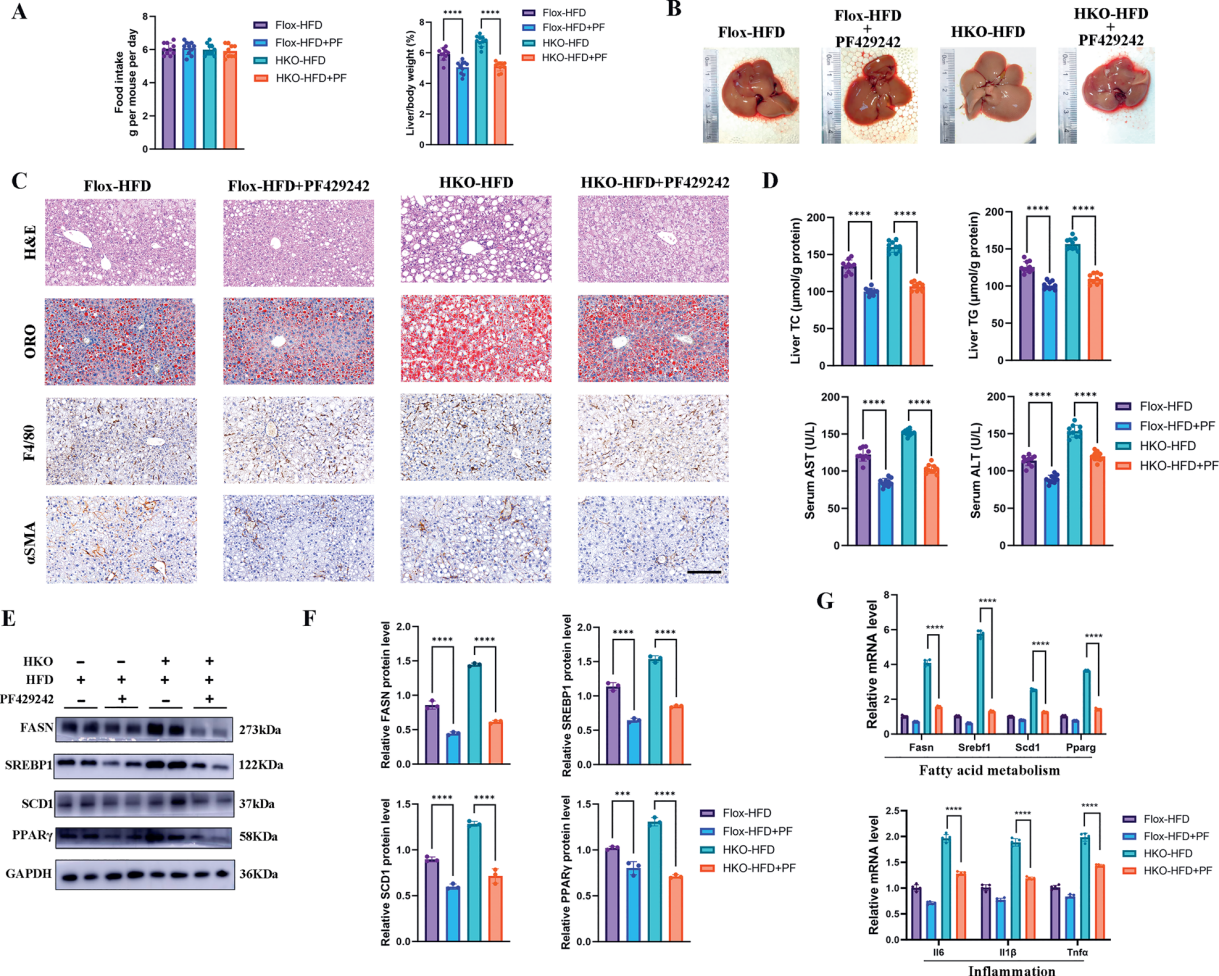
Pharmacological inhibition of SREBP1 rescues the exacerbated MASLD phenotype in TIA1-deficient mice. **A**–**G** TIA1-Flox and TIA1-HKO mice with the SREBP1 inhibitor PF-429242 (20 mg/kg/d) or vehicle control for the final 8 weeks of a 12-week HFD regimen (*n* = 10 mice per group). **A** Average daily food intake measured over 3 consecutive days (Left panel) and liver to body weight ratios (%) (Right panel) in the indicated groups. (*n* = 10 per group; mean ± SEM). **B** Representative gross morphological photographs of livers from the indicated experimental groups, showing that PF-429242 treatment ameliorates the hepatomegaly and pale appearance in HKO-HFD mice. **C** Representative photomicrographs of liver sections stained with H&E, ORO, F4/80, and αSMA. Scale bar: 100 μm. **D** Biochemical analyses of hepatic TC and TG levels, and serum AST and ALT activities (*n* = 10 per group; mean ± SEM). **E** Western blot analysis of key proteins involved in fatty acid metabolism (FASN, mature SREBP1, SCD1, and PPARγ) in liver lysates from the indicated groups. GAPDH served as a loading control. **F** Densitometric quantification of western blotting results shown in (**E**). (*n* = 3 biologically independent mice per condition; mean ± SEM). **G** Hepatic mRNA expression levels of genes related to lipid metabolism (*Fasn, Srebf1, Scd1* and *Pparg*), and inflammation (*Il1β, Il6* and *Tnfα*), normalized to *Gapdh*. (*n* = 5 biologically independent mice per condition; mean ± SEM). *P < 0.05, **P < 0.01, ***P < 0.001; ****P < 0.0001; ns indicates not significant.

Compared to vehicle-treated TIA1-HKO mice, those receiving PF-429242 exhibited a reduced liver-to-body weight ratio (13.8% decrease, *P* < 0.001) and improved gross liver morphology, with diminished hepatomegaly and pallor (Fig. 7A, B; S10B). Histological analysis confirmed substantial improvement. H&E staining revealed markedly reduced macrovesicular lipid droplets and lower NAS in PF-429242-treated TIA1-HKO mice (Fig. 7C; S10C). Oil Red O staining demonstrated significantly decreased neutral lipid accumulation (30.4% reduction, *P* < 0.0001) (Fig. 7C; S10D). Immunohistochemical analyses showed attenuated αSMA-positive myofibroblast activation (34.3% decrease, *P* < 0.05) and reduced F4/80-positive macrophage infiltration (25.6% decrease, *P* < 0.001) (Fig. 7C; S10D).

Biochemical profiling corroborated these morphological improvements. PF-429242 treatment reduced hepatic TC (20.5% decrease, *P* < 0.001) and TG (19.4% decrease, *P* < 0.001) levels, paralleled by decreases in serum TC (10.6% decrease, *P* < 0.001) and TG (15.2% decrease, *P* < 0.001) (Fig. 7D; S10D). Serum AST (16.7% decrease, *P* < 0.001) and ALT (19.5% decrease, *P* < 0.001) levels were significantly normalized, indicating reduced hepatocellular injury (Fig. 7D). The treatment also restored hepatic antioxidant capacity, increasing GSH content (85.7% increase, *P* < 0.001) and SOD activity (37.9% increase, *P* < 0.001) (Figure. S10E).

At the molecular level, PF-429242 effectively reversed the heightened expression of lipogenic (*Fasn, Srebdf1, Scd1* and *Pparg*) and pro-inflammatory (*Il1β, Il6* and *Tnfα*) genes that characterized HFD-fed TIA1-HKO mice (Fig. 7E–G).

These results demonstrate that SREBP1 inhibition robustly rescues the exacerbated MASLD phenotype in TIA1-deficient mice. The markedly enhanced therapeutic response in the knockout background confirms that SREBP1 hyperactivation is the principal mechanistic driver of metabolic dysregulation following the loss of TIA1-mediated repression.

## Discussion

Recent reappraisal of disease nomenclature has positioned MASLD, formerly termed NAFLD, as a preeminent focus within biological, pharmacological, pharmaceutical, and clinical research domains [34, 35]. The escalating global prevalence of obesity and type 2 diabetes has precipitated a concurrent rise in MASLD, substantially compromising human health while imposing significant socioeconomic burdens [36]. Consequently, MASLD pathogenesis has emerged as a critical research priority. Our investigation identifies TIA1 as a hepatoprotective regulator and a promising therapeutic target that negatively modulates MASLD/MASH progression.

Our initial impetus for investigating TIA1 derived from integrated bioinformatic interrogation of clinical hepatic specimens and publicly accessible repositories (GSE160016, GSE130970, GSE193080), which revealed significant TIA1 upregulation negatively correlated with disease severity and adverse prognoses in MASH patients. An intriguing and seemingly paradoxical observation from our study and public data is that high TIA1 expression is associated with MASLD/MASH, yet lower expression correlates with increased risk of HCC progression. We propose a model wherein TIA1 serves as a dynamic stress responder: initially upregulated to maintain homeostasis by sequestering pro-lipogenic and inflammatory transcripts, but ultimately succumbing to chronic lipotoxic stress. In early MASLD, TIA1 is upregulated as a protective stress response, attempting to maintain cellular homeostasis by sequestering and regulating transcripts involved in lipid synthesis and inflammation. However, its chronic engagement and eventual functional impairment (e.g., through sustained lipotoxic stress) could contribute to metabolic dysfunction, making its presence a marker of stress but not necessarily of functional efficacy. In later stages of HCC, complete loss of TIA1 function may be selected for, as its roles in promoting apoptosis and suppressing the translation of pro-oncogenic transcripts would be detrimental to tumor survival and proliferation [37–39]. Thus, its downregulation in advanced HCC signifies a loss of tumor-suppressive functions. This model positions TIA1 not as a straightforward biomarker but as a dynamic mediator whose functional outcome is critically dependent on the disease context. Complementary experimental analyses demonstrated concordant TIA1 elevation in multiple murine MASH models induced by HFD, MCD, and HFHC diet regimens. Hepatocyte-specific TIA1 expression under MASH-associated pathological conditions was established through albumin co-staining in healthy murine liver sections, further corroborated by single-cell RNA-sequencing dataset analysis.

Critically, our investigation demonstrated that hepatocyte-specific TIA1 knockout substantially aggravated MASH histopathological, biochemical, and molecular biological features across all dietary murine models, while TIA1 overexpression via adeno-associated virus serotype 8 (AAV8)-mediated gene delivery conferred significant hepatic protection. Given the established clinical success of AAV vectors in therapeutic gene manipulation [40], AAV-mediated TIA1 reconstitution represents a viable translational strategy for mitigating steatosis and inflammation in MASH.

Complementing these in vivo observations, PA-challenged AML12 hepatocytes exhibited TIA1 upregulation, prompting subsequent RIP-seq analysis to delineate its downstream regulatory circuitry. GO analysis of differential expressed binding peaks under metabolic stress revealed TIA1-bound mRNAs primarily enriched in translational regulation, RNA stabilization, mRNA catabolic processes, and cytoplasmic ribonucleoprotein granule organization. Genomic binding peak distribution analysis further demonstrated preferential TIA1 occupancy within 3’ UTRs.

Pathological RNA granule formation represents a well-established neuropathological mechanism, with TIA1 co-localization documented in Alzheimer’s disease (AD), frontotemporal dementia with parkinsonism (FTDP-17), frontotemporal lobar dementia (FTLD-TDP), amyotrophic lateral sclerosis (ALS), Huntington’s disease, Creutzfeld-Jakob disease, and spinomuscular atrophy, observations recapitulated across corresponding animal models [41, 42]. Cytoplasmic SGs constitute dynamic ribonucleoprotein aggregates that form in response to diverse environmental stressors, including lipotoxicity. SGs involvement has been explored across MASLD pathogenesis contributors including obesity, type 2 diabetes mellitus (T2DM), genetic predisposition, and microbiome dysbiosis. Adipocytes exposed to elevated fatty acids or glucose initiate SGs formation as an adaptive mechanism to suppress global translation during proteotoxic stress [43], while SGs dysregulation contributes to diabetic pathogenesis [44]. Genetic polymorphisms affecting SGs dynamics may further modify cellular stress responses, potentially influencing obesity and diabetes susceptibility [45, 46]. Despite growing recognition in metabolic disorders, SGs biology, particularly TIA1 involvement, remains unexplored in MASH context, which progress from simple steatosis to steatohepatitis, fibrosis, and ultimately cirrhosis involves chronic inflammation, lipotoxicity, insulin resistance, and oxidative stress responses.

We then validated that hepatocytes employ SGs formation as a cytoprotective adaptation against lipid-induced cellular damage. Our in vitro study uncovered that the hepatoprotective function of TIA1 during lipotoxicity is regulated at least at two levels: protein abundance and subcellular localization. We observed a significant increase in total TIA1 protein levels upon PA treatment. Concurrently, TIA1 underwent a pronounced redistribution from the nucleus to the cytoplasm. This stress-induced nucleocytoplasmic translocation is functionally critical, as it delivers TIA1 to the site of action—the forming stress granules. The dual response of upregulation and translocation likely serves to amplify the cell’s capacity to sequester target transcripts (such as *Srebf1* mRNA) efficiently, thereby robustly suppressing lipogenic flux. While the precise signaling that triggers this translocation requires further investigation, it underscores that TIA1’s role as an RNA buffer is dynamically regulated by its compartmentalization in response to metabolic stress.

The differential capacity of fatty acids to induce SGs formation in hepatocytes reveals a diversiform cellular stress response. While PA robustly triggered SGs assembly in AML12 cells—consistent with its well-documented role in inducing ER stress and oxidative stress [47, 48]—co-treatment with OA or OA alone markedly attenuated this response (Figure S6A–C). This observation aligns with the established cytoprotective properties of OA. Unlike saturated fatty acids such as PA, OA, a monounsaturated fatty acid, has been shown to alleviate lipotoxicity and cellular stress [49, 50]. We therefore postulate that the inability of PA + OA or OA to induce SGs is probably a consequence of OA’s efficacy in mitigating the primary triggers of stress response. Specifically, OA may dampen the ER stress and redox imbalance that are necessary for the nucleation of SGs. This protective effect could be mediated through mechanisms such as the modulation of membrane fluidity [51], activation of adaptive signaling pathways [52], or direct reduction in reactive oxygen species generation [53]. Our findings underscore that the metabolic fate and stress-inducing potential of fatty acids are critically determined by their saturation state. Future studies identifying the precise molecular switch whereby OA diversion into neutral lipids quells the stress signal for SGs formation will deepen our understanding of metabolic stress resolution in hepatocytes.

Our experimental data further revealed that chemically induced SG formation exacerbated PA-induced lipid deposition and lipogenic gene expression, whereas SGs induction conversely attenuated these phenotypes. Consistently, TIA1 knockdown substantially impaired SGs formation under metabolic stress, aggravating oxidative stress and lipid accumulation, while rescued by SGs agonist administration. Sodium arsenite activates SGs assembly via oxidative stress-induced eukaryotic initiation factor 2α (eIF2α) phosphorylation and translational arrest [54, 55], though its hepatotoxicity presents clinical limitations [56]. Arsenic compounds influence diverse cellular processes including apoptosis, proliferation, inflammation, angiogenesis, and immune responses [57–59], and despite historical applications in traditional medicine for dental, rheumatological, dermatological, and infectious diseases [60, 61], contemporary utility remains restricted primarily to acute promyelocytic leukemia therapy due to its narrow therapeutic window [62, 63].

An intriguing observation from our study is that sodium arsenite treatment effectively reduced lipid accumulation even under TIA1-downregulated conditions. This result suggests that the lipid-lowering effect of sodium arsenite may not be exclusively mediated through the TIA1/SREBP1 axis, implicating the involvement of additional or parallel regulatory pathways. Firstly, sodium arsenite may directly activate AMP-activated protein kinase (AMPK), a master regulator of cellular energy homeostasis. AMPK activation phosphorylates and inhibits key lipogenic enzymes such as acetyl-CoA carboxylase (ACC) and suppresses SREBP1 activity, thereby promoting fatty acid oxidation over synthesis, independently of TIA1’s RNA-regulatory function [64, 65]. Besides, sodium arsenite could potently stimulate alternative lipid clearance pathways, such as lipophagy (selective autophagic degradation of lipid droplets) or lipolysis via activating enzymes like adipose triglyceride lipase (ATGL). The potent activation of these clearance pathways could overwhelm any pro-lipogenic effects resulting from TIA1 downregulation [66].

Mechanistic integration of RIP-seq, RIP-PCR and RNA-seq data identified *Srebf1* mRNA as a direct TIA1 target, featuring differential binding peaks within its 3’ UTR. The hallmark of MASLD pathogenesis involves excessive hepatocyte triglyceride (TG) accumulation via heightened de novo lipogenesis (DNL), plasma free fatty acid (FFA) esterification, or dietary fatty acid uptake [67]. Substantial evidence implicates augmented hepatic DNL—demonstrating threefold elevation in MASLD patients via isotope-labeling studies [68]—as a principal pathogenic contributor, though regulatory mechanisms remain incompletely characterized. SREBP1 serve as master transcriptional regulators of DNL pathway genes, playing pivotal roles in MASLD development [69]. RIP-PCR and luciferase reporter assays further established that TIA1 directly bind to *Srebf1* mRNA 3’ UTR and suppresses its transcriptional activity. Mechanistically, we demonstrated that *Srebf1* mRNA is recruited to TIA1-positive SGs under PA-induced metabolic stress based on FISH. This specific partitioning was enhanced by canonical SG inducers and crucially abolished upon TIA1 knockdown, which resulted in the diffuse cytosolic distribution of *Srebf1* transcripts. These observations suggest that TIA1-mediated sequestration into SGs is a primary event limiting the accessibility of Srebf1 mRNA to the translational machinery. Beyond spatial control, we provide mechanistic evidence that TIA1 binding accelerates the decay of *Srebf1* mRNA. The measured shortening of *Srebf1* half-life upon TIA1 overexpression, and its concomitant prolongation after TIA1 knockdown, directly links TIA1 activity to the mRNA’s turnover rate. Collectively, these findings support a dual-layer regulatory model: TIA1 first temporarily silences *Srebf1* translation by sequestering its mRNA within SGs, which then facilitates its commitment to the mRNA decay pathway, thereby ensuring the suppression of SREBP1 protein production and downstream lipogenic signaling.

To establish a direct causal link between the loss of TIA1, unrestrained SREBP1 activity, and the ensuing metabolic pathology, we performed definitive rescue experiments both in vitro and in vivo. Apart from the normalization of the exacerbated lipid accumulation and the elevated expression of lipogenic effectors induced by either TIA1 knockdown or chemically induced SG formation in PA-treated AML12 cells, pharmacological inhibition of SREBP1 with PF-429242 in HFD-fed TIA1-HKO mice effectively reversed the exacerbated steatosis, inflammation, and metabolic dysfunction. Crucially, the therapeutic efficacy of PF-429242 was markedly more pronounced in TIA1-deficient mice than in wild-type controls, which is a particularly informative genotype-dependent response. It demonstrates that the severe MASLD phenotype in TIA1-HKO mice is not merely an amplification of general diet-induced stress but is specifically attributable to the hyperactivation of the SREBP1 pathway upon loss of TIA1-mediated repression. This in vivo rescue successfully mirrors our cellular findings where SREBP1 inhibition rectified the lipid accumulation caused by TIA1 knockdown or SGs disruption. The concordance between in vitro and in vivo models solidifies the linearity of the TIA1-SGs assembly-SREBP1 translational suppression axis and underscores its physiological relevance in maintaining hepatic lipid homeostasis. Therefore, our genetic and chemical evidence converges to position SREBP1 not merely as a correlative downstream factor but as the principal mechanistic effector responsible for the metabolic havoc wreaked by TIA1 deficiency. This conclusion significantly strengthens our proposed model and transforms the TIA1-SGs-SREBP1 axis from a descriptive pathway into a druggable target for therapeutic intervention.

Collectively, these results elucidate TIA1’s molecular role in hepatocyte lipid homeostasis and establish the mechanistic linkage between SGs dynamics and MASLD progression. Our findings define the TIA1-SREBP1 regulatory axis as a crucial intracellular signaling module that functionally connects SGs formation to lipogenesis suppression, suggesting hepatocyte TIA1 maintenance represents a promising therapeutic strategy for mitigating oxidative stress and lipid peroxidation in MASH.

While our current data robustly establish the TIA1/SGs/SREBP1 axis as a primary target, these potential auxiliary mechanisms warrant further investigation. Future studies aimed at identifying the direct molecular targets of SGs and dissecting their relative contributions will provide a more holistic understanding of its therapeutic potential against NAFLD.

### Study Limitations

Several study limitations warrant acknowledgement. First, the TIA1-SGs-MASLD relationship necessitates validation in large-scale clinical cohorts correlating hepatic TIA1 expression with MASLD, MASH severity. Due to the limited availability of clinical samples, single-cell RNA sequencing was not performed in this study. While our analysis of the GepLiver atlas indicated TIA1 expression in hepatocytes, its signal may be low or inconsistent in other references, such as the LiverCellAtlas. This is likely attributable to dropout effects (where transcripts are not detected due to limited sequencing depth) and differences in data filtering thresholds or clustering algorithms applied by different atlas projects. This does not necessarily indicate the absence of the protein, as post-transcriptional regulation and the sensitivity of protein detection methods can differ. Secondly, a specific mechanism deserves to be further investigated. The formation of SGs is thought to temporarily store translationally blocked mRNAs, maintain intracellular nucleic acid homeostasis and regulate mRNA degradation [70, 71]. Our present study found that TIA1 controls hepatic lipid accumulation and inflammation through the regulation of *Srebf1* mRNA translation; therefore, we hypothesise that lipotoxicity induces the persistence of hepatocyte SGs, resulting in a decrease in SREBP1 expression by encapsulating *Srebf1* mRNA and inhibiting its translation. However, after stress relief, these membrane-free organelles are quickly disassembled to release the captured mRNA for use by the cell [72, 73]. Thus, further research is needed for a more detailed explanation of this process.

Another key limitation of our study is that all in vivo experiments were conducted using male mice. While this approach allowed us to define the TIA1/SREBP1 regulatory axis within a controlled, high-susceptibility context, it necessarily limits the generalizability of our findings. Sexual dimorphism in MASLD is well-established, with premenopausal females exhibiting relative protection attributed largely to the actions of estrogen [74, 75]. Estrogen signaling is known to modulate hepatic lipid metabolism and inflammation through pathways that may intersect with our proposed mechanism, such as regulating ER stress and de novo lipogenesis [76]. Therefore, it remains to be determined whether the exacerbation of MASLD upon TIA1 deletion and its rescue by SREBP1 inhibition are equally salient in female mice, or if protective hormonal milieu attenuates this pathway. Future studies employing both sexes, with attention to hormonal status (e.g., ovariectomized models with or without estrogen supplementation), are essential to establish the broader relevance of our findings and to understand potential sex-biased therapeutic implications. This work provides a foundational mechanistic insight primarily relevant to male hepatic biology, which must now be tested in a sex-specific context.

It is also important to acknowledge the inherent technical limitations of RIP-seq in definitively identifying RNA-binding protein targets. The resolution of RIP-seq is relatively low, preventing precise mapping of binding sites to specific nucleotide sequences or regions. Furthermore, the method is susceptible to false positives due to non-specific antibody interactions or the co-precipitation of RNAs associated with large protein complexes rather than directly bound by TIA1. Future studies utilizing crosslinking-based techniques such as CLIP-seq or iCLIP in relevant cell models could provide higher-resolution mapping of TIA1-RNA interactions.

## Supplementary information

Figure S1

Figure S2

Figure S3

Figure S4

Figure S5

Figure S6

Figure S7

Figure S8

Figure S9

Figure S10

Table S1

Table S2

Table S3

Table S4

Supplementary Materials and Methods

Supplementary Figure Legends

Original Blot

Unprocessed data

Represent Raw Images

## Data Availability

The data that support the findings of this study are available from the corresponding authors upon reasonable request. The datasets generated and/or analyzed during the current study are available from the corresponding author on reasonable request.
