# Supplementary figures and images for: Hepatocyte TIA1 constrains metabolic steatohepatitis by translationally suppressing Srebf1 mRNA in stress granules
Source: Cell Death Dis. 2026 Mar 24;17(1):357. doi: 10.1038/s41419-026-08682-5 (PMC13039281; doi:10.1038/s41419-026-08682-5)

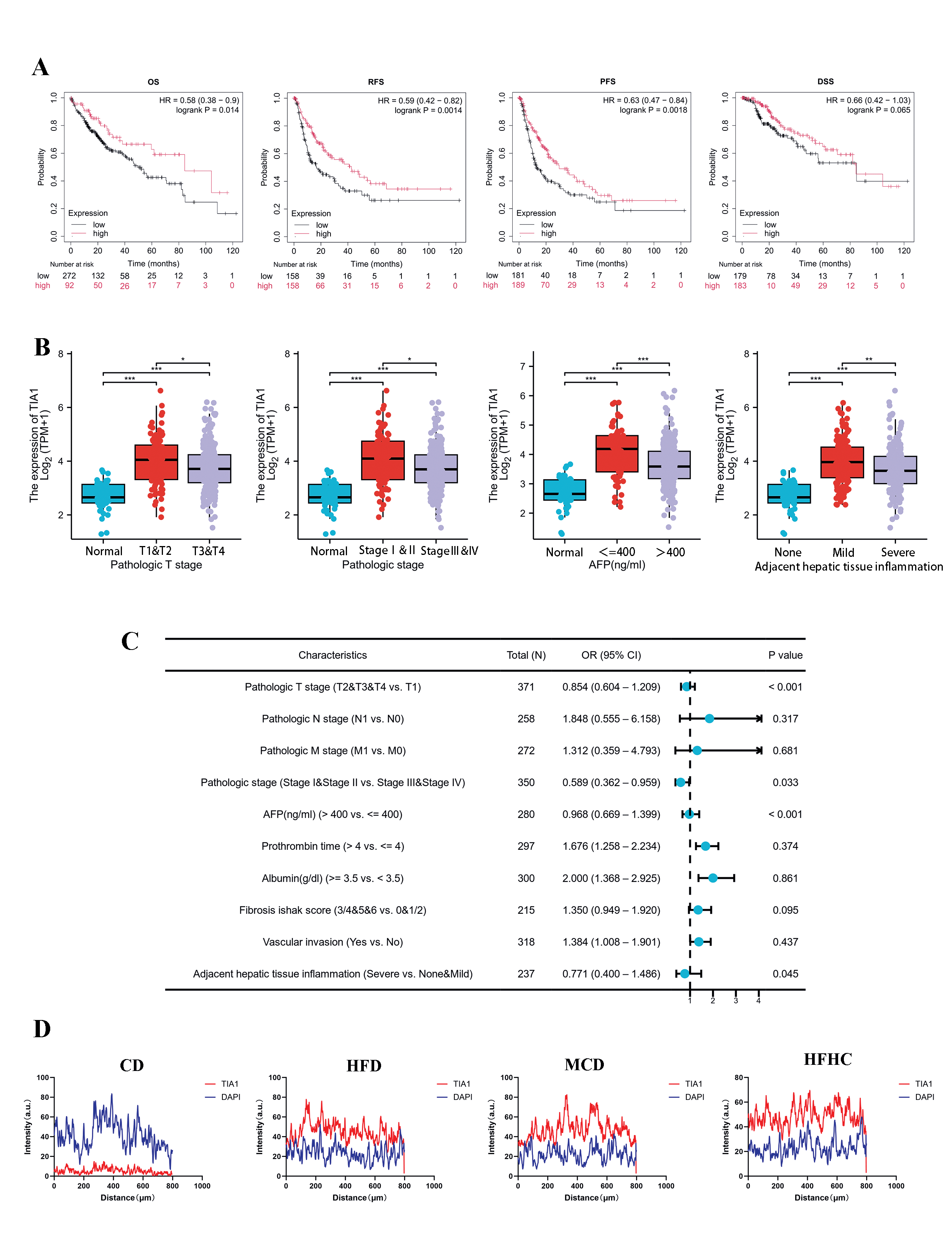

Supplement: Supplementary file 1 — Figure S1 [file 41419_2026_8682_MOESM1_ESM.png]

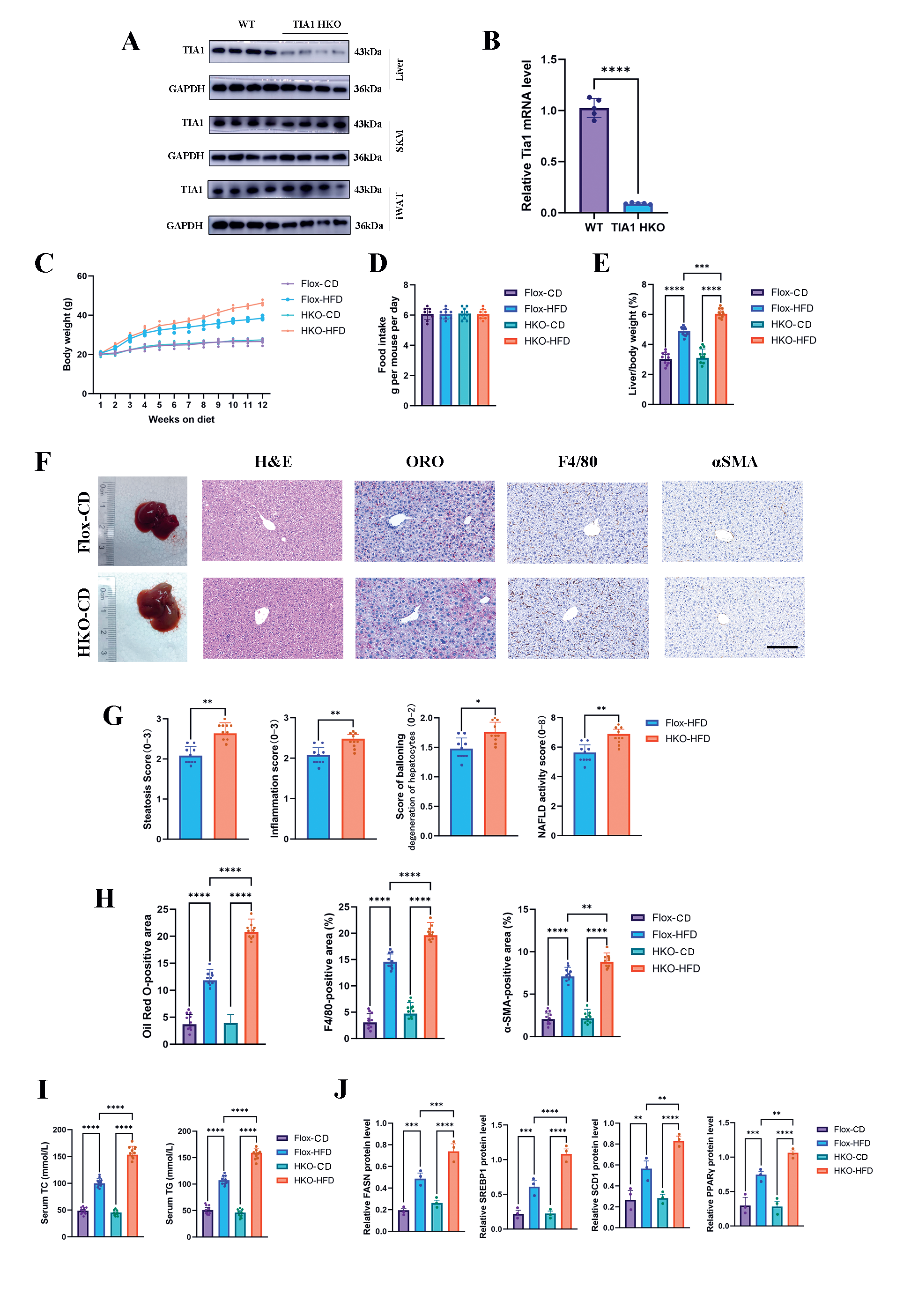

Supplement: Supplementary file 2 — Figure S2 [file 41419_2026_8682_MOESM2_ESM.png]

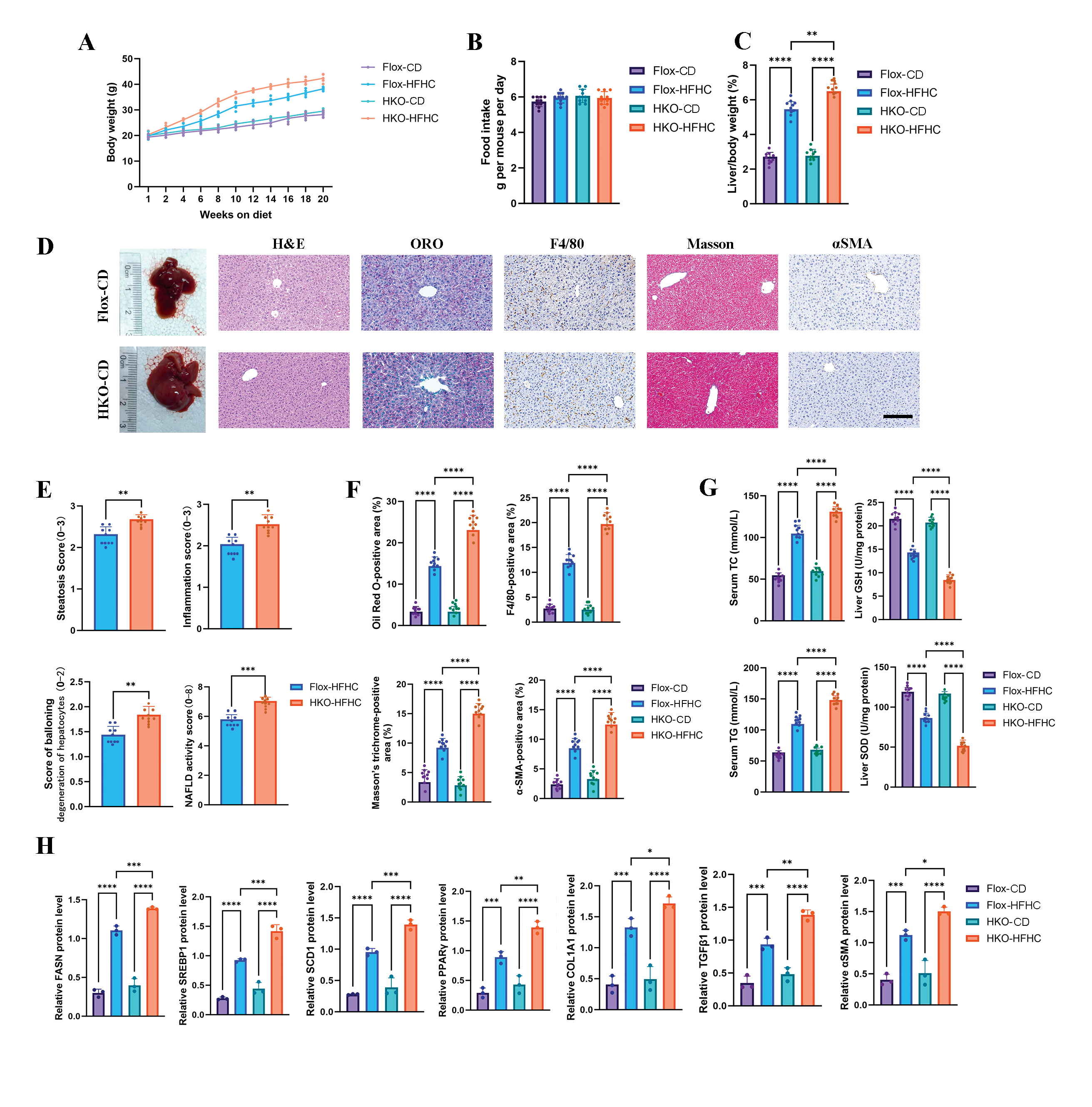

Supplement: Supplementary file 3 — Figure S3 [file 41419_2026_8682_MOESM3_ESM.png]

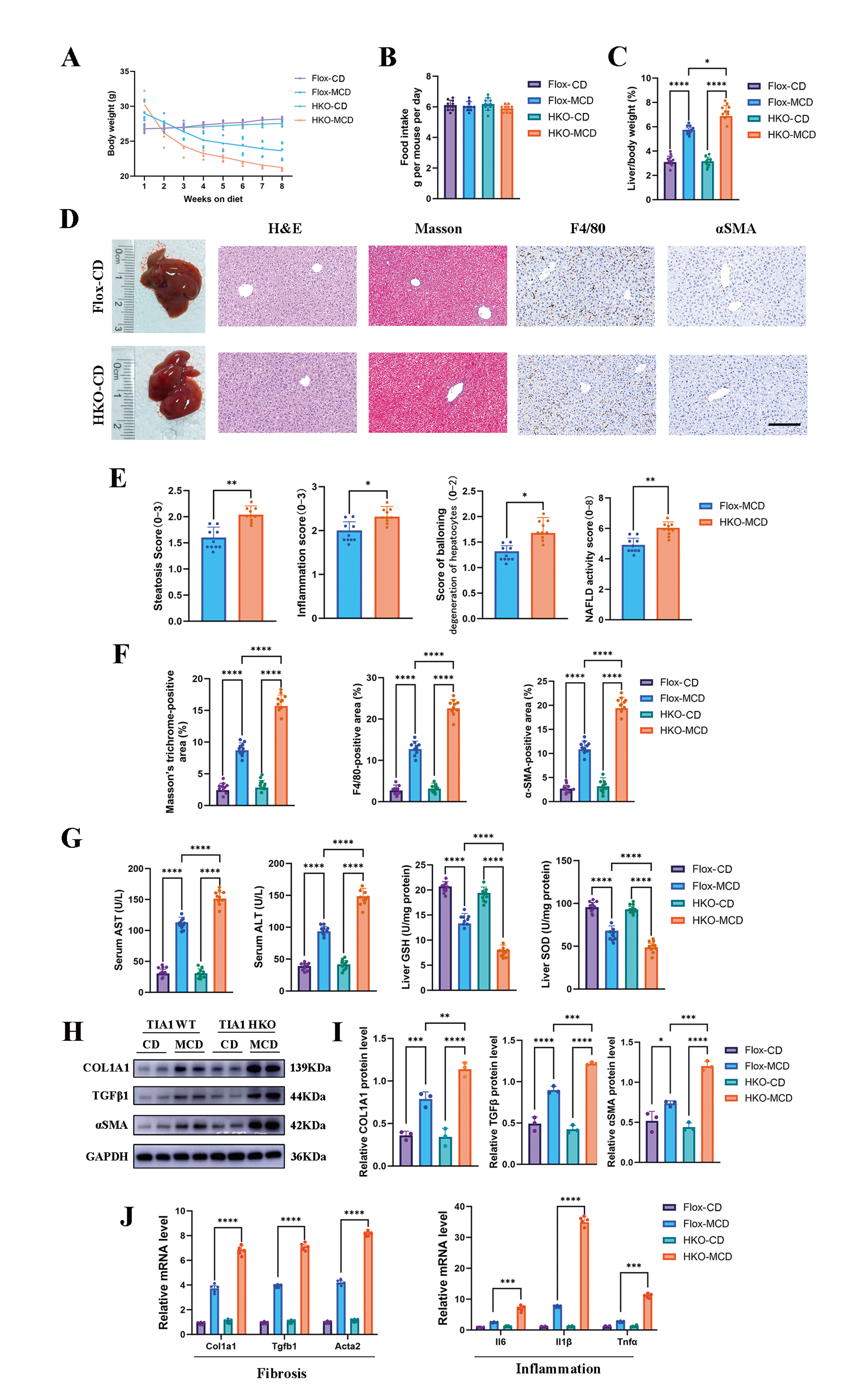

Supplement: Supplementary file 4 — Figure S4 [file 41419_2026_8682_MOESM4_ESM.png]

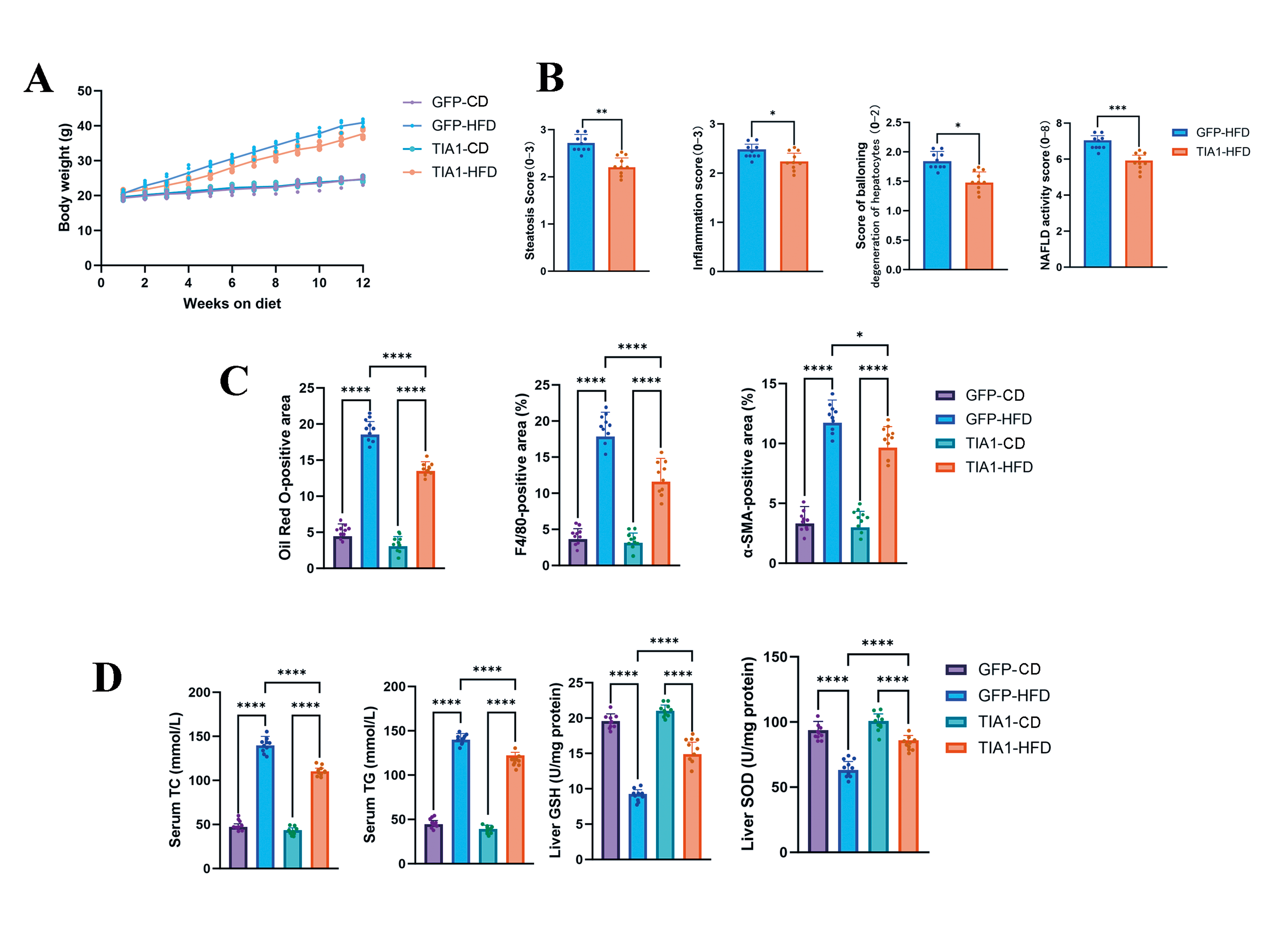

Supplement: Supplementary file 5 — Figure S5 [file 41419_2026_8682_MOESM5_ESM.png]

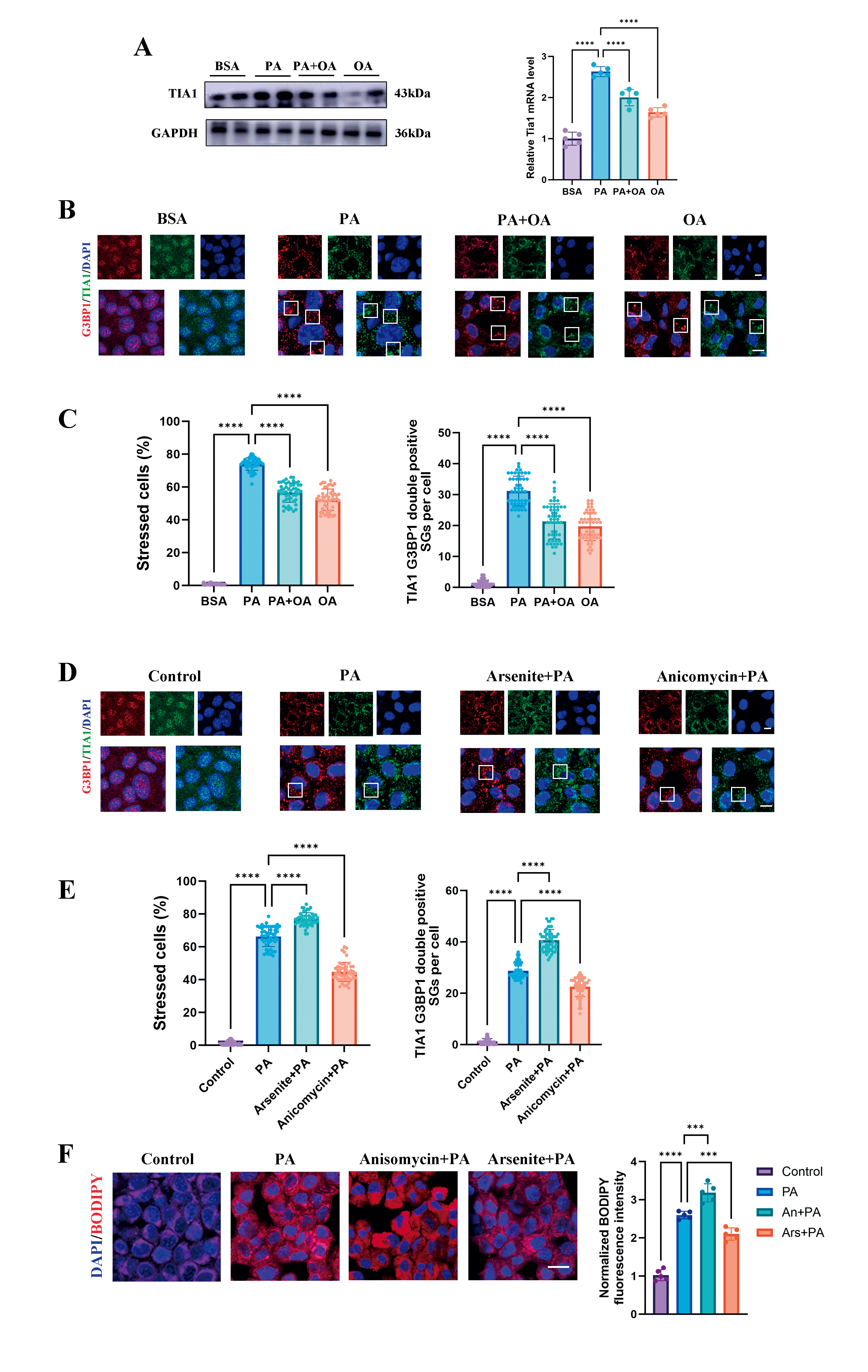

Supplement: Supplementary file 6 — Figure S6 [file 41419_2026_8682_MOESM6_ESM.png]

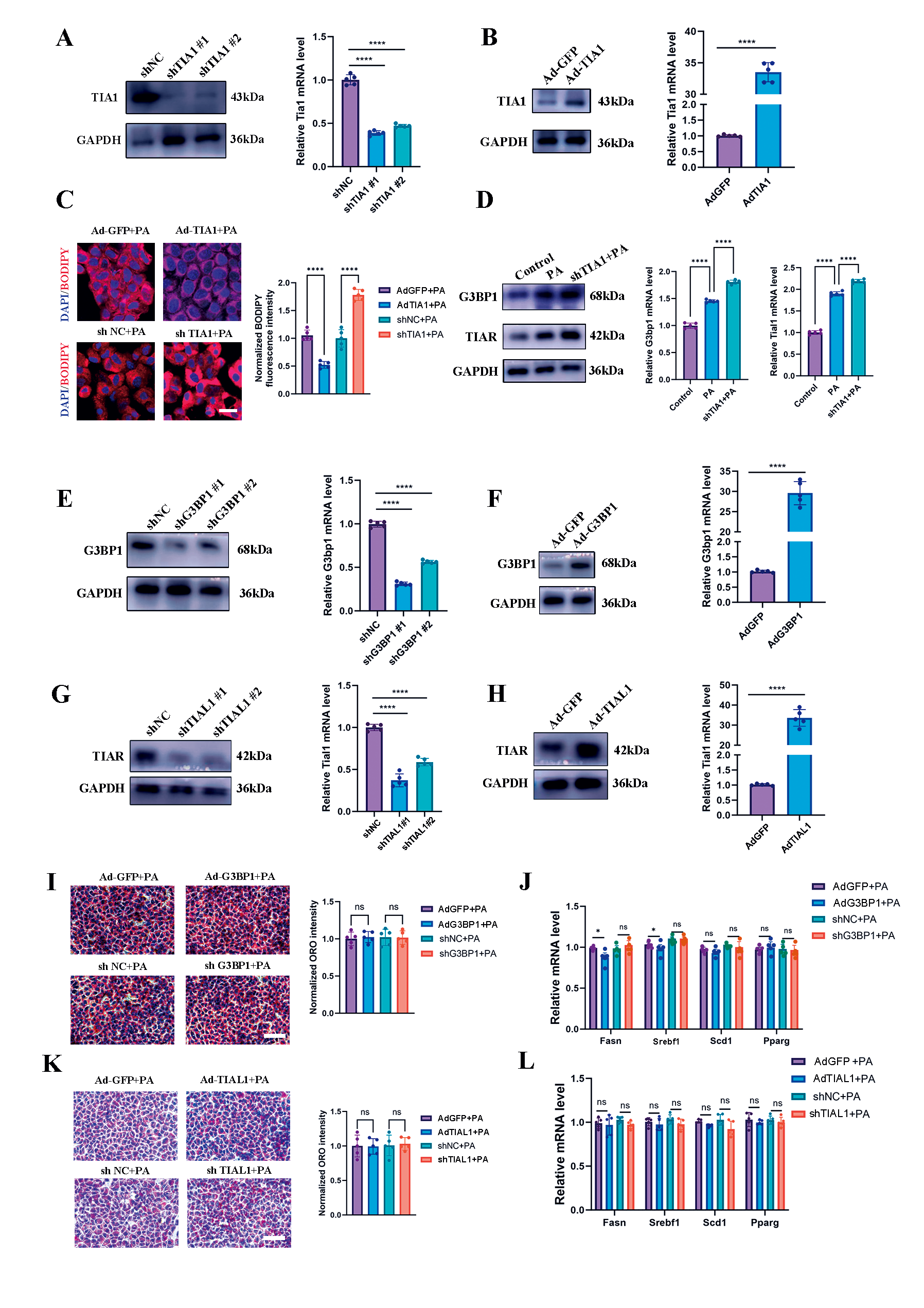

Supplement: Supplementary file 7 — Figure S7 [file 41419_2026_8682_MOESM7_ESM.png]

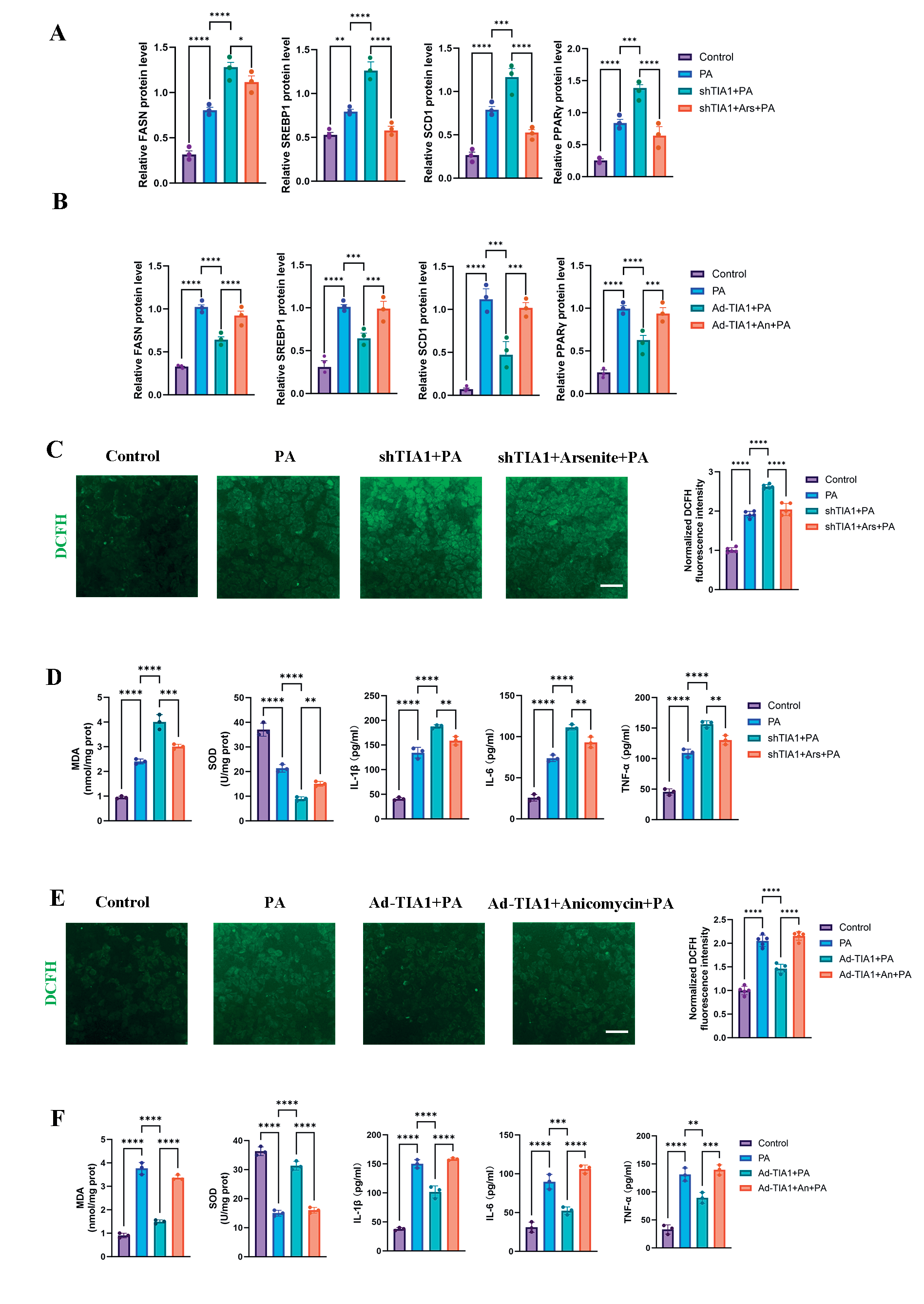

Supplement: Supplementary file 8 — Figure S8 [file 41419_2026_8682_MOESM8_ESM.png]

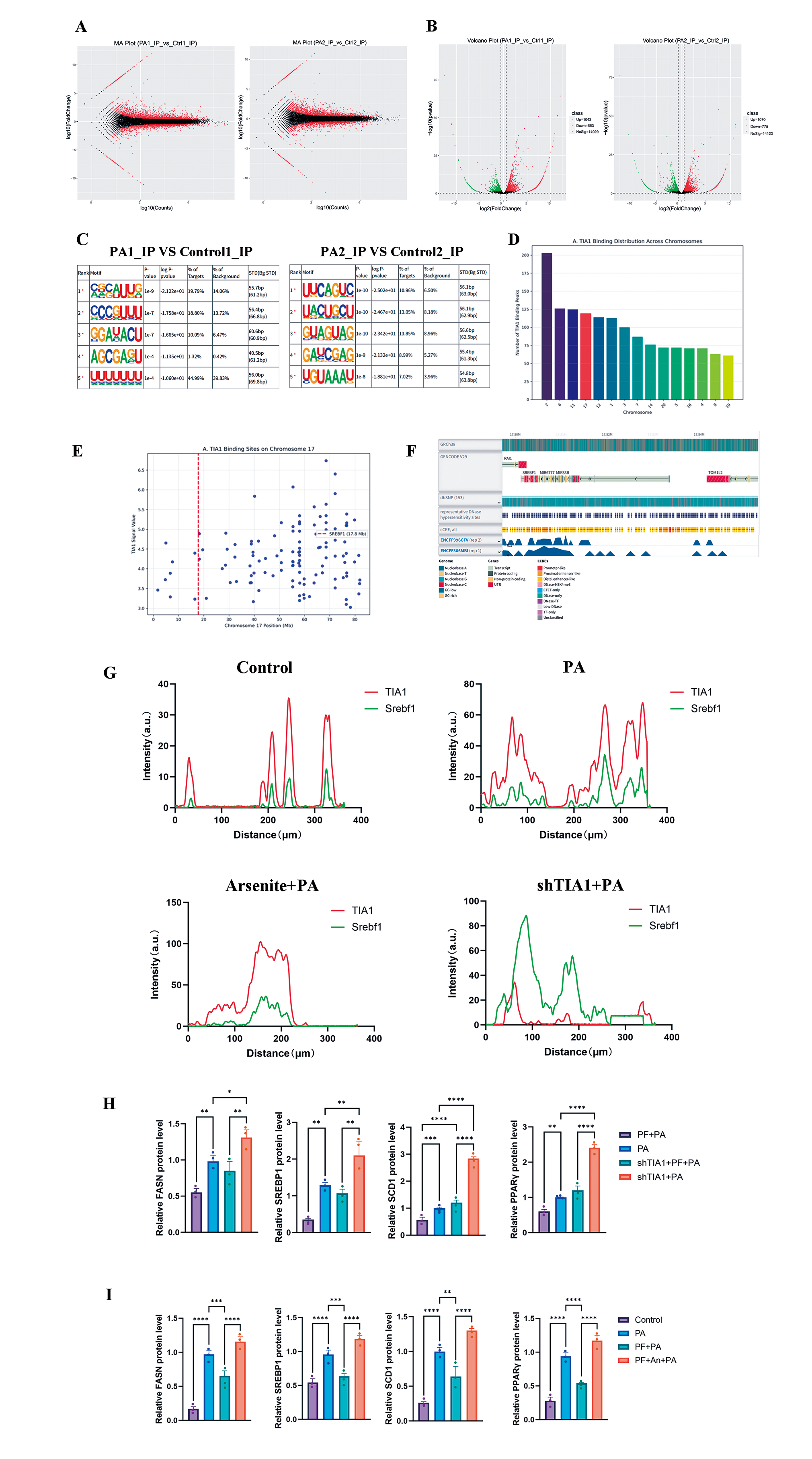

Supplement: Supplementary file 9 — Figure S9 [file 41419_2026_8682_MOESM9_ESM.png]

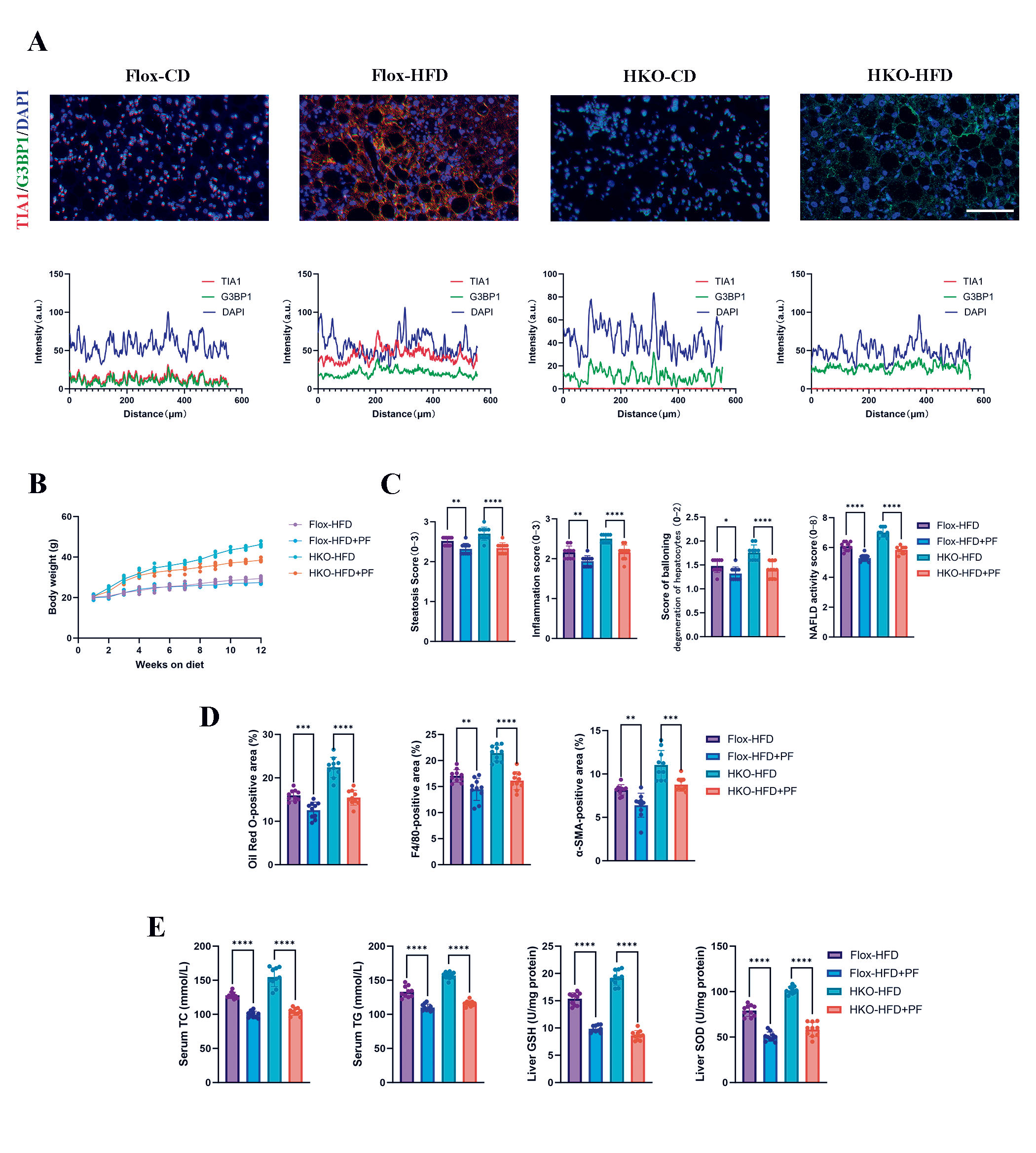

Supplement: Supplementary file 10 — Figure S10 [file 41419_2026_8682_MOESM10_ESM.png]

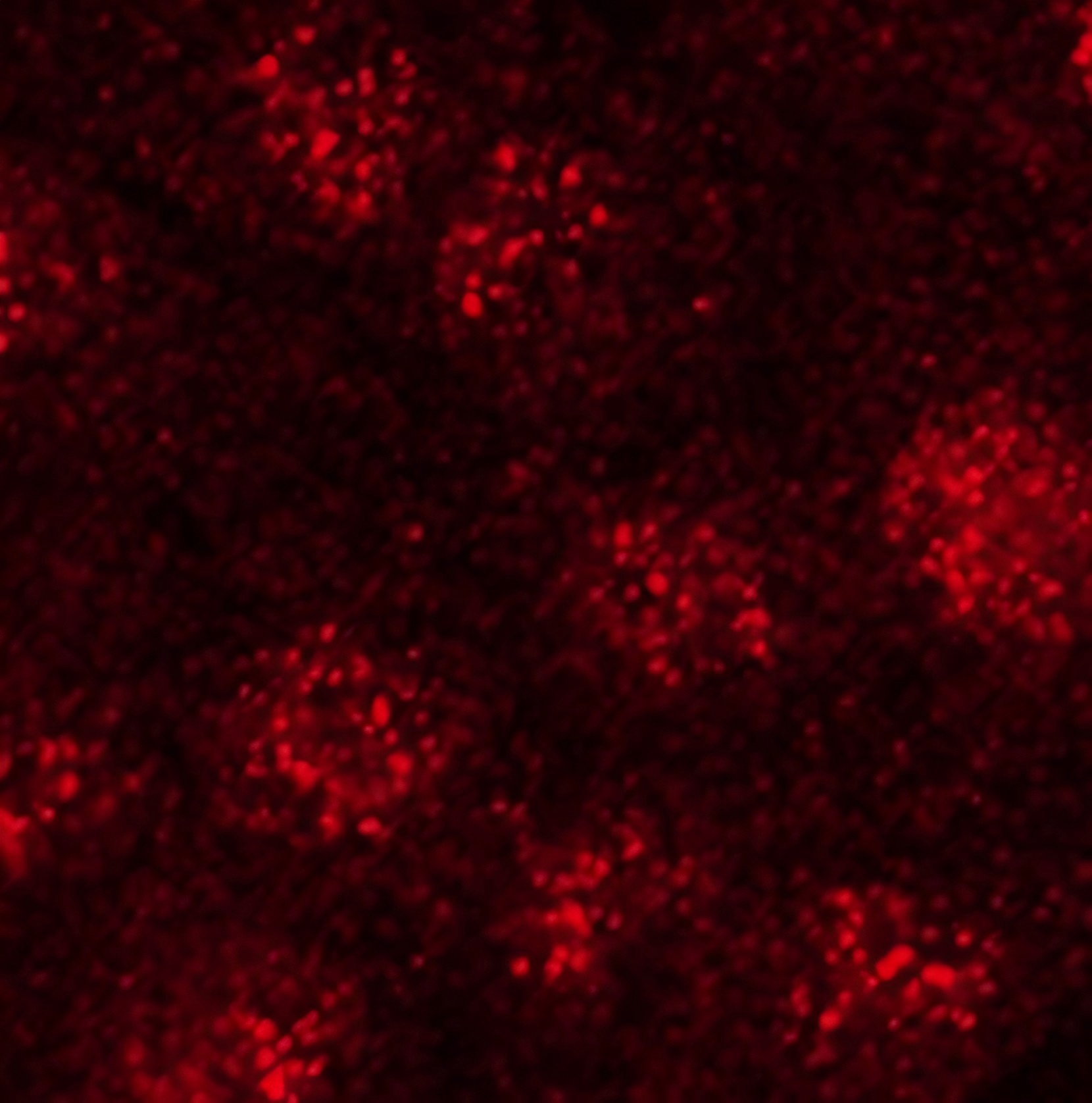

Supplement: Supplementary file 19 — Represent Raw Images [file 41419_2026_8682_MOESM19_ESM.zip › IFRAW/1-1-1.bmp]

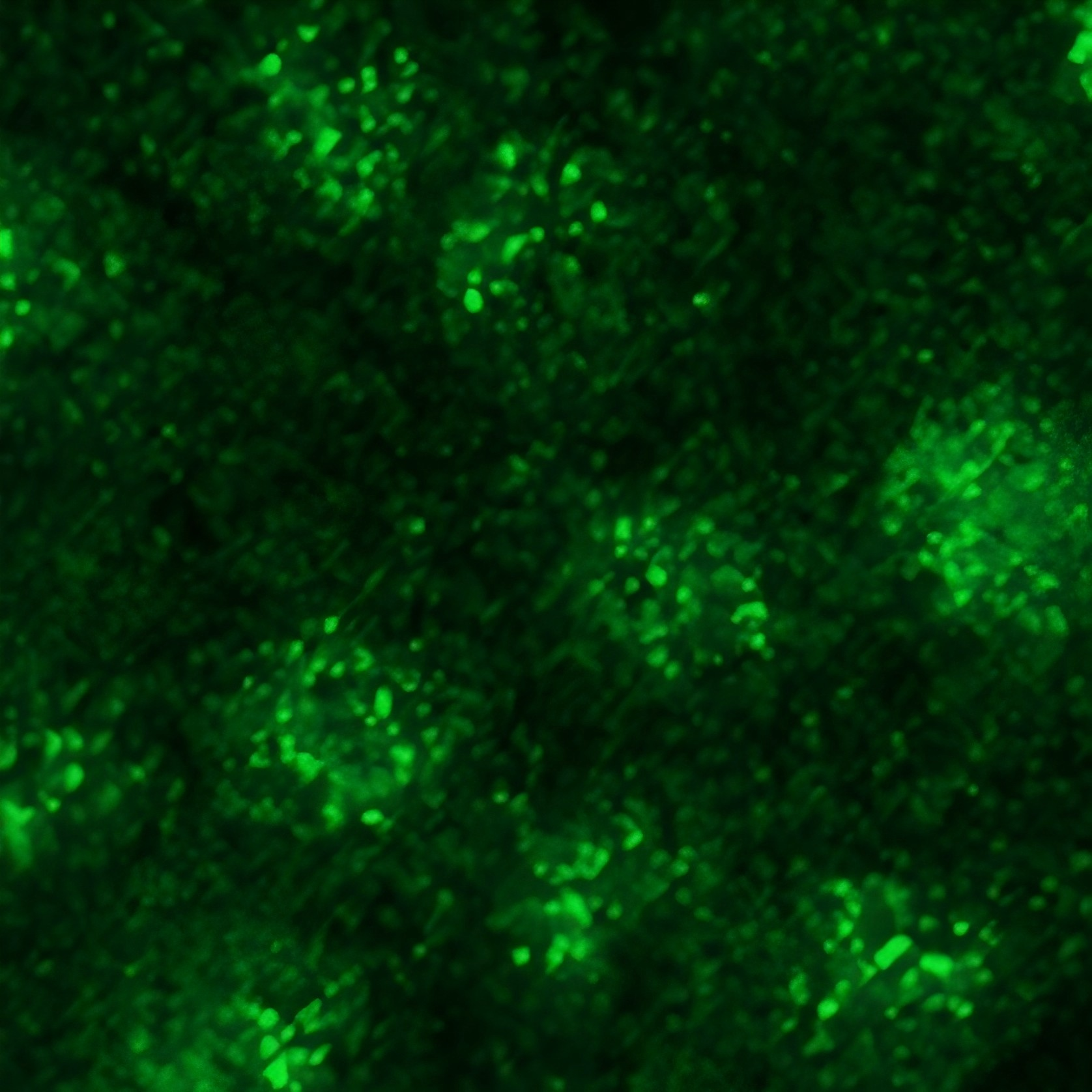

Supplement: Supplementary file 19 — Represent Raw Images [file 41419_2026_8682_MOESM19_ESM.zip › IFRAW/1-1-2.bmp]

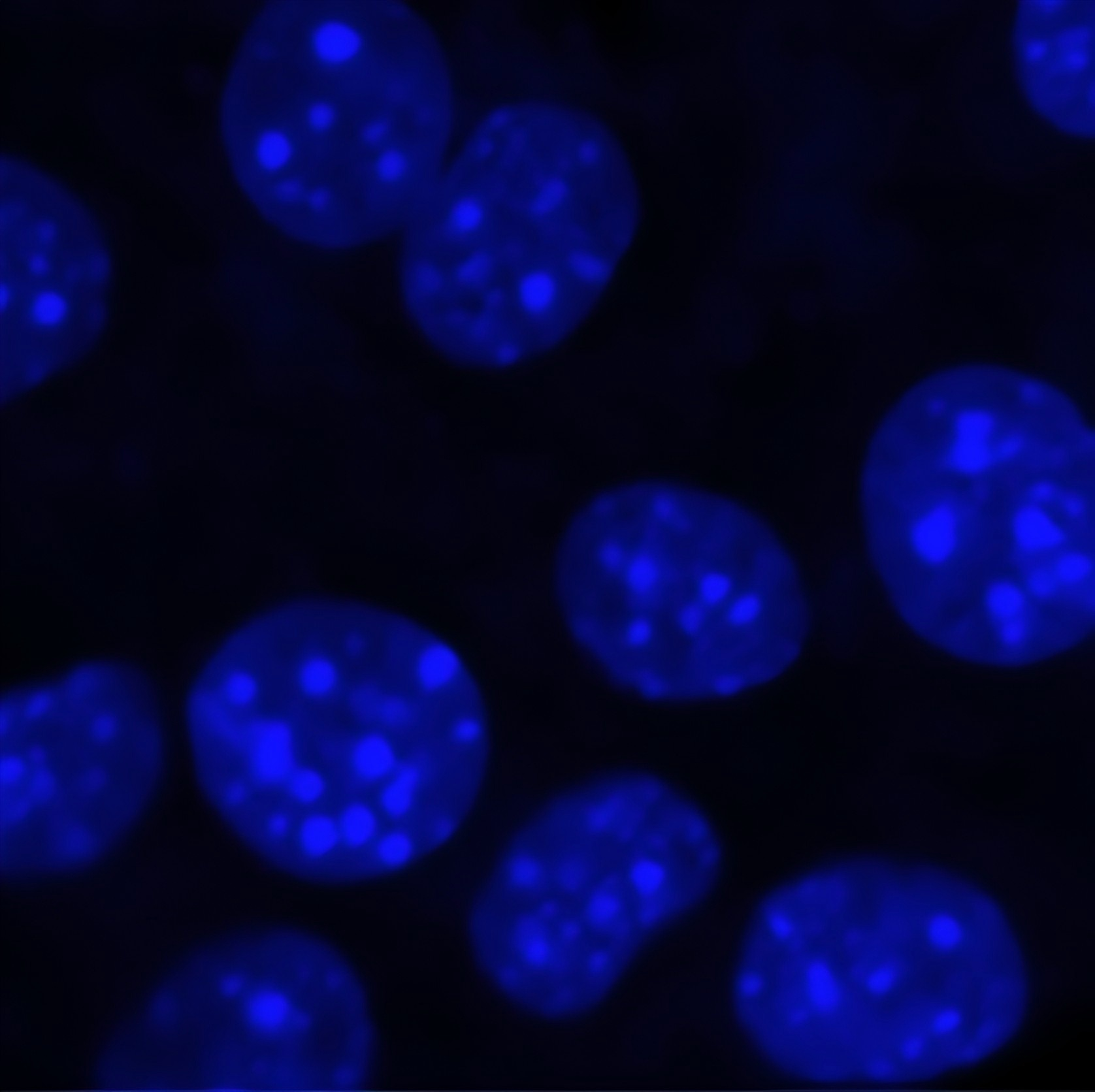

Supplement: Supplementary file 19 — Represent Raw Images [file 41419_2026_8682_MOESM19_ESM.zip › IFRAW/1-1-3.bmp]

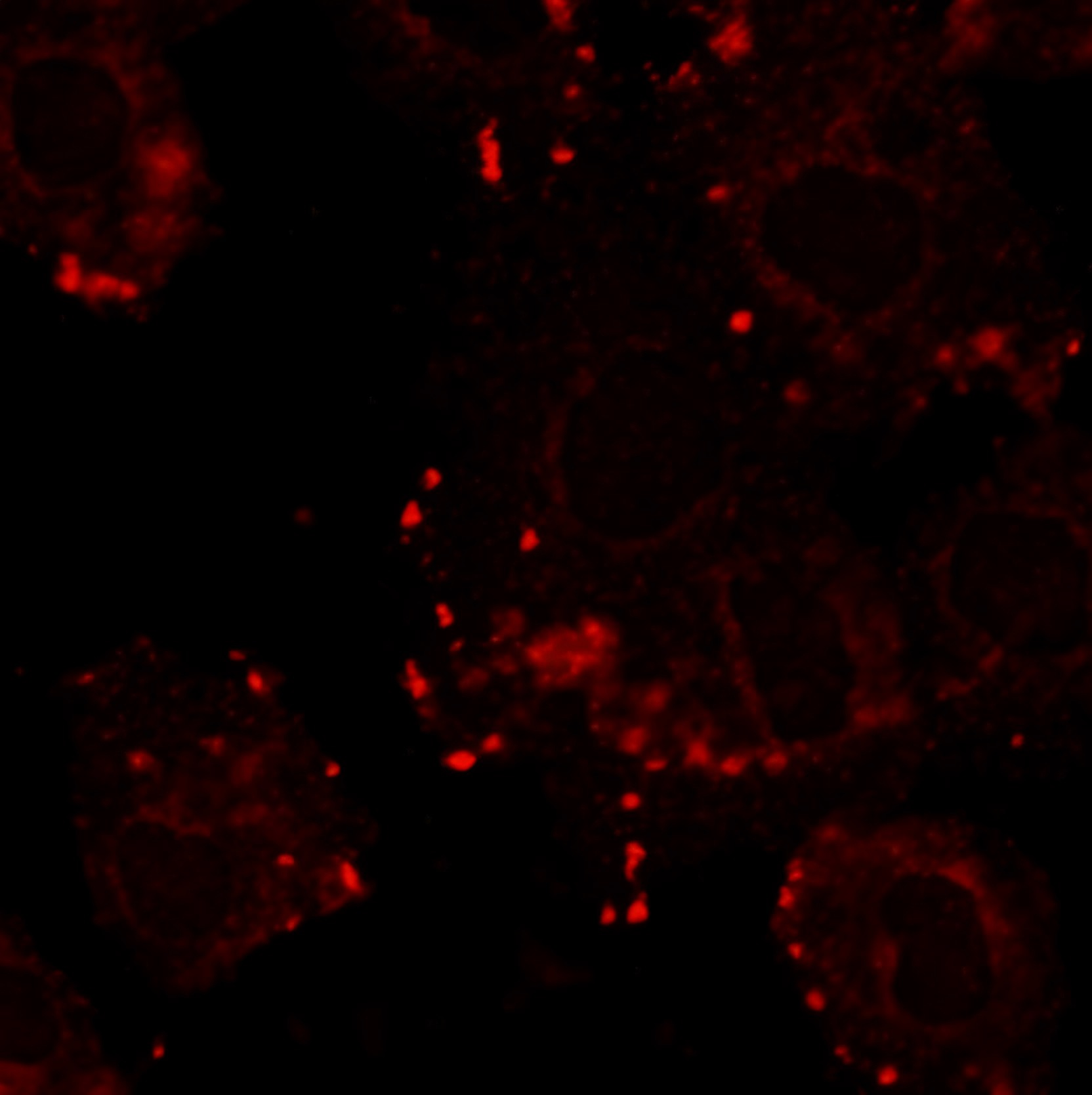

Supplement: Supplementary file 19 — Represent Raw Images [file 41419_2026_8682_MOESM19_ESM.zip › IFRAW/1-2-1.bmp]

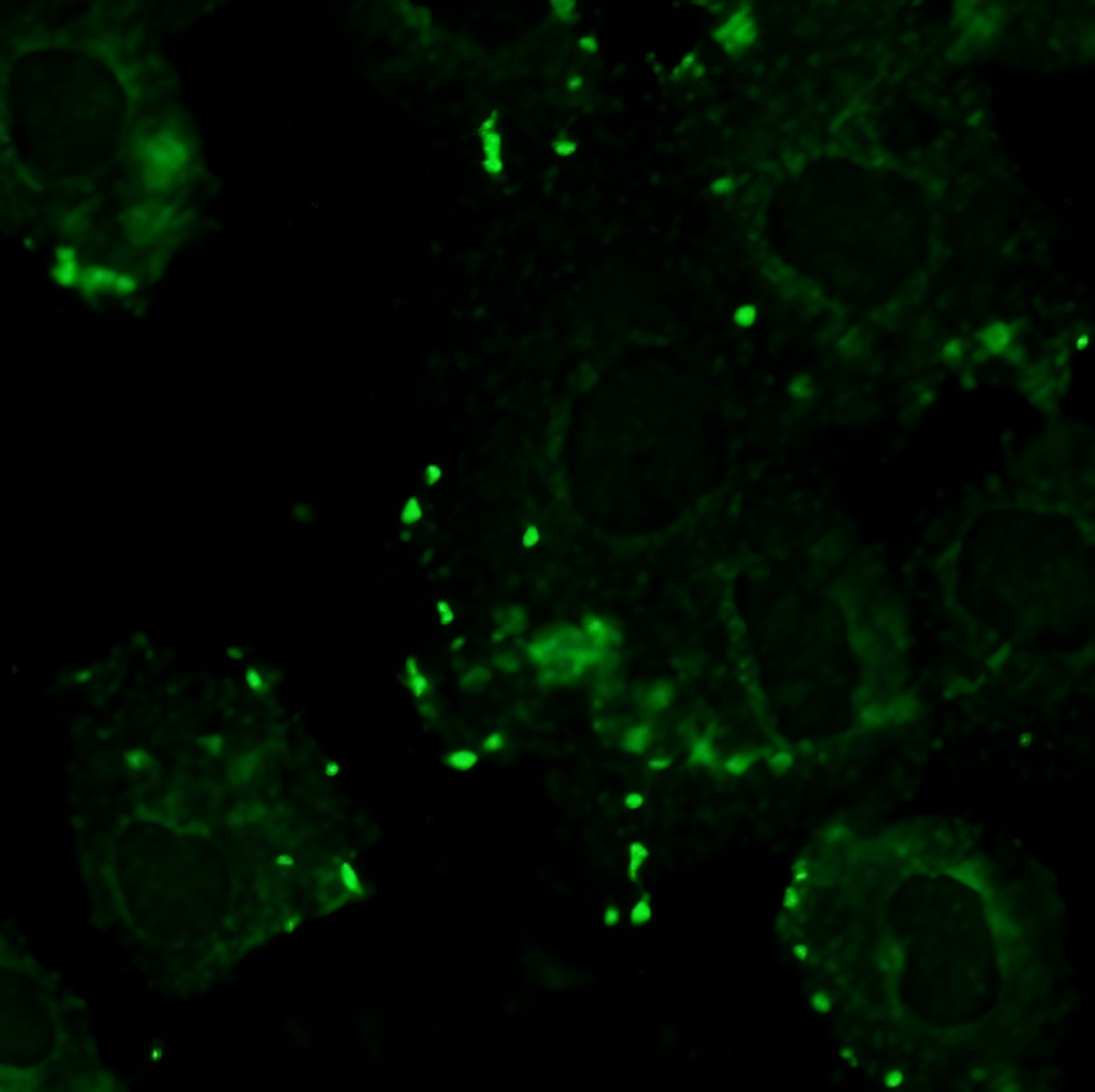

Supplement: Supplementary file 19 — Represent Raw Images [file 41419_2026_8682_MOESM19_ESM.zip › IFRAW/1-2-2.bmp]

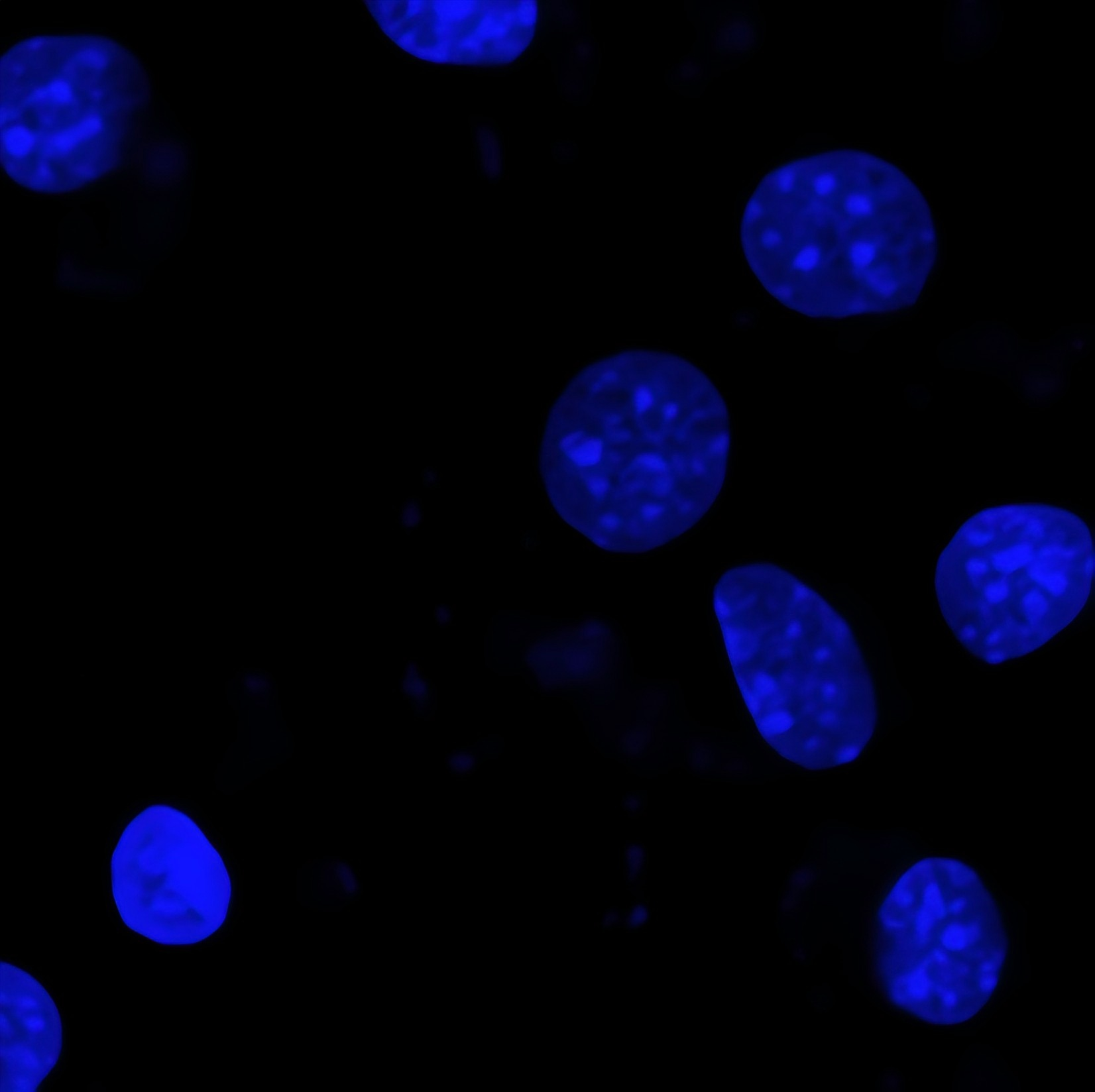

Supplement: Supplementary file 19 — Represent Raw Images [file 41419_2026_8682_MOESM19_ESM.zip › IFRAW/1-2-3.bmp]

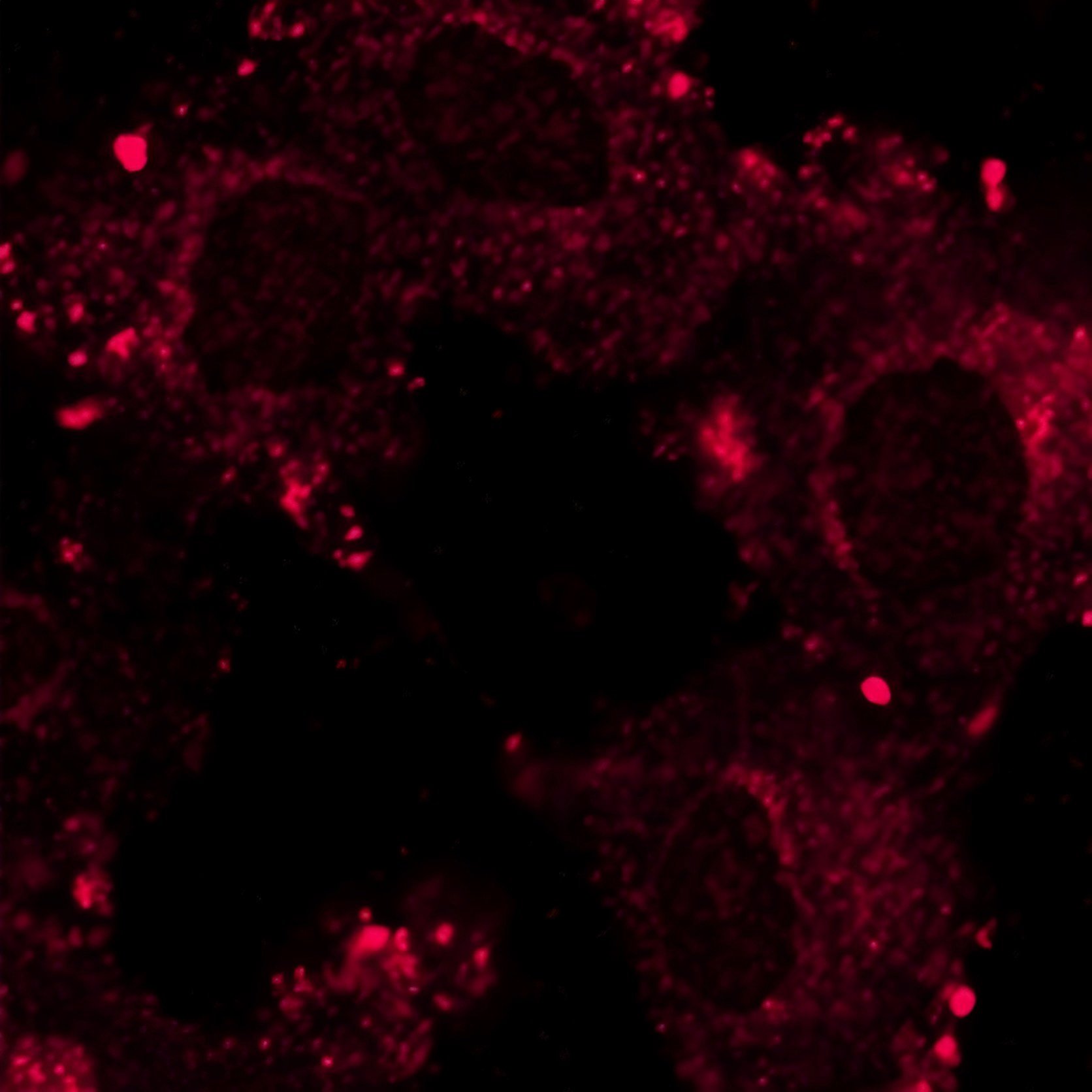

Supplement: Supplementary file 19 — Represent Raw Images [file 41419_2026_8682_MOESM19_ESM.zip › IFRAW/1-3-1.bmp]

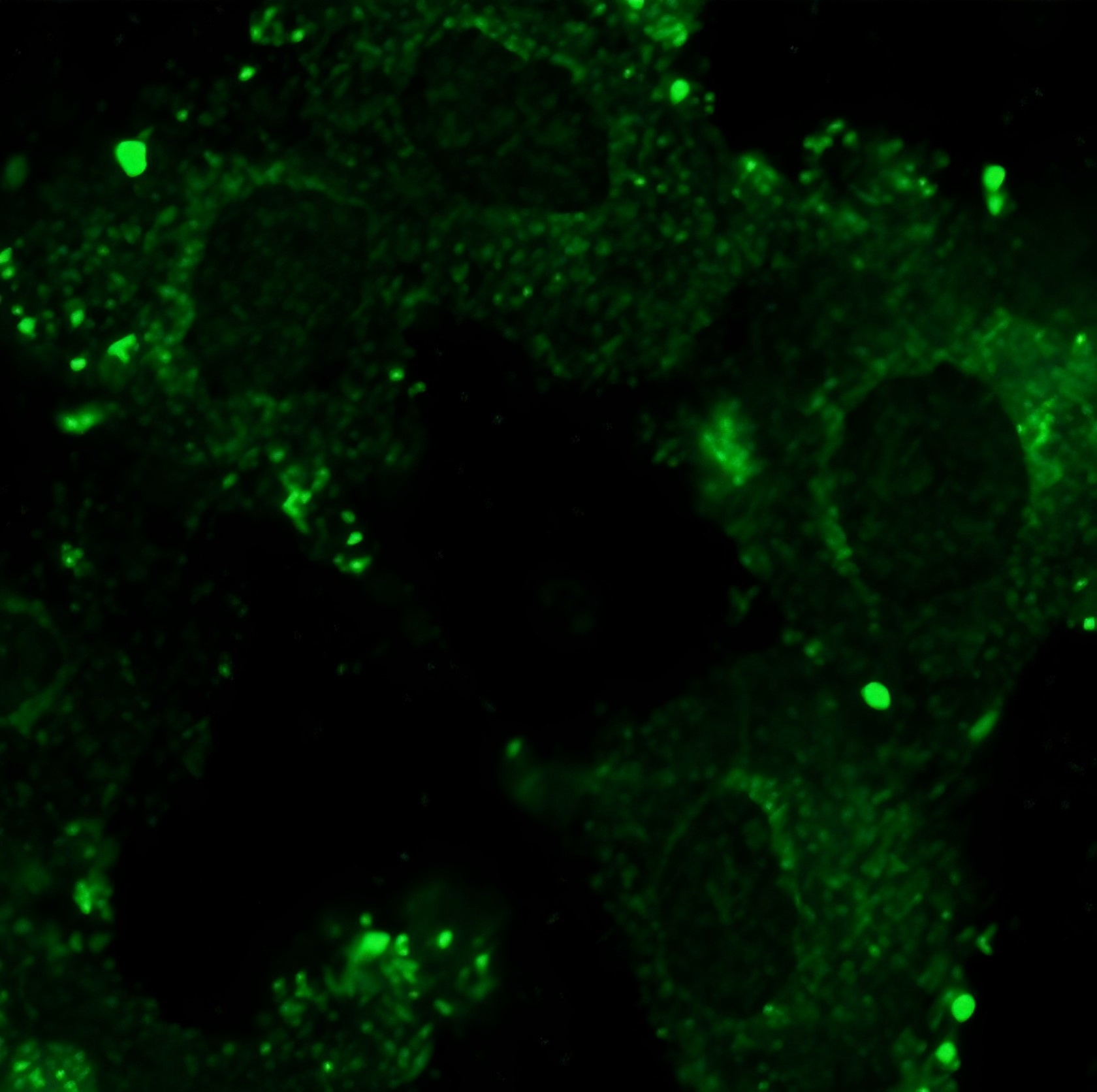

Supplement: Supplementary file 19 — Represent Raw Images [file 41419_2026_8682_MOESM19_ESM.zip › IFRAW/1-3-2.bmp]

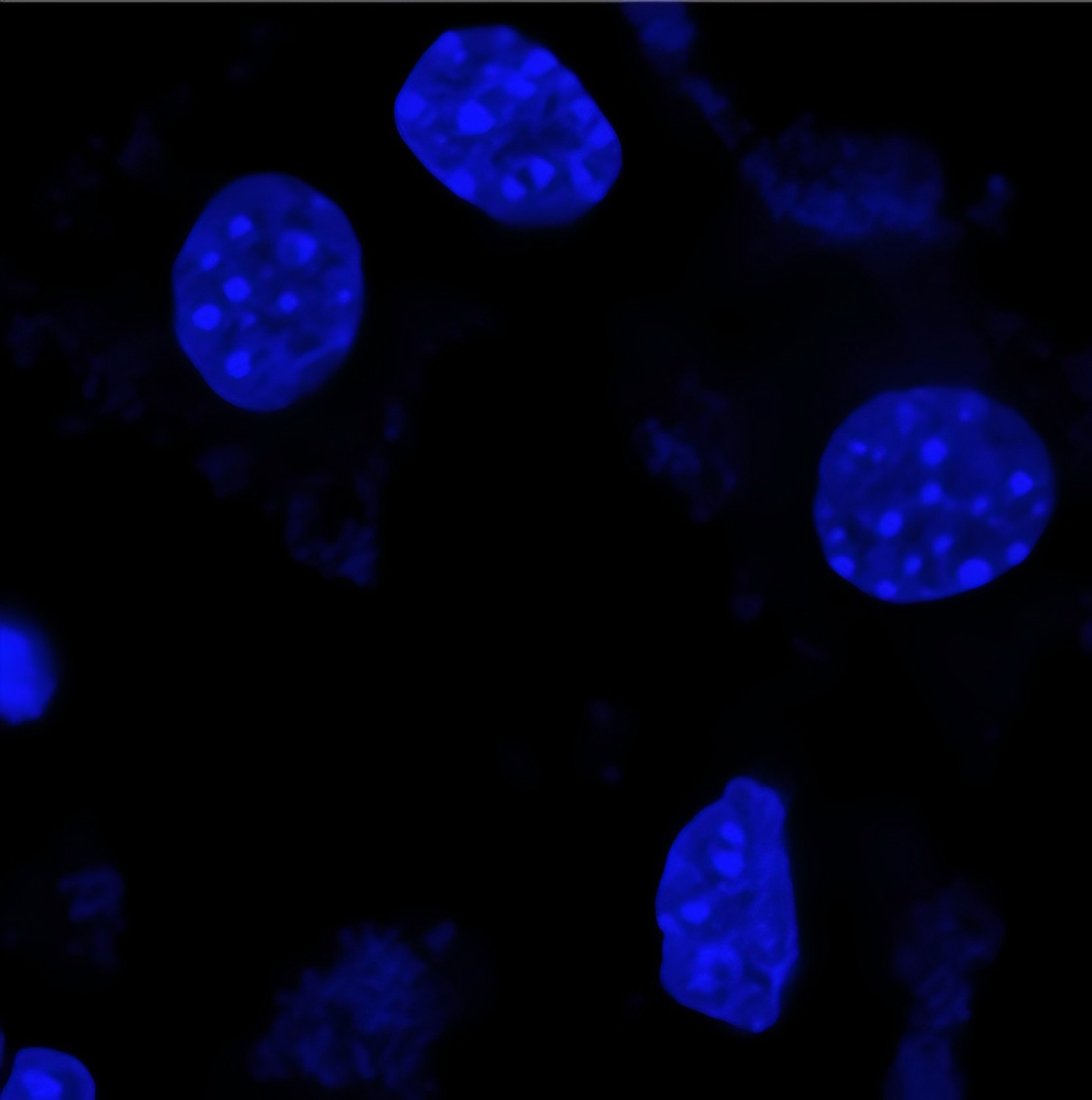

Supplement: Supplementary file 19 — Represent Raw Images [file 41419_2026_8682_MOESM19_ESM.zip › IFRAW/1-3-3.bmp]

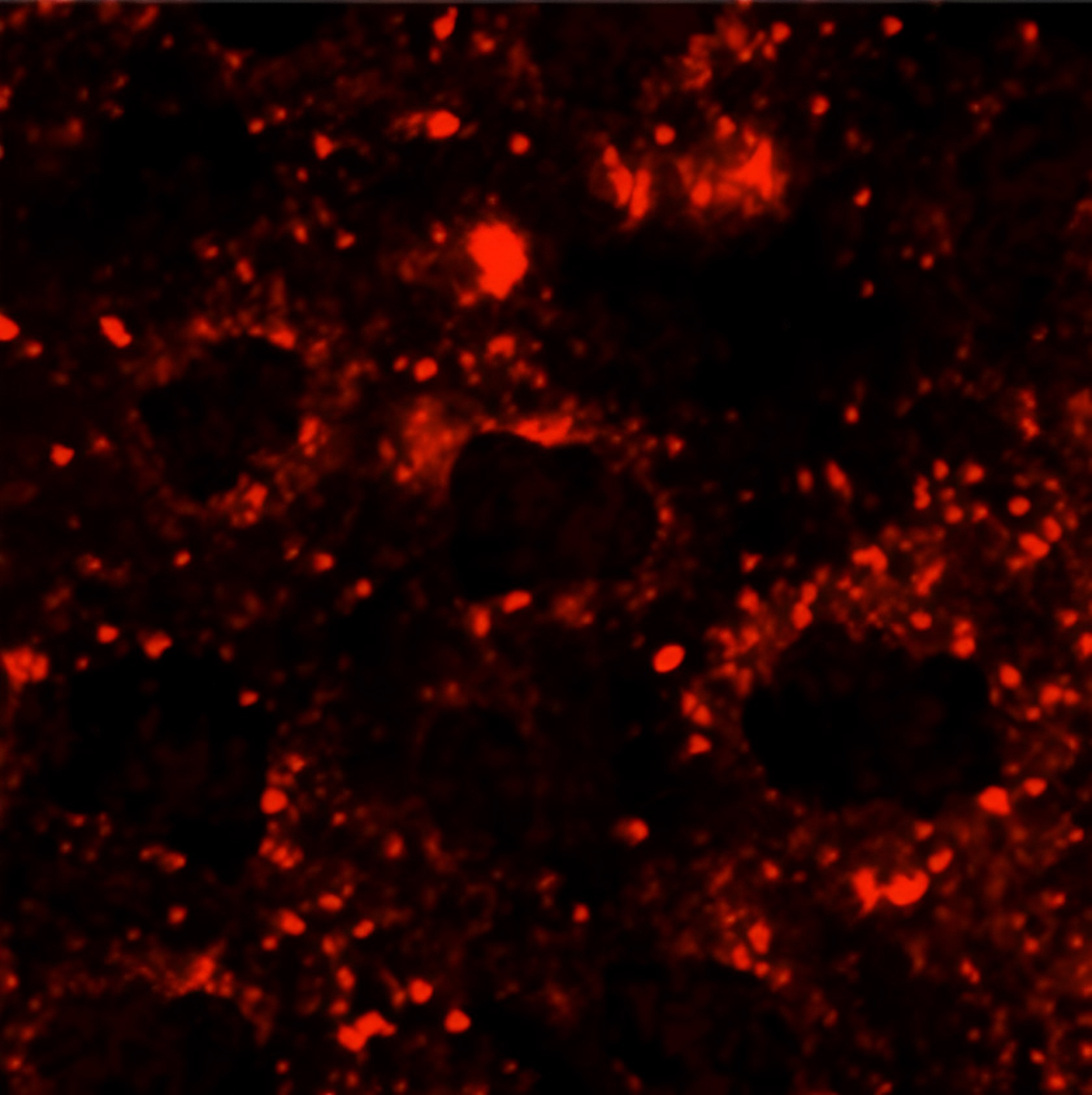

Supplement: Supplementary file 19 — Represent Raw Images [file 41419_2026_8682_MOESM19_ESM.zip › IFRAW/1-4-1.bmp]

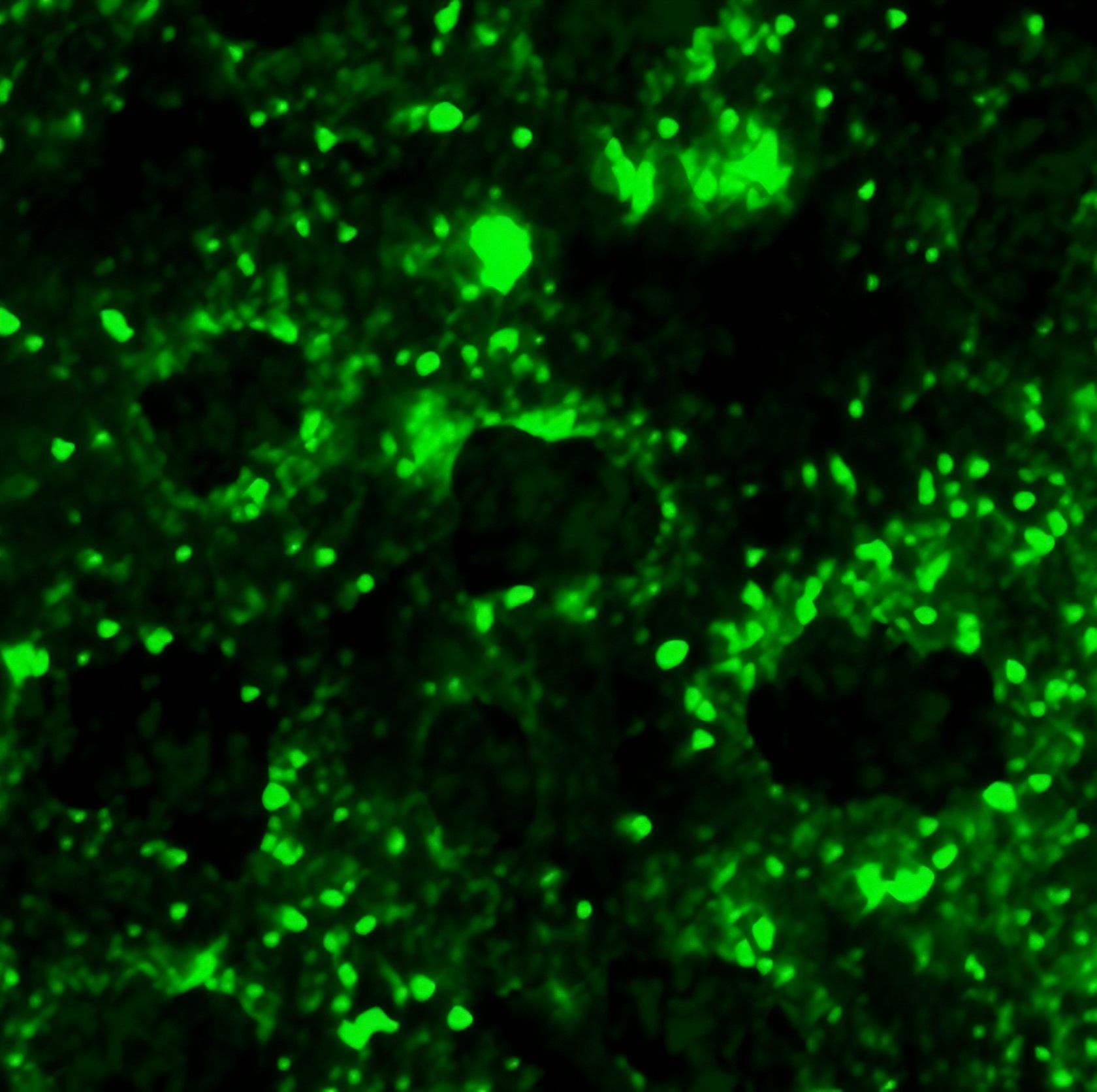

Supplement: Supplementary file 19 — Represent Raw Images [file 41419_2026_8682_MOESM19_ESM.zip › IFRAW/1-4-2.bmp]

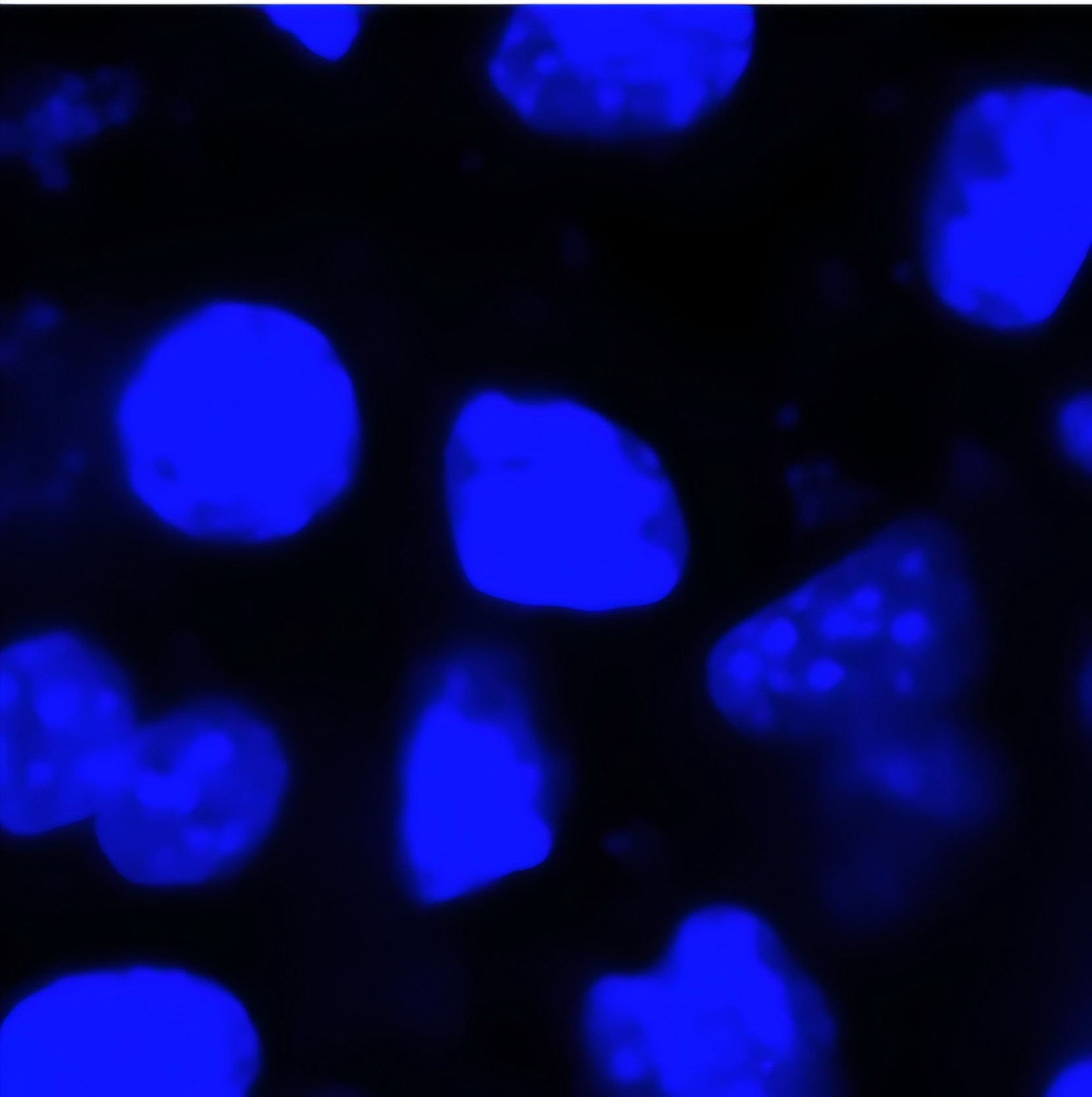

Supplement: Supplementary file 19 — Represent Raw Images [file 41419_2026_8682_MOESM19_ESM.zip › IFRAW/1-4-3.bmp]

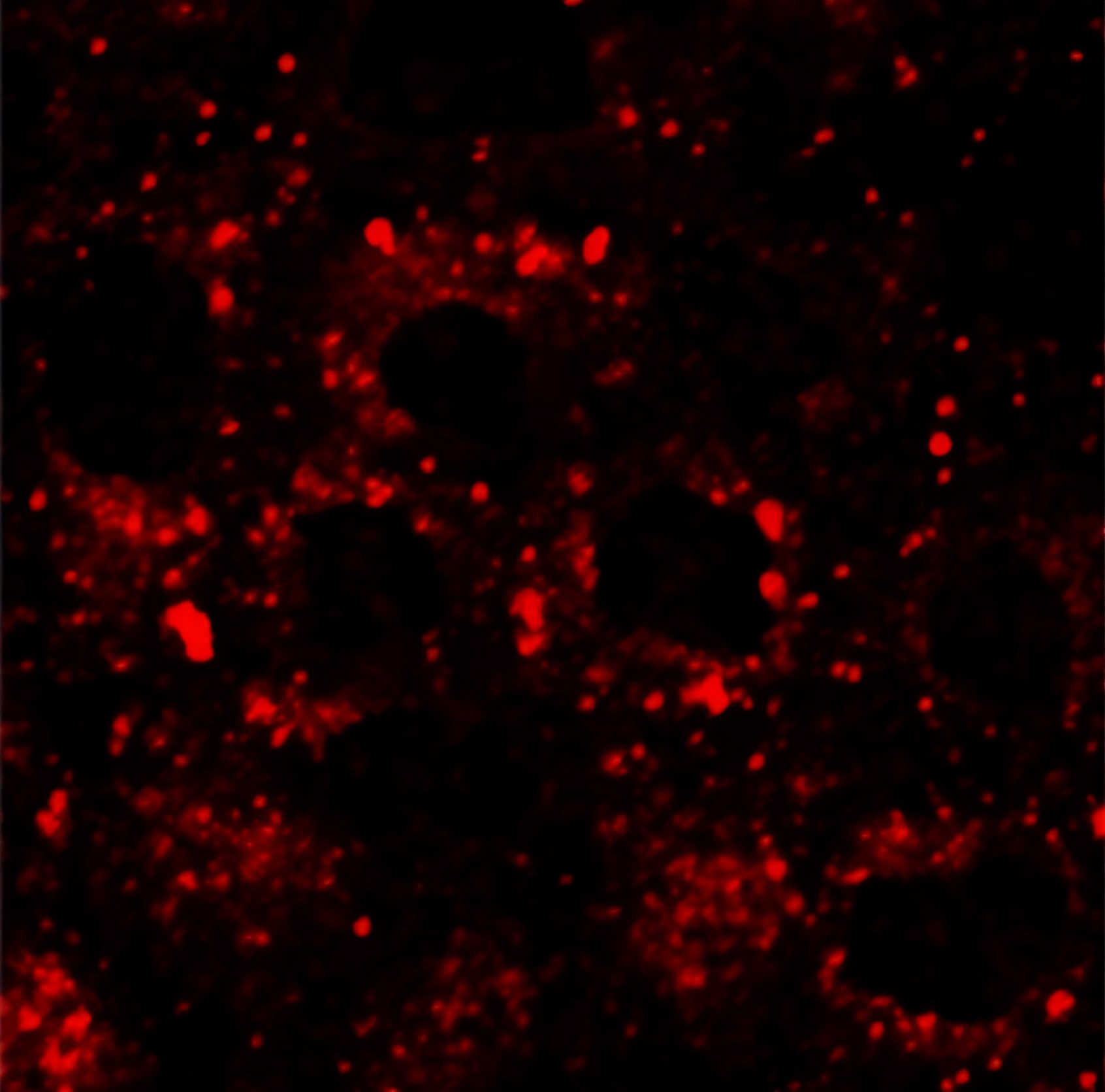

Supplement: Supplementary file 19 — Represent Raw Images [file 41419_2026_8682_MOESM19_ESM.zip › IFRAW/1-5-1.bmp]

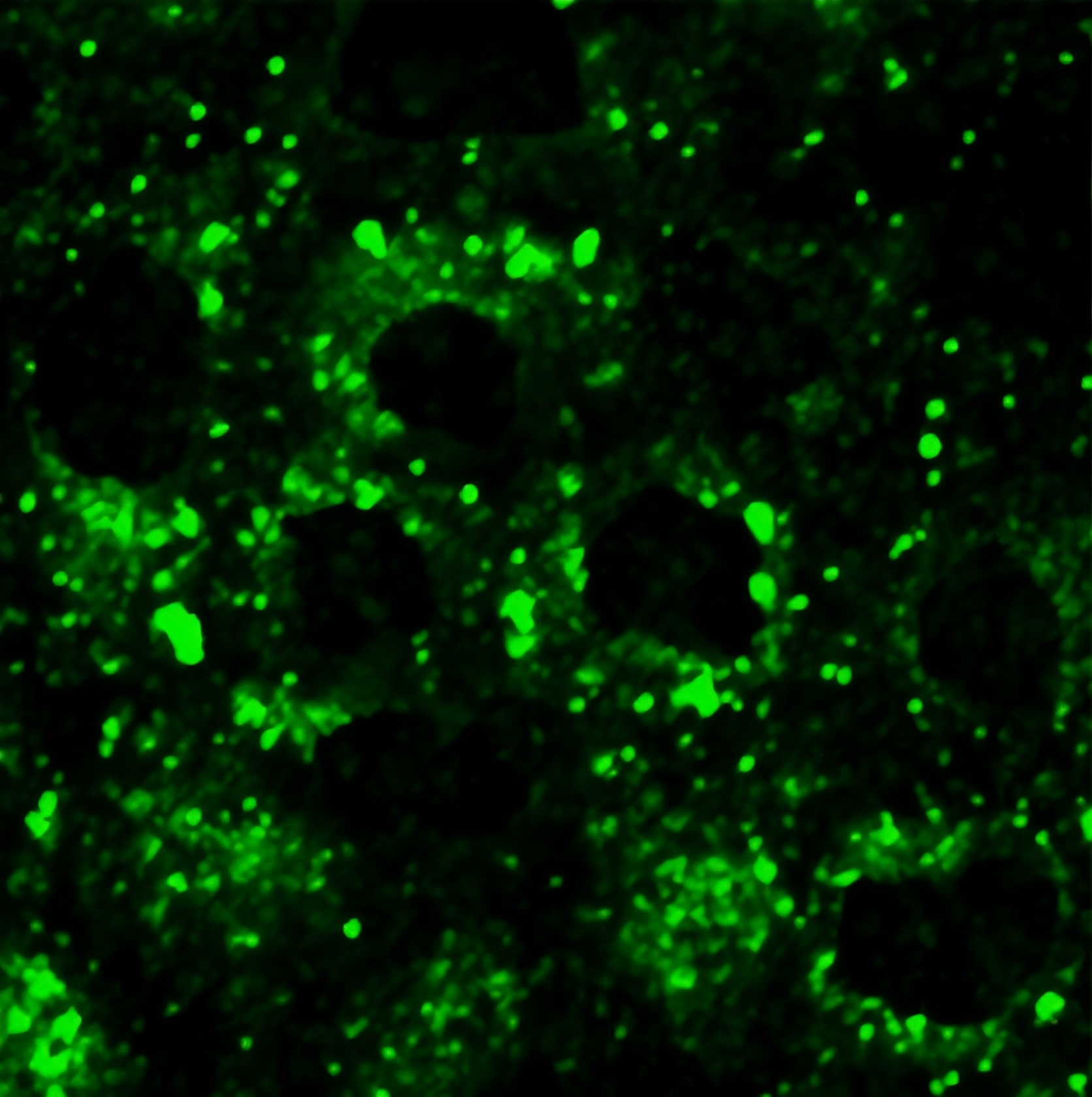

Supplement: Supplementary file 19 — Represent Raw Images [file 41419_2026_8682_MOESM19_ESM.zip › IFRAW/1-5-2.bmp]

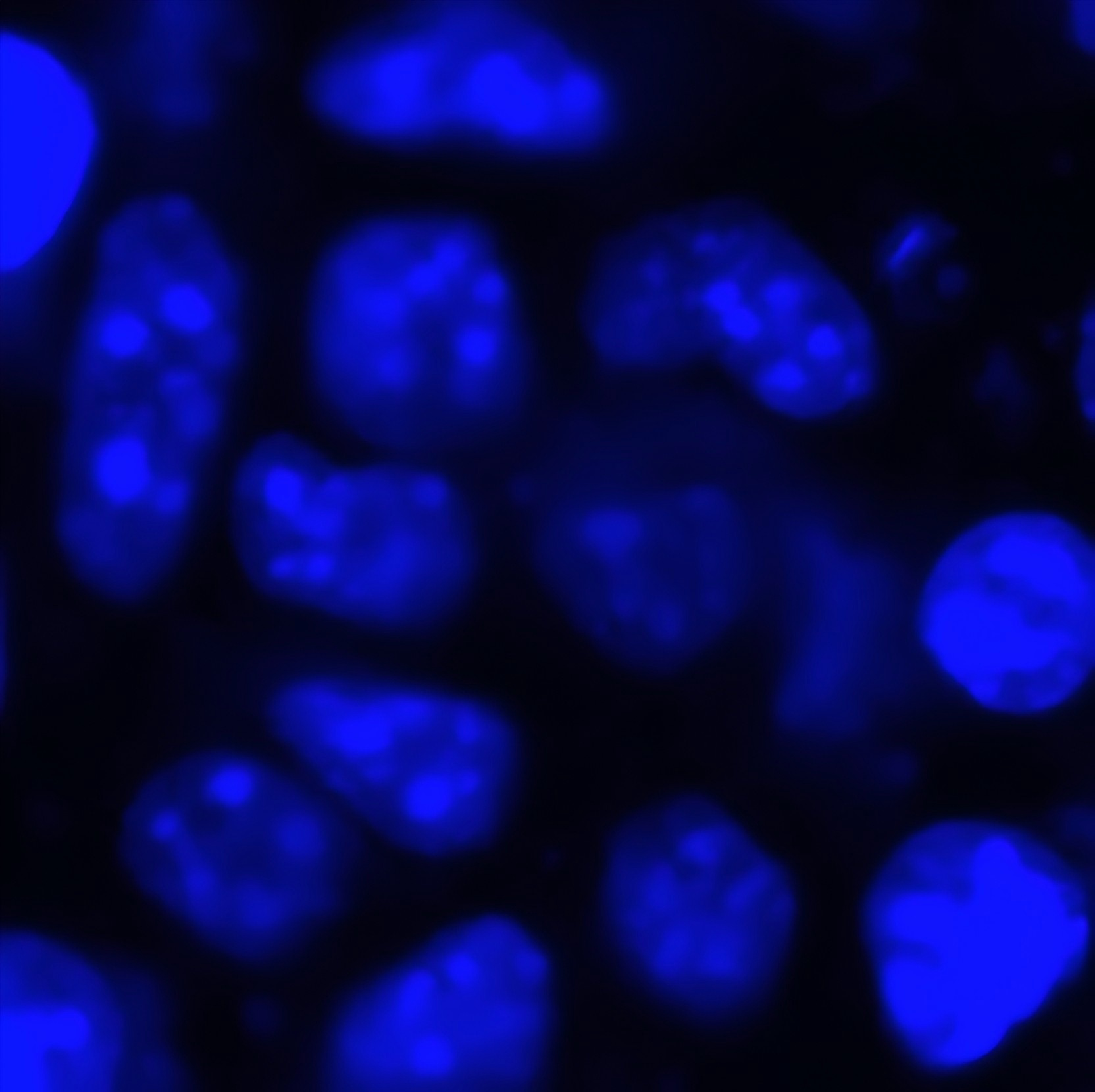

Supplement: Supplementary file 19 — Represent Raw Images [file 41419_2026_8682_MOESM19_ESM.zip › IFRAW/1-5-3.bmp]

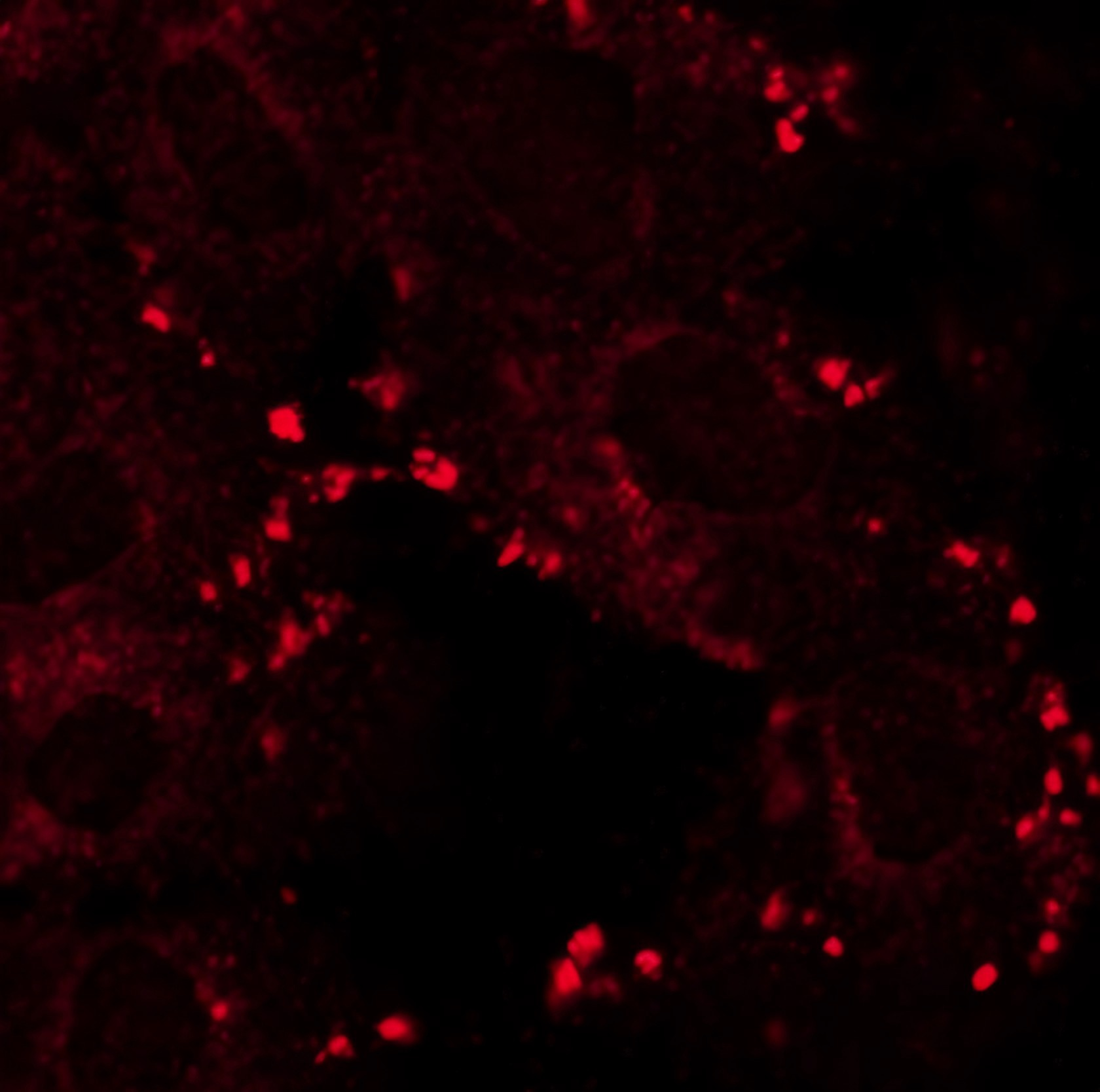

Supplement: Supplementary file 19 — Represent Raw Images [file 41419_2026_8682_MOESM19_ESM.zip › IFRAW/1-6-1.bmp]

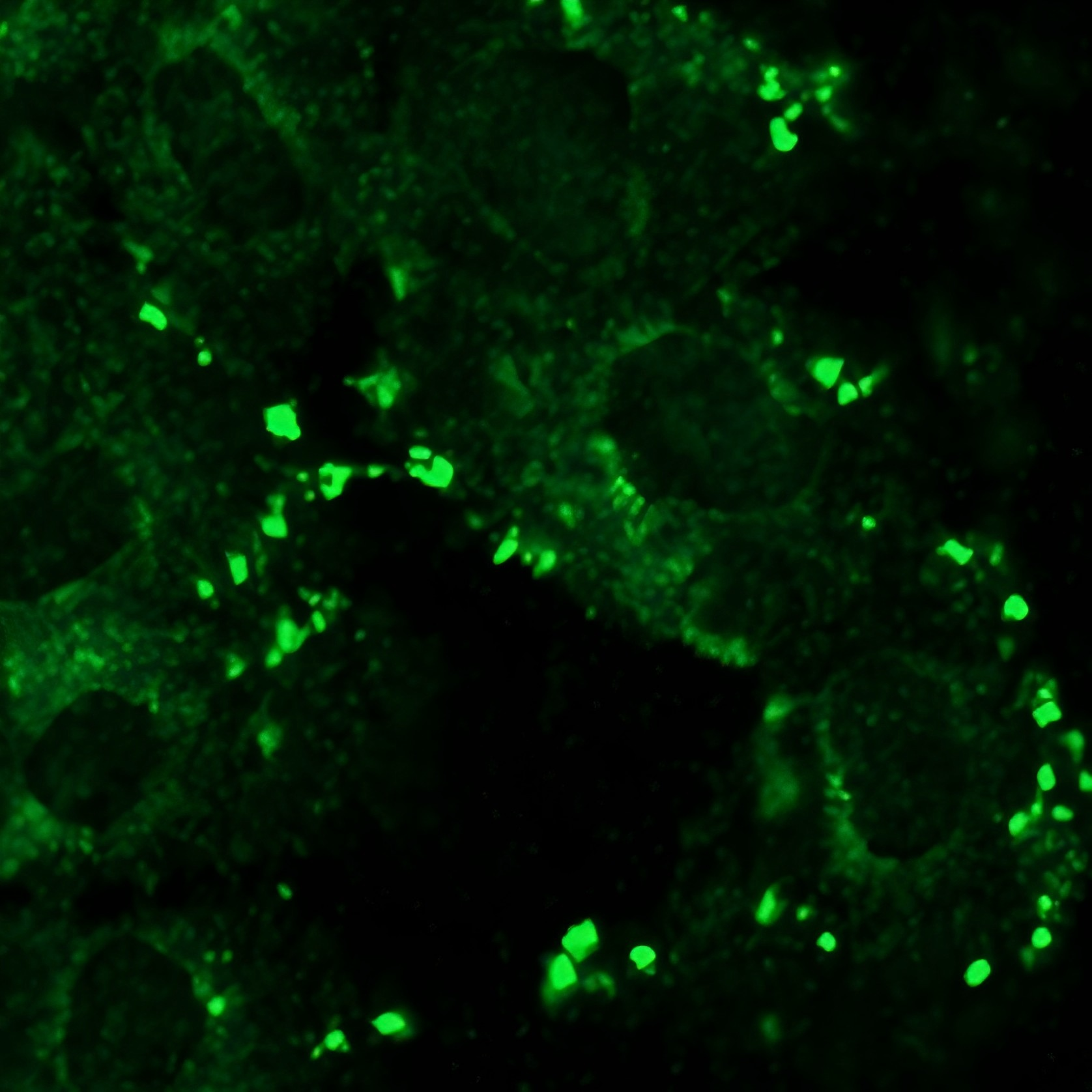

Supplement: Supplementary file 19 — Represent Raw Images [file 41419_2026_8682_MOESM19_ESM.zip › IFRAW/1-6-2.bmp]

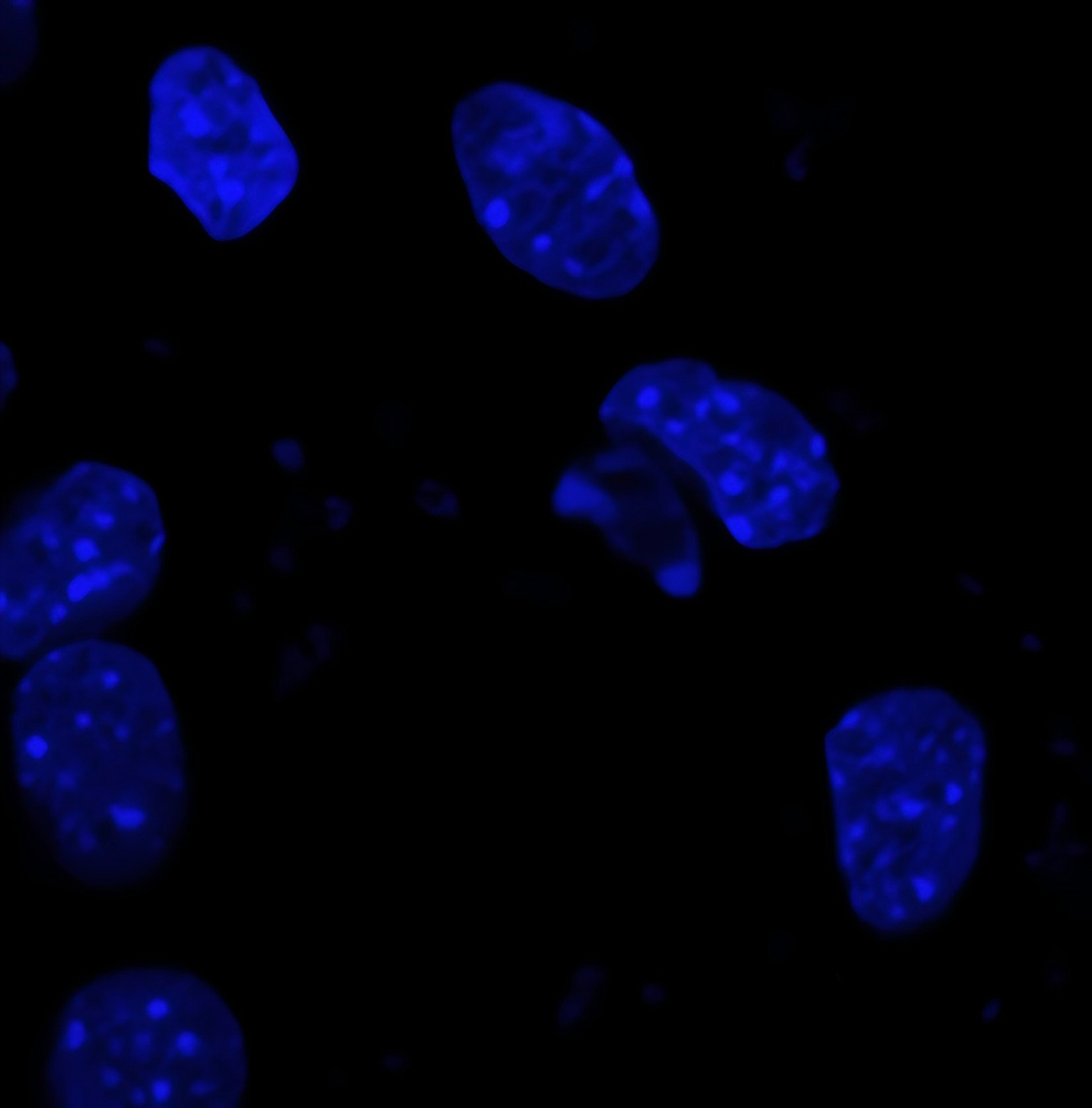

Supplement: Supplementary file 19 — Represent Raw Images [file 41419_2026_8682_MOESM19_ESM.zip › IFRAW/1-6-3.bmp]

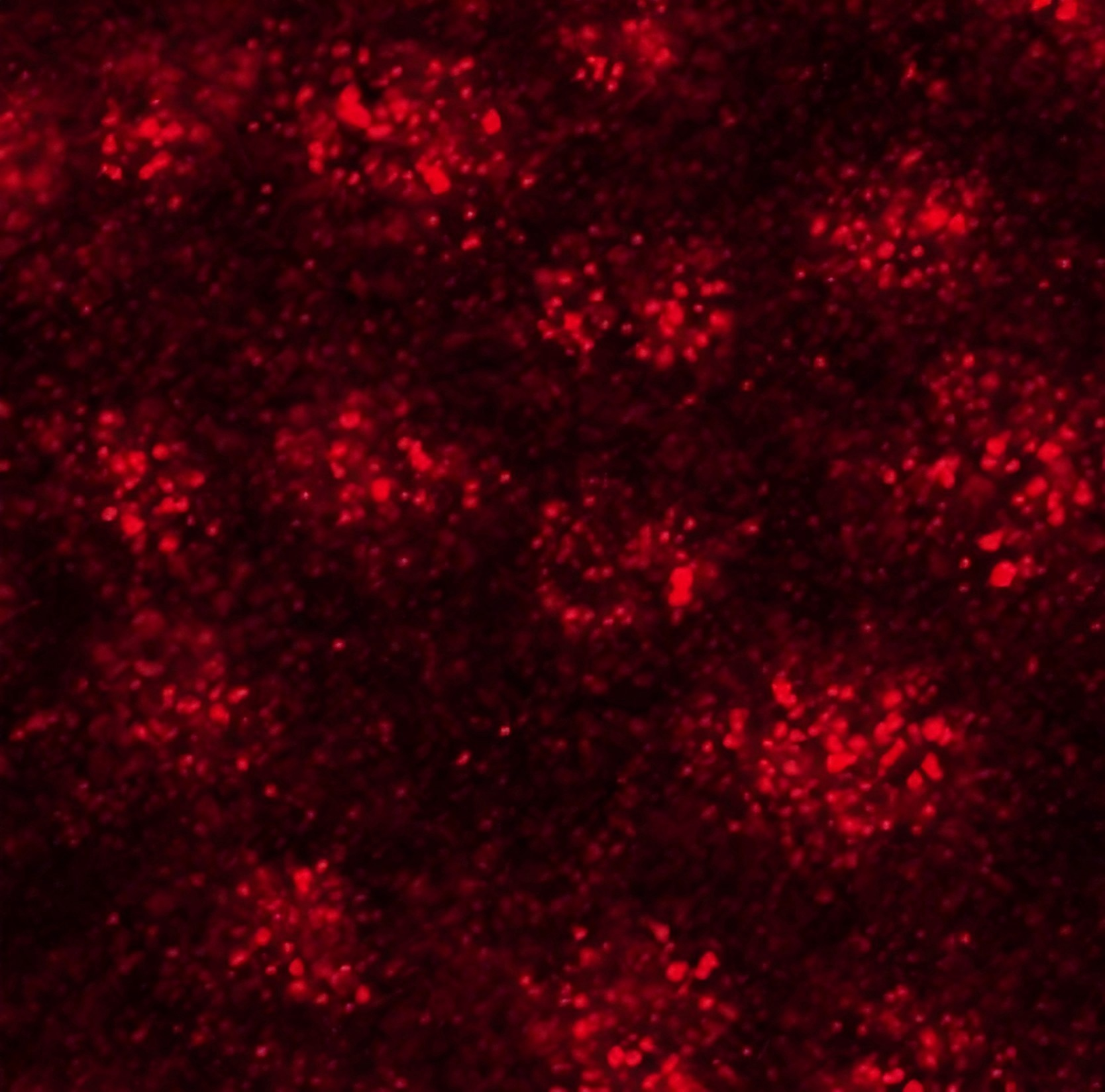

Supplement: Supplementary file 19 — Represent Raw Images [file 41419_2026_8682_MOESM19_ESM.zip › IFRAW/2-1-1.bmp]

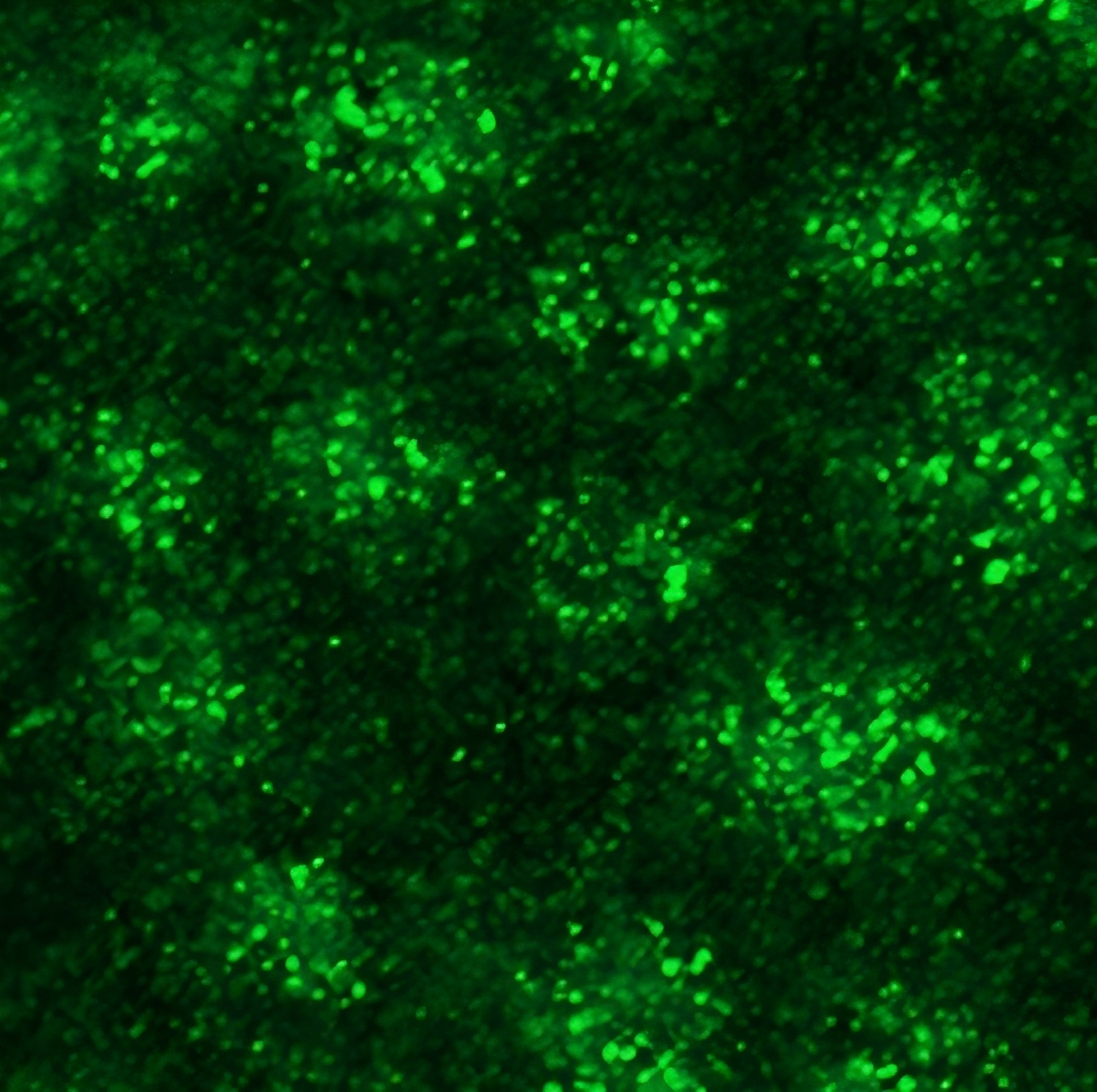

Supplement: Supplementary file 19 — Represent Raw Images [file 41419_2026_8682_MOESM19_ESM.zip › IFRAW/2-1-2.bmp]

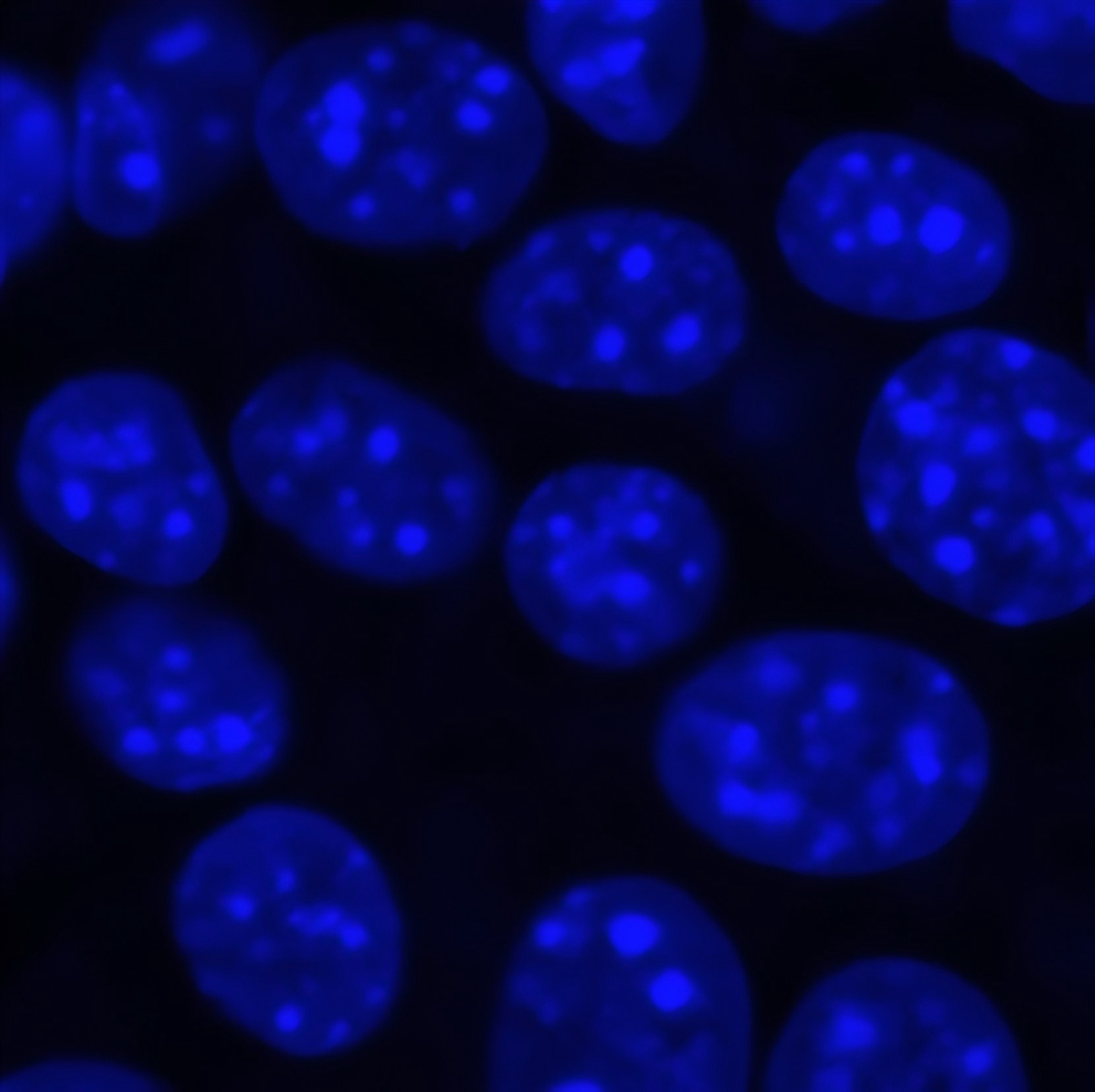

Supplement: Supplementary file 19 — Represent Raw Images [file 41419_2026_8682_MOESM19_ESM.zip › IFRAW/2-1-3.bmp]

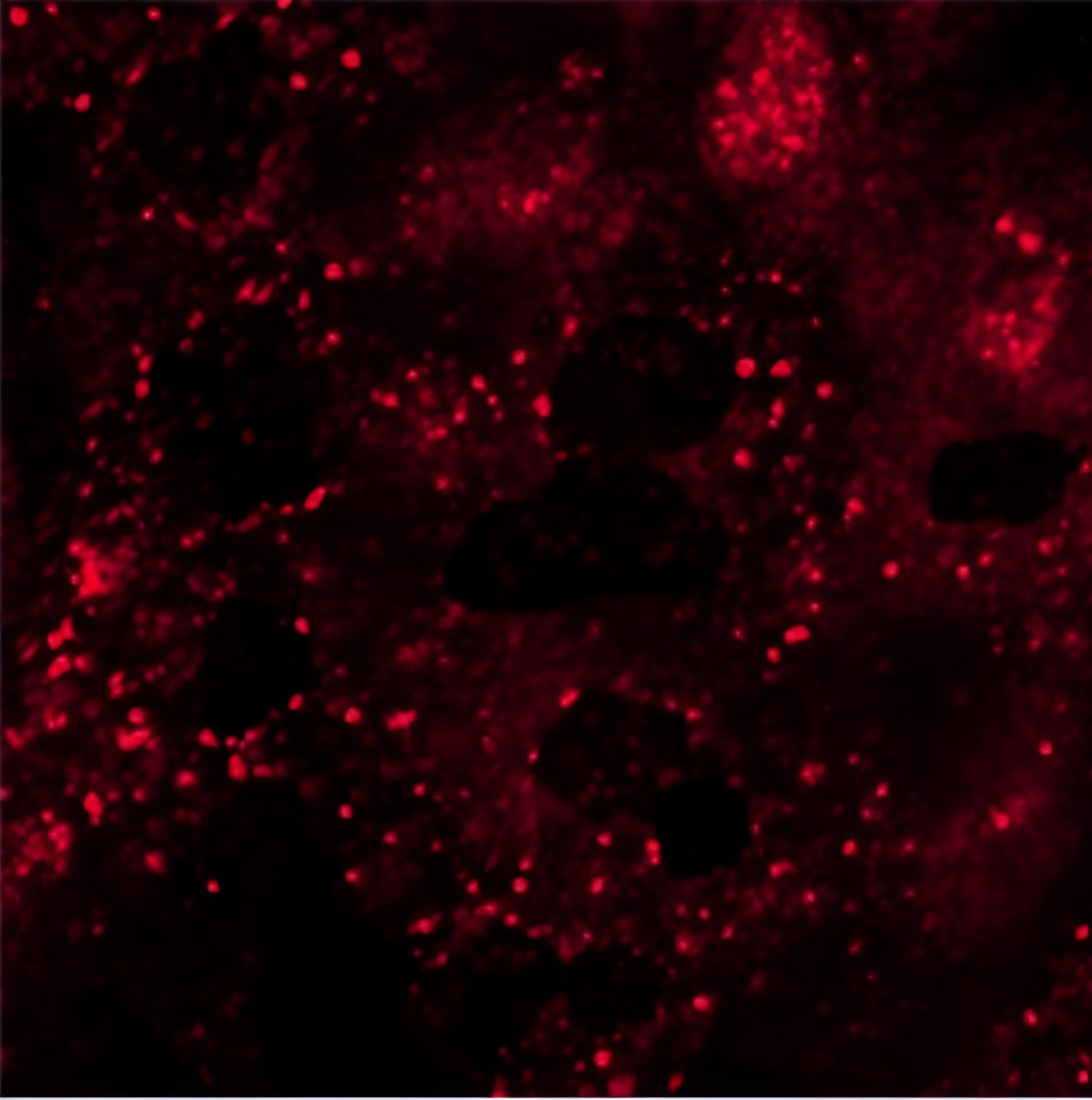

Supplement: Supplementary file 19 — Represent Raw Images [file 41419_2026_8682_MOESM19_ESM.zip › IFRAW/2-2-1.bmp]

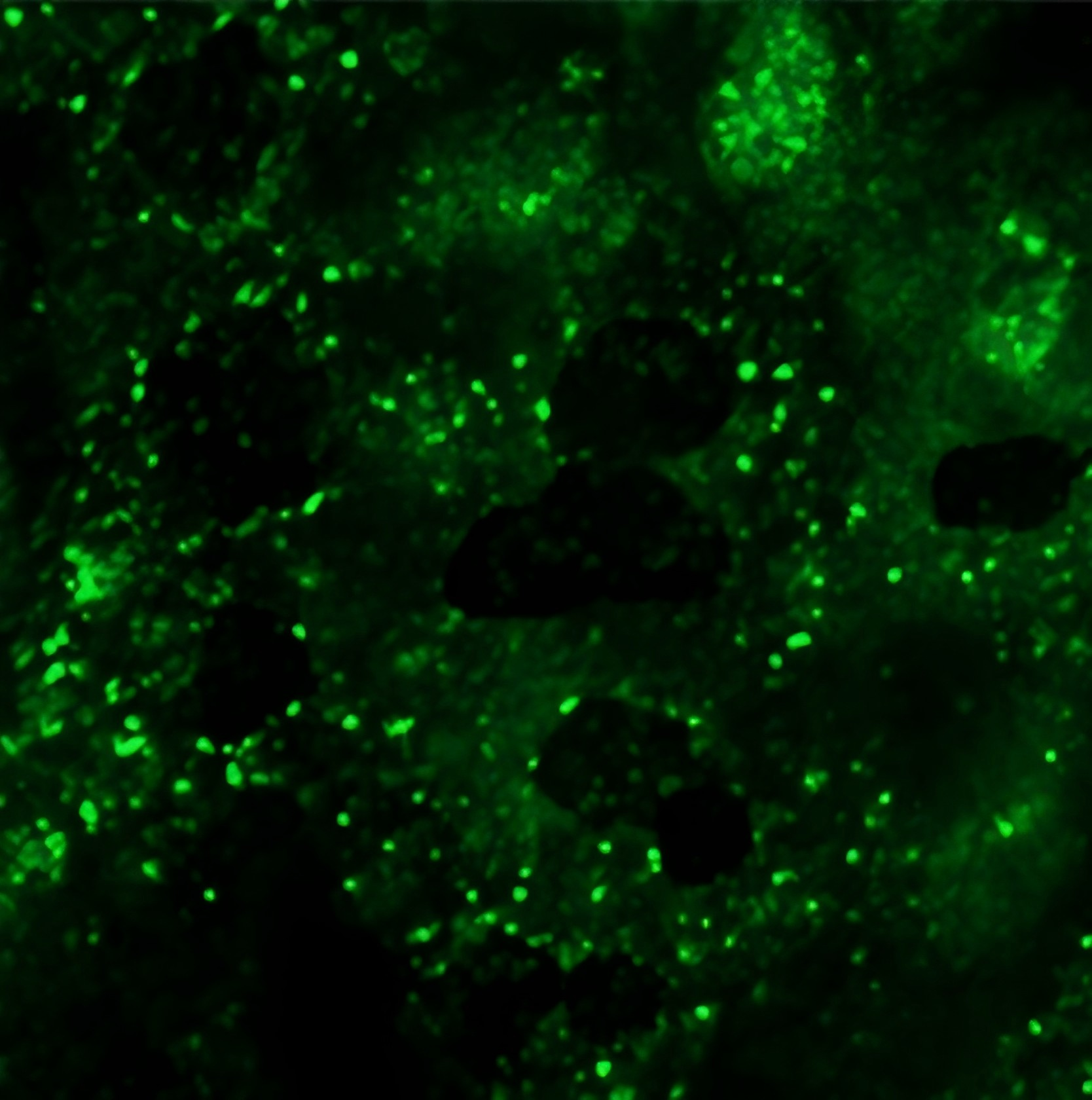

Supplement: Supplementary file 19 — Represent Raw Images [file 41419_2026_8682_MOESM19_ESM.zip › IFRAW/2-2-2.bmp]

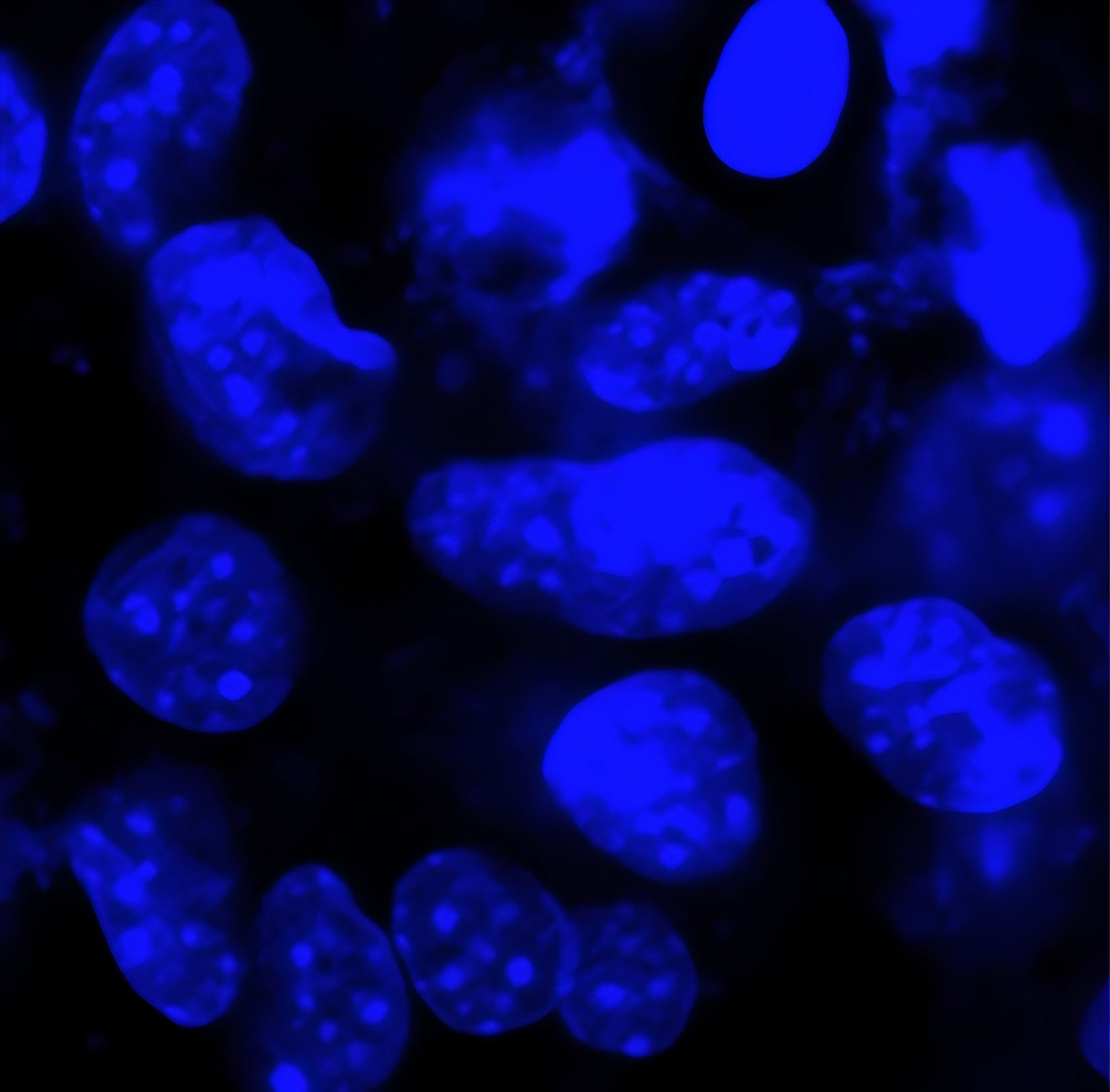

Supplement: Supplementary file 19 — Represent Raw Images [file 41419_2026_8682_MOESM19_ESM.zip › IFRAW/2-2-3.bmp]

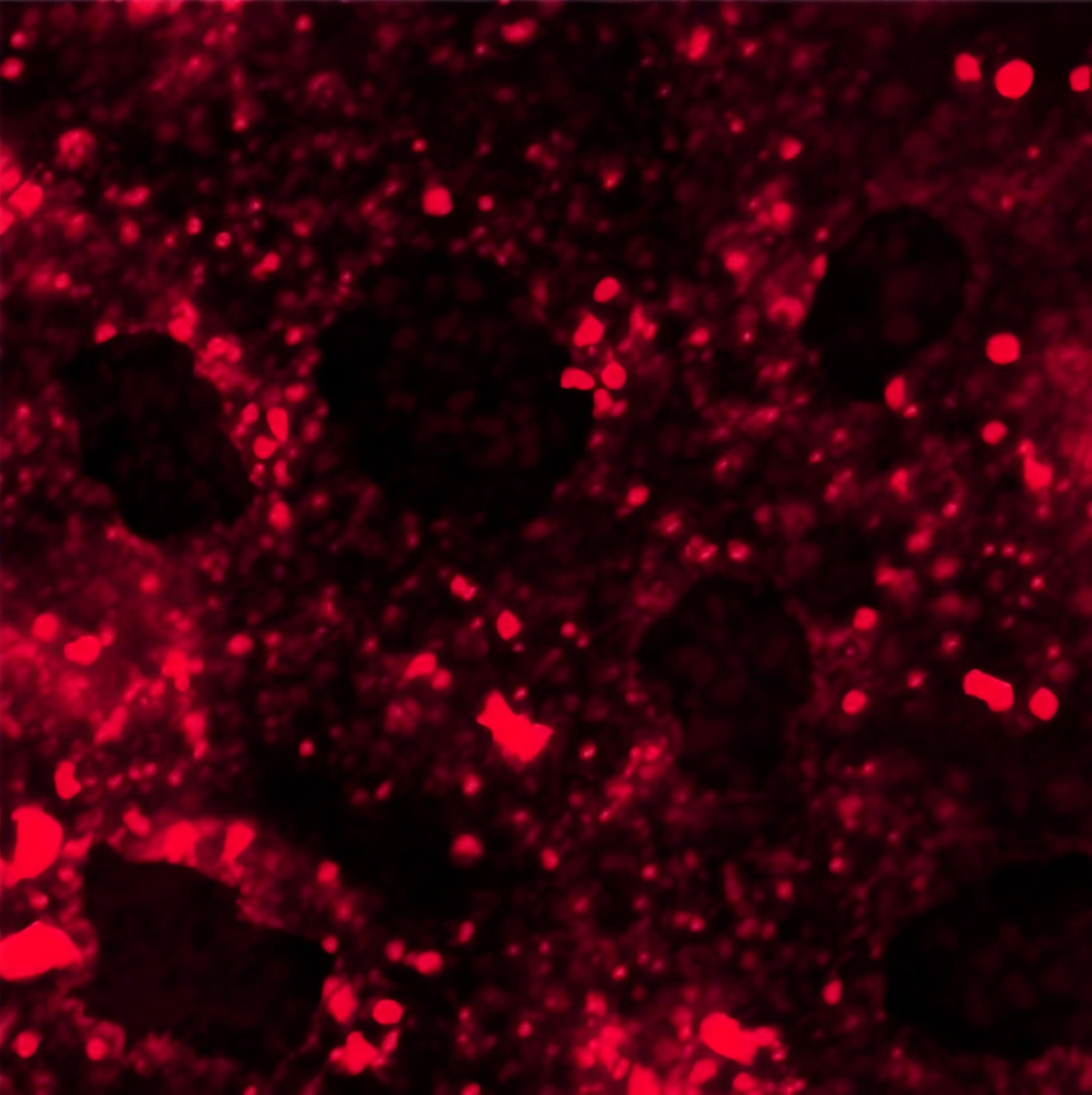

Supplement: Supplementary file 19 — Represent Raw Images [file 41419_2026_8682_MOESM19_ESM.zip › IFRAW/2-3-1.bmp]

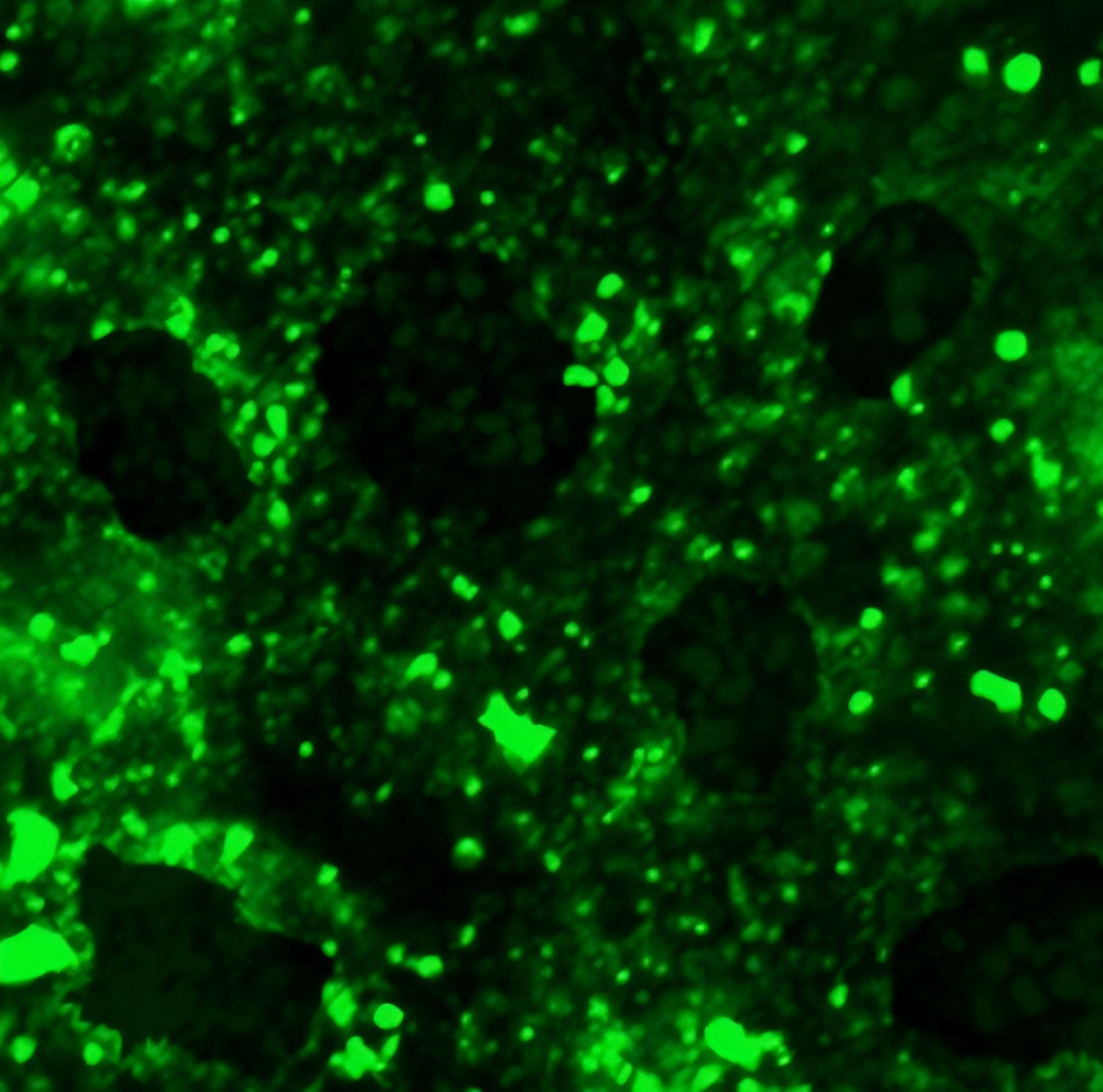

Supplement: Supplementary file 19 — Represent Raw Images [file 41419_2026_8682_MOESM19_ESM.zip › IFRAW/2-3-2.bmp]

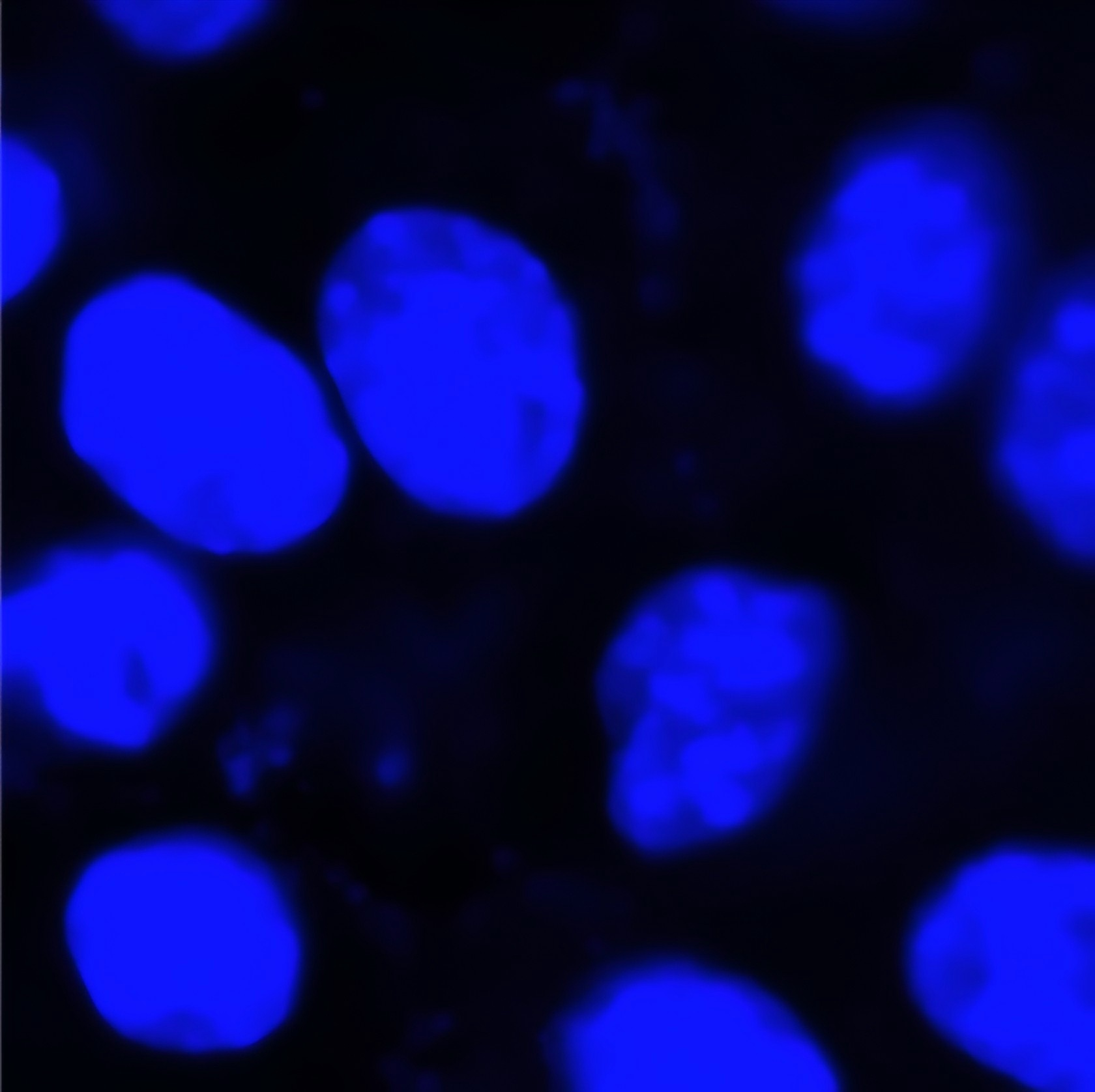

Supplement: Supplementary file 19 — Represent Raw Images [file 41419_2026_8682_MOESM19_ESM.zip › IFRAW/2-3-3.bmp]

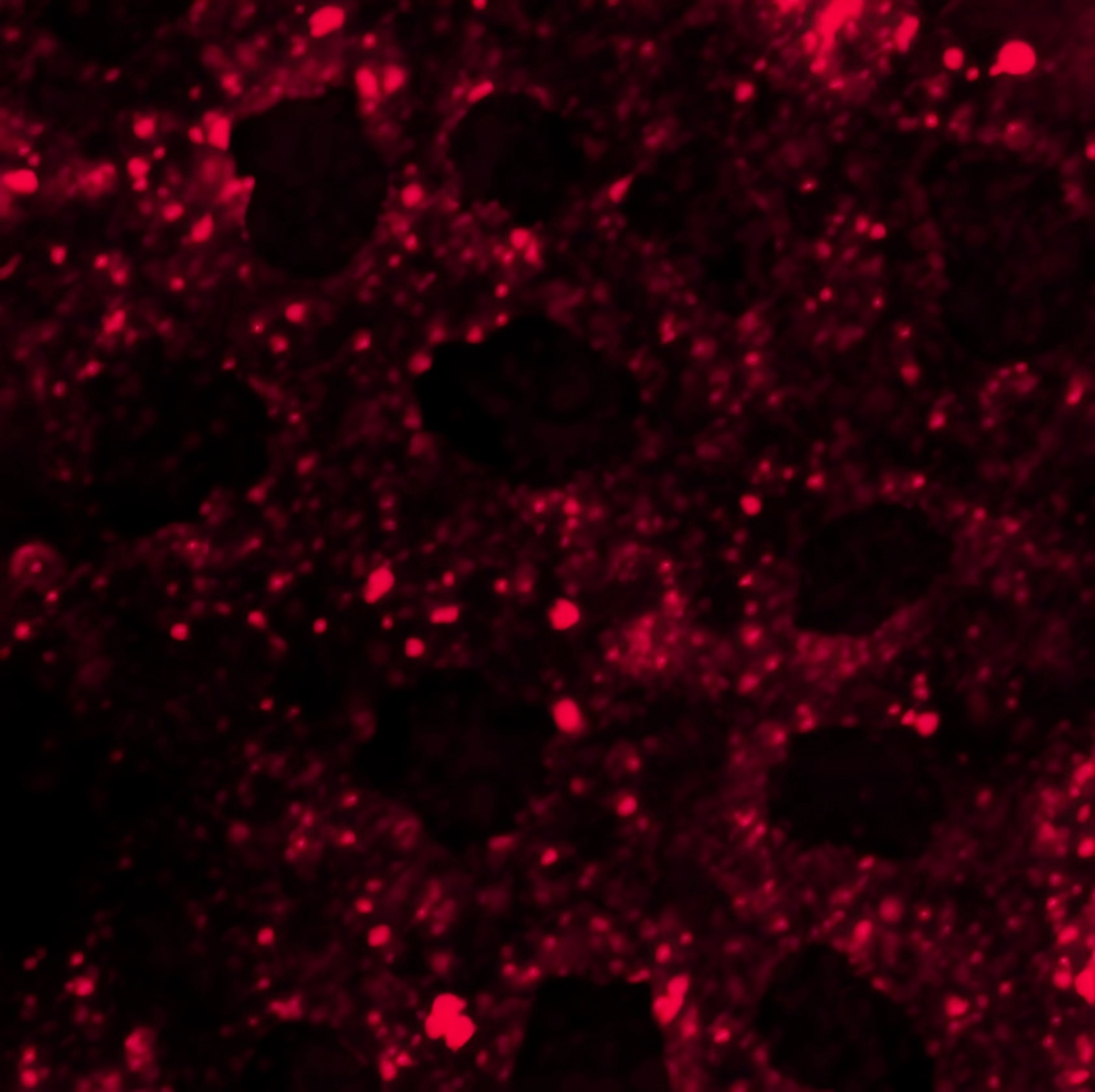

Supplement: Supplementary file 19 — Represent Raw Images [file 41419_2026_8682_MOESM19_ESM.zip › IFRAW/2-4-1.bmp]

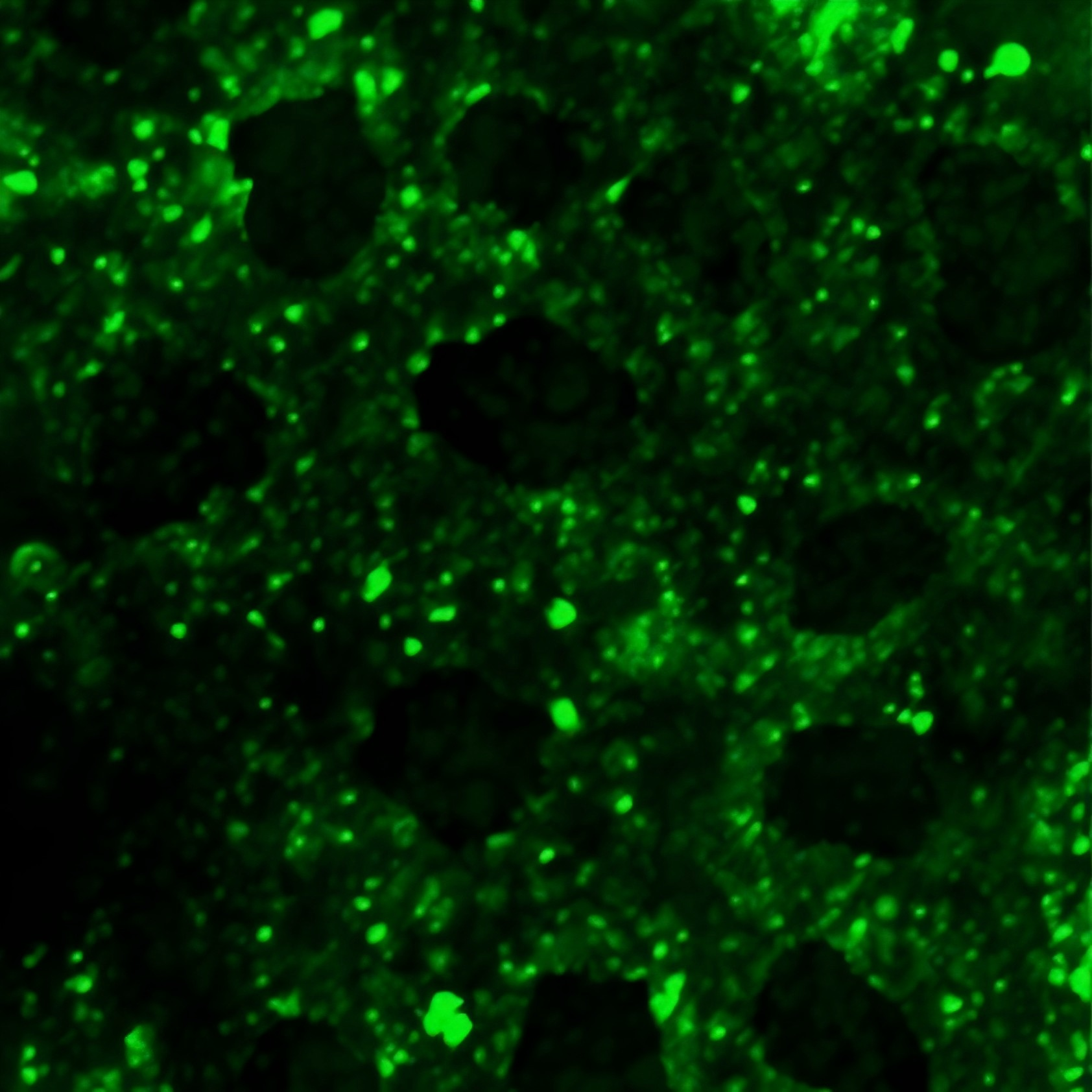

Supplement: Supplementary file 19 — Represent Raw Images [file 41419_2026_8682_MOESM19_ESM.zip › IFRAW/2-4-2.bmp]

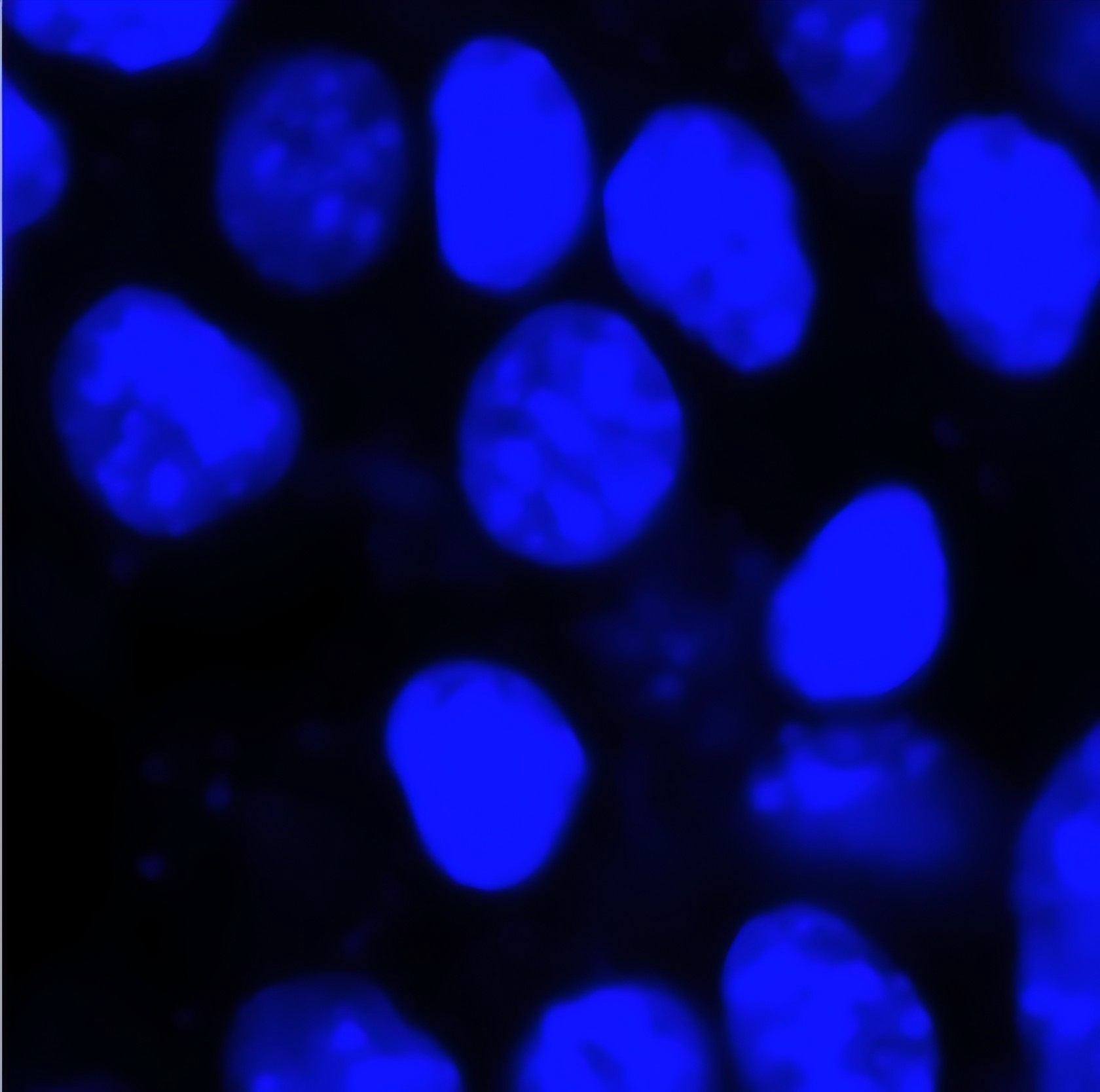

Supplement: Supplementary file 19 — Represent Raw Images [file 41419_2026_8682_MOESM19_ESM.zip › IFRAW/2-4-3.bmp]

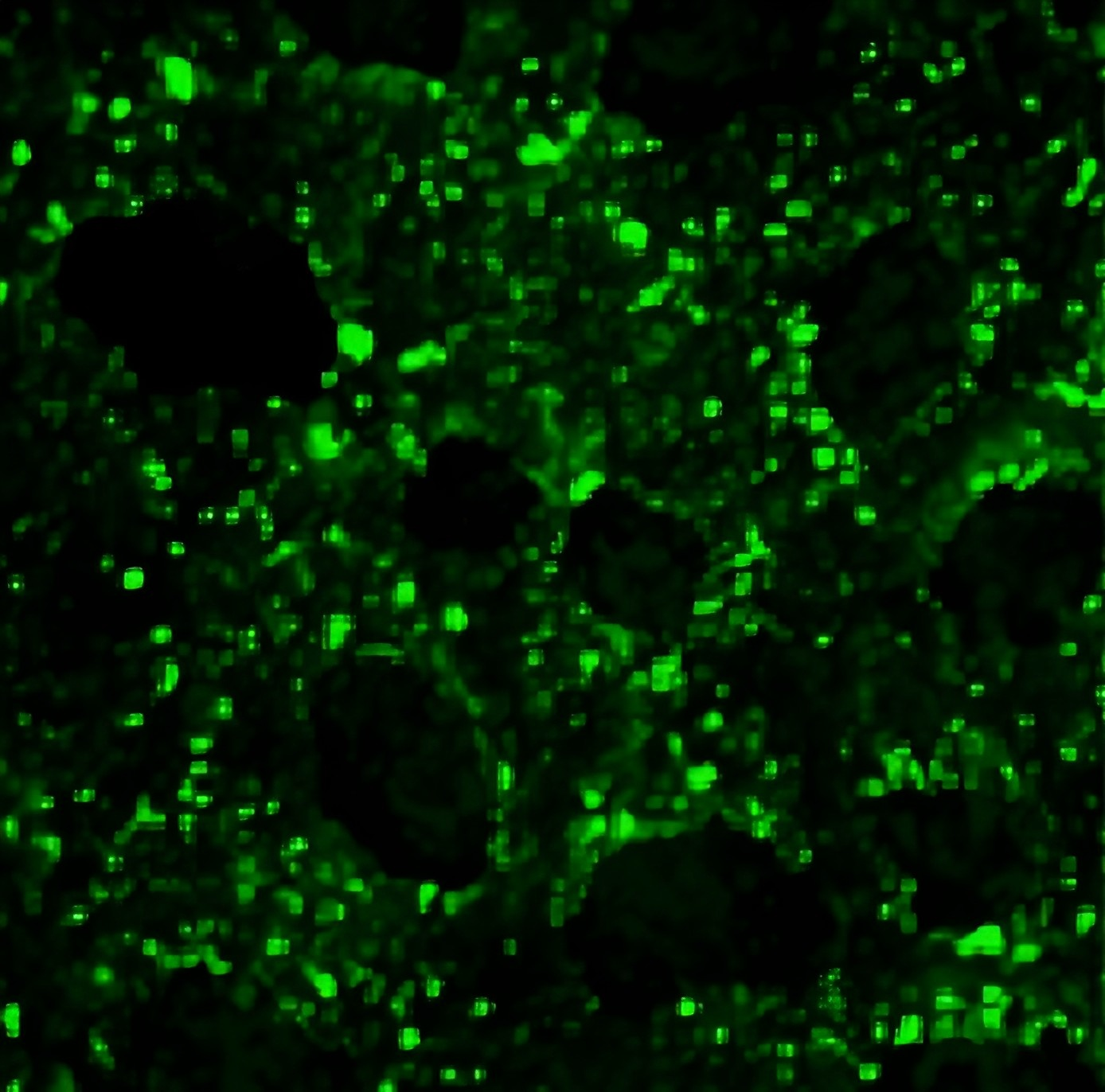

Supplement: Supplementary file 19 — Represent Raw Images [file 41419_2026_8682_MOESM19_ESM.zip › IFRAW/3-1-2.bmp]

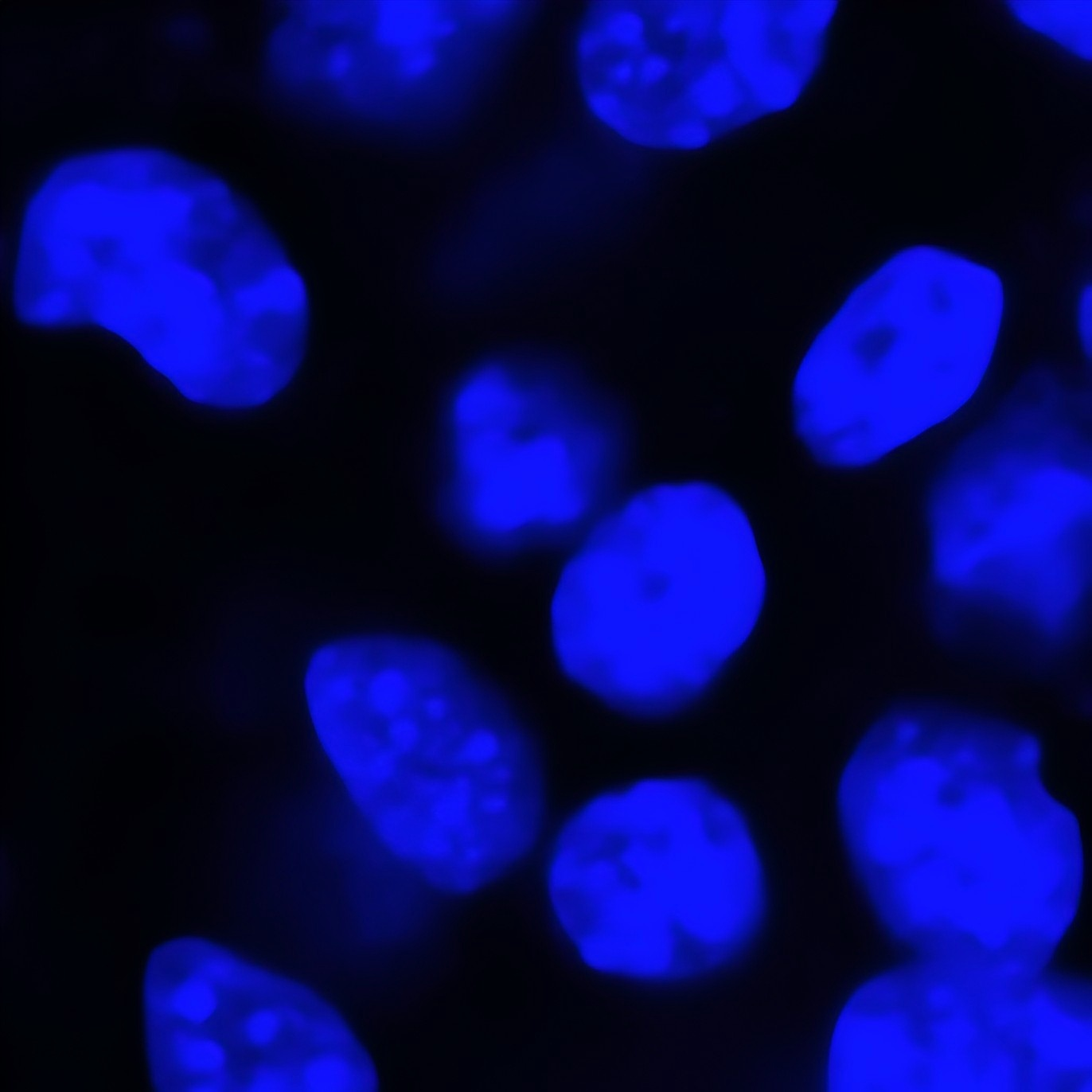

Supplement: Supplementary file 19 — Represent Raw Images [file 41419_2026_8682_MOESM19_ESM.zip › IFRAW/3-1-3.bmp]

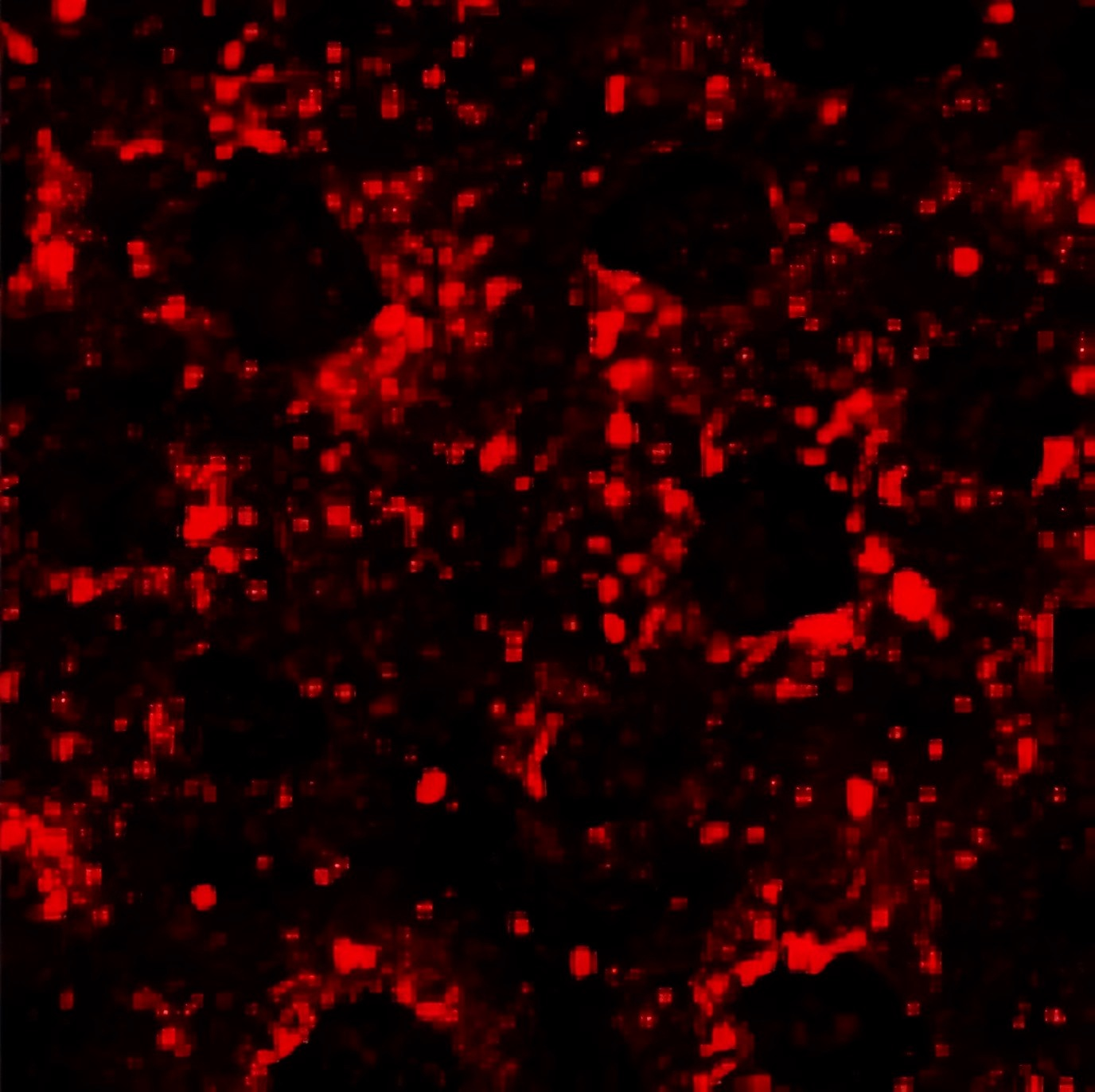

Supplement: Supplementary file 19 — Represent Raw Images [file 41419_2026_8682_MOESM19_ESM.zip › IFRAW/3-2-1.bmp]

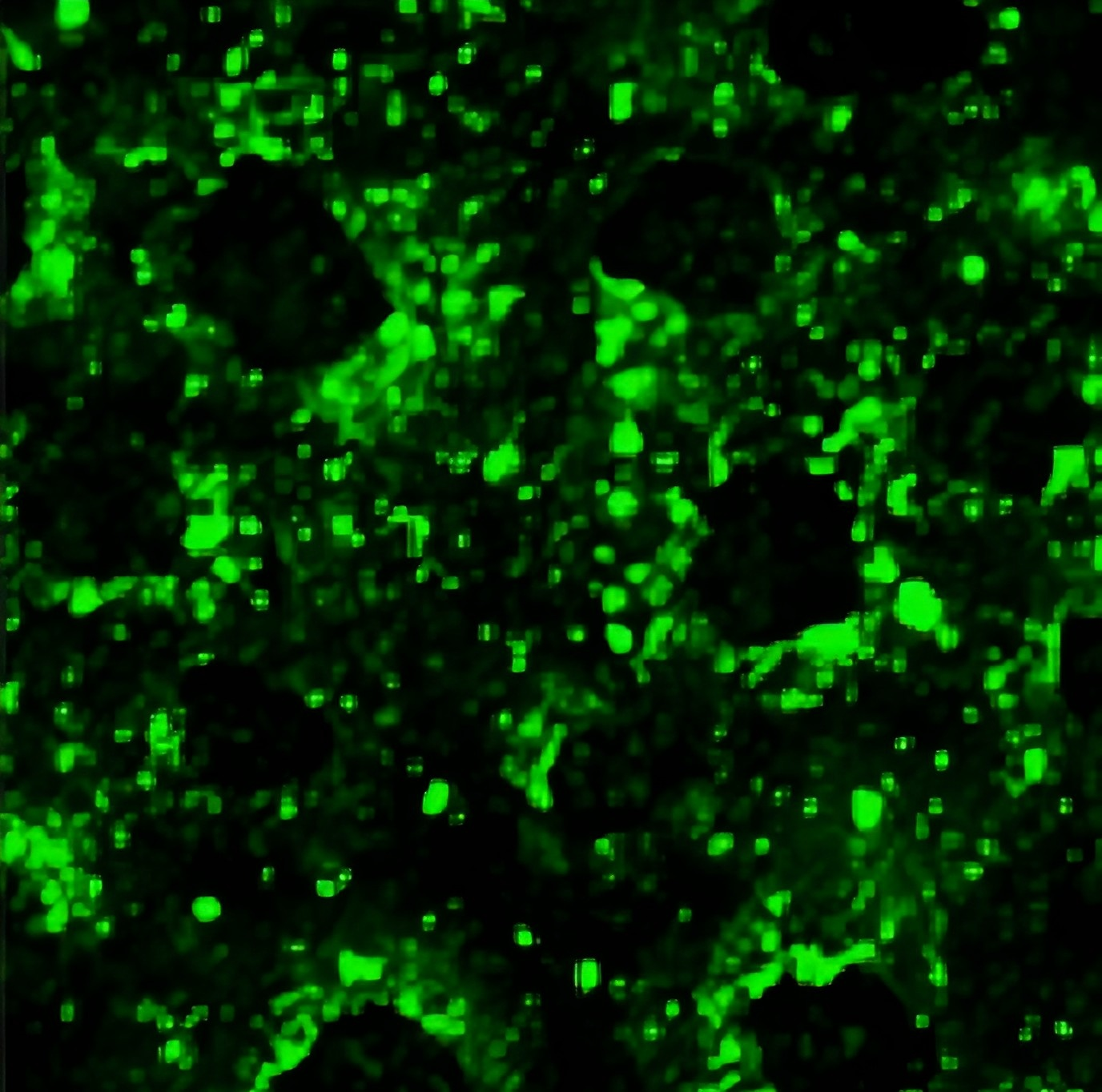

Supplement: Supplementary file 19 — Represent Raw Images [file 41419_2026_8682_MOESM19_ESM.zip › IFRAW/3-2-2.bmp]

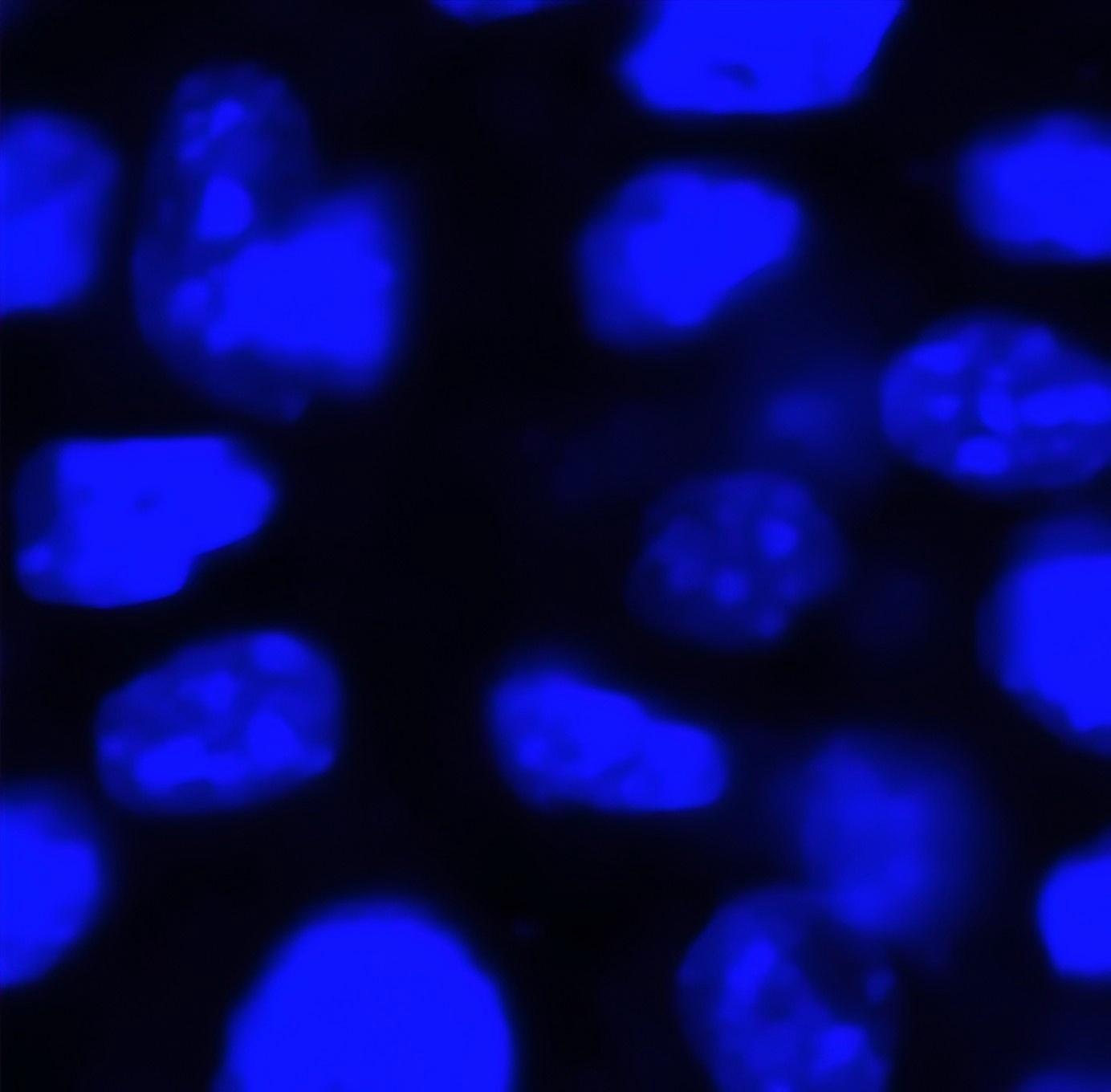

Supplement: Supplementary file 19 — Represent Raw Images [file 41419_2026_8682_MOESM19_ESM.zip › IFRAW/3-2-3.bmp]

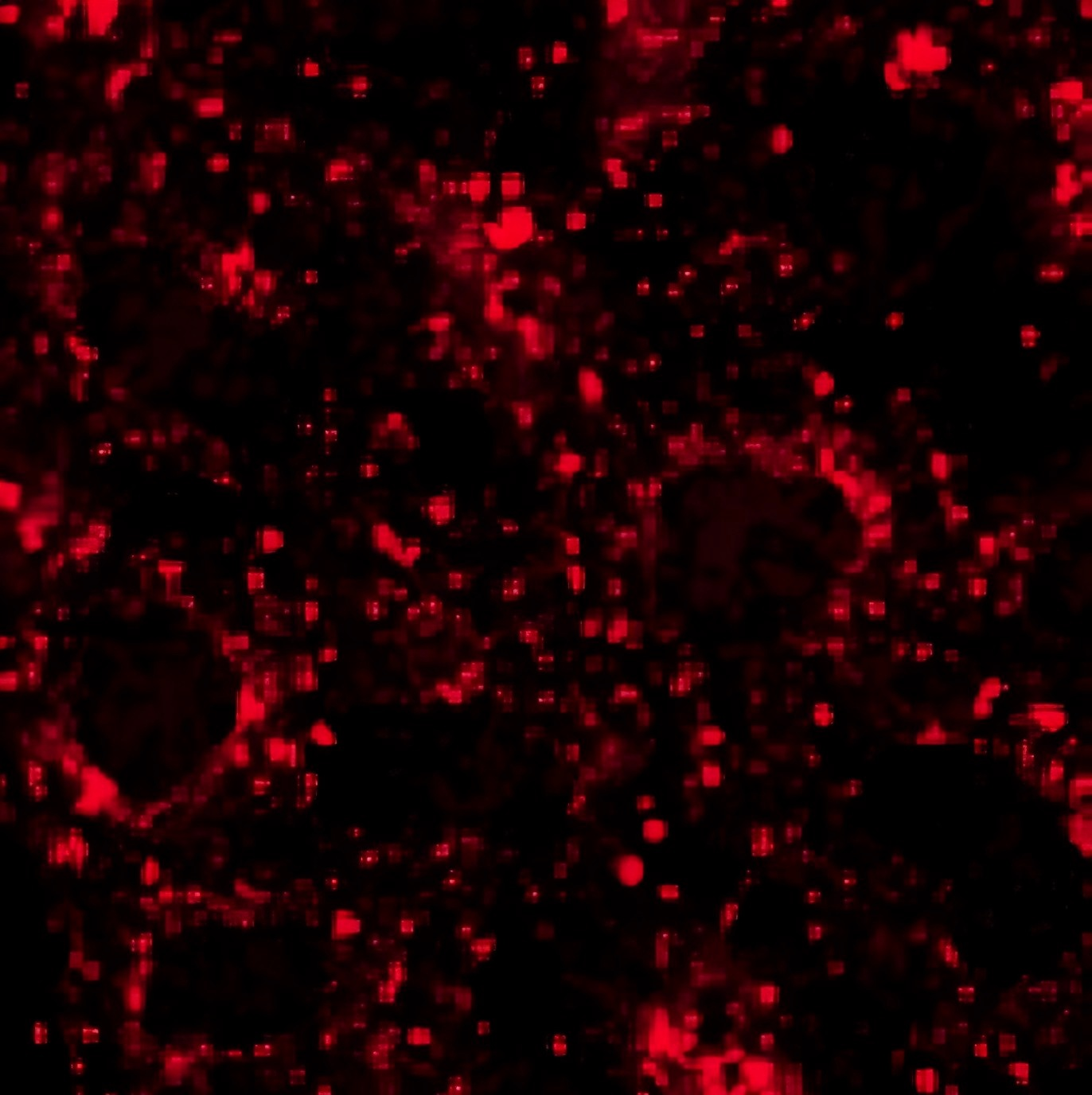

Supplement: Supplementary file 19 — Represent Raw Images [file 41419_2026_8682_MOESM19_ESM.zip › IFRAW/3-3-1.bmp]

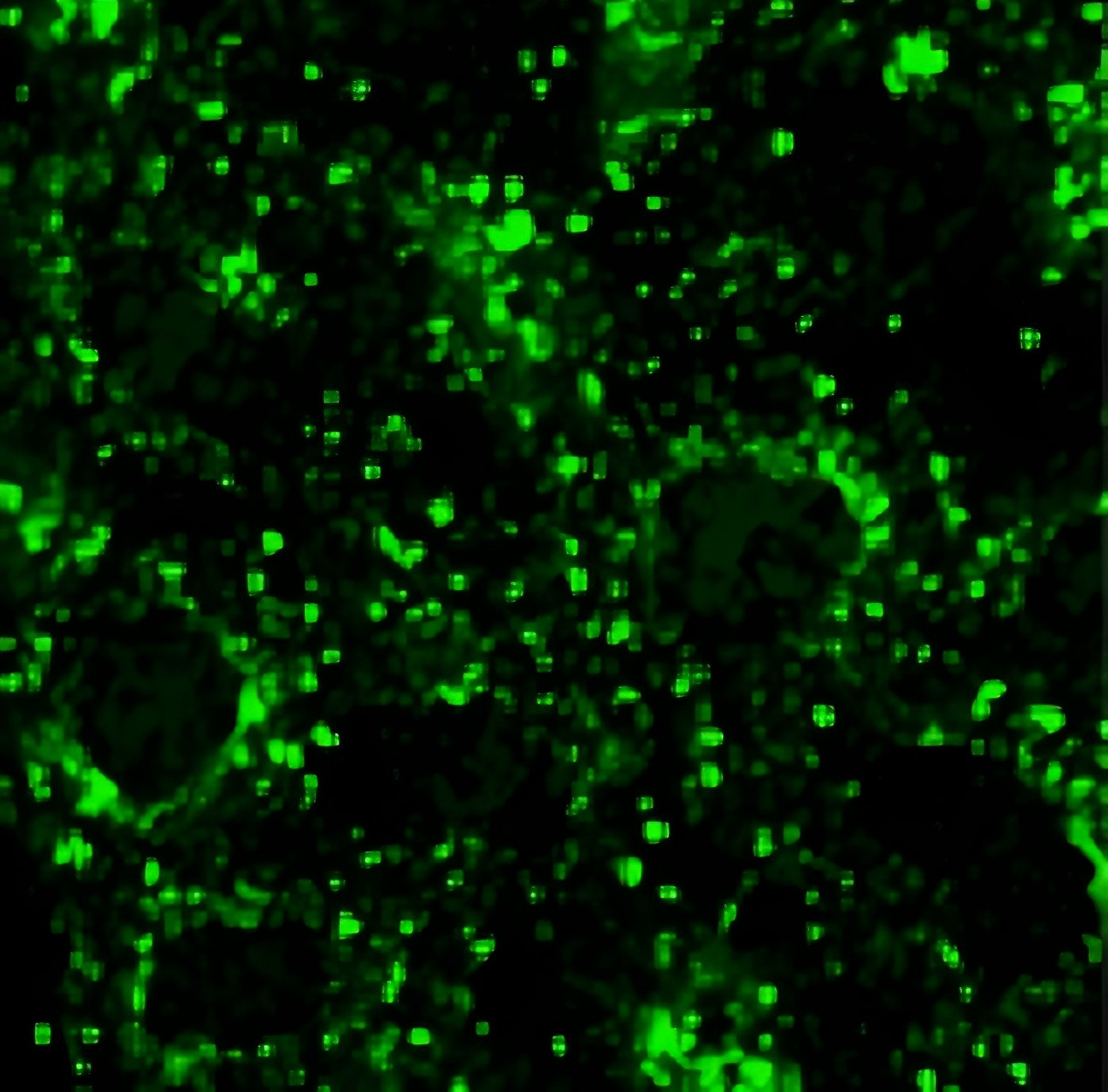

Supplement: Supplementary file 19 — Represent Raw Images [file 41419_2026_8682_MOESM19_ESM.zip › IFRAW/3-3-2.bmp]

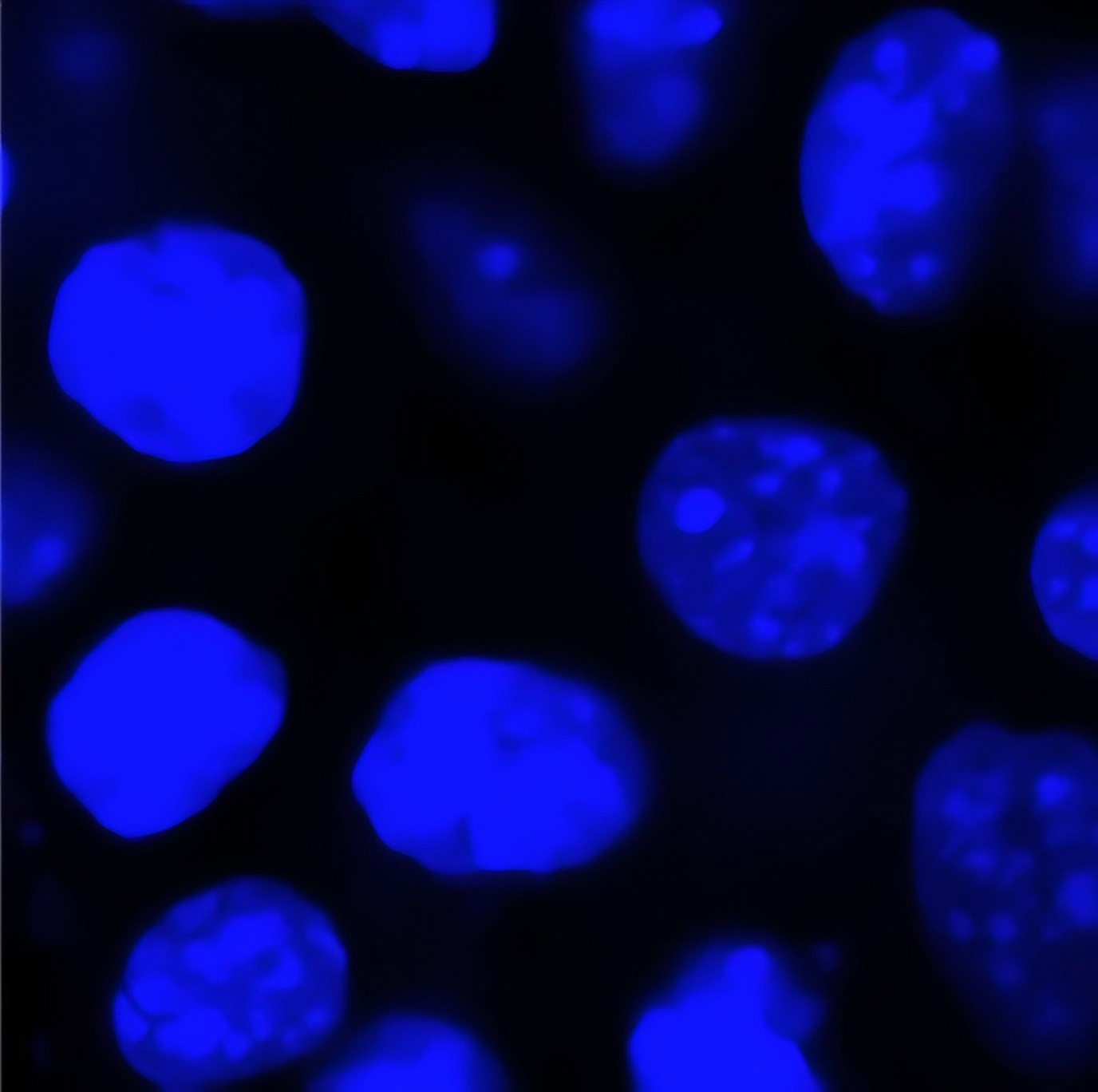

Supplement: Supplementary file 19 — Represent Raw Images [file 41419_2026_8682_MOESM19_ESM.zip › IFRAW/3-3-3.bmp]

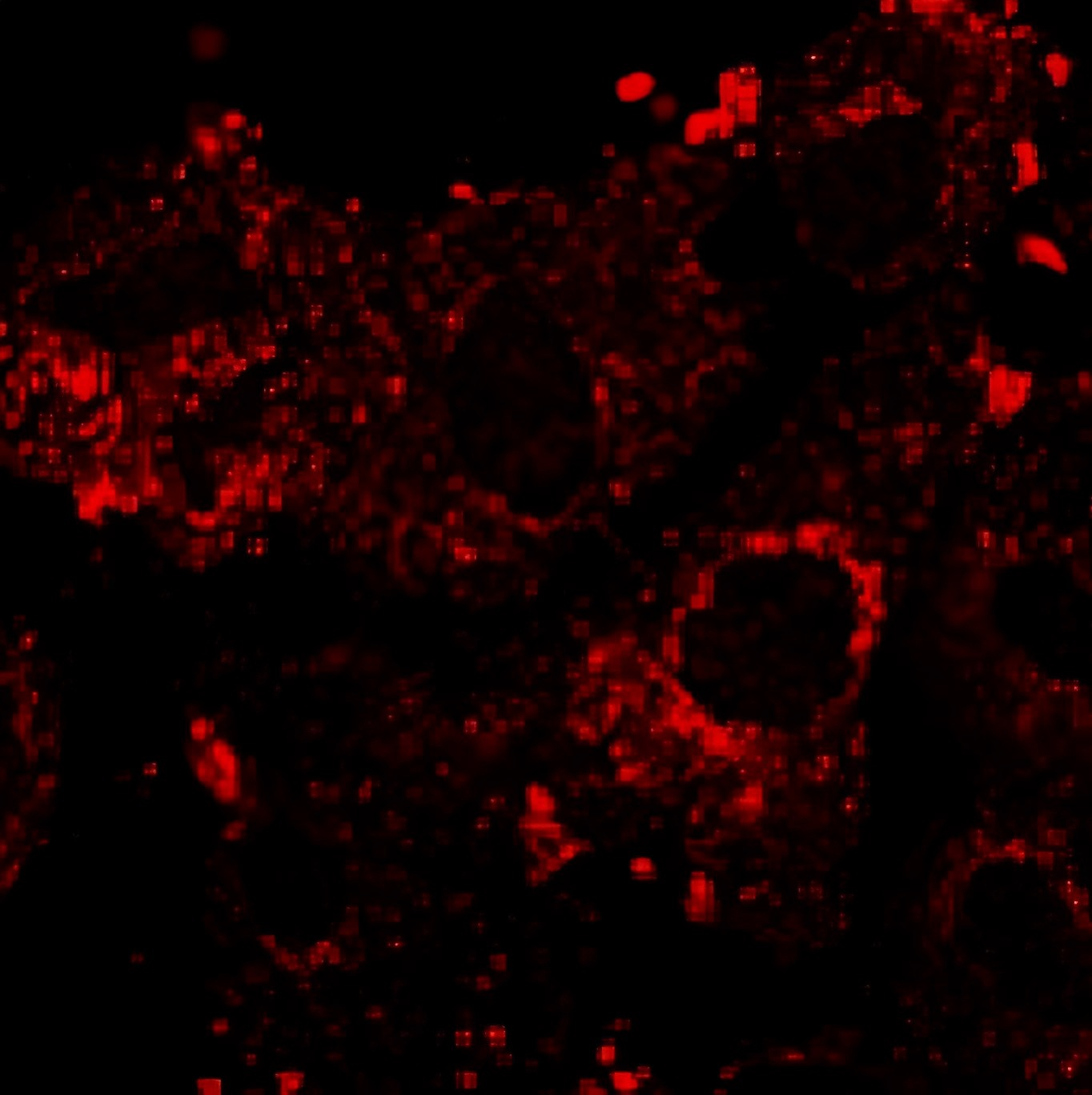

Supplement: Supplementary file 19 — Represent Raw Images [file 41419_2026_8682_MOESM19_ESM.zip › IFRAW/3-4-1.bmp]

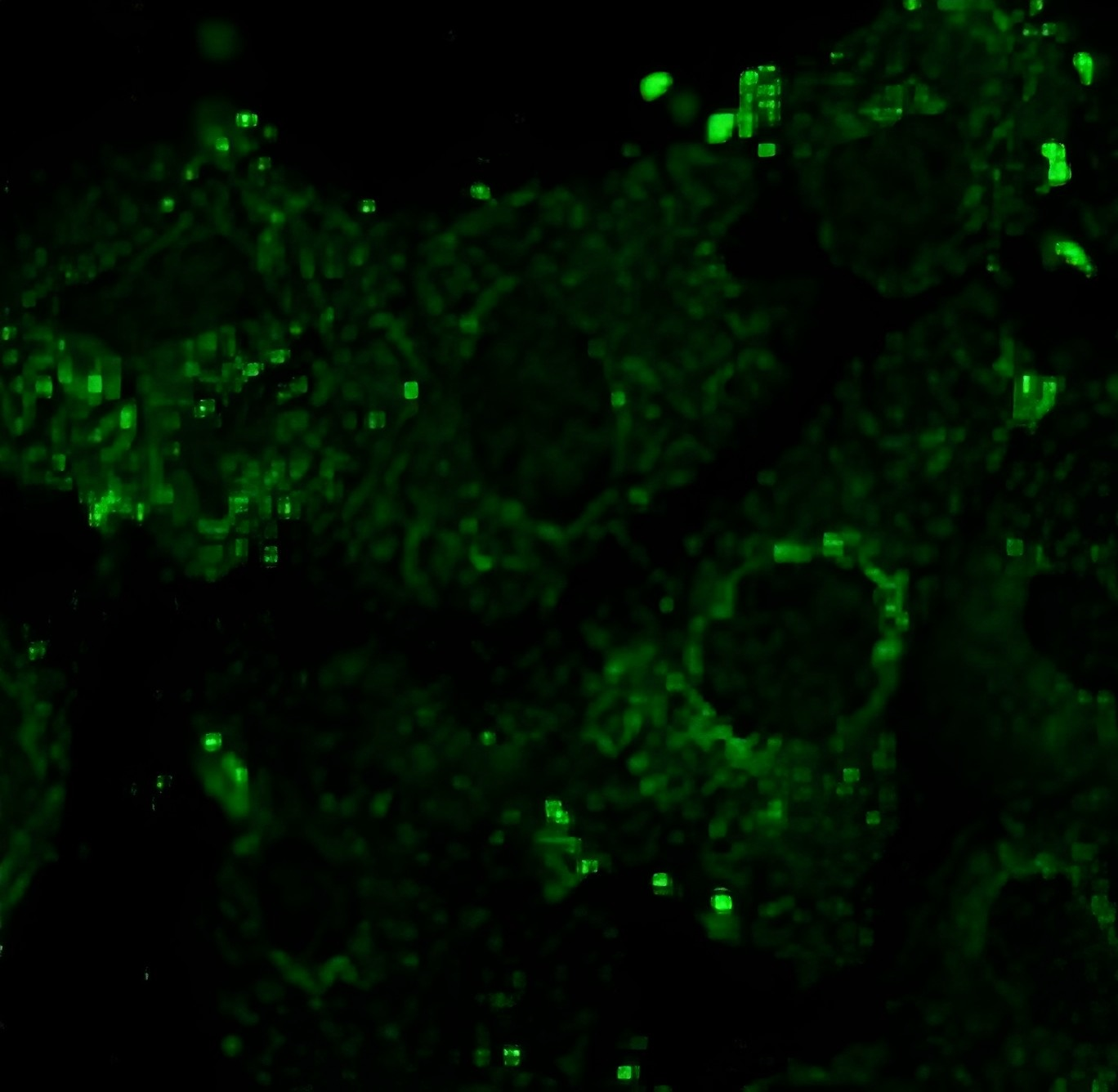

Supplement: Supplementary file 19 — Represent Raw Images [file 41419_2026_8682_MOESM19_ESM.zip › IFRAW/3-4-2.bmp]

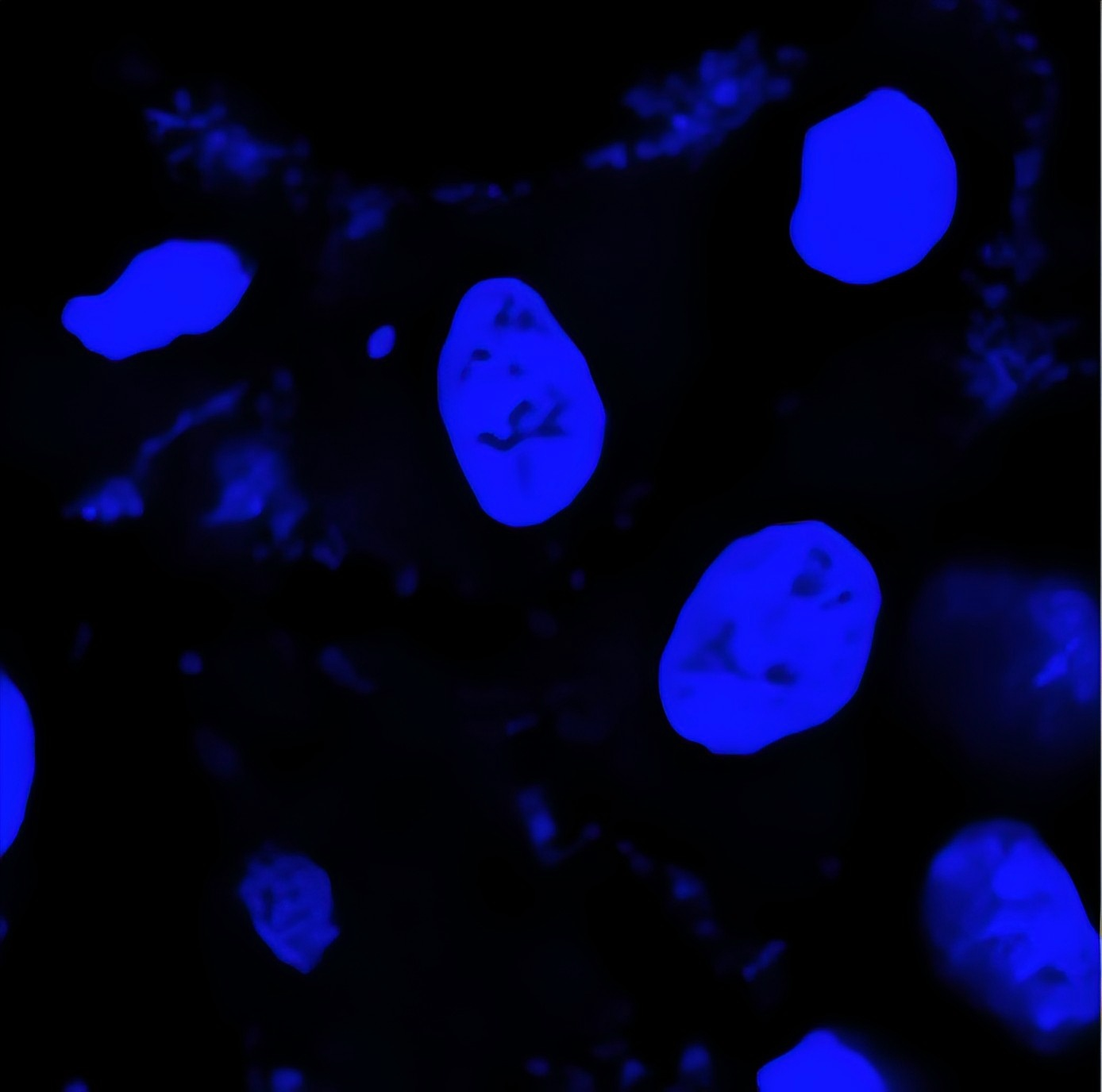

Supplement: Supplementary file 19 — Represent Raw Images [file 41419_2026_8682_MOESM19_ESM.zip › IFRAW/3-4-3.bmp]

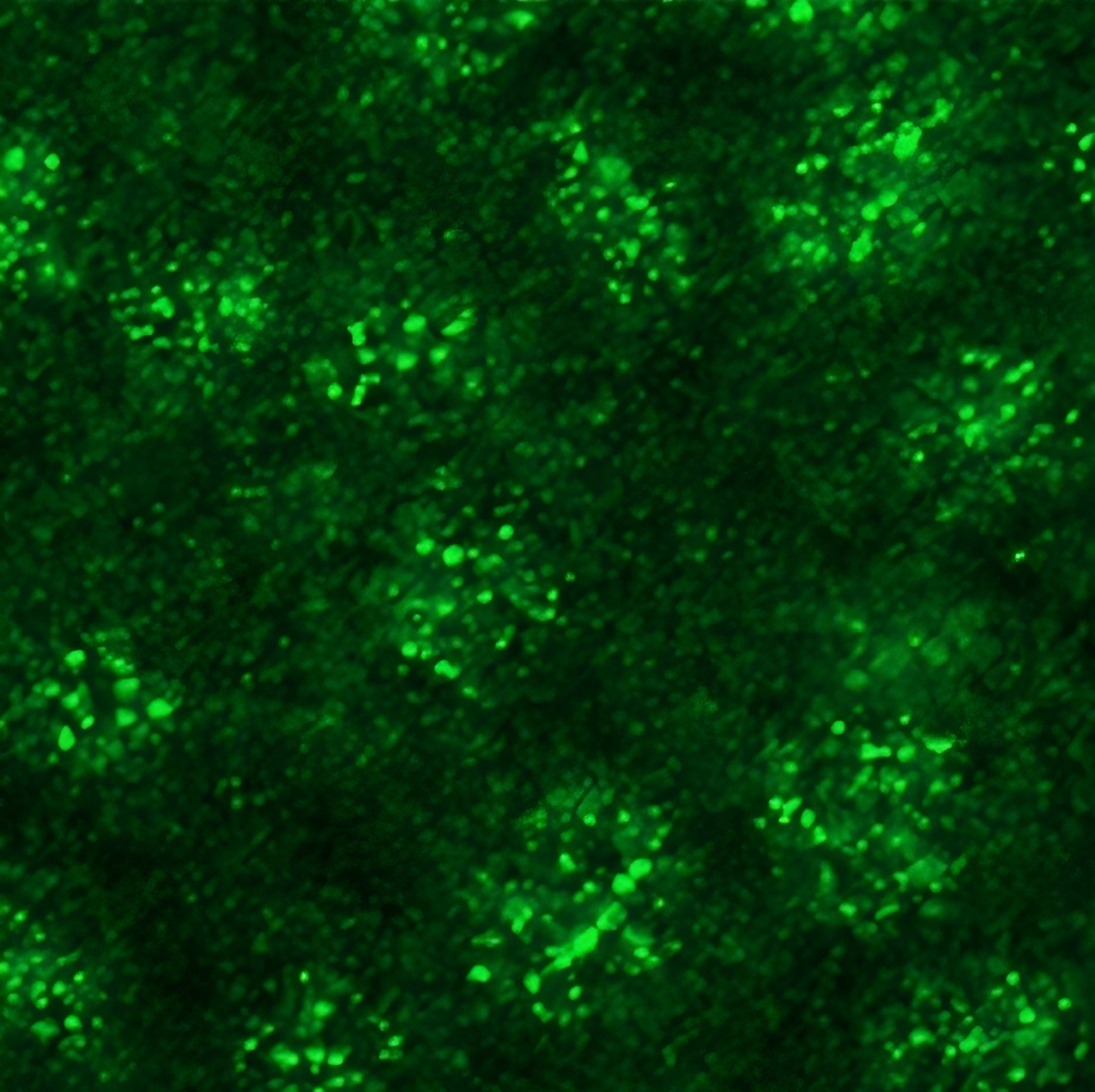

Supplement: Supplementary file 19 — Represent Raw Images [file 41419_2026_8682_MOESM19_ESM.zip › IFRAW/4-1-1.bmp]

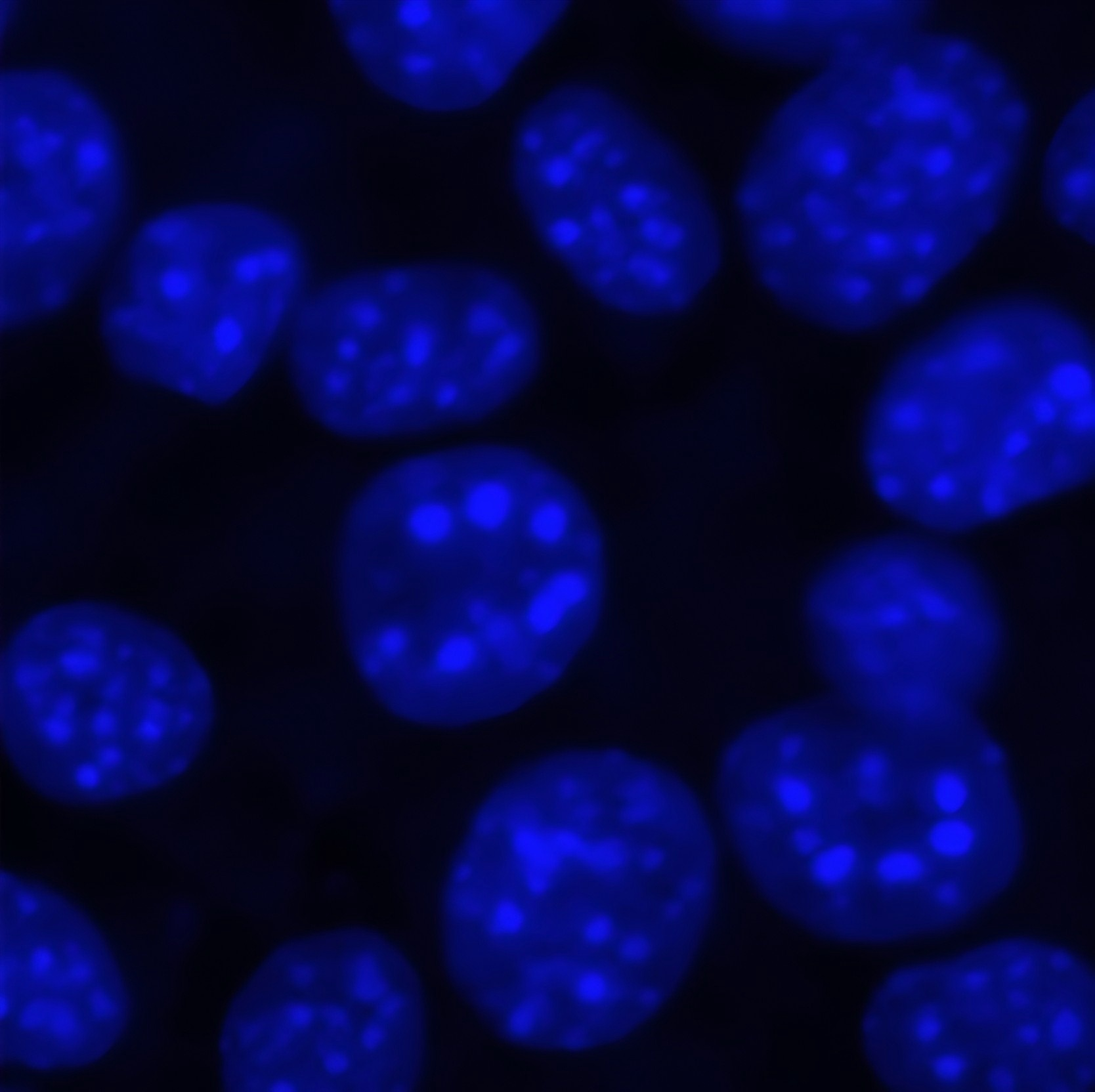

Supplement: Supplementary file 19 — Represent Raw Images [file 41419_2026_8682_MOESM19_ESM.zip › IFRAW/4-1-2.bmp]

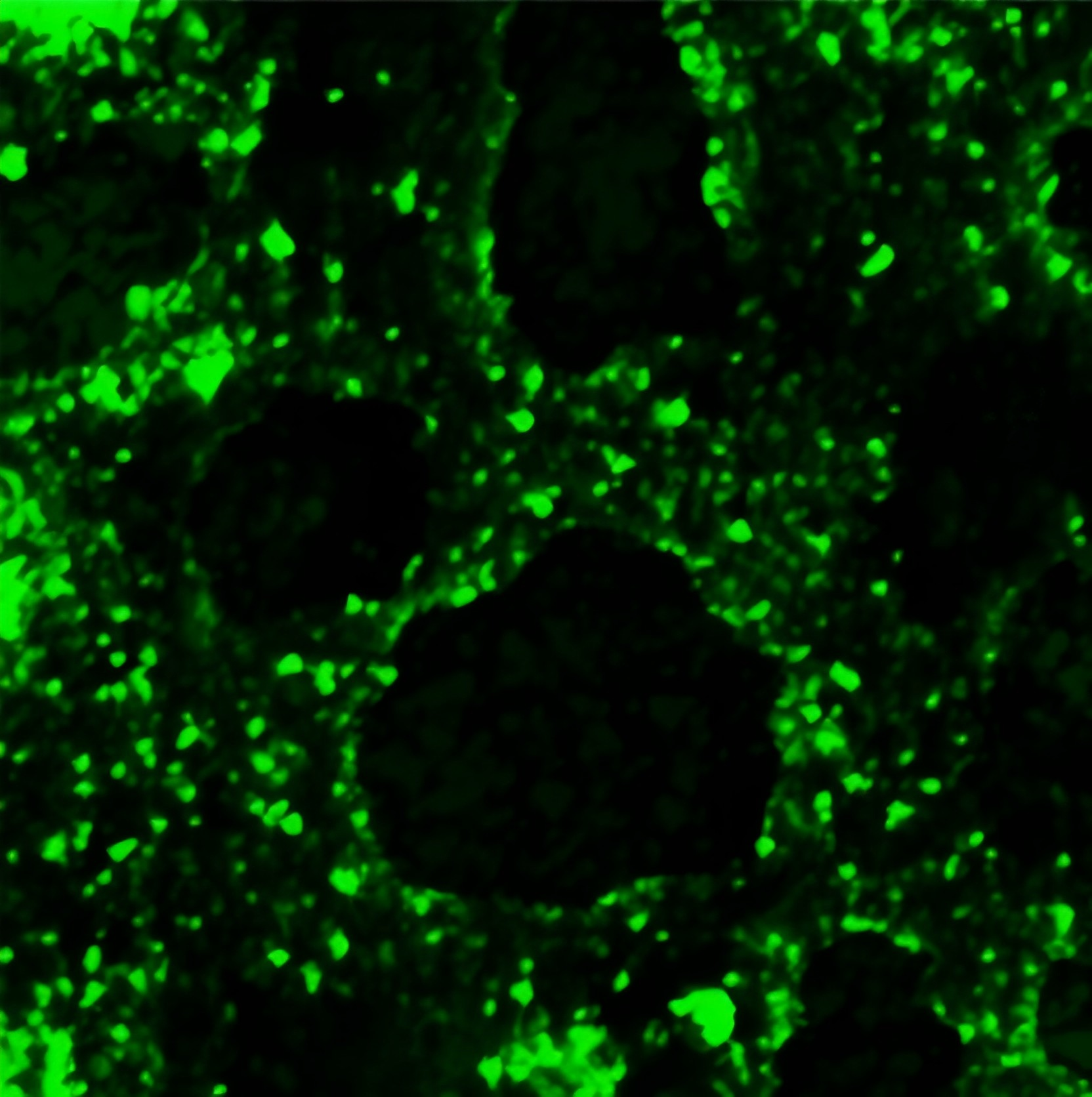

Supplement: Supplementary file 19 — Represent Raw Images [file 41419_2026_8682_MOESM19_ESM.zip › IFRAW/4-2-1.bmp]

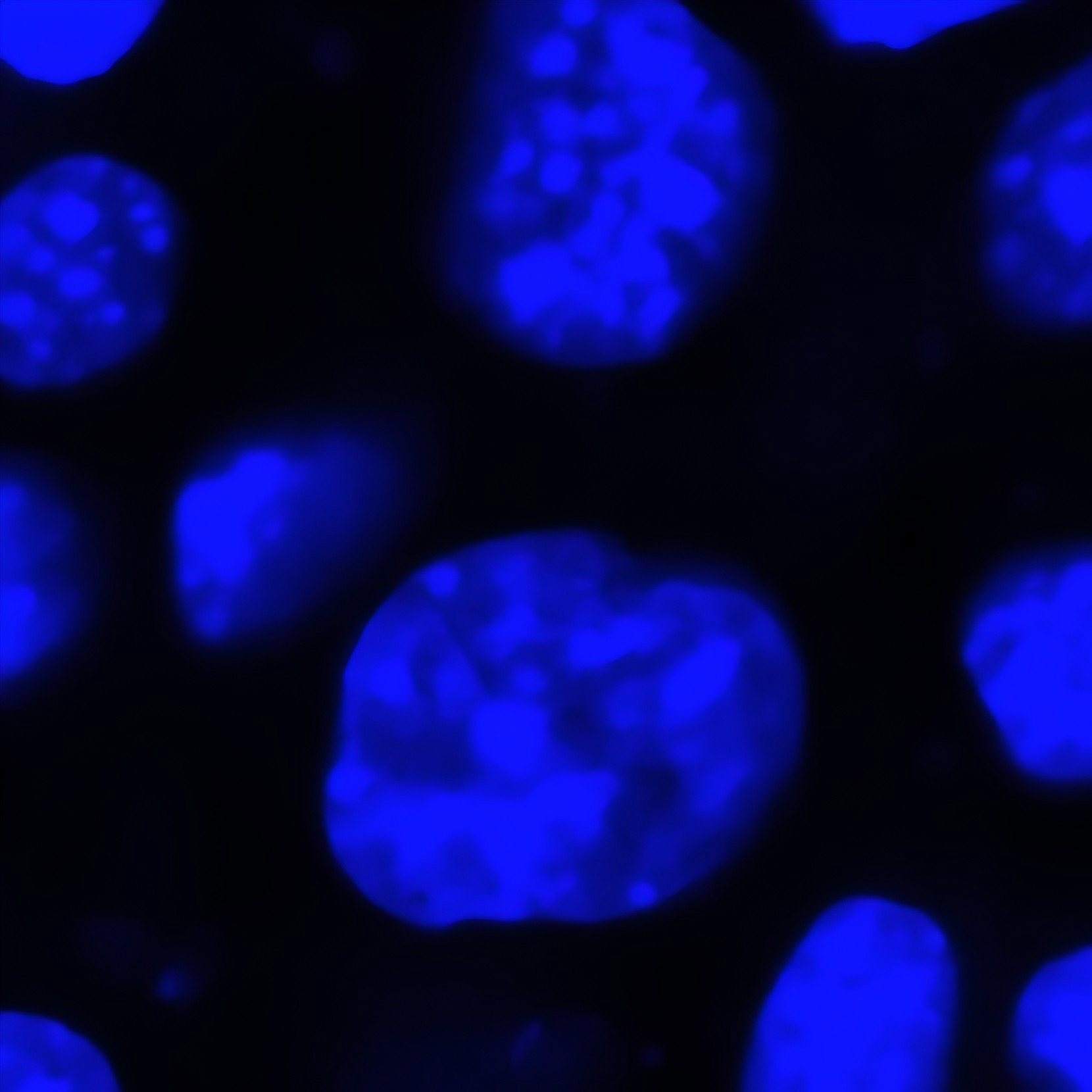

Supplement: Supplementary file 19 — Represent Raw Images [file 41419_2026_8682_MOESM19_ESM.zip › IFRAW/4-2-2.bmp]

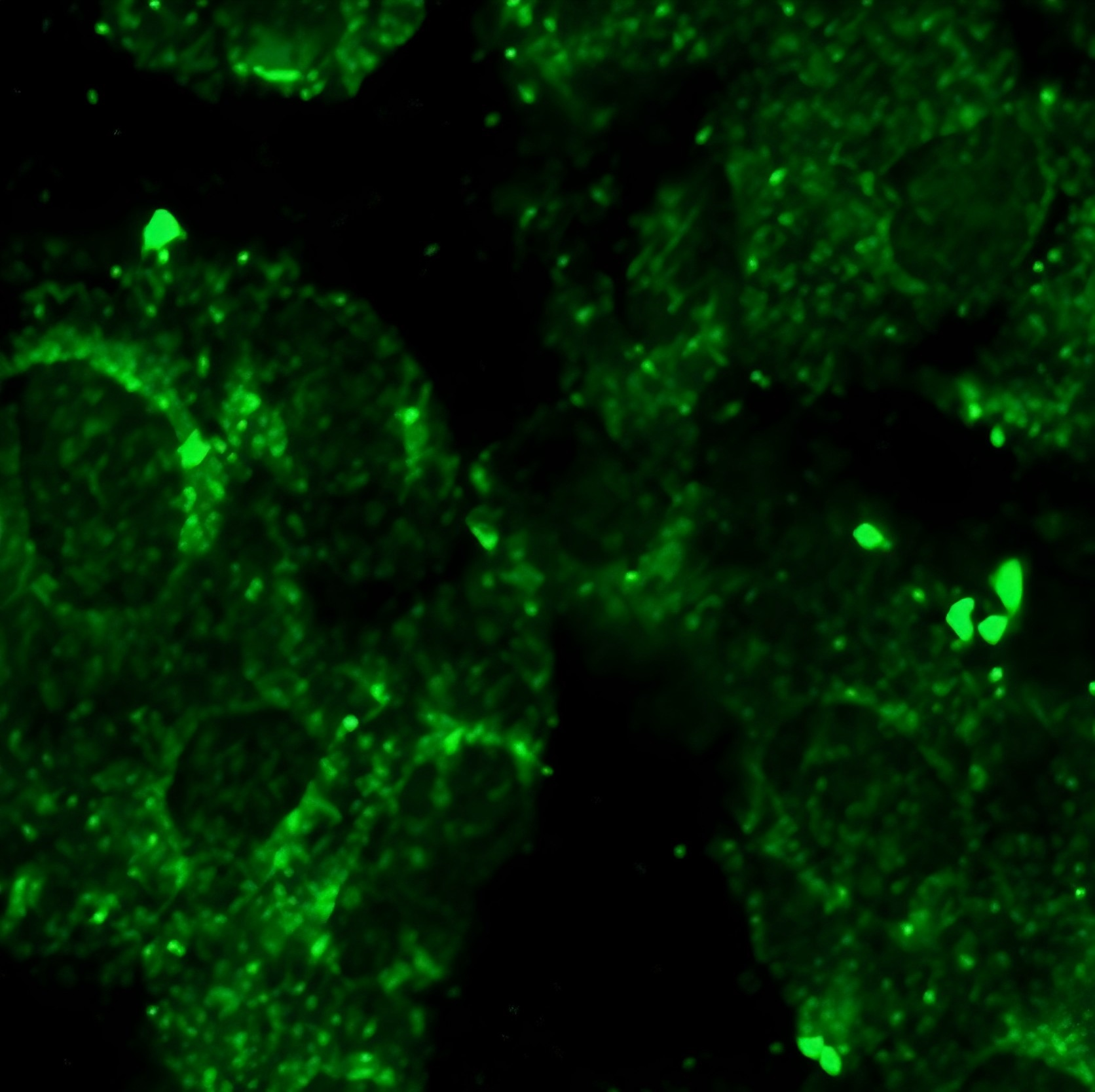

Supplement: Supplementary file 19 — Represent Raw Images [file 41419_2026_8682_MOESM19_ESM.zip › IFRAW/4-3-1.bmp]

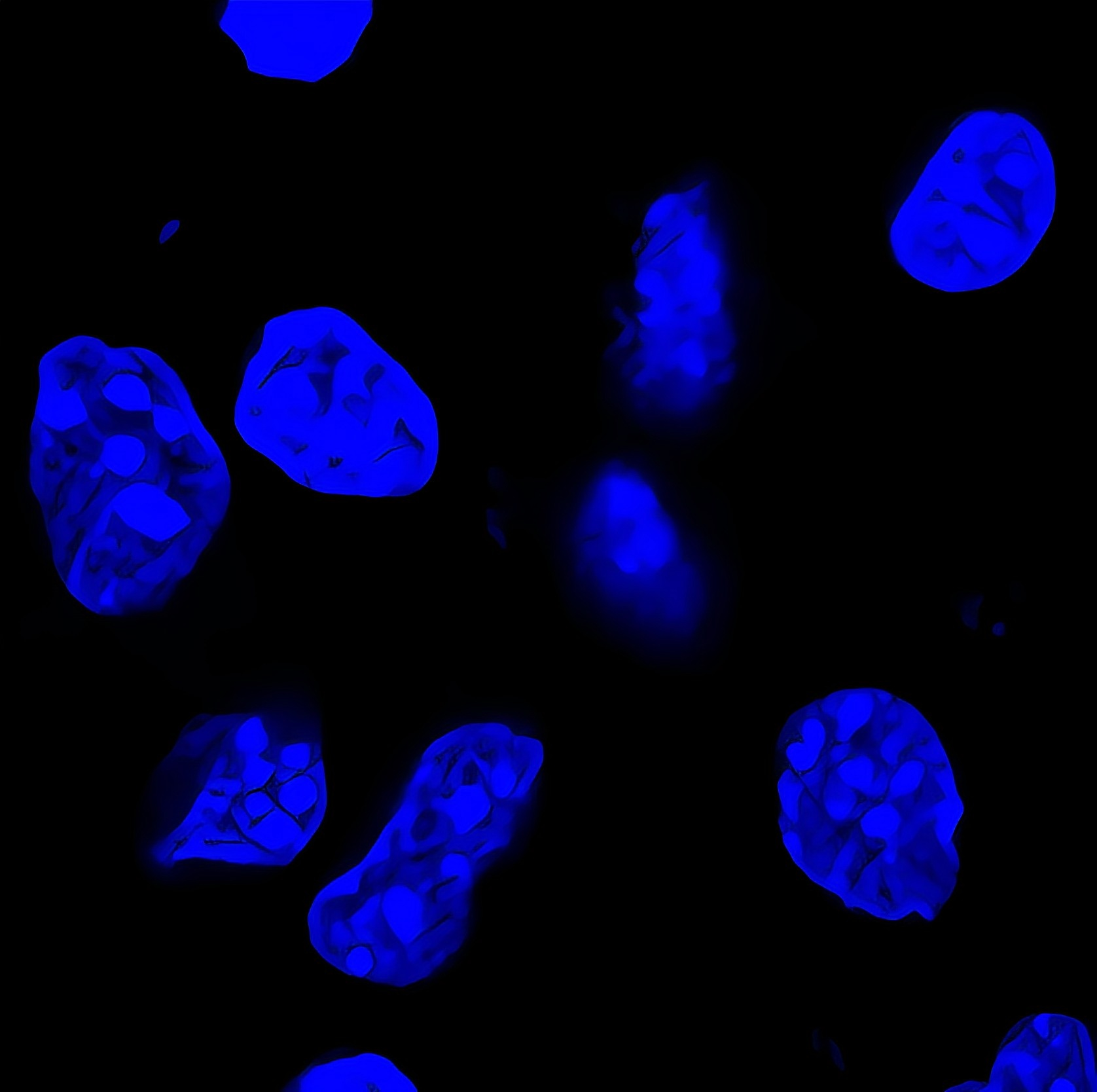

Supplement: Supplementary file 19 — Represent Raw Images [file 41419_2026_8682_MOESM19_ESM.zip › IFRAW/4-3-2.bmp]

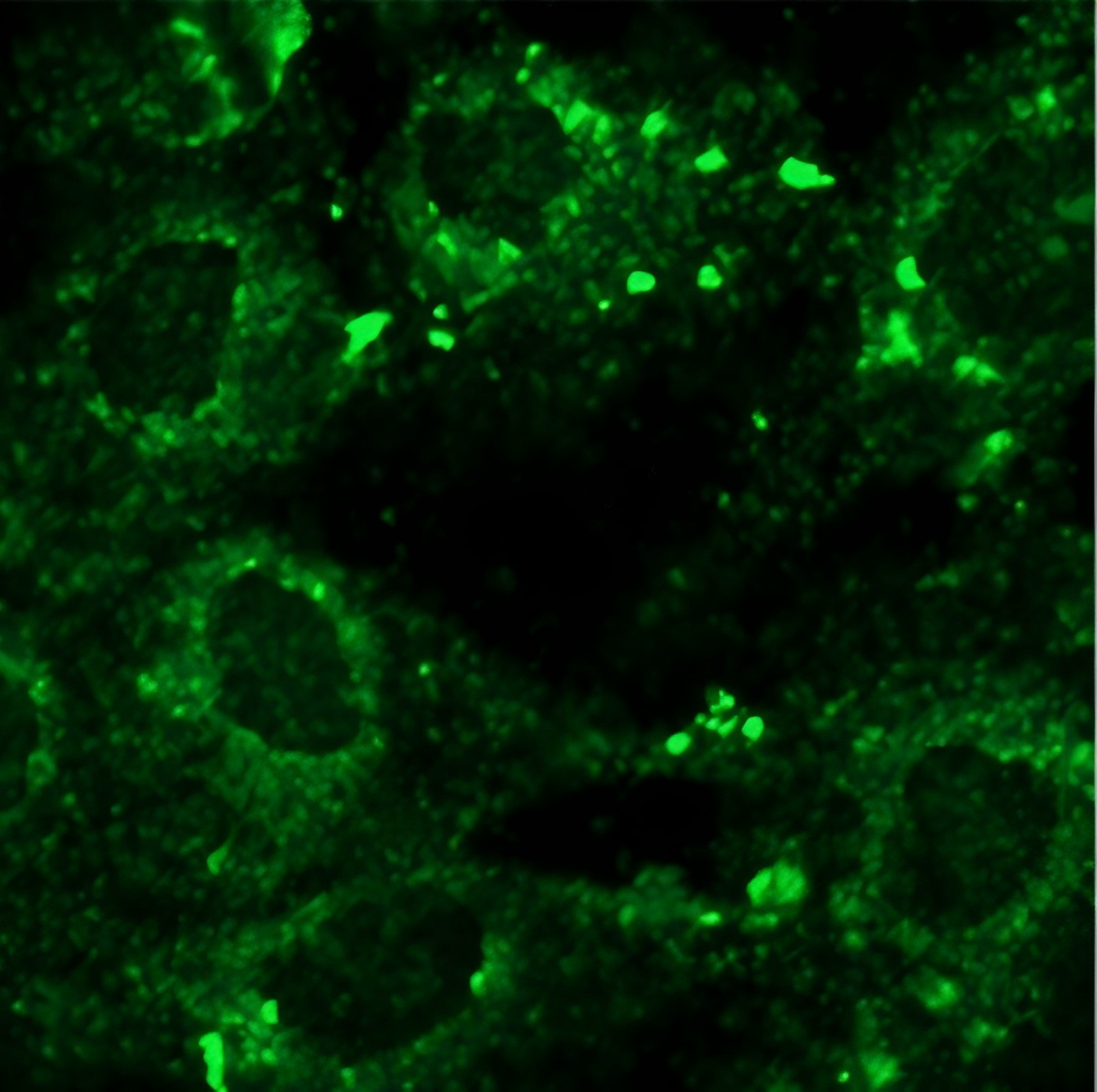

Supplement: Supplementary file 19 — Represent Raw Images [file 41419_2026_8682_MOESM19_ESM.zip › IFRAW/4-4-1.bmp]

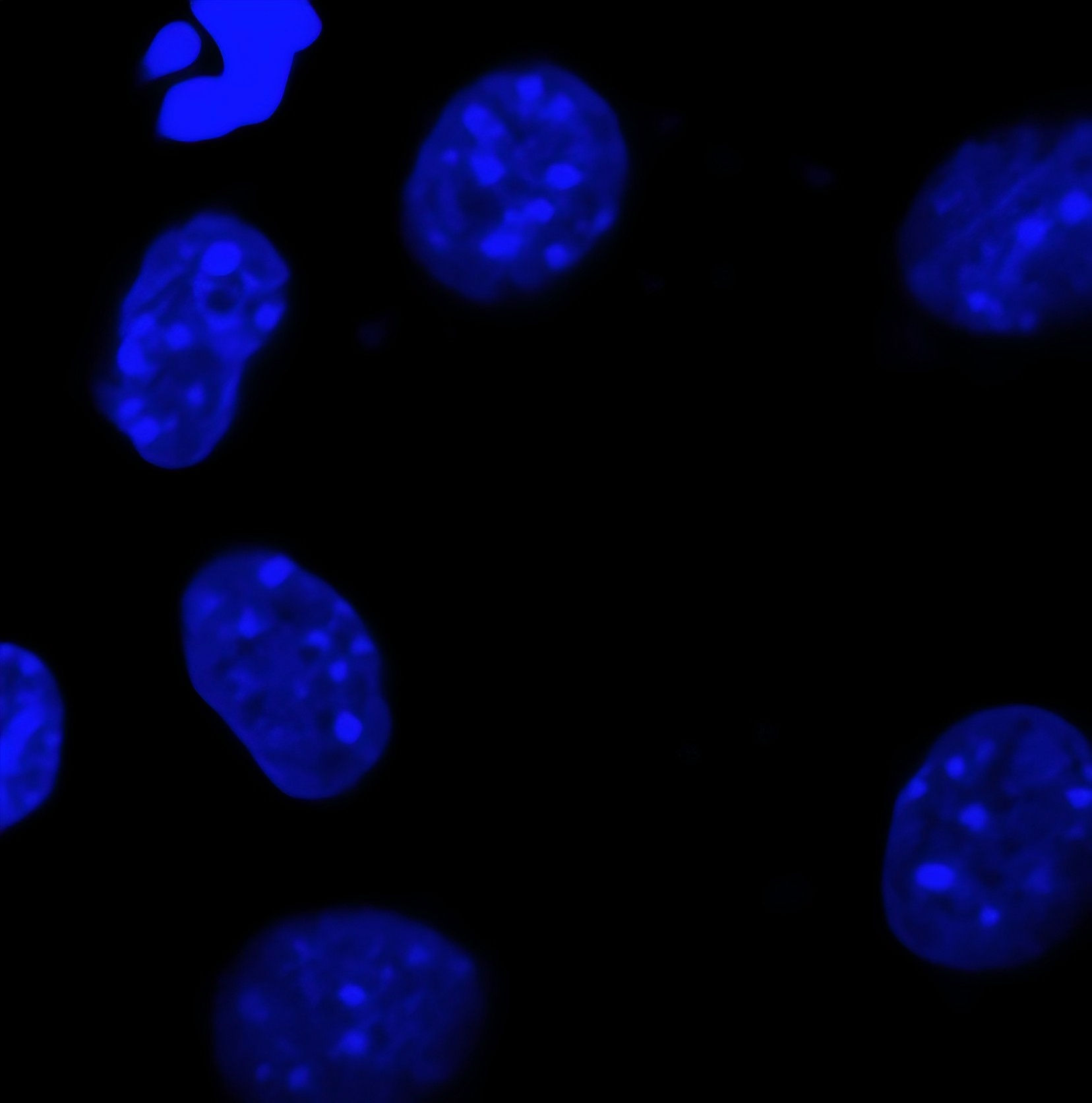

Supplement: Supplementary file 19 — Represent Raw Images [file 41419_2026_8682_MOESM19_ESM.zip › IFRAW/4-4-2.bmp]

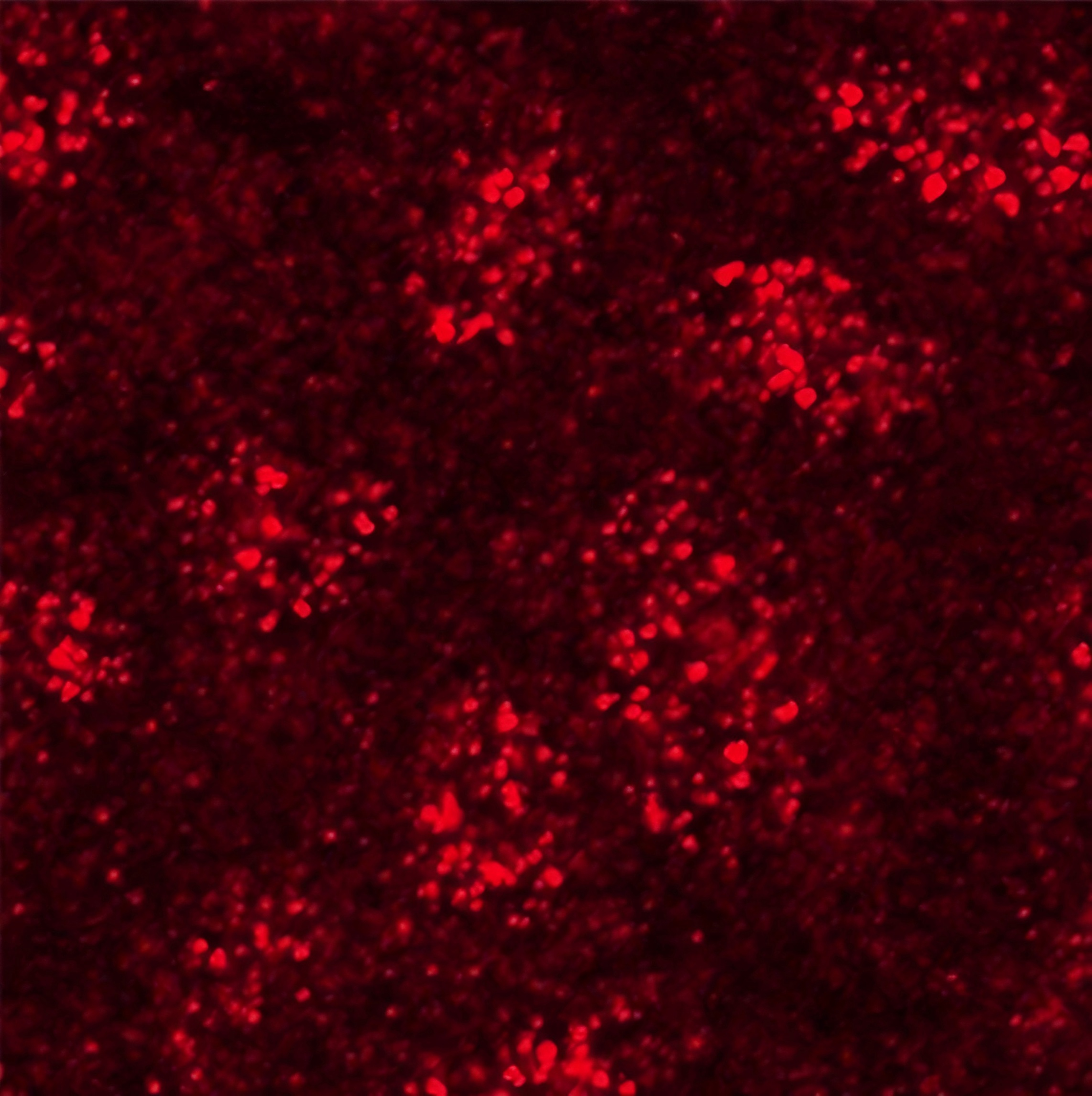

Supplement: Supplementary file 19 — Represent Raw Images [file 41419_2026_8682_MOESM19_ESM.zip › IFRAW/5-1-1.bmp]

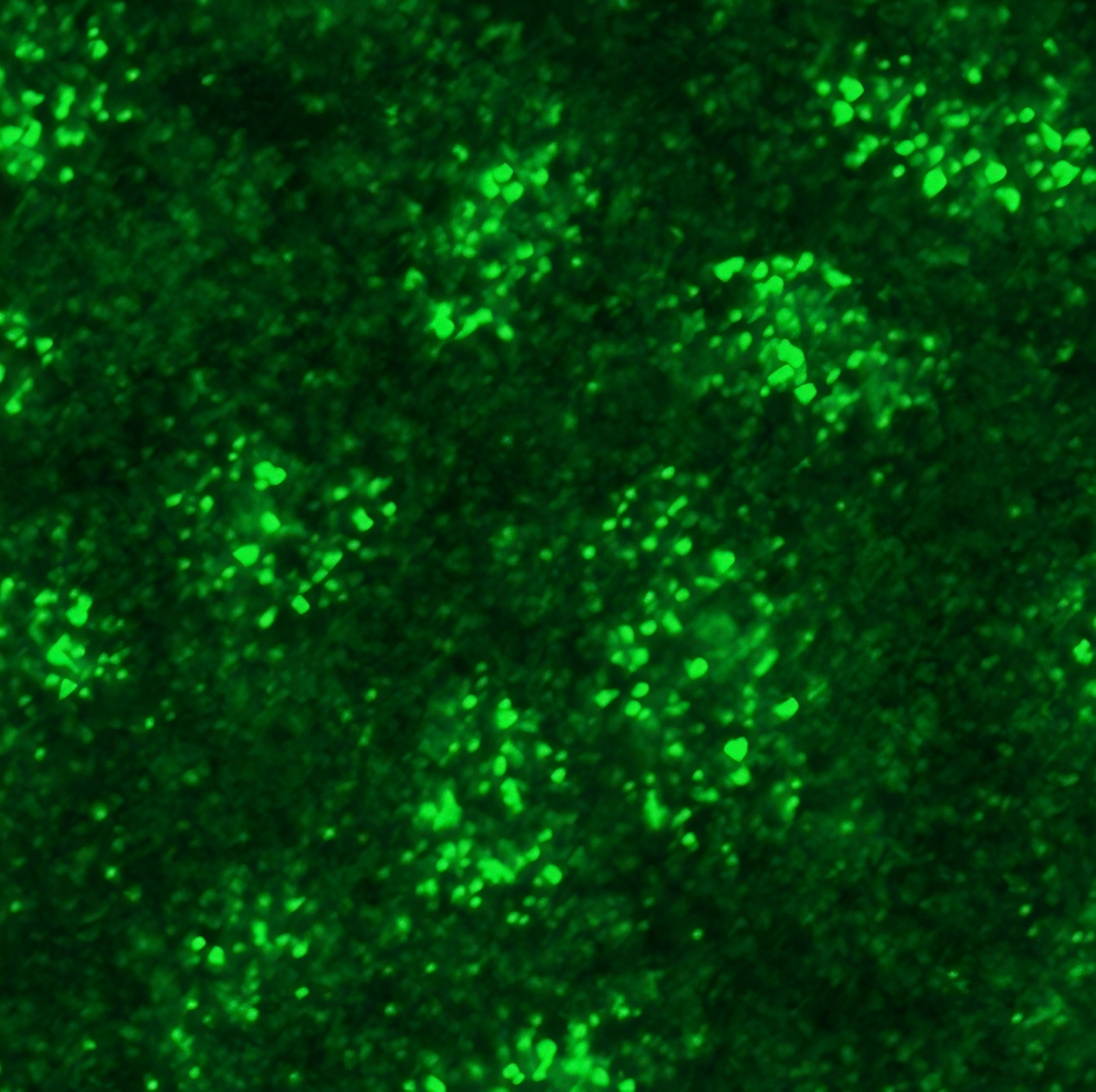

Supplement: Supplementary file 19 — Represent Raw Images [file 41419_2026_8682_MOESM19_ESM.zip › IFRAW/5-1-2.bmp]

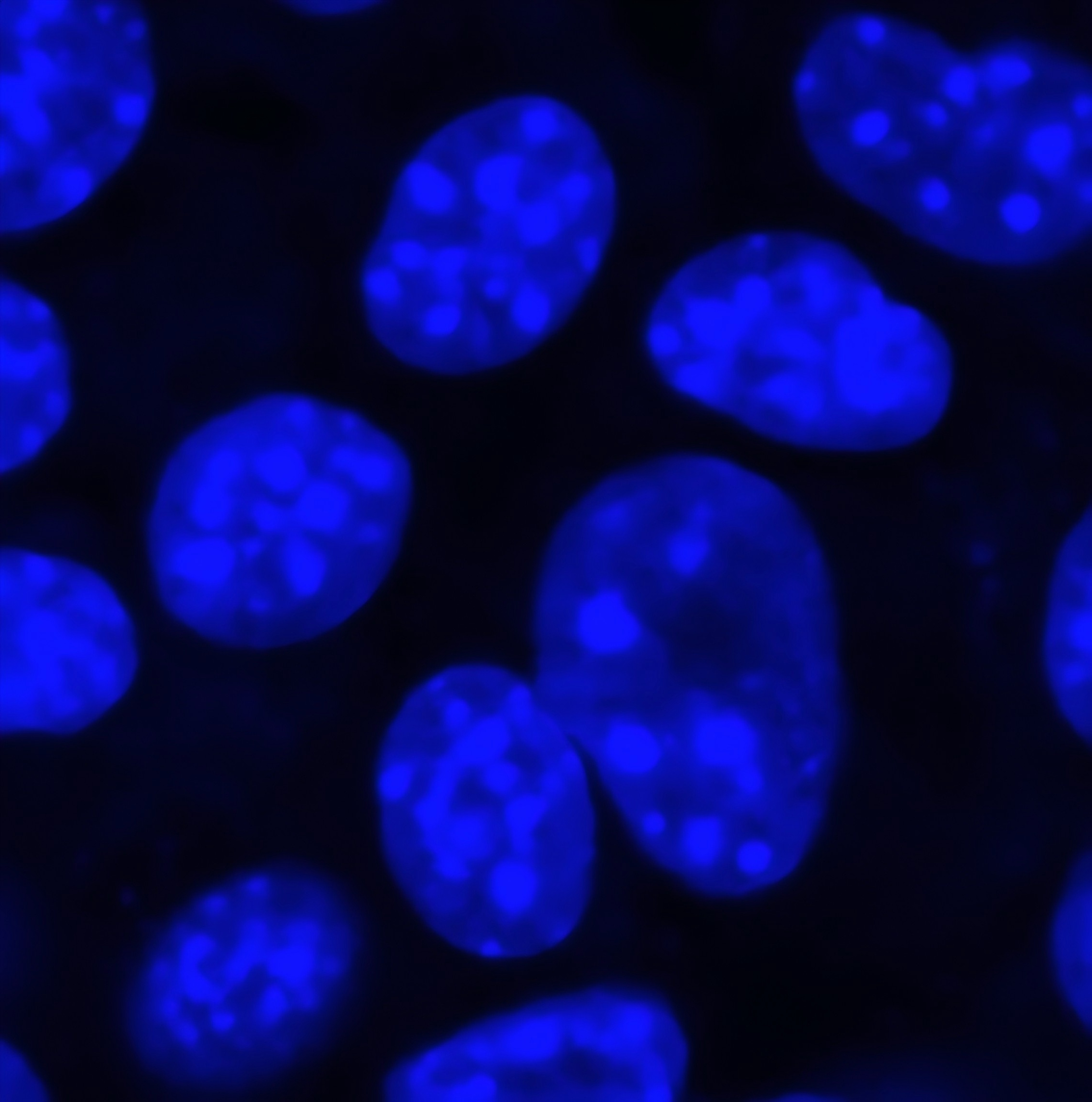

Supplement: Supplementary file 19 — Represent Raw Images [file 41419_2026_8682_MOESM19_ESM.zip › IFRAW/5-1-3.bmp]

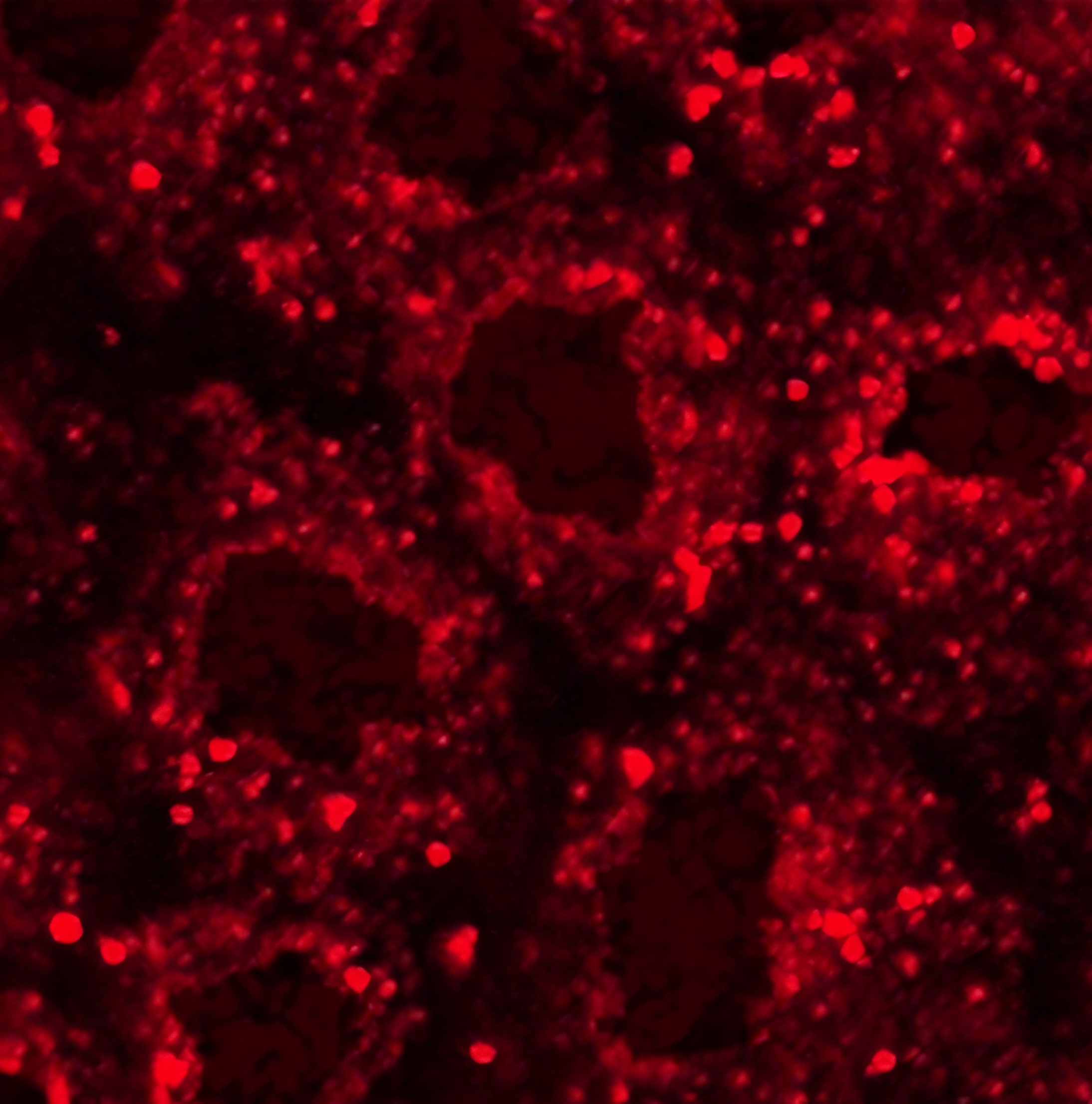

Supplement: Supplementary file 19 — Represent Raw Images [file 41419_2026_8682_MOESM19_ESM.zip › IFRAW/5-2-1.bmp]

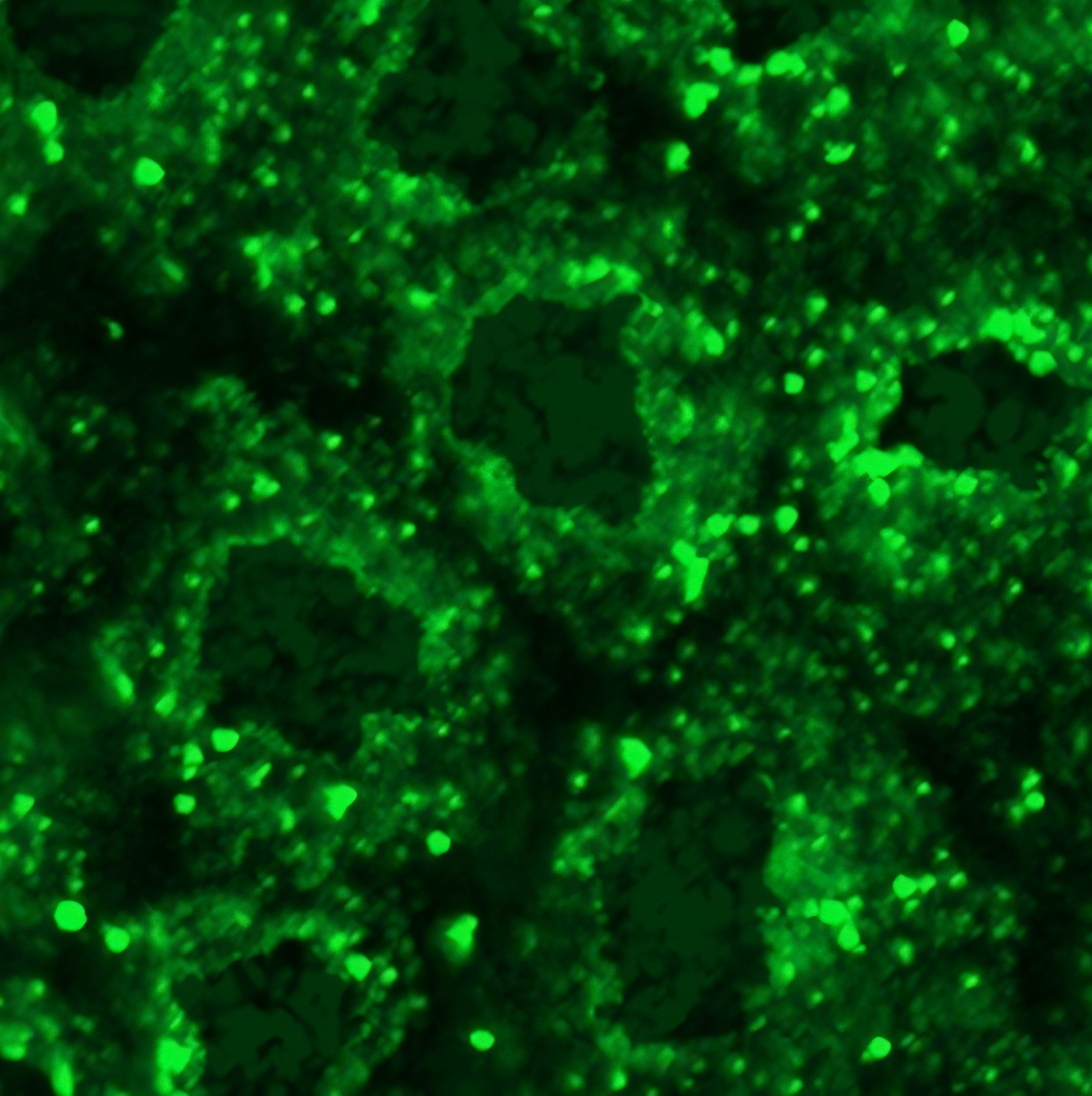

Supplement: Supplementary file 19 — Represent Raw Images [file 41419_2026_8682_MOESM19_ESM.zip › IFRAW/5-2-2.bmp]

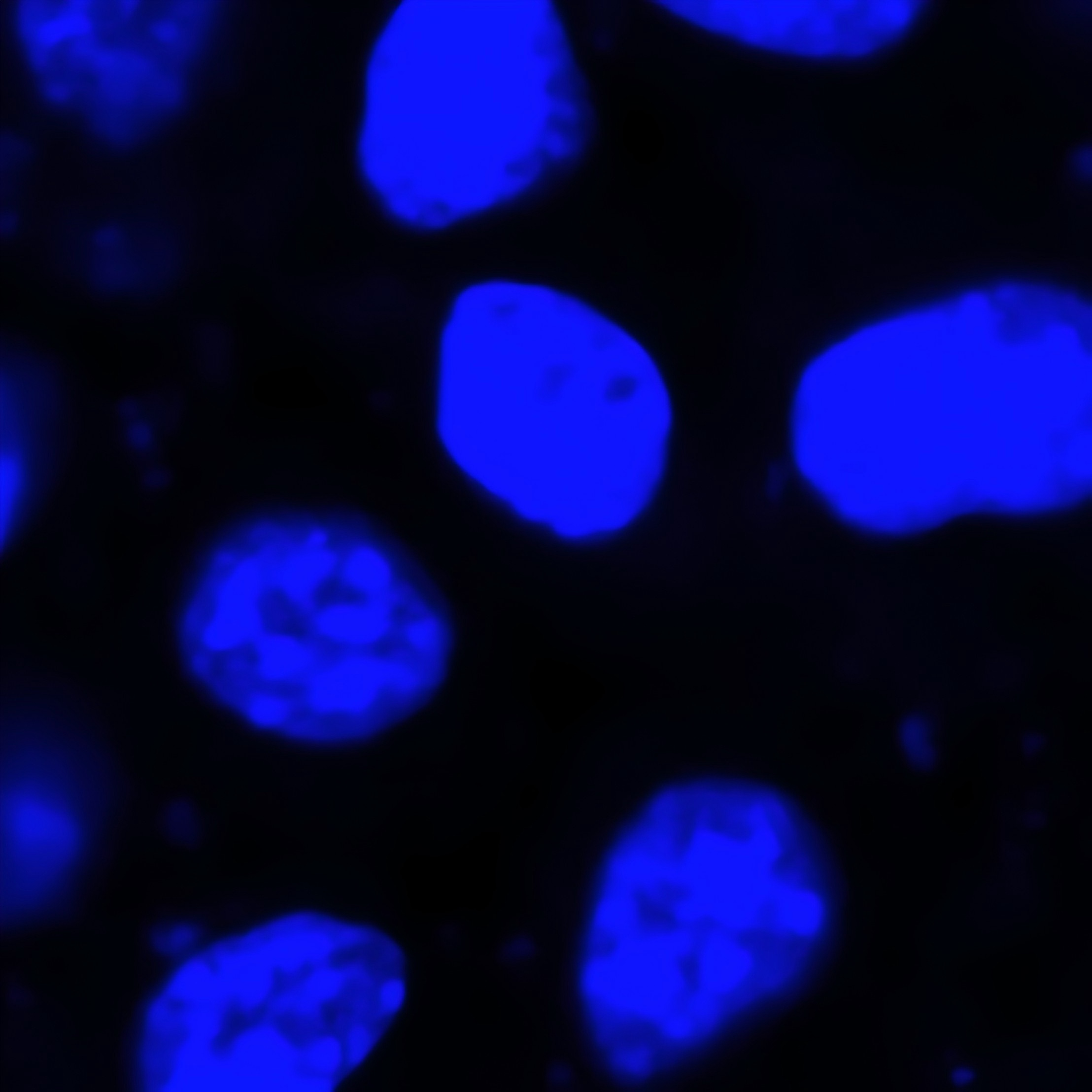

Supplement: Supplementary file 19 — Represent Raw Images [file 41419_2026_8682_MOESM19_ESM.zip › IFRAW/5-2-3.bmp]

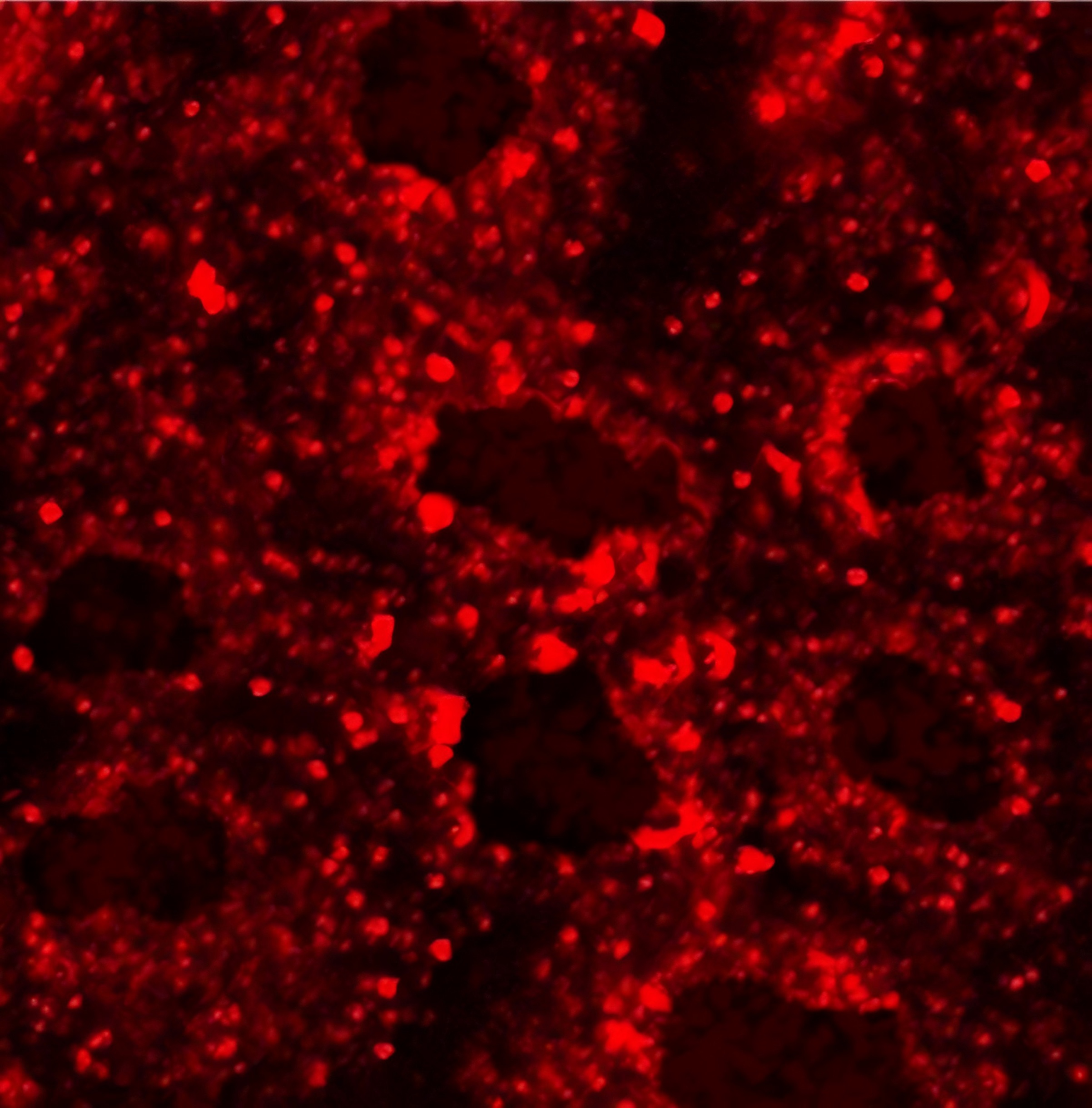

Supplement: Supplementary file 19 — Represent Raw Images [file 41419_2026_8682_MOESM19_ESM.zip › IFRAW/5-3-1.bmp]

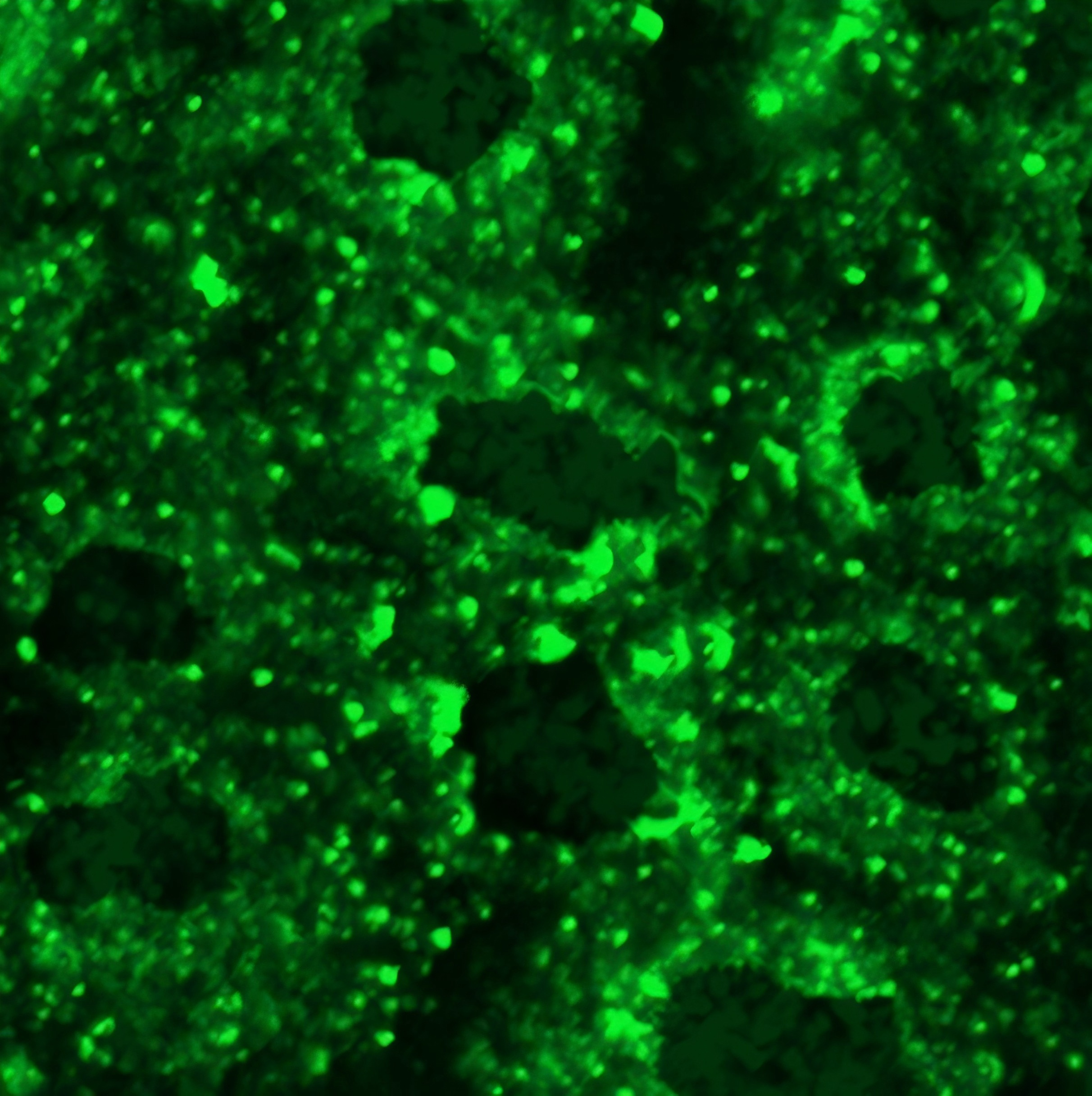

Supplement: Supplementary file 19 — Represent Raw Images [file 41419_2026_8682_MOESM19_ESM.zip › IFRAW/5-3-2.bmp]

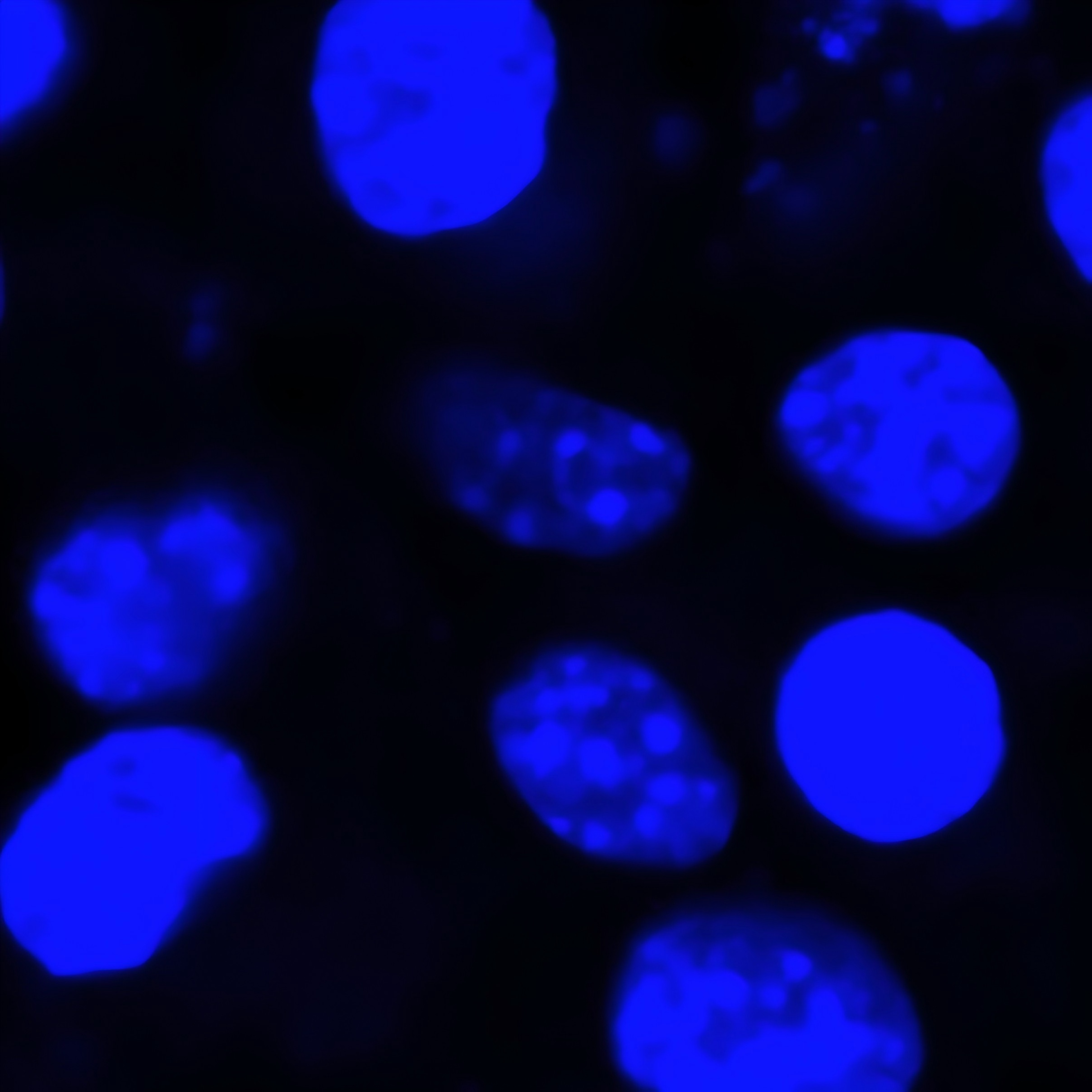

Supplement: Supplementary file 19 — Represent Raw Images [file 41419_2026_8682_MOESM19_ESM.zip › IFRAW/5-3-3.bmp]

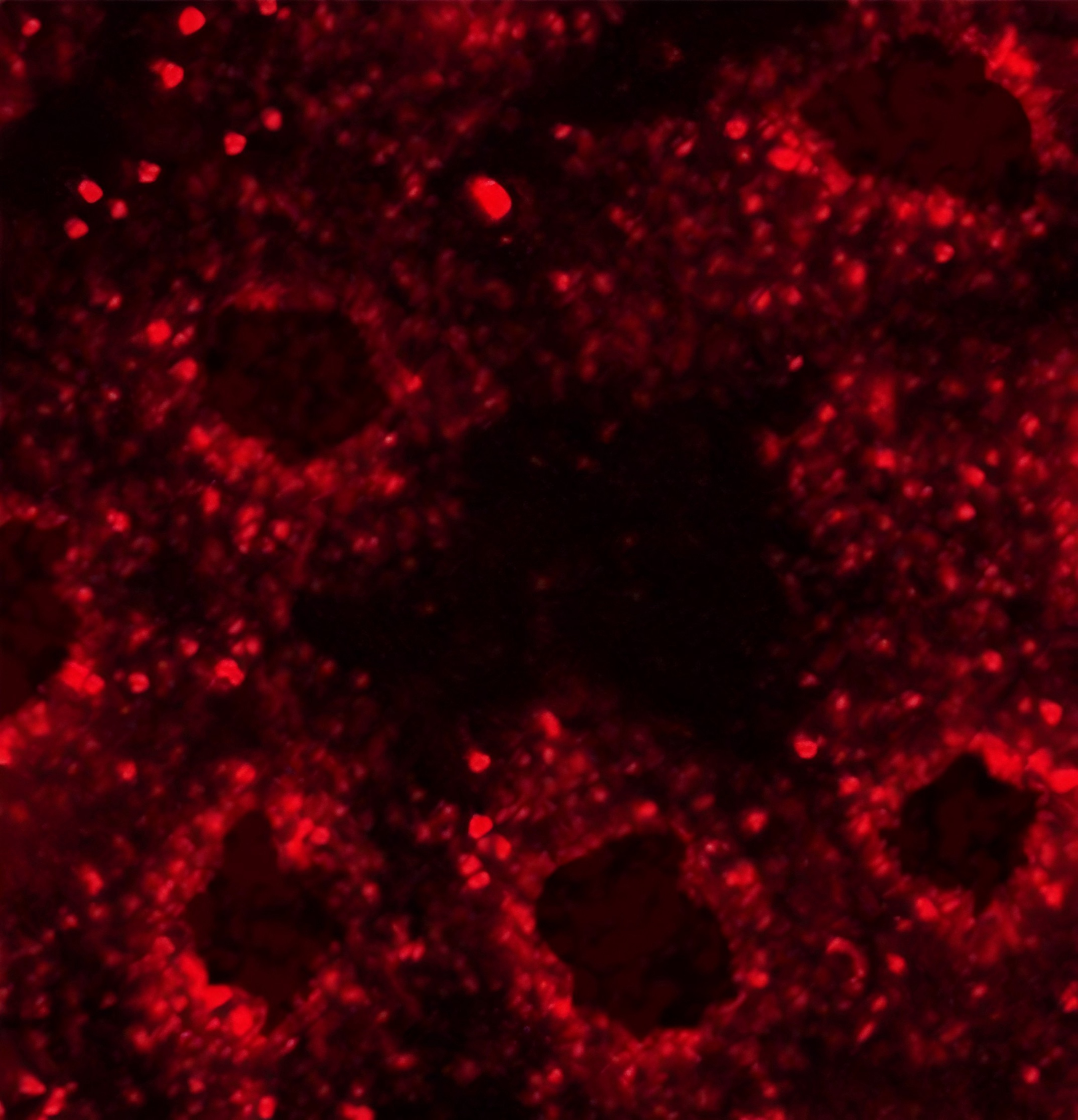

Supplement: Supplementary file 19 — Represent Raw Images [file 41419_2026_8682_MOESM19_ESM.zip › IFRAW/5-4-1.bmp]

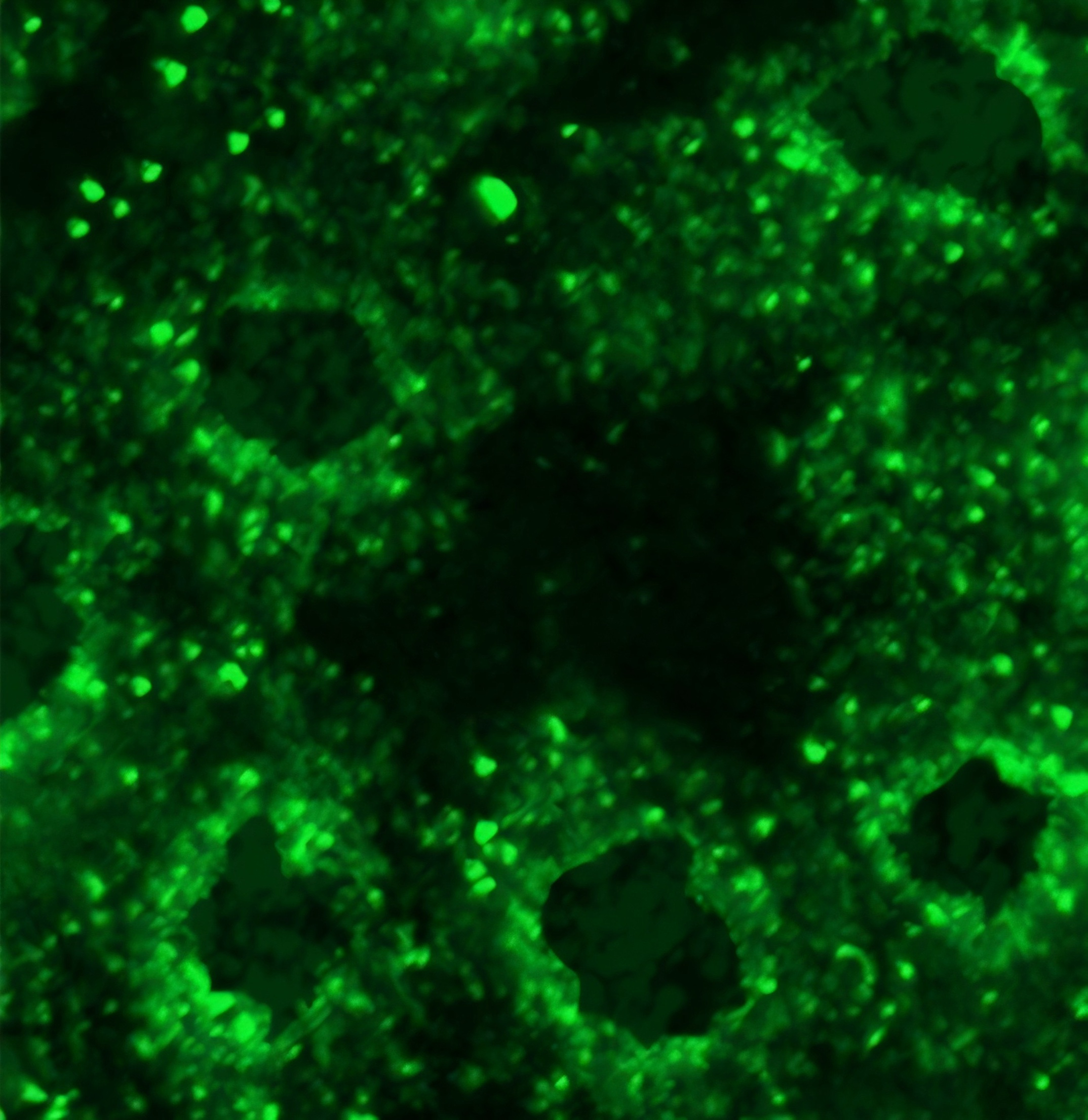

Supplement: Supplementary file 19 — Represent Raw Images [file 41419_2026_8682_MOESM19_ESM.zip › IFRAW/5-4-2.bmp]
