# Supplementary material for: Hepatocyte TIA1 constrains metabolic steatohepatitis by translationally suppressing Srebf1 mRNA in stress granules
Source: Cell Death Dis. 2026 Mar 24;17(1):357. doi: 10.1038/s41419-026-08682-5 (PMC13039281; doi:10.1038/s41419-026-08682-5)
Supplement: Supplementary file 11 — Table S1 [file 41419_2026_8682_MOESM11_ESM.docx]

**Table S1.** Clinical assessment of human subjects

| **Factors** | **Histological normal**  **Control (n=5)** | **MASLD (n=5)** | |
| --- | --- | --- | --- |
| **Clinical characteristics (mean ± SD)** | | | |
| Gender, n(M/F) | 3/2 | 4/1 | |
| Age, years(mean±SD) | 57.8±3.7 | 54.8±10.08 | |
| BMI,kg/m^2^ | 24.11±1.12 | 25.39±3.39 | |
| **Biochemical characteristics (mean ± SD)** | | | ***P* value** |
| ALT, U/L | 22.16±8.92 | 132.38±71.93 | 0.009 |
| AST, U/L | 35.38±33.65 | 80.4±11.48 | 0.022 |
| ALP, U/L | 84.96±21.69 | 134.1±99.48 | 0.312 |
| γ-GT,IU/L | 25.32±6.03 | 199.42±197.34 | 0.084 |
| LAP | 55.4±7.5 | 128±91.72 | 0.116 |
| SOD | 229±14.44 | 221.6±25.33 | 0.586 |
| Dierect Bilirubin, μmol/L | 4.52±1.93 | 10.6±6.95 | 0.096 |
| Total Bilirubin, μmol/L | 14.26±4.72 | 33.74±10.11 | 0.005 |
| **Histopathological characteristics of MASLD patients （n=5）** | | | |
| **Steatosis grade 0/1/2/3** | | 0/2/2/1 | |
| **Lobular inflammation grade 0/1/2/3** | | 0/3/2/0 | |
| **Hepatocellular ballooning 0/1/2** | | 0/2/3 | |
| **NAS score 0/1/2/3/4/5/6/7** | | 0/0/0/2/0/2/1 | |
| **Fibrosis stage 0/1/2/3/4** | | 0/3/2/0 | |
