# Supplementary material for: Hepatocyte TIA1 constrains metabolic steatohepatitis by translationally suppressing Srebf1 mRNA in stress granules
Source: Cell Death Dis. 2026 Mar 24;17(1):357. doi: 10.1038/s41419-026-08682-5 (PMC13039281; doi:10.1038/s41419-026-08682-5)
Supplement: Supplementary file 12 — Table S2 [file 41419_2026_8682_MOESM12_ESM.docx]

**Table S2.** Primary Antibodies Used for western blotting.

| **Antibody** | **Source** | **Vendor** | **Catlog no.** | **Dilution** |
| --- | --- | --- | --- | --- |
| SREBP1 | Rabbit | Abcam | ab313881 | 1:1000 |
| FASN | Rabbit | Proteintech | 10624-2-AP | 1:1000 |
| PPARγ | Rabbit | Proteintech | 16643-1-AP | 1:1000 |
| SCD1 | Rabbit | Proteintech | 28678-1-AP | 1:1000 |
| TGFβ1 | Rabbit | Proteintech | 81746-2-RR | 1:1000 |
| COL1A1 | Mouse | Abcam | ab6308 | 1:1000 |
| αSMA | Mouse | Abcam | ab7817 | 1:1000 |
| TIA1 | Rabbit | Proteintech | 12133-2-AP | 1:1000 |
| G3BP1 | Rabbit | Proteintech | 13057-2-AP | 1:1000 |
| TIAR | Rabbit | Proteintech | 17649-1-AP | 1:1000 |
| GAPDH | Mouse | Proteintech | 60004-1-Ig | 1:1000 |
