# Supplementary material for: Hepatocyte TIA1 constrains metabolic steatohepatitis by translationally suppressing Srebf1 mRNA in stress granules
Source: Cell Death Dis. 2026 Mar 24;17(1):357. doi: 10.1038/s41419-026-08682-5 (PMC13039281; doi:10.1038/s41419-026-08682-5)
Supplement: Supplementary file 13 — Table S3 [file 41419_2026_8682_MOESM13_ESM.docx]

**Table S3.** Primer sequences used for quantitative RT-PCR in this study.

| **Gene** | **Sequence (5'-3')** | |
| --- | --- | --- |
| Mouse *Tia1* | F: | GAGAAGGGCTATTCGTTT |
|  | R: | CCATACTGTTGTGGGTTT |
| Mouse *G3bp1* | F: | CAGACCAACCTGGAGAGCG |
|  | R: | GGCTTCACACTCGGCTTC |
| Mouse *Tial1* | F: | ACAGACATTCTCACCATTTGGAC |
|  | R: | GTAGTACCGTTCACCGAAACAAT |
| Mouse *Srebp1* | F: | GGCCGAGATGTGCGAACT |
|  | R: | TTGTTGATGAGCTGGAGCATGT |
| Mouse *Fasn* | F: | CTGCGGAAACTTCAGGAAATG |
|  | R: | GGTTCGGAATGCTATCCAGG |
| Mouse *Pparg* | F: | ATTCTGGCCCACCAACTTCGG |
|  | R: | TGGAAGCCTGATGCTTTATCCCCA |
| Mouse *Scd1* | F: | TTCTTGCGATACACTCTGGTGC |
|  | R: | CGGGATTGAATGTTCTTGTCGT |
| Mouse *Il6* | F: | TAGTCCTTCCTACCCCAATTTCC |
|  | R: | TTGGTCCTTAGCCACTCCTTC |
| Mouse *Il-1β* | F: | CCGTGGACCTTCCAGGATGA |
|  | R: | GGGAACGTCACACACCAGCA |
| Mouse *Tnfα* | F: | CATCTTCTCAAAATTCGAGTGACAA |
|  | R: | TGGGAGTAGACAAGGTACAACCC |
| Mouse *Tgfβ1* | F: | CTCCCGTGGCTTCTAGTGC |
|  | R: | GCCTTAGTTTGGACAGGATCTG |
| Mouse *Col1a1* | F: | TGCTAACGTGGTTCGTGACCGT |
|  | R: | ACATCTTGAGGTCGCGGCATGT |
| Mouse *Acta2* | F: | ACGTAAGCACTGGTGGACAG |
|  | R: | CCGGCTGGAAAGAAGTCTGA |
| Mouse *GAPDH* | F: | AGGTCATCCCAGAGCTGAACG |
|  | R: | ACCCTGTTGCTGTAGCCGTAT |
