# Supplementary material for: Hepatocyte TIA1 constrains metabolic steatohepatitis by translationally suppressing Srebf1 mRNA in stress granules
Source: Cell Death Dis. 2026 Mar 24;17(1):357. doi: 10.1038/s41419-026-08682-5 (PMC13039281; doi:10.1038/s41419-026-08682-5)
Supplement: Supplementary file 14 — Table S4 [file 41419_2026_8682_MOESM14_ESM.docx]

**Table S4.** Target sequences of shRNA.

| **Cat. Number** | **gene** | **Name in manuscript** | **Type** | **target region** | **target sequence**  **(5'-3')** |
| --- | --- | --- | --- | --- | --- |
| TRCN0000077158 | mouse TIA1 | shRNA-1 TIA1 | shRNA | CDS | CGATGGTGGATGTTTGCCAAT |
| TRCN0000077159 | mouse TIA1 | shRNA-2 TIA1 | shRNA | CDS | CGAAGACATCAAAGCAGCGTT |
| TRCN0000028944 | mouse G3BP1 | shRNA-1 G3BP1 | shRNA | CDS | GCCTGATGATTCTGGAACTTT |
| TRCN0000028945 | mouse G3BP1 | shRNA-2 G3BP1 | shRNA | CDS | CCTTAGTAATAGGCCCATCAT |
| TRCN0000102618 | mouse TIAL1 | shRNA-1 TIAL1 | shRNA | CDS | GTACACAAGAAACTAACACTA |
| TRCN0000102619 | mouse TIAL1 | shRNA-2 TIAL1 | shRNA | CDS | GCATACAAGCAATGACCCATA |
| SHC002 | Non-Targeting shRNA Controls | shRNA Control | shRNA | CDS | CAACAAGATGAAGAGCACCAA |
