# Supplementary material for: Hepatocyte TIA1 constrains metabolic steatohepatitis by translationally suppressing Srebf1 mRNA in stress granules
Source: Cell Death Dis. 2026 Mar 24;17(1):357. doi: 10.1038/s41419-026-08682-5 (PMC13039281; doi:10.1038/s41419-026-08682-5)
Supplement: Supplementary file 15 — Supplementary Materials and Methods [file 41419_2026_8682_MOESM15_ESM.docx]

**Supplementary Methods**

**RNA-Sequencing analysis**

**Sample processing and sequencing**

Total RNA was extracted from cryopreserved human liver biopsy samples (n = 5 per group: MASH vs. healthy controls) and mice liver samples (n = 3 per group: TIA1-HKO vs. TIA1-Flox) using TRIzol Reagent (Thermo Fisher, 15596026). RNA integrity was assessed using an Agilent Bioanalyzer 2100, and only samples with an RNA Integrity Number (RIN) ≥ 7.0 were used for subsequent analysis. Poly(A)+ mRNA was enriched and strand‑specific cDNA libraries were prepared using the TruSeq Stranded mRNA LT Kit (Illumina, RS‑122‑2103). Libraries were sequenced on an Illumina NovaSeq 6000 platform to generate 150 bp paired‑end reads, achieving a minimum depth of 40 million reads per sample (OE Biotech, Shanghai).

**Bioinformatics processing**

Raw sequencing reads were subjected to quality control using FastQC (v0.12.1). Adapters and low‑quality bases were trimmed with Trimmomatic (v0.38) using the following parameters: “SLIDINGWINDOW:30:15 AVGQUAL:15 LEADING:15 TRAILING:15 MINLEN:30 HEADCROP:5”. Cleaned reads were aligned to the human reference genome (GRCh38) using HISAT2 (v2.2.1) with default splice‑aware settings. Transcript abundance was quantified as Transcripts Per Million (TPM) using StringTie (v2.1.7).

**Differential expression and functional enrichment**

Differential gene expression analysis was performed using DESeq2 (v1.34.0) based on raw read counts. Genes with an adjusted p‑value (Benjamini‑Hochberg) < 0.05 and an absolute log₂ fold‑change ≥ 1 were considered significantly differentially expressed. Functional enrichment analysis of Gene Ontology (GO) terms and Kyoto Encyclopedia of Genes and Genomes (KEGG) pathways was conducted using the R package clusterProfiler (v4.4.2) with a hypergeometric test; results with a false‑discovery rate (FDR) < 0.05 were regarded as significant. All statistical results were visualized using ggplot2 (v3.3.5).

The raw RNA‑seq data have been deposited in the NCBI Sequence Read Archive (SRA) under BioProject accession number PRJNA1287246 (available at: <http://www.ncbi.nlm.nih.gov/bioproject/1287246/>) and PRJNA109228 (http:// www.ncbi.nlm.nih.gov/bioproject/1090228/).

.

**Biochemical measurements**

All serum and liver samples were collected and stored at -80 ℃ until detecting serum and hepatic levels of triglyceride (TG) and total cholesterol (TC), activity of the liver-associated enzymes alanine aminotransferase (ALT) and aspartate aminotransferase (AST), and levels of antioxidant and cytoprotective proteins, including superoxide dismutase (SOD) and glutathione (GSH). AML12 cells were processed and the supernatant was separated for the detection of Malondialdehyde (MDA), SOD, Interleukin-1β (IL-1β), Interleukin-6 (IL-6), Tumor necrosis factor-α (TNF-α). All the steps were performed according to the manufacturer′s instructions.were evaluated according to the manufacturer's recommendations (Nanjing Jiancheng Biological Technology, Inc., Nanjing, China).

**Histopathologic evaluation**
 Liver tissues were fixed in 10% neutral-buffered formalin for >24 h, embedded in paraffin, and sectioned at 5 μm thickness. Serial sections underwent hematoxylin and eosin (H&E) staining using standard protocols. NAFLD activity scoring (NAS) was performed by a blinded hepatopathologist following established criteria, where steatosis and lobular inflammation were graded 0-3, hepatocyte ballooning 0-2. Fibrosis extent was quantified via Masson's trichrome staining. Lipid accumulation was visualized by Oil Red O staining of optimal cutting temperature compound (OCT)-embedded cryosections. For immunohistochemistry (IHC), sections were treated with 3% H₂O₂ for 30 min at room temperature (RT) to inhibit endogenous peroxidase, followed by 1 hour blocking in 3% fatty acid-free bovine serum albumin (BSA). Primary antibody incubation employed anti-F4/80 (1:100, Proteintech, 29414-1-AP) or anti-α smooth muscle actin (αSMA, 1:100, Proteintech, 14395-1-AP) at 4 ℃ overnight. Horseradish peroxidase (HRP)-conjugated goat anti-rabbit or anti-mouse IgG secondary antibodies (Jackson Immunoresearch Inc., USA; 1:500), followed by incubation for 5 min with 3,3'-diaminobenzidine tetrahydrochloride chromogenic reagent (AR1022; Boster; Wuhan, China) to visualize specific staining using light microscopy. Sections were counterstained with hematoxylin. Quantification of positively stained areas was performed using ImageJ software (v1.53), excluding vascular and capsular regions.

**RIP analysis**

The interaction between TIA1 protein and target RNA was analyzed using the EZ-Magna RIP™ Kit (Millipore, 17-701). AML12 hepatocytes at approximately 90% confluency were lysed in complete RIP lysis buffer containing RNase and protease inhibitors. A 100 μL aliquot of whole-cell extract was incubated with magnetic beads conjugated to either anti-TIA1 antibody (1:100; Proteintech, 12133-2-AP) or normal rabbit IgG (Cell Signaling Technology, 2729) as a negative control for 2 hours at 4 °C with gentle rotation.

After extensive washing with RIP wash buffer, the immunoprecipitated RNA-protein complexes were treated with proteinase K to digest the protein. RNA was then extracted using phenol:chloroform:isoamyl alcohol (25:24:1), precipitated with ethanol, and dissolved in RNase-free water.

The purified RNA was reverse-transcribed using a PrimeScript RT Reagent Kit (Takara). Enrichment of specific RNA transcripts in the TIA1 immunoprecipitate was quantified by qRT-PCR using SYBR Premix Ex Taq™ II (Takara) and normalized to the input RNA sample. Each RIP experiment was performed with at least three independent biological replicates, and the results are presented as fold enrichment relative to the IgG control.

**RNA interference and knockdown in AML12 hepatocytes**

Short hairpin RNA (shRNA) sequences targeting mouse *Tia1*(encoding TIA1), *G3bp1* (encoding G3BP1), and *Tial1* (encoding TIAR) were designed and cloned into the lentiviral vector pLKO.1-puro (Shanghai Genechem). A non-targeting scrambled shRNA was used as a negative control. For lentivirus production, HEK293T cells were co-transfected with the shRNA expression plasmid along with the packaging plasmids psPAX2 and pMD2.G using Lipofectamine 3000 (Invitrogen). Virus-containing supernatants were collected 48 and 72 hours post-transfection, filtered through a 0.45 μm sterile filter, and concentrated.

For transduction, AML12 hepatocytes were seeded in 6-well plates at a density of 2 × 10⁵ cells/well and cultured in complete DMEM/F12 medium overnight to reach ~70% confluency. Cells were then transduced with the concentrated lentivirus at a multiplicity of infection (MOI) of 20 in the presence of 6 μg/mL polybrene. After 24 hours, the medium was replaced with fresh culture medium. To establish stable knockdown cell lines, cells were selected with 2 μg/mL puromycin for 7-10 days, or until all cells in the negative control wells were eliminated.

Knockdown efficiency was validated by qRT-PCR and western blot analyses. qRT-PCR analysis confirmed that mRNA levels of *Tia1*,*G3bp1*, and *Tial1* were reduced by at least 75% compared to the scrambled shRNA control. Western blotting was performed using the following primary antibodies: anti-TIA1 (1:1000; Proteintech 12133-2-AP), anti-G3BP1 (1:1000; Proteintech 13057-2-AP), and anti-TIAR (1:1000; Proteintech 17649-1-AP). GAPDH was used as a loading control (1:1000; Proteintech 60004-1-Ig). All shRNA sequences are provided in Supplementary Table S4.

**Adenovirus-mediated gene overexpression in AML12 hepatocytes**

For gene overexpression, AML12 hepatocytes were seeded at a density of 6 × 10⁴ cells/well in collagen I-coated 12-well plates (Corning 354408) and allowed to adhere overnight (~60-70% confluency). Cells were transduced with replication-deficient adenovirus serotype 5 (Ad5) vectors expressing mouse *Tia1* (VectorBuilder, VB900217-8714bnx), *G3bp1* (VectorBuilder, VB900169-6914kuq), *Tial1* (VectorBuilder, VB900217-8713msr), or green fluorescent protein (VectorBuilder, VB900000-0001) under the control of a CMV promoter.

Viral particles were diluted in serum-free DMEM/F12 medium to achieve a multiplicity of infection (MOI) of 50, as determined by preliminary dose-response experiments. After 6 hours of incubation at 37 °C, the virus-containing medium was replaced with complete growth medium. Transduction efficiency was assessed 24 hours post-infection either by quantifying adenoviral genomic copies using qRT-PCR targeting the E1A region (for all viruses) or by direct fluorescence microscopy (for Ad-GFP). Protein overexpression was confirmed 72 hours post-transduction by western blot analysis using the following antibodies: anti-TIA1 (1:1000; Abcam ab263945), anti-G3BP1 (1:1000; Proteintech 13057-2-AP), anti-TIAR (for TIAL1; 1:1000; Proteintech 17649-1-AP), and anti-GAPDH (1:1000; Proteintech 60004-1-I) as a loading control.

**Cellular Oil Red O staining**

Following experimental treatments, AML12 cells were washed three times with phosphate-buffered saline (PBS) and fixed with 4% paraformaldehyde (PFA) for 15 min at room temperature. Cells were then washed with PBS and stained with freshly prepared Oil Red O working solution for 15 min. The working solution was prepared by mixing saturated Oil Red O in isopropanol with deionized water at a 3:2 ratio, followed by centrifugation at 12,000 rpm for 5 min to remove precipitates, and the clear supernatant was used for staining.

Following staining, cells were washed three times with deionized water to remove unbound dye, and then briefly differentiated with 60% isopropanol to reduce background. Nuclei were counterstained with hematoxylin for 1 min, followed by three washes with deionized water.

Lipid droplet visualization employed an inverted microscope (Olympus) at 400× magnification. Quantification of Oil Red O-positive areas was performed using standardized thresholding in ImageJ software (v1.53).

**Intracellular ROS measurement**

Intracellular ROS levels were measured using the fluorogenic probe 2′,7′-dichlorodihydrofluorescein diacetate (DCFH-DA; R252, Dojindo). AML12 hepatocytes were seeded in 6-well plates at a density of 1 × 10⁵ cells/well and treated as indicated. After 24 h of treatment, the culture medium was removed and cells were incubated with 10 µM DCFH-DA in serum-free medium for 20 min at 37 °C in the dark. Cells were then washed three times with pre-warmed PBS to remove excess probe. Fluorescent images were acquired immediately using a confocal laser scanning microscope (TCS SP8; Leica; Wetzler, Germany) with excitation/emission set at 488/525 nm. For quantitative analysis, fluorescence intensity was determined in three randomly selected fields per well using ImageJ software (version 1.53).

**Protein isolation and western blot analysis**

Liver tissues and AML12 cells were homogenized in Radioimmunoprecipitation assay (RIPA) lysis buffer containing protease inhibitor cocktail and phosphatase inhibitor cocktail (Beyotime Institute of Biotechnology, Shanghai, China). Lysates were centrifuged at 12,000 rpm (4 ℃, 10 min), with protein concentrations determined by a modified bicinchoninic acid protein concentration assay kit (Beyotime Institute of Biotechnology, Shanghai, China). Samples (20 μg/lane) underwent SDS-PAGE separation on 6-12% gels and transferred to polyvinylidene membranes (IPVH00010; Millipore, Billerica, MA, USA). Membranes were blocked for 2 hours at RT in 5% skim milk dissolved in Tris-buffered saline with Tween 20 (TBST; 100 mM Tris-HCl, pH 7.5, 0.9% NaCl, 0.1% Tween 20), followed by overnight incubation at 4 ℃ with primary antibodies (1:1000 in blocking buffer) under constant agitation. After three TBST washes, membranes were incubated for 1 hour at RT with HRP-conjugated secondary goat anti-rabbit antibody (1:2000; Beyotime A0208) or goat anti-mouse antibody(1:2000; Beyotime A0216). Protein bands were visualized using BeyoECL Plus (Cat #P0018, Beyotime) and imaged on a Tanon 5200 machine (Tanon Science and Technology Co., Ltd., Shanghai, China). Band intensities were quantified using ImageJ software (version 1.53) and normalized to glyceraldehyde‑3‑phosphate dehydrogenase (GAPDH) as loading control. Primary and secondary antibodies are cataloged in the Supplementary Table S2.

**Quantitative real-time polymerase chain reaction (qRT-PCR)**

In short, total RNA from the liver was extracted using RNAisoPlus® reagent (Takara Biotech, Tokyo, Japan) according to the manufacturer's instructions. cDNA was produced using PrimeScript® RT Reagent Kit with gDNA Eraser (Takara Biotech, Tokyo, Japan) and was amplified using SYBR Premix Ex TaqTM Kit (Takara Biotech, Tokyo, Japan). Real-time PCR for quantitative assessment of mRNA expression was performed using an ABI Prism 7900HT Fast Real-Time PCR System (Applied Biosystems, Tokyo, Japan), according to the manufacturer's instruction. Relative expression levels (defined as fold change) of each target gene were normalized to that of GAPDH mRNA levels in each sample using the 2^-ΔΔCT^ method. The primers for targeted genes are listed in Supplementary Table S3.

**Luciferase reporter assays**

AML12 hepatocytes were seeded in 24-well plates at a density of 8 × 10⁴ cells/well and allowed to adhere overnight (~70% confluency). For each well, cells were co-transfected with 400 ng of a firefly luciferase reporter construct (pGL4.10 backbone) harboring either the wild-type or mutant *Srebf1* promoter sequence, together with 100 ng of a TIA1 expression plasmid $pcDNA3.1+$ or the corresponding empty vector control. Transfection was performed using Lipofectamine 3000 (Thermo Fisher, L3000015) at a ratio of 2 µL reagent per 1 µg of total DNA. To normalize for transfection efficiency, 10 ng of a Renilla luciferase control plasmid (pRL-TK; Promega) was included in each transfection mixture.

After 24 hours of incubation, cells were lysed with 1× Passive Lysis Buffer (Promega, E1941) for 15 minutes at room temperature with gentle agitation. Luciferase activity was measured using the Dual-Glo Luciferase Assay System (Promega, E1910) on a GloMax 20/20 Luminometer (Promega). Firefly luciferase signal was measured first, followed by quenching and measurement of the Renilla luciferase signal. The relative promoter activity for each sample was calculated as the ratio of firefly luciferase luminescence to Renilla luciferase luminescence. All experiments were performed with at least three independent biological replicates, each containing three technical replicates.

**mRNA stability assays**

The stability of *Srebf1* mRNA was assessed in AML12 hepatocytes following gene manipulation. Cells were transduced with either Ad-TIA1 or TIA1-targeting shRNA (as described in Sections 2.6 and 2.7) for 24 hours prior to the assay. To monitor mRNA decay, transcription was globally inhibited by adding 5 µg/mL actinomycin D (Sigma-Aldrich, A9415). Cells were harvested at defined time points (T = 0, 2, 4, 6, and 8 hours post-inhibition), and total RNA was extracted using TRIzol reagent (Thermo Fisher, 15596026).

cDNA was synthesized from 1 µg of total RNA, and the abundance of *Srebf1* transcripts was quantified by qRT-PCR using gene-specific primers (Forward: 5′-GGCCGAGATGTGCGAACT-3′; Reverse: 5′-TTGTTGATGAGCTGGAGCATGT′). mRNA levels were normalized to the housekeeping gene Gapdh, and the relative quantity at each time point was expressed as a percentage of the level at T=0.

The mRNA decay rate constant (Kdecay) was determined by fitting the natural logarithm of the relative mRNA quantity versus time to a first-order kinetic model: ln(C/C₀) = -K_decay_ · t, where t represents the time of transcription inhibition (hours), and C and C₀ represent mRNA quantities at time t and time zero, respectively. The mRNA half-life (t₁/₂) was then calculated as t₁/₂ = ln(2) / Kdecay. Curve fitting and parameter estimation were performed using GraphPad Prism software (version 9.0). Each experiment included three biological replicates, and data are presented as mean ± SEM.

**RNAscope multiplex fluorescent assay with immunofluorescence**

Fixed cellular specimens underwent RNAscope® HiPlex analysis (Advanced Cell Diagnostics) using *Srebf1* mRNA-specific probes (Probe-Mm-Srebf1; ACD Bio 538181) following the manufacturer's SweAMI protocol. Sections were pre-hybridized at 40 ℃ for 1 hour, followed by overnight hybridization at 40 ℃ in HybEZ™ oven. Stringent washes employed SSC buffers at increasing stringency (0.5×→0.1×). Signal amplification utilized branch DNA technology with sequential AMP probes, culminating in channel-specific fluorophore conjugation. For dual detection, immunofluorescence was performed post-RNAscope: sections were blocked with 3% BSA/PBS and incubated overnight at 4 ℃ with anti-TIA1 antibody (1:200; Servicebio GB113725), then with Alexa Fluor® 647-conjugated secondary antibodies (1:500; Abcam ab150083) for 2 hours at RT. Nuclei were counterstained with DAPI (1 μg/mL; Sigma D9542). Z-stack images (0.3 μm steps) were acquired using Nikon A1R confocal microscope with 60× oil immersion objective (NA 1.4) and NIS-Elements software (v5.30).
