# Supplementary material for: Hepatocyte TIA1 constrains metabolic steatohepatitis by translationally suppressing Srebf1 mRNA in stress granules
Source: Cell Death Dis. 2026 Mar 24;17(1):357. doi: 10.1038/s41419-026-08682-5 (PMC13039281; doi:10.1038/s41419-026-08682-5)
Supplement: Supplementary file 16 — Supplementary Figure Legends [file 41419_2026_8682_MOESM16_ESM.docx]

**Legends of Supplementary Figures**

**Fig.S1.** Clinical characteristics of TIA1 expression based on TCGA database. (A) Kaplan-Meier survival curves of HCC patients stratified by TIA1 expression levels (high vs. low) based on the Kaplan-Meier plotter database ([https://kmplot.com](https://kmplot.com/)). Analyses incorporate Overall Survival (OS), Recurrence-Free Survival (RFS), Progression-Free Survival (PFS), and Disease-Specific Survival (DSS). Lower TIA1 expression is significantly associated with worse survival outcomes (Log-rank test, P < 0.05). (B, C) Correlation analysis of TIA1 mRNA expression (from TCGA database) with clinical-pathological parameters in HCC patients. (B) Box plots show significantly lower TIA1 expression in advanced pathological stages (III & IV compared to I & II) and in patients with serum AFP levels >400 ng/mL. (C) Forest plot showing univariate logistic regression analysis of the association between TIA1 mRNA expression (dichotomized as high/low) and various clinicopathological characteristics in HCC patients from the TCGA cohort. Odds ratios (OR) with 95% confidence intervals are displayed for each variable. (D) Profile intensity showing TIA1/DAPI fluorescence signals of the images in Figure 1G. *P < 0.05, **P < 0.01, ***P < 0.001; ****P < 0.0001; ns indicates not significant.

# **Fig.S2.** Validation of hepatocyte-specific TIA1 knockout and comprehensive phenotypic characterization under normal and HFD conditions. (A) Western blot analysis confirming tissue-specific deletion of TIA1 protein in liver tissues, but not in skeletal muscle (SKM) or inguinal white adipose tissue (iWAT), of 8-week-old TIA1-HKO mice compared to TIA1-Flox controls (1 technical replicate of 3 biological replicates for each group). GAPDH served as a loading control. (B) qRT-PCR analysis of *Tia1* mRNA expression normalized to *Gapdh*. (n = 3 independent biological replicates per group; mean ± SEM). (C) Body weight of mice in the indicated groups throughout the 12-week HFD or CD feeding period (n=10 mice per group; mean ± SEM). (D, E) Metabolic parameters including average daily food intake (measured over 3 consecutive days) and liver-to-body weight ratio (%) of TIA1-FLox and TIA1-HKO mice fed CD or HFD diet for 12 weeks (n=10 per group; mean ± SEM). (F) Representative gross liver morphology and photomicrographs of liver sections from 12-week-old TIA1-Flox and TIA1-HKO mice under CD condition, stained with H&E, ORO, F4/80, and SMA. Scale bar: 100 μm. (G) NAFLD Activity Score (NAS) and its individual components (steatosis, lobular inflammation and hepatocyte ballooning) based on histopathological assessment of H&E-stained liver sections (mean of 5 fields per section, 3 sections per mouse). (n=10 per group; mean ± SEM). (H) Quantification of ORO-, F4/80-, and αSMA- positive areas. ImageJ software was used for quantification of stained areas (mean of 5 fields per section, 3 sections per mouse). (n=10 per group; mean ± SEM). (I) Serum TC and TG levels in the indicated groups (n=10 per group; mean ± SEM). (J) Densitometric quantification of western blot analysis showing protein levels of key regulators of fatty acid metabolism (FASN, SREBP1, SCD1, PPARγ) in liver lysates. GAPDH served as loading control (n = 3 independent biological replicates per group; mean ± SEM). *P < 0.05, **P < 0.01, ***P < 0.001; ****P < 0.0001; ns indicates not significant.

# **Fig.S3.** Comprehensive phenotypic analysis of TIA1-HKO mice in the HFHC diet-induced MASH Model. (A) Body weight of mice in the indicated groups over the 20-week HFHC diet or CD feeding period (n=10 mice per group; mean ± SEM). (B, C) Metabolic parameters including average daily food intake (measured over 3 consecutive days) and liver-to-body weight ratio (%) (n=10 per group; mean ± SEM). (D) Representative gross liver morphology and photomicrographs of liver sections from 20-week-old TIA1-Flox and TIA1-HKO mice under CD condition, stained with H&E, ORO, F4/80, Masson's Trichrome, and αSMA. Scale bar: 100 μm. (E) NAS and its individual components based on histopathological assessment of H&E-stained liver sections (mean of 5 fields per section, 3 sections per mouse). (n=10 per group; mean ± SEM). (F) Quantification of ORO-, F4/80-, Masson's Trichrome- and αSMA-positive areas. ImageJ software was used for quantification of stained areas (mean of 5 fields per section, 3 sections per mouse). (n=10 per group; mean ± SEM). (G) Serum TC and TG levels, and hepatic GSH and SOD levels (n=10 per group; mean ± SEM). (H) Densitometric quantification of Western blot analysis showing protein levels of key regulators of fatty acid metabolism (FASN, SREBP1, SCD1, PPARγ) and fibrogenesis (COL1A1, TGF-β1, αSMA) in liver lysates. GAPDH served as the loading control (n = 3 independent biological replicates per group; mean ± SEM). *P < 0.05, **P < 0.01, ***P < 0.001; ****P < 0.0001; ns indicates not significant.

# **Fig.S4.** Hepatocyte-specific TIA1 knockout exacerbates MCD-induced liver Injury, fibrosis, and inflammation. (A) Body weight of mice in the indicated groups throughout the 8-week MCD or CD feeding period (n=10 mice per group; mean ± SEM). (B, C) Metabolic parameters including average daily food intake (measured over 3 consecutive days) and liver-to-body weight ratio (%) (n=10 per group; mean ± SEM). (D) Representative gross liver morphology and photomicrographs of liver sections from 8-week-old TIA1-Flox and TIA1-HKO mice under CD condition, stained with H&E, Masson's Trichrome, F4/80, and αSMA. Scale bar: 100 μm. (E) NAS and its individual components based on histopathological assessment of H&E-stained liver sections (mean of 5 fields per section, 3 sections per mouse). (n=10 mice per group; mean ± SEM). (F) Quantification of Masson's Trichrome-, F4/80- , and αSMA-positive areas. ImageJ software was used for quantification of stained areas (mean of 5 fields per section, 3 sections per mouse). (n=10 per group; mean ± SEM). (G) Serum levels of ALT and AST, and hepatic levels of GSH and SOD (n=10 per group; mean ± SEM). (H) Western blot analysis of proteins involved in fibrogenesis (COL1A1, TGF-β1, αSMA). GAPDH served as a loading control. (I) Densitometric quantification of western blotting results shown in (H). (n= 3 biologically independent mice per condition; mean ± SEM). (J) qRT-PCR analysis of genes related to fibrogenesis (*Col1a1, Tgfβ1* and *Acta2*), and inflammation (*Il1β, Il6* and*Tnfα*), normalized to *Gapdh*.  (n= 5 biologically independent mice per condition; mean ± SEM). *P < 0.05, **P < 0.01, ***P < 0.001; ****P < 0.0001; ns indicates not significant.

# **Fig.S5.** Effects of AAV-mediated TIA1 overexpression on metabolic and histopathological phenotypes in HFD-fed mice. (A) Body weights of TIA1-Flox and TIA1-HKO mice fed treated with AAV-TIA1 or AAV-GFP throughout the 12-week HFD or CD feeding period (n=10 mice per group; mean ± SEM). (B) NAS and its individual components based on histopathological assessment of H&E-stained liver sections (mean of 5 fields per section, 3 sections per mouse). (n=10 per group; mean ± SEM). (C) Quantification of ORO-, F4/80-, and αSMA- positive areas. ImageJ software was used for quantification of stained areas (mean of 5 fields per section, 3 sections per mouse). (n=10 per group; mean ± SEM). (D) Serum total cholesterol (TC) and triglyceride (TG) levels, and hepatic glutathione (GSH) and superoxide dismutase (SOD) levels (n=10 per group; mean ± SEM). *P < 0.05, **P < 0.01, ***P < 0.001; ****P < 0.0001; ns indicates not significant.

**Fig.S6.** **Modulation of TIA1 expression and stress granule formation by fatty acids and chemical inducers' impact on lipid accumulation. (A)** Effect of different fatty acid treatments on TIA1 expression. AML12 hepatocytes were treated with BSA (control), PA (400 μM), a combination of PA and oleic acid (PA+OA, 200 μM each), or OA alone (400 μM). Left panel: Western blot analysis of TIA1 protein levels (1 technical replicate of 3 biological replicates for each group). GAPDH served as a loading control. Right panel: qRT-PCR analysis of *Tia1* mRNA expression normalized to *Gapdh* (n = 5 independent biological replicates; mean ± SEM). **(B, D)** Representative immunofluorescence images of AML12 cells in the indicated groups, co-stained for TIA1 protein (green) and the core SGs marker G3BP1 (red). Nuclei were counterstained with DAPI (blue). White boxes in indicate regions shown representative SGs exhibiting co-localization. Scale bar: 10 µm. **(C, E)** Quantification of SGs formation from experiments in (B&D). Left panel: Percentage of cells containing SGs (n=50 cells per group). Right panel: Average number of TIA1 G3BP1 double positive SGs per cell (n=50 views per group). **(F)** Assessment of lipid accumulation via BODIPY staining and the normalized quantification (n = 5 independent biological replicates; mean ± SEM). AML12 cells were pre-treated with anisomycin (An, 25 ng/mL for 30 min) or sodium arsenite (Ars, 0.5 mM for 1 hour) prior to PA challenge (0.3 mM for 24 hours). Representative BODIPY fluorescence (red) images show neutral lipid content. Nuclei were counterstained with DAPI (blue). Scale bar: 50 µm. *P < 0.05, **P < 0.01, ***P < 0.001; ****P < 0.0001; ns indicates not significant.

**Fig.S7.** The role of TIA1 and related RNA-binding proteins in regulating lipid metabolism in hepatocytes. (A, B) Validation of TIA1 manipulation efficiency. AML12 cells were transfected with adenovirus overexpressing TIA1 (Ad-TIA1) or GFP control (Ad-GFP), or with shRNA targeting TIA1 (shTIA1) or negative control shRNA (shNC). Left panel: Western blot analysis of TIA1 protein levels following knockdown or overexpression (1 technical replicate of 3 biological replicates for each group). GAPDH served as a loading control. Right panel: qRT-PCR analysis of *Tia1* mRNA levels following corresponding manipulations normalized to *Gapdh* (n = 5 independent biological replicates; mean ± SEM). (C) Representative images of BODIPY staining and the normalized quantification (n = 5 independent biological replicates; mean ± SEM) in AML12 cells transfected with Ad-TIA1 or Ad-GFP, or with shTIA1 or shNC. Representative BODIPY fluorescence (red) images show neutral lipid content. Nuclei were counterstained with DAPI (blue). Scale bar: 50 µm. (D) Analysis of G3BP1 and TIAR expression upon TIA1 knockdown under PA stress. Left panel: Western blot analysis of G3BP1 and TIAR protein levels in AML12 cells treated with BSA (control), PA, or PA + shTIA1 (1 technical replicate of 3 biological replicates for each group). Right panel: qRT-PCR analysis of*G3bp1* and*Tial1* mRNA levels under the same conditions, normalized to *Gapdh* (n = 5 independent biological replicates; mean ± SEM). (E, F) Validation of G3BP1 manipulation efficiency. AML12 cells were transfected with adenovirus overexpressing G3BP1 (Ad-G3BP1) or GFP control (Ad-GFP), or with shRNA targeting G3BP1 (shG3BP1) or negative control shRNA (shNC). Left panel: Western blot analysis of G3BP1 protein levels following knockdown or overexpression (1 technical replicate of 3 biological replicates for each group). GAPDH served as a loading control. Right panel: qRT-PCR analysis of *G3bp1* mRNA levels following corresponding manipulations normalized to*Gapdh* (n = 5 independent biological replicates; mean ± SEM). (G, H) Validation of TIAL1 manipulation efficiency. AML12 cells were transfected with adenovirus overexpressing TIAL1 (Ad-TIAL1) or GFP control (Ad-GFP), or with shRNA targeting TIAL1 (shTIAL1) or negative control shRNA (shNC). Left panel: Western blot analysis of TIAR protein levels following knockdown or overexpression (1 technical replicate of 3 biological replicates for each group). GAPDH served as a loading control. Right panel: qRT-PCR analysis of *Tial1* mRNA levels following corresponding manipulations normalized to*Gapdh* (n = 5 independent biological replicates; mean ± SEM). (I, J) Functional analysis of G3BP1 in lipid metabolism. (I) Representative ORO staining and quantification in AML12 cells transfected with Ad-G3BP1 or Ad-GFP, or with shRNA targeting G3BP1 or negative control (n = 5 independent biological replicates; mean ± SEM). Scale bar: 50 μm. (J) qRT-PCR analysis of mRNA expression levels of genes related to lipid metabolism (*Fasn, Srebf1, Scd1* and *Pparg*), normalized to *Gapdh*. (n = 5 independent biological replicates; mean ± SEM). (K, L) Functional analysis of TIAR in lipid metabolism. (K) Representative ORO staining and quantification in AML12 cells transfected with Ad-TIAL1 or Ad-GFP, or with shRNA targeting TIAL1 or negative control (n = 5 independent biological replicates; mean ± SEM). Scale bar: 50 μm. (L) qRT-PCR analysis of mRNA expression levels of genes related to lipid metabolism (*Fasn, Srebf1, Scd1* and *Pparg*), normalized to *Gapdh*. (n = 5 independent biological replicates; mean ± SEM). *P < 0.05, **P < 0.01, ***P < 0.001; ****P < 0.0001; ns indicates not significant.

**Fig.S8.** Assessment of oxidative stress and inflammation in TIA1-modulated hepatocytes under metabolic stress. (A, B) Densitometric quantification of protein levels from Figure 5 (n = 3 independent biological replicates; mean ± SEM). (C, E) Representative fluorescence microscopy images visualizing intracellular reactive oxygen species (ROS) levels in AML12 hepatocytes under indicated experimental conditions and the normalized quantification (n = 5 independent biological replicates; mean ± SEM). Scale bar: 50 μm. (D, F) Corresponding quantitative analyses of oxidative stress and inflammatory markers in cell lysates or culture supernatants. (D) Oxidative stress markers: Malondialdehyde (MDA) content (lipid peroxidation) and superoxide dismutase (SOD) activity. (n = 3 independent biological replicates; mean ± SEM). (F) Inflammatory cytokines: Levels of IL-1β, IL-6, and TNF-α measured by ELISA. (n = 3 independent biological replicates; mean ± SEM). *P < 0.05, **P < 0.01, ***P < 0.001; ****P < 0.0001; ns indicates not significant.

**Fig.S9.** TIA1-RNA binding characteristics in hepatocytes under metabolic stress. (A, B) Identification of differential TIA1-bound RNAs by RIP-seq. AML12 hepatocytes were treated with palmitic acid (PA) or BSA (control). (A) MA plot visualizing the enrichment of transcripts in TIA1 RIP-seq samples (PA-treatment versus BSA-treatment). The x-axis represents the average expression level (Log₁₀ of mean normalized counts). The y-axis shows the magnitude of enrichment (Log_10_ fold change, RIP/Input). (B) Volcano plot highlighting RNAs with statistically significant differences in TIA1 binding between PA and BSA conditions. Dashed lines indicate thresholds for significance (adjusted p < 0.05) and fold-change (|log2FC| > 1). (C) Motif analysis results showing the differential enriched motifs from TIA1-bound peaks of *Srebf1* mRNA from the two biological replicates (IP1 and IP2). Binding motifs were ranked by p-value, and the statistical results including the percentage of targets and, the background are shown. (D, E) Genomic distribution of TIA1 binding sites. (D) Bar chart showing the distribution of all TIA1 binding peaks across the chromosomes in PA-treated AML12 cells. Data are integrated from the ENCODE eCLIP database. (E) A detailed snapshot of the distribution of 119 high-confidence TIA1 binding peaks along chromosome 17, with the Srebf1 gene locus highlighted. Data are integrated from the ENCODE eCLIP database. (F) Integrative Genomics Viewer (IGV) screenshot validating the binding of TIA1 to the Srebf1 genomic locus. The top track (light blue) shows TIA1 eCLIP signal from the ENCODE database, and the bottom track (gray) indicates the gene model of *Srebf1*, confirming the direct interaction in a publicly available dataset. (G) Profile intensity showing TIA1/*Srebf1* fluorescence signals of the images in Figure 6F. (G, H) Densitometric quantification of protein levels from Figure 6 (n = 3 independent biological replicates; mean ± SEM). *P < 0.05, **P < 0.01, ***P < 0.001; ****P < 0.0001; ns indicates not significant.

**Fig.S10.** Pharmacological inhibition of SREBP partially rescues metabolic and histopathological phenotypes in HFD-fed TIA1-HKO mice. (A) Representative immunofluorescence staining images of TIA1 (red) and G3BP1 (green) of the liver sections of the mice fed with CD and HFD, in which nuclei were stained with DAPI (blue). Scale bar: 100 μm. Profile intensity showing TIA1/G3BP1/DAPI fluorescence signals of the images in (A). (B) Body weight of TIA1-Flox and TIA1-HKO mice treated with or without the SREBP inhibitor PF-429242 (20 mg/kg/day, i.p.) during a 12-week HFD or CD feeding period (n = 10 mice per group; mean ± SEM). (C) NAS and its individual components based on histopathological assessment of H&E-stained liver sections (mean of 5 fields per section, 3 sections per mouse). (n=10 per group; mean ± SEM). (D) Quantification of ORO-, F4/80-, and αSMA- positive areas. ImageJ software was used for quantification of stained areas (mean of 5 fields per section, 3 sections per mouse). (n=10 per group; mean ± SEM). (E) Serum TC and TG levels, and hepatic GSH and SOD levels (n=10 per group; mean ± SEM). *P < 0.05, **P < 0.01, ***P < 0.001; ****P < 0.0001; ns indicates not significant.
