# Supplementary material for: Hepatocyte TIA1 constrains metabolic steatohepatitis by translationally suppressing Srebf1 mRNA in stress granules
Source: Cell Death Dis. 2026 Mar 24;17(1):357. doi: 10.1038/s41419-026-08682-5 (PMC13039281; doi:10.1038/s41419-026-08682-5)
Supplement: Supplementary file 17 — Original Blot [file 41419_2026_8682_MOESM17_ESM.pdf]

Figure1

TIA1

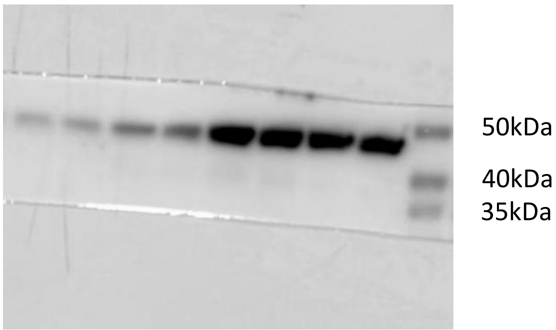

GAPDH

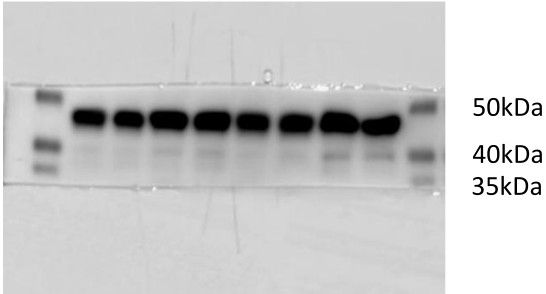

TIA1

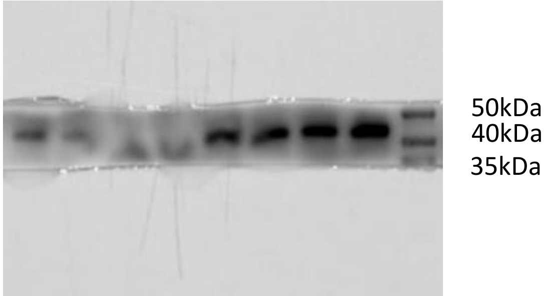

GAPDH

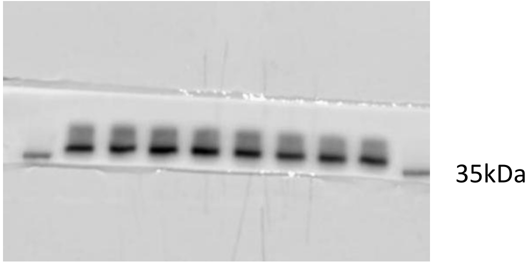

TIA1

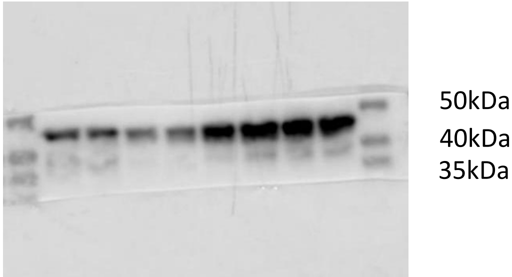

GAPDH

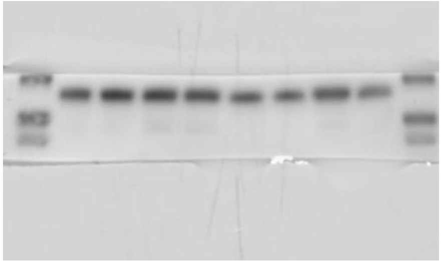

TIA1

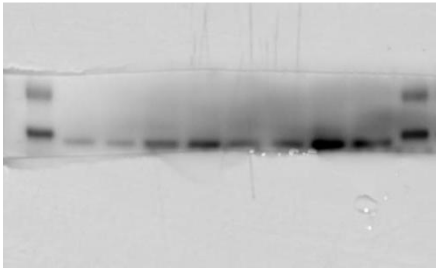

GAPDH

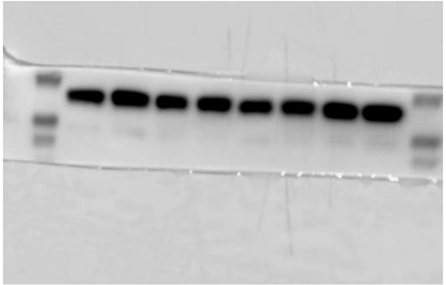

Figure2

FASN

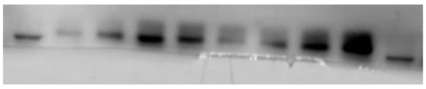

SREBP1

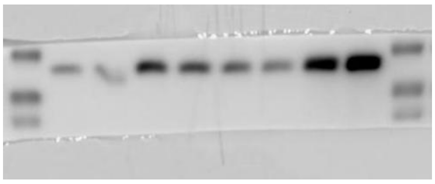

SCD1

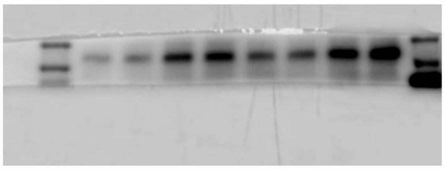

PPARγ

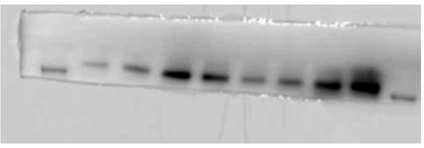

50kDa

GAPDH

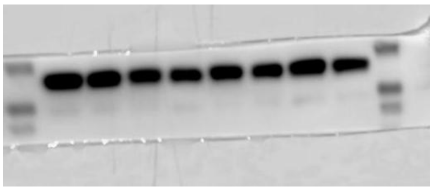

50kDa
40kDa
35kDa

FASN

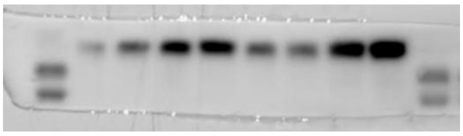

250kDa
150kDa

SREBP1

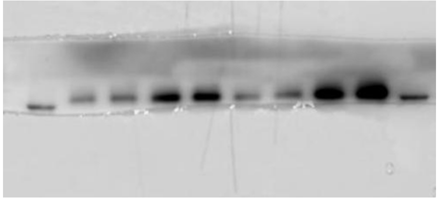

100kDa

SCD1

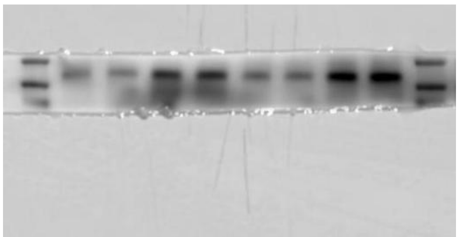

40kDa
35kDa

PPARγ

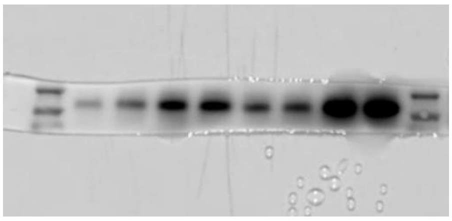

70kDa
50kDa

COL1A1

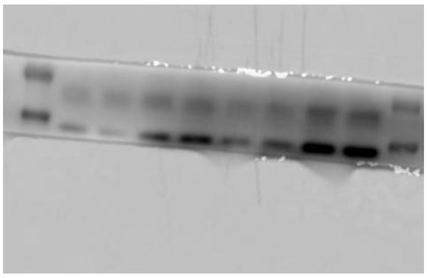

250kDa
150kDa

TGFβ1

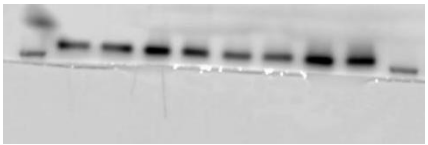

40kDa

αSMA

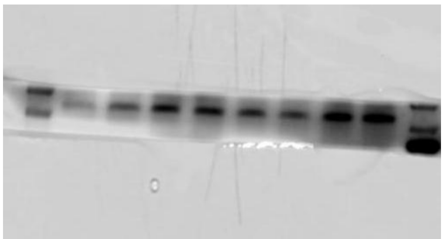

50kDa
40kDa

GAPDH

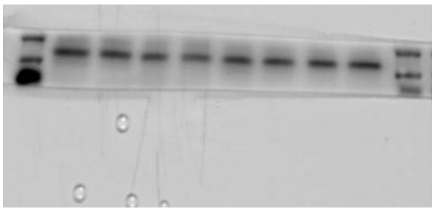

40kDa
35kDa

Figure 3

TIA1

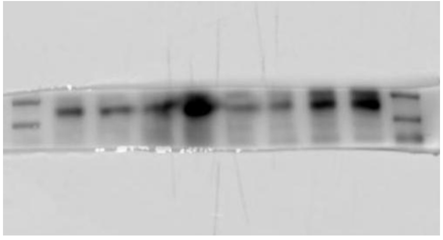

50kDa
40kDa
35kDa

GAPDH

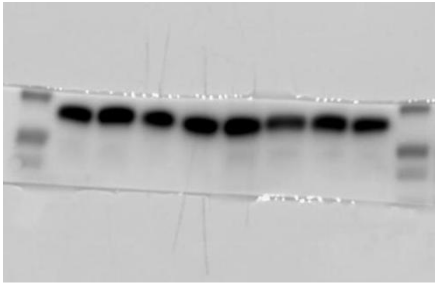

50kDa
40kDa
35kDa

FASN

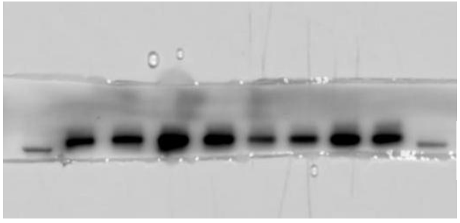

250kDa

SREBP1

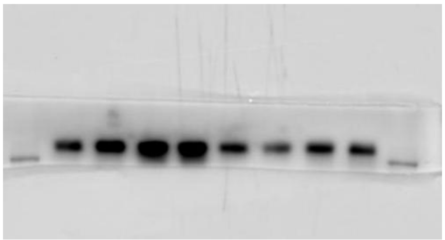

100kDa

SCD1

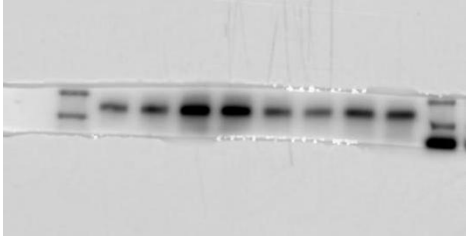

40kDa
35kDa

PPARγ

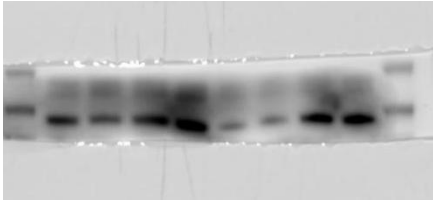

70kDa
50kDa

GAPDH

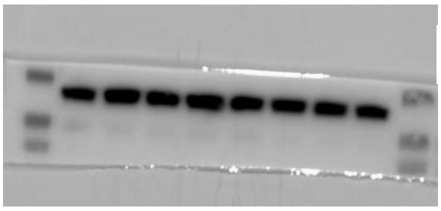

50kDa
40kDa
35kDa

Figure4

TIA1

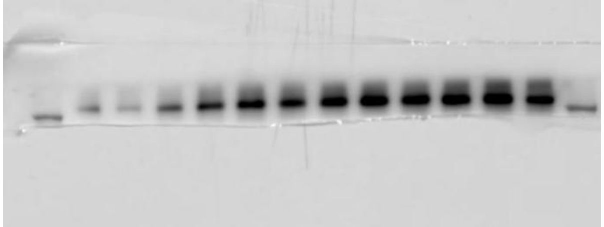

40kDa
35kDa

TIA1

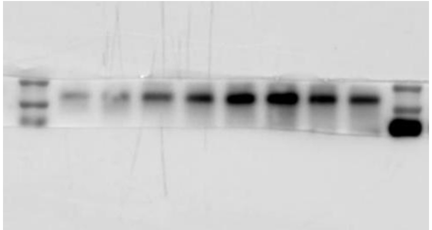

40kDa
35kDa

GAPDH

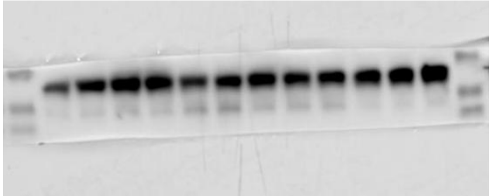

50kDa
40kDa
35kDa

GAPDH

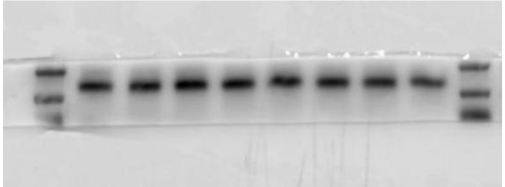

50kDa
40kDa
35kDa

FASN

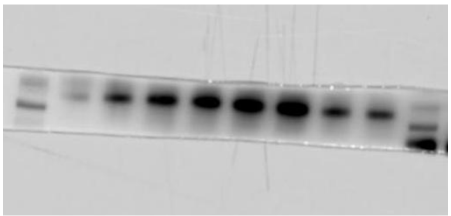

250kDa

SREBP1

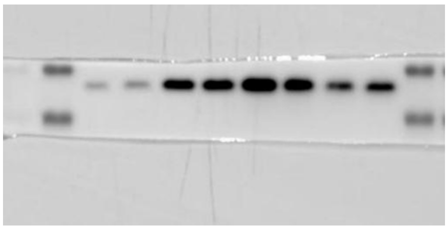

150kDa
100kDa

SCD1

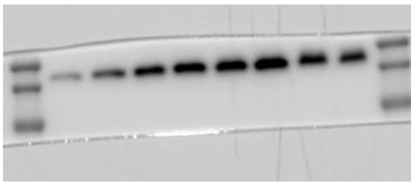

50kDa
40kDa
35kDa

PPARγ

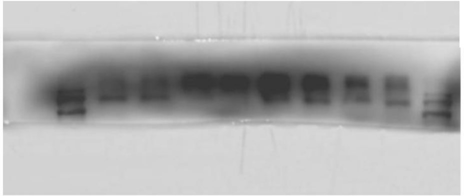

70kDa
50kDa
40kDa

GAPDH

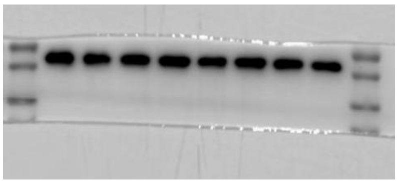

50kDa
40kDa
35kDa

Figure 5

FASN

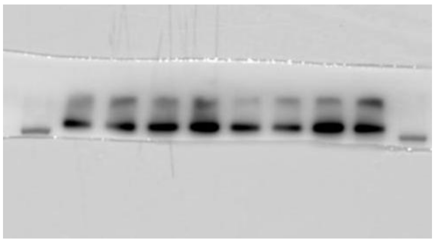

250kDa

SREBP1

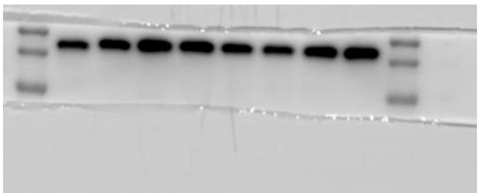

150kDa
100kDa
70kDa

SCD1

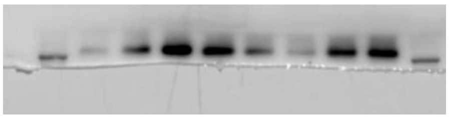

35kDa

PPARγ

70kDa
50kDa

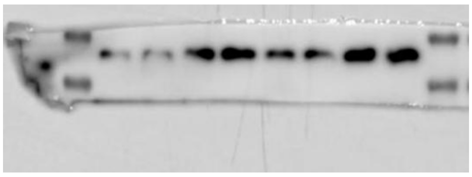

GAPDH

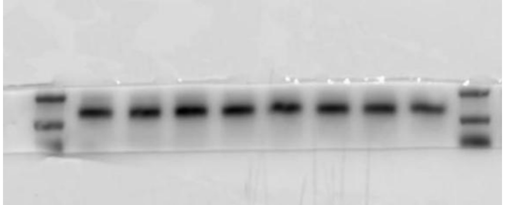

50kDa
40kDa
35kDa

FASN

250kDa

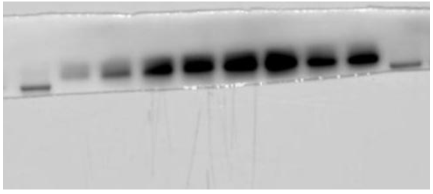

SREBP1

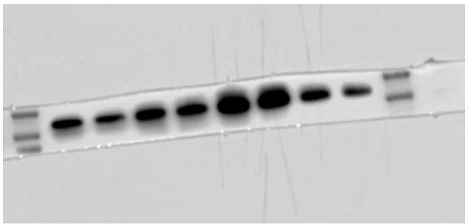

150kDa
100kDa

PPARγ

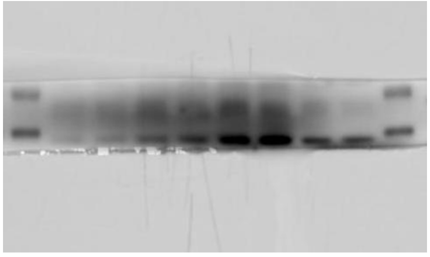

70kDa
50kDa

SCD1

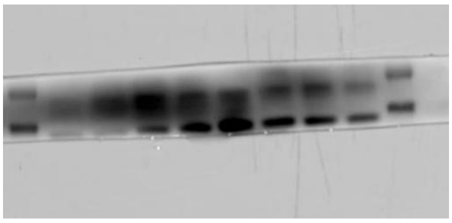

40kDa
35kDa

GAPDH

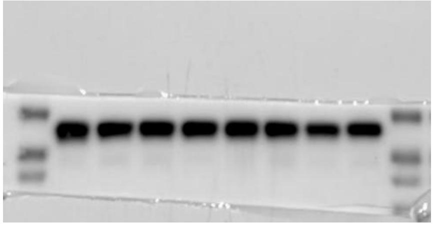

50kDa
40kDa
35kDa

Figure 6

FASN

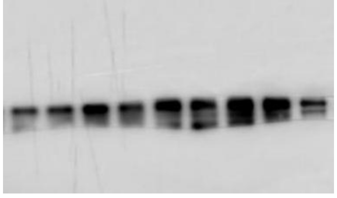

250kDa

SREBP1

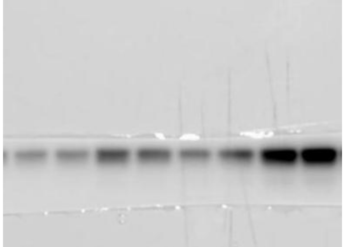

150kDa
100kDa

SCD1

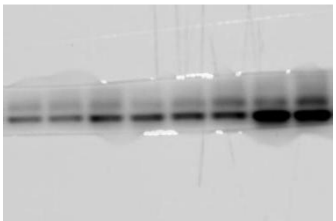

40kDa
35kDa

PPARγ

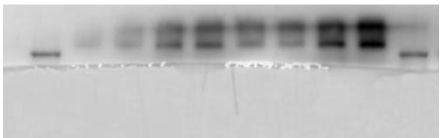

70kDa
50kDa

GAPDH

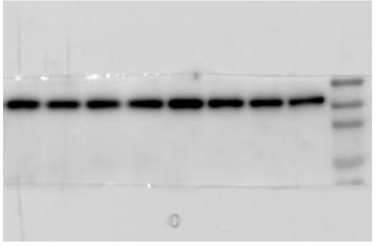

50kDa
40kDa
35kDa

FASN

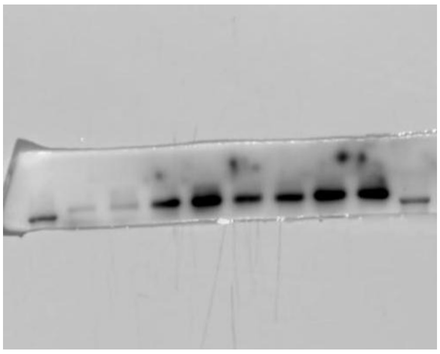

250kDa

SREBP1

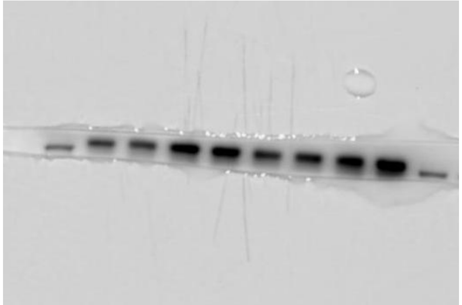

100kDa

SCD1

50kDa
40kDa
35kDa

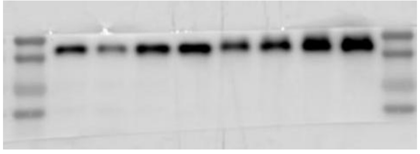

PPARγ

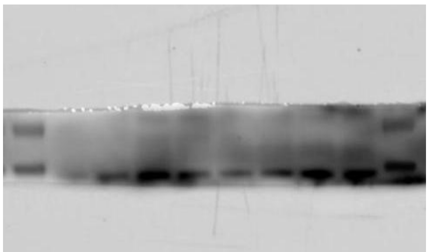

70kDa
50kDa

GAPDH

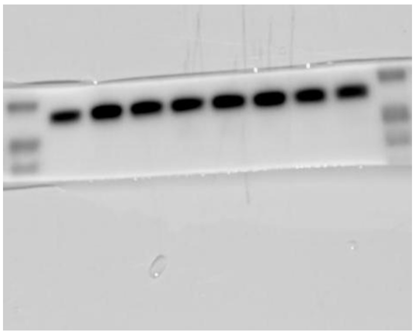

50kDa
40kDa
35kDa

Figure 7

FASN

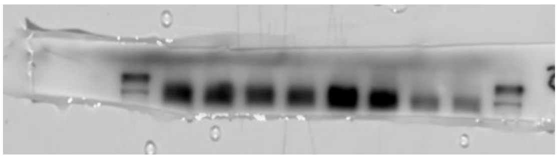

250kDa

SREBP1

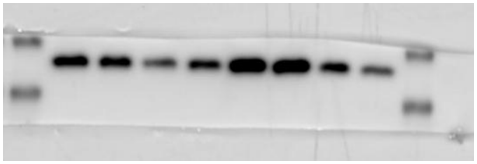

150kDa
100kDa

SCD1

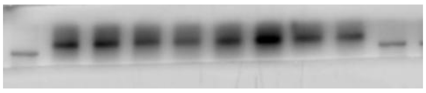

40kDa
35kDa

PPARγ

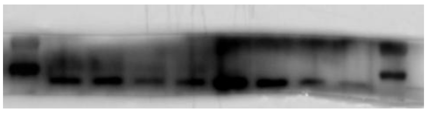

70kDa
50kDa

GAPDH

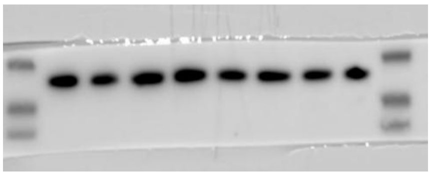

50kDa
40kDa
35kDa

Supplymentary

S2 TIA1

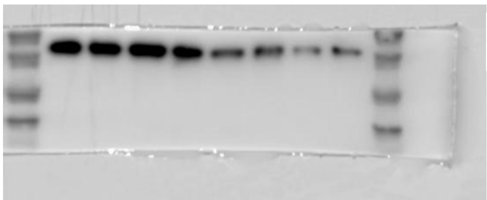

50kDa
40kDa
35kDa

S2 GAPDH

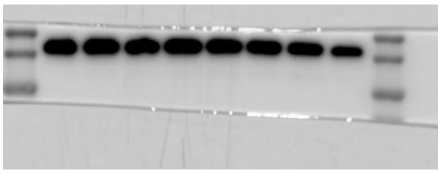

50kDa
40kDa
35kDa

S2 TIA1

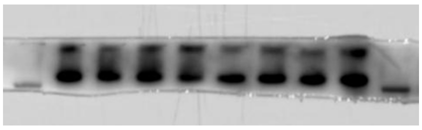

50kDa
40kDa

S2 GAPDH

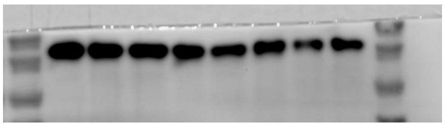

50kDa
40kDa
35kDa

S2 GAPDH

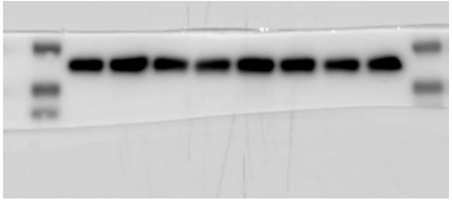

50kDa
40kDa

S2 TIA1

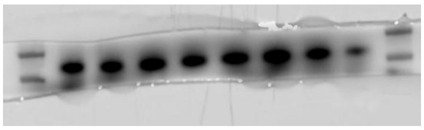

50kDa
40kDa

S4 TGFβ

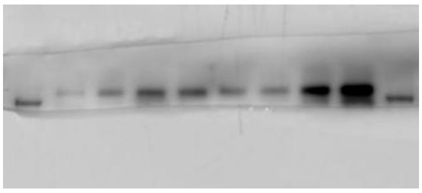

100kDa

S4 COL1A1

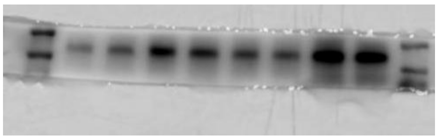

50kDa
40kDa

S4 αSMA

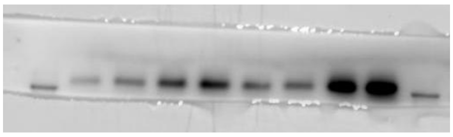

40kDa

S4 GAPDH

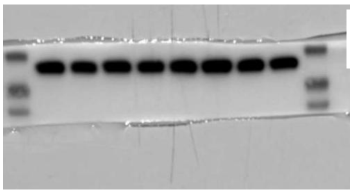

50kDa
40kDa
35kDa

S6 TIA1

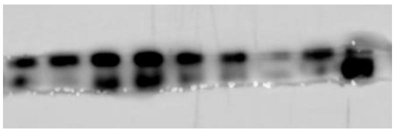

50kDa
40kDa
35kDa

S6 GAPDH

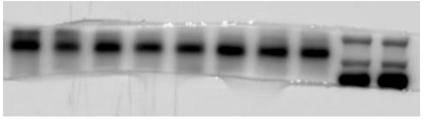

40kDa
35kDa

S7 TIAR

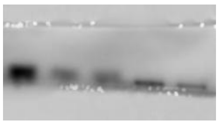

40kDa

S7 GAPDH

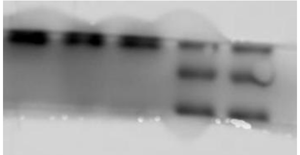

40kDa
35kDa

S7 TIAR

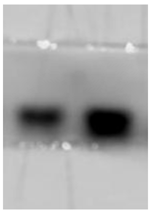

40kDa

S7 GAPDH

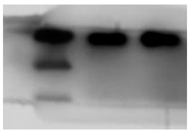

S7 TIA1

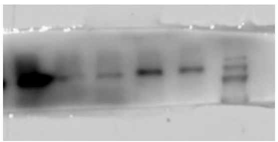

40kDa

S7 GAPDH

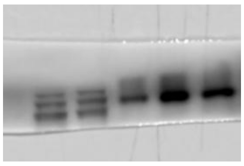

40kDa
35kDa

S7 TIA1

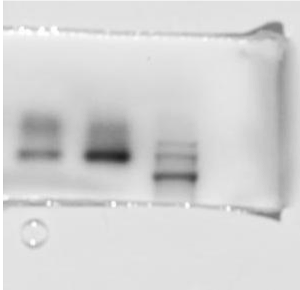

40kDa

S7 GAPDH

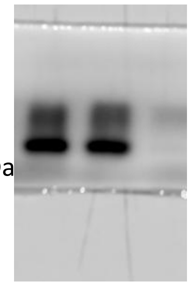

40kDa
35kDa

S7 G3BP1

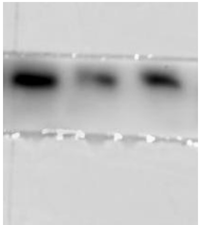

70kDa
50kDa

S7 GAPDH

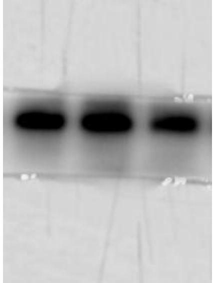

40kDa
35kDa

S7G3BP1

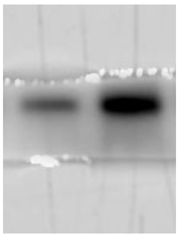

70kDa
50kDa

S7 GAPDH

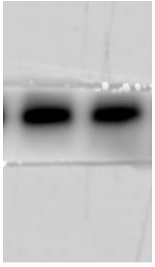

40kDa
35kDa

S7 G3BP1

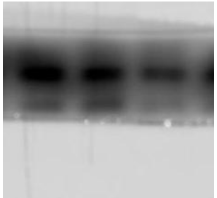

70kDa
50kDa

S7 TIA1

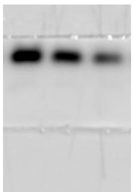

40kDa
35kDa

S7 GAPDH

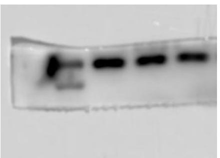

40kDa
35kDa
